# Genome-Wide Association Study for Yield and Yield Related Traits under Reproductive Stage Drought in a Diverse *indica-aus* Rice Panel

**DOI:** 10.1186/s12284-020-00406-3

**Published:** 2020-08-06

**Authors:** Aditi Bhandari, Nitika Sandhu, Jérôme Bartholome, Tuong-Vi Cao-Hamadoun, Nourollah Ahmadi, Nilima Kumari, Arvind Kumar

**Affiliations:** 1 Rice Breeding Platform, International Rice Research Institute, DAPO Box, 7777 Metro Manila, Philippines; 2 Banasthali Vidyapith, Banasthali, 304022 India; 3 Punjab Agricultural University, Ludhiana, 141004 India; 4 CIRAD, UMR, AGAP, Montpellier, France; 5 AGAP, Univ Montpellier, CIRAD, INRA, Montpellier SupAgro, Montepellier, France; 6 IRRI South Asia Regional Centre, Varanasi, 221006 India

**Keywords:** Reproductive-stage drought, Rice, Genetic diversity, Association mapping, Quantitative trait loci

## Abstract

**Background:**

Reproductive-stage drought stress is a major impediment to rice production in rainfed areas. Conventional and marker-assisted breeding strategies for developing drought-tolerant rice varieties are being optimized by mining and exploiting adaptive traits, genetic diversity; identifying the alleles, and understanding their interactions with genetic backgrounds for their increased contribution to drought tolerance. Field experiments were conducted in this study to identify marker-trait associations (MTAs) involved in response to yield under reproductive-stage (RS) drought. A diverse set of 280 *indica-aus* accessions was phenotyped for ten agronomic traits including yield and yield-related traits under normal irrigated condition and under two managed reproductive-stage drought environments. The accessions were genotyped with 215,250 single nucleotide polymorphism markers.

**Results:**

The study identified a total of 219 significant MTAs for 10 traits and candidate gene analysis within a 200 kb window centred from GWAS identified SNP peaks detected these MTAs within/ in close proximity to 38 genes, 4 earlier reported major grain yield QTLs and 6 novel QTLs for 7 traits out of the 10. The significant MTAs were mainly located on chromosomes 1, 2, 5, 6, 9, 11 and 12 and the percent phenotypic variance captured for these traits ranged from 5 to 88%. The significant positive correlation of grain yield with yield-related and other agronomic traits except for flowering time, observed under different environments point towards their contribution in improving rice yield under drought. Seven promising accessions were identified for use in future genomics-assisted breeding programs targeting grain yield improvement under drought.

**Conclusion:**

These results provide a promising insight into the complex genetic architecture of grain yield under reproductive-stage drought in different environments. Validation of major genomic regions reported in the study will enable their effectiveness to develop drought-tolerant varieties following marker-assisted selection as well as to identify genes and understanding the associated physiological mechanisms.

## Background

Drought is one of the major pervasive and limiting factors affecting rice productivity in the Asian-Pacific region under rainfed lowland (46 million hectares; Mha) and upland (10Mha) rice ecosystems (Pandey et al. 2007). Each year, varying intensities of drought stress at different crop stages- seedling, vegetative and reproductive (Price and Courtois 1999; Tripathy et al. 2000; Xu et al. 2011; Nguyen and Bui 2008) affect approximately 34 Mha of rainfed lowland and 8 Mha of upland rice production in Asia (Huke and Huke 1997) as the popular high-yielding green revolution varieties, bred primarily for yield under high input conditions, experience drastic yield reductions even under mild drought stress (O’Toole 1982; Kumar et al. 2008; Torres and Henry 2018). Drought is particularly damaging in the reproductive stage (RS), especially during flowering (Venuprasad et al. 2007; Serraj et al. 2009) reducing both the number of grains per panicle and grain weight and increasing grain sterility. Worldwide, rice production is predicted to be further challenged by an erratic and increasing frequency and severity of drought due to climate change (Wassmann et al. 2009). Combining high productivity with climate resilience is thereby essential to stabilize production by developing climate-smart varieties for adverse ecologies.

Over the years, efforts at International Rice Research Institute (IRRI) for improving yield under drought have documented the effectiveness and response of direct selection for grain yield under drought in upland rice (Venuprasad et al. 2007) and lowland rice (Kumar et al. 2008, 2009), proving the effectiveness of direct selection for grain yield over secondary traits under drought, as a result of which many varieties have been developed (Kumar et al. 2014; Sandhu and Kumar 2017). The different breeding methods followed to improve drought tolerance ranged from marker-assisted breeding (MAB) wherein numerous studies (Fernando and Grossman 1989; Lande and Thompson 1990; Zhang et al. 1992; Howes et al. 1998; Bonnett et al. 2005; Bernardo and Charcosset 2006; Xu and Crouch 2008) used different strategies for increasing favorable alleles in breeding populations for quantitative traits to genomics-assisted breeding (GAB) for improving breeding efficiency by exploiting genome characterization for diversity and function (Varshney et al. 2005, 2014; Abbai et al. 2019) and transgenic breeding (Bhatnagar-Mathur et al. 2008; Yang et al. 2010a ), all of which have helped obtain yield gains and ensured both yield and grain quality improvements over existing varieties. However, complex quantitative traits like grain yield under drought, resistance to other existing and emerging abiotic and biotic stresses are a challenge as they are characterized by interactions of several large and small effect genes for a single trait; of genes for different traits as well as of genes with the environment and genetic backgrounds (Xue et al. 2009; Wang et al. 2012; Kumar et al. 2014; Yadav et al. 2019). To tackle the restrictive applicability of breeding for complex traits, studies conducted have exploited germplasm for desirable variability (Dixit et al. 2014; Mondal et al. 2016; Kumar et al. 2018) and applied precise selection in experiments under different environments and stress intensity levels to emulate farmers’ field conditions.

Genome-wide association study (GWAS) is an important tool in GAB with enormous potential to accelerate breeding for stress tolerance as it enables breeders to make selection based on marker-trait associations (MTAs) as a response to combined effect of all favorable alleles. The transfer of well-characterized genes/ QTLs in breeding programs for varietal development was initially low as the genomic regions of interest were being identified in biparental populations. Subsequently, identification of genomic regions associated with agronomic traits has been accelerated by association mapping in panels with larger genetic background allowing the use of ancestral recombination events, which led to non-random association of alleles at different loci across the genome, and that too at a higher mapping resolution than the biparental linkage analysis (Zhu et al. 2008).

Using different methods, GWAS has been successfully employed in rice for a wide range of traits like yield and yield components (Agrama et al. 2007), harvest index (Li et al. 2012), flowering time (Ordonez Jr et al. 2010) among others. GWAS in diversity panels (unrelated diverse germplasm) including locally adapted breeding material is highly advantageous to breeders (Bernardo 2008) for incorporation of detected beneficial alleles to develop climate-smart varieties (Pauli et al. 2014) as maximum allelic diversity contributing to agronomic traits is identified, as exemplified by Huang et al. (2012) for flowering time and grain yield in worldwide rice germplasm collection; Zhao et al. (2011) and Yang et al. (2010b) for revealing the rich genetic architecture and natural variants of complex traits. Effective population size to select for desired plant type and high yield under upland ecosystem with tolerance to moderate drought stress in lowland ecosystem (Gu et al. 2012) is essential for crossing and successful selection in breeding programs that integrate modern and affordable strategies for varietal development across environments (Kondo et al. 2000; Samejima et al. 2016; Xia et al. 2016).

In the present study, GWAS was performed on ten agronomic traits including grain yield and its components in a diverse set of 280 *indica-aus* accessions to identify the significant MTAs/ QTLs/ genes to study the effect of trait architecture in identifying genomic regions associated with traits of interest across seasons and environments. The analysis was conducted using different model algorithms and results reported include the consistent MTAs detected by two multi-locus methods- SUPER and Farm-CPU. The diverse set used in the study included accessions from both lowland and upland ecosystems with the premise to identify highly drought-tolerant rice accessions in either ecosystem or having at least moderate drought-tolerance in the other to breed for reproductive stage drought-tolerant rice varieties for different growing environments.

## Results

### Phenotypic and Genotypic Characteristics of the Population

#### Distribution, Heritability and Correlation of the Measured Phenotypic Traits

**Fig. 1:**
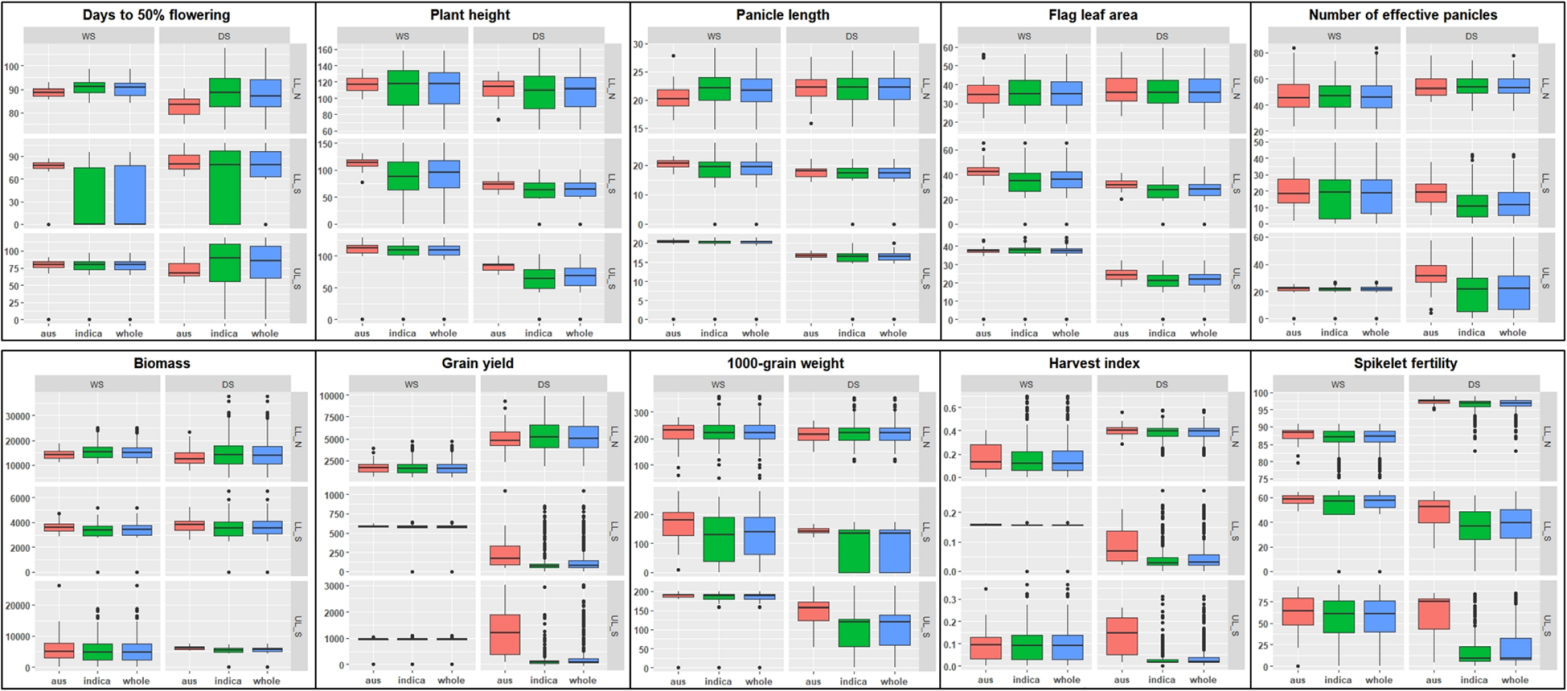
Boxplots of ten phenotypic variables within diverse set. Days to 50% flowering, Plant height (cm), Panicle length (cm), Flag leaf area (cm^2^), Number of effective panicles, Biomass dry weight at maturity (kg ha^−1^), Grain yield (kg ha^-1^), 1000 grain weight (g), Harvest index and Spikelet fertility (percentage ratio by weight) for each of the six experiments (lowland non stress-LL_N, lowland stress-LL_S and upland stress-UL_S) in the two seasons- wet season-WS and dry season-DS. The boxplots are divided into population and subpopulation levels for each trait-whole population level (with all 280 *indica-aus* accessions): 245 accessions representing the four major genetic subgroups of *indica* genetic background and 35 accessions of *aus* genetic background. Within the boxplots, bold line represents the median, box edges represent upper and lower quantiles, and whiskers are 1.5 times the quantile of the data. Open dots represent the outliers

Box-plots of the adjusted means of the ten phenotypic variables - days to 50% flowering (DTF), plant height (PH), panicle length (PL), flag leaf area (FlgLA), number of effective panicles (NBP), biomass at maturity (BMDW), grain yield (GY), 1000-grain weight (TGW), harvest index (HI) and spikelet fertility (SPKFT) under three environments (control condition or non-stress experiment in lowland- LL_N; reproductive-stage drought-stress experiments in lowland- LL_S and upland- UL_S) in two seasons- 2014 wet season (WS) and 2015 dry season (DS) are presented in Fig. 1. The plots depicted at two levels- whole population and genetic subgroup, highlight that the mean phenotypic performances of different levels (whole population with 280 lines, *indica* genetic subgroup with 245 lines and *aus* genetic subgroup with 35 lines) in each environment and season were not significantly different except for DTF, HI, TGW and SPKFT. Overall, trait range was higher in DS than WS; PH, FlgLA, BMDW and NBP exhibited a reasonably symmetric distribution while DTF, GY and SPKFT exhibited skewed distributions across environments and seasons. Under drought, all traits values except for DTF decreased as compared to the LL_N environment. Multidimensional analysis of phenotypic data for WS and DS was performed with experiment-wise data projected on the space defined by the first two axes of factorial discriminant analysis (FDA) using the Y_adj_ values for the 10 agronomic traits. In general, the phenotypic distribution was greater in DS than WS (Additional file 1: Figure S1a). Fisher distances were highly significant (*p* < 0.001) between the experiments of WS and DS. The projection of the ten traits across experiments revealed different degrees of relatedness between the traits measured at different growth stages of life cycle (Additional file 1: Figure S1b). PH, PL, GY, SPKFT and NBP were the major factors affecting about 80% variance explained in both WS and DS. Higher grain yield reduction was observed in DS lowland stress experiment (98%) followed by upland stress experiment (94%) (Additional file 1: Table S1). Significant effects of drought stress were observed on DTF as reflected by early flowering in WS but late flowering in DS for tolerant accessions. Heritability was in medium and high range for all the 10 traits in the two seasons, with relatively higher trait heritability in DS than WS (Table 1). The phenotypic variance was partitioned into different sources of variations using the mixed model analysis. As shown (Table 1), genotype effect contributed significantly to the observed variation for all traits across environments.

**Table 1:** Summary statistics and sources of variation for the ten phenotypic traits under three different environments and two seasons for the diverse set of 280 lines

Trait	Env	Wet Season					Dry Season				
		Mean (SD)	Phenotypic variance				Mean (SD)	Phenotypic variance			
			Fixed	Random		H^**2**^		Fixed	Random		H^**2**^
				Genotype	Residual				Genotype	Residual	
DTF	**LL_N**	90.4 (8.69)	0.36	22.55^NS^	52.22	0.34	87.83 (9.53)	0.12	76.31^d^	23.49	0.75
	**LL_S**	79.66 (6.7)	2.29	37.89^d^	6.02	0.90	88.09 (16.68)	5.96	212.92^d^	68.03	0.85
	**UL_S**	80.28 (9.64)	0.01	55.82^d^	39.41	0.72	93.07 (20.09)	4.78	357.44^d^	51.19	0.93
PH	**LL_N**	113.79 (24.23)	1.18	579.55^d^	42.06	0.93	108.63 (21.2)	0.08	538.47^d^	21.62	0.96
	**LL_S**	102.42 (26.61)	8.70	605.08^d^	104.52	0.92	69.09 (16.67)	3.34E-15	191.30^d^	90.86	0.79
	**UL_S**	110.23 (29.58)	20.55	241.92^NS^	616.22	0.43	71.03 (15.55)	8.56	196.20^d^	46.21	0.88
PL	**LL_N**	21.75 (3.41)	0.01	1042^d^	1.86	0.91	22.15 (3.16)	0.02	9.29^d^	2.05	0.81
	**LL_S**	20.05 (3.12)	0.07	5.65^d^	4.13	0.79	17.87 (3.35)	0.04	5.19^d^	5.88	0.63
	**UL_S**	20.41 (3.22)	0.64	1.64^NS^	8.13	0.16	16.45 (2.09)	0.41	1.45^d^	2.58	0.50
FlgLA	**LL_N**	36.0 (10.92)	0.02	83.89^d^	36.74	0.68	36.73 (9.74)	0.09	98.77^d^	12.34	0.89
	**LL_S**	40.19 (13.46)	1.31	79.15^d^	101.37	0.68	29.85 (9.81)	1.65	43.95^d^	50.54	0.71
	**UL_S**	37.77 (11.95)	6.51	24.81^NS^	111.33	0.19	22.96 (6.04)	1.67	17.56^d^	17.99	0.63
BMDW	**LL_N**	15.57E + 03 (92E + 02)	0.05	3.13E + 07^d^	5.48E + 08	0.34	14.78E + 03 (67.81E + 02)	1.92E + 05	4.23E + 07^d^	9.9E + 06	0.82
	**LL_S**	36.13E + 02 (14.4E + 02)	1.11E + 05	4.86E + 05^a^	15.06E + 05	0.40	36.50E + 02 (15.49E + 02)	3.37E + 04	6.6E + 05^d^	1.75E + 06	0.50
	**UL_S**	54.61E + 02 (56.5E + 02)	2.07E + 05	5.87E + 07^NS^	3.18E + 07	0.35	55.29E + 02 (18.14E + 02)	3.65E + 05	7.29E + 05^NS^	2.34E + 06	0.42
NBP	**LL_N**	46.99 (18.24)	0.03	214.91^b^	126.33	0.70	54.81 (17.02)	1.93	130.23^c^	162.03	0.44
	**LL_S**	20.38 (14.57)	2.38	15.81^NS^	211.05	0.20	14.99 (13.59)	2.91	102.80^d^	83.08	0.76
	**UL_S**	23.32 (16.38)	11.35	0.22^NS^	255.78	0.23	24.84 (14.38)	2.9	136.37^d^	73.28	0.78
GY	**LL_N**	16.66E + 02 (10.03E + 02)	0.05	7.94E + 05^d^	2.28E + 05	0.84	5.21E + 02 (16.8E + 02)	2.41E + 04	3.92E + 06^d^	1.55E + 06	0.68
	**LL_S**	5.73E + 02 (6.28E + 02)	7.79E + 03	3.68E + 04^NS^	3.52E + 05	0.20	1.58E + 02 (2.89 E + 02)	1.4E + 03	4.91E + 04^d^	3.49E + 04	0.74
	**UL_S**	9.42E + 02 (9.24E + 02)	0.02	3.26E + 04^NS^	8.23E + 05	0.13	3.36E + 02 (6.1 E + 02)	2.29E + 09	8.12E + 09^d^	2.52E+ 04	0.91
TGW	**LL_N**	198.13 (77.77)	79.37	2221.13^b^	3824.46	0.37	218.53 (39.82)	112.63	1528.06^d^	191.65	0.89
	**LL_S**	149.2 (80.97)	75.33	0.01^NS^	6481.26	0.20	113.26 (79.5)	158.25	2139.19^d^	4015.32	0.52
	**UL_S**	177.96 (84.55)	96.99	0.01^NS^	7100.32	0.10	83.69 (78.41)	138.69	4111.16^d^	1870.32	0.81
HI	**LL_N**	0.17 (0.15)	0.01	0.001^NS^	0.02	0.30	0.39 (0.11)	2.4E-04	0.006^d^	0.005	0.61
	**LL_S**	0.15 (0.17)	4.8E-06	2.03E-03^NS^	0.03	0.21	0.05 (0.08)	5.2E-05	0.004^d^	0.003	0.76
	**UL_S**	0.11 (0.1)	0.001	8E-05^NS^	0.01	0.14	0.05 (0.09)	8.2E-05	0.006^d^	0.003	0.82
SPKFT	**LL_N**	86.81 (9.64)	0.02	27.88^a^	64.19	0.80	96.41 (3.25)	0.51	6.83^d^	4.22	0.78
	**LL_S**	57.49 (29.94)	29.36	106.46^NS^	762.52	0.22	47.03 (31.33)	81.07	315.92^d^	615.27	0.60
	**UL_S**	61.19 (29.59)	0.01	86.21^NS^	892.48	0.15	25.19 (34.85)	27.01	809.67^d^	349.53	0.81

*SD* standard deviation, *Env* environment (lowland non-stress, LL_N; lowland stress, LL_S and upland stress, UL_S), *Fixed effects* block (rep), *Random effects* genotype and residual, *H*^*2*^ broad sense heritability for single environment analysis, *DTF* days to 50% flowering, *PH* plant height (cm), *PL* panicle length (cm), *FlgLA* flag leaf area (cm^2^), *BMDW* biomass dry weight at maturity (kg ha^−1^), *NBP* number of effective panicles, *GY* grain yield (kg ha^−1^), *TGW* 1000 grain weight (g), *HI* harvest index and *SPKFT* spikelet fertility from each of the six experiments in two seasons (wet and dry). ^a^significant at 5%, ^b^significant at 1%, ^c^ significant at 0.1%, ^d^significant at 0.01% levels and NS- Non-significant

**Fig. 2:**
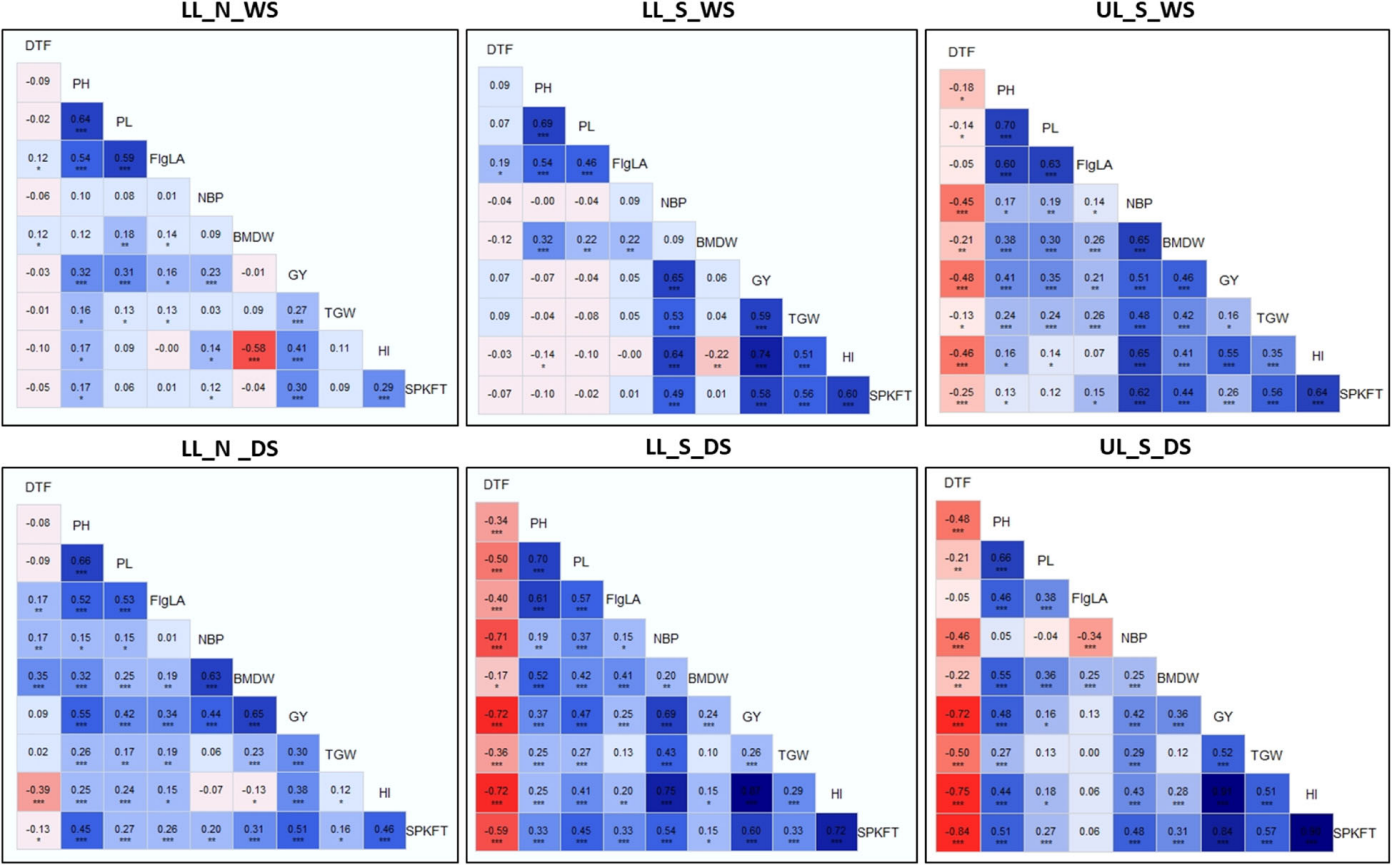
Plots of Pearson’s *r-*values showing correlation between each of the ten traits in each of the six experiments (LL_N_WS-lowland non-stress 2014WS, LL_N_DS-lowland non-stress 2015DS, LL_S_WS-lowland stress 2014WS, LL_S_DS-lowland stress 2015DS, UL_S_WS-upland stress 2014WS and UL_S_DS-upland stress 2015DS). Blue color indicates positive correlation and red color indicates negative correlation among different traits, with color intensity variance depicting the strength of correlation. *significant at < 0.05 level, **significant at <0.01 level, *** significant at < 0.001 level, blank for non-significant

Trait correlation within DS and WS was studied for the 10 traits for each experiment (Fig. 2). As expected, DTF was negatively correlated to grain yield and yield-related traits under drought stress in both seasons (in the range of − 0.03 to − 0.72). The grain yield related traits such as NBP, BMDW, TGW and HI as well as PL showed significant positive correlation with GY across seasons and experiments; for the LL_S and UL_S environments, this correlation was positive (0.06–0.91) while for LL_N condition, the range of this correlation was from 0 to 0.51. Overall, the correlation was strong in DS as compared to WS. Seasonal variation was observed in trait correlations between environments- while in DS, the correlations between all three environments was significantly positive for all traits (range of − 0.18-0.72), the correlations between two drought environments was negligible in WS, in the range of − 0.11-0.12 (Additional file 1: Figure S2).

### Phenotypic Effect of the Drought Stress on Tolerance and Susceptibility in the Diverse Set

Differences in response to RS drought at agronomic level are presented in Table 1. Significant effect of treatments (control and RS drought stress), environments (lowland and upland) and seasons (WS and DS) was observed on the traits measured in the present study. The DTF increased under both, lowland and upland reproductive-stage drought stress in DS (from 87.83 days in LL_N to 88.09 days and 93.07 days in LL_S and UL_S, respectively) while in WS, early flowering was observed under both stress environments (from 90.4 days in LL_N to 79.66 days and 80.28 days under LL_S and UL_S, respectively). Moreover, as shown in Fig.1, effect of subpopulation on DTF was significant in LL_S in WS and UL_S in DS. In both WS and DS, plant height, panicle length and spikelet fertility reduced significantly under the two stress environments. The extent of reduction was more under very severe stress levels realized in DS (Additional file 1: Table S1) under both LL_S and UL_S, where PH was reduced by 39.54 cm and 37.6 cm, respectively; PL was reduced by 4.28 cm and 5.7 cm, respectively and SPKFT was reduced by 49% and 71%, respectively. SPKFT was quite variable at subpopulation level as well, especially in DS under LL_S and UL_S; while under LL_S, SPKFT at population level (280 accessions) and in the *indica* subset (245 accessions) was reduced to 47% and 40%, respectively, it was interestingly greater in the *aus* subset (35 accessions) at 60% and similarly, these figures under UL_S in DS were 25%, 16% and 70%, respectively. Yield was markedly reduced under RS drought in both lowland and upland stress environments and this was reflected in the various component trait measurements, especially in NBP and SPKFT. The reduction in GY under LL_S and UL_S was more in DS; in WS, GY was reduced by 64% in LL_S and 57% in UL_S while in DS, it was reduced by 98% in LL_S and 93% in UL_S.

Consequently, analysis of variance for all traits measured in the present study revealed high significant differences between the two treatments (control and RS drought stress) for all traits (Table 2). However, the growing environment (lowland and upland) did not significantly affect the PL and SPKFT measurements while the growing season (WS / DS) had the least effect on the differences in genotypic trait performances for DTF, NBP and BMDW. The drought susceptibility index (DSI) calculated for each genotype under both lowland and upland environments for the selected traits (Additional file 1: Table S2). indicated that the accessions with high levels of drought tolerance and good recovery ability (recorded by leaf rolling scores) could still produce some grains even under severe level of drought stress at reproductive stage. The number of tolerant genotypes differed by DSI under different growing environments.

**Table 2:** Analysis of variance (F-values) for grain yield, yield components and agronomic traits among treatments, conditions and seasons for the diverse set of 280 lines

Sources of variations	Df	DTF	PH	PL	FlgLA	NBP	BMDW	GY	HI	TGW	SPKFT
**Treatment**	1	348.69^c^	497.02^c^	403.82^c^	227.76^c^	3136.14^c^	4062.87^c^	4524.94^c^	1856.72^c^	652.32^c^	2508.07^c^
**Condition**	1	173.25^c^	19.93^c^	0.5^NS^	8.07^b^	58.63^c^	76.61^c^	30.86^c^	12.14^c^	21.2^c^	2.61^NS^
**Season**	1	4.35^a^	223.68^c^	40.59^c^	155.89^c^	7.19^b^	1.31^NS^	430.86^c^	50.45^c^	51.74^b^	166.89^c^
**Treatment:Season**	1	19.73^c^	67.91^c^	29.64^c^	94.02^c^	84.76^c^	4.69^a^	1951.55^c^	1045.64^c^	100.08^c^	241.19^c^
**Condition:Season**	1	42.3^c^	11.62^c^	19.1^c^	30.39^c^	18.88^c^	0.85^NS^	3.51^.^	9.1^b^	39.13^c^	57.03^c^

Sources of variation analysed using two treatment levels (control, stress); two growing conditions (lowland, upland) and two seasons (wet and dry) for ten traits at ^a^significant at 5%, ^b^significant at 1%, ^c^ significant at 0.1% levels and NS- Non-significant levels

### Population Structure Analysis

The density, distribution of allele frequencies and heterozygosity of the working set of 215,250 loci (215 k) is summarized in Additional file 1: Table S3. For this 215 k SNP set, there is an uneven distribution of markers along the genome. Average density of markers per Mb of the genome was 503 SNPs. High-density marker regions were observed on chromosomes 2 and 4, with a magnitude of about 493 and 438 SNPs per Mb and on chromosome 11 with a magnitude of about 1231 SNPs between 22 and 27 Mb region. The distribution of markers along each chromosome is depicted as heatmap in Additional file 1: Figure S3.

For the 215 k marker set, average observed heterozygosity (Ho) at the accession level was 0.86% with a minimum and maximum of 0.4% and 4.81%, respectively. The distribution of Ho varied among the 12 rice chromosomes in the working set and with an average of 0.36%, chromosomes, more heterozygous calls were mainly on chromosomes 7, 9, 10 and 12 (Additional file 1: Table S3).

**Fig. 3:**
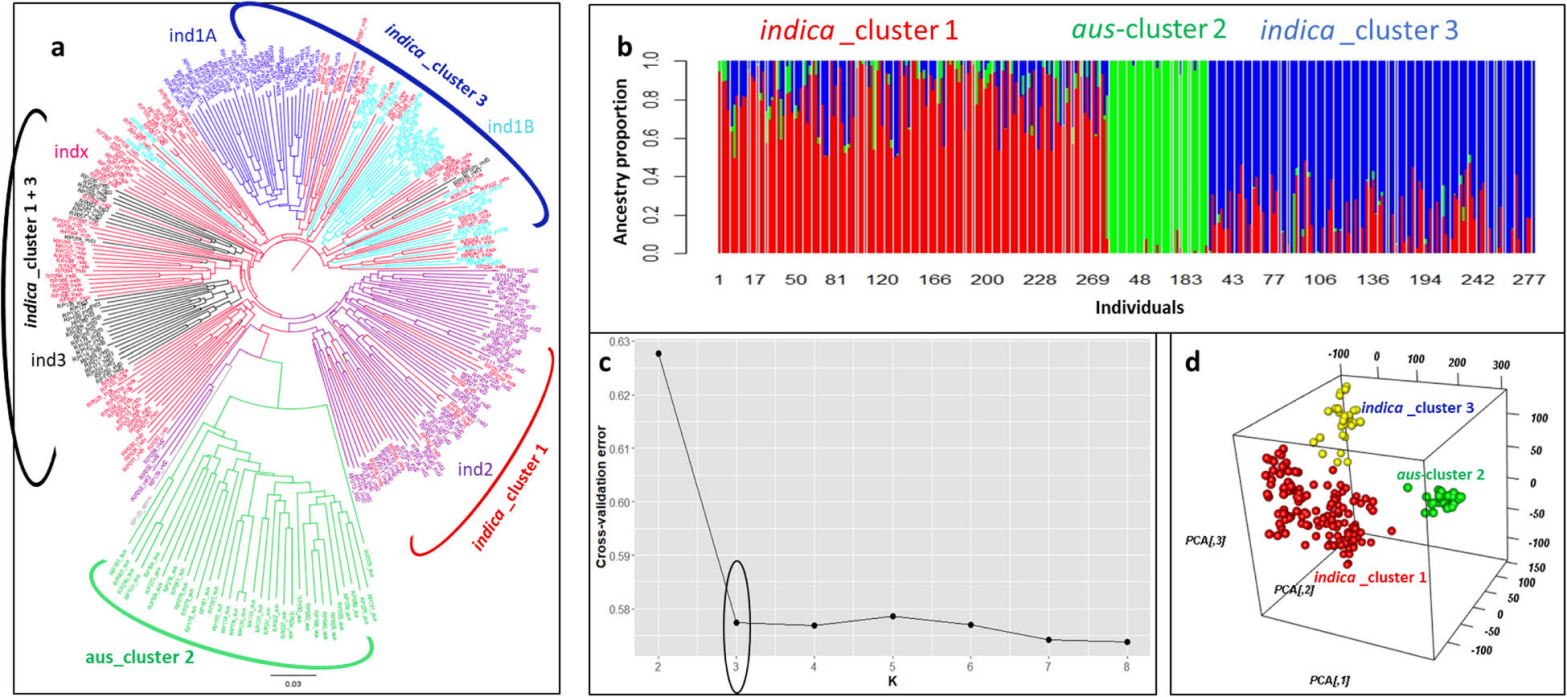
Genetic relatedness and population structure of the diversity panel; **a** Genetic diversity depicted through the unweighted neighbour-joining tree method within the population as *indica (ind*1A, *ind*1B, *ind*2, *ind*3 and *indx)* and *aus* accessions, as established in the 3k rice genome project; **b** Ancestory proportions from ADMIXTURE analysis represented for k = 3, the optimal with the lowest cross-validation error for K = 2–8 and visualized using R/pophelper package; **c** Cross-validation error for k = 2–8 from ADMIXTURE analysis; **d** Variation captured by the PCs using R/GAPIT corresponding to the 3 clustered distribution along the first three PC components

Phylogenetic diversity illustrated by the unweighted NJ tree (Fig. 3a) validated the population structure analysis of diversity panel clustering into three main groups (Fig. 3b): Cluster-I with *indica* subgroups of *ind*2, *ind*3 and *ind*x, Cluster-II *aus* background and Cluster-III with *indica* subgroups of *ind*1A, *ind*1B, *ind*3 and *ind*x genetic background. The ideal K value with the least cross-validation error detected by the population structure analysis was determined as 3 (Fig. 3c). PCA output of R/GAPIT illustrated accessions clustering in 3 distinct groups when plotted against the first three PC components (Fig. 3d). The decay of linkage disequilibrium along the physical distance is depicted in Additional file 1: Figure S4. The rapid decay of r^2^ of 0.145 between markers with distance of 0–25 kb to half of the initial level was observed around 200 kb.

#### Effect of Trait Architecture and Heritability on MTA Identification Across Seasons and Environments

The phenotypic variance (PV) captured for the ten traits by the GWAS models reveal that severity of drought stress realized in the experiment and correlation of trait to grain yield cause significant differences in variations across seasons. For DTF, high heritability and negative correlation to GY under reproductive-stage drought in upland environment and zero to negligibly positive correlation in lowland environment, the PV ranged from 15 to 29% in the wet season while in the dry season, it was between 19 and 42% across lowland and upland environments. Similarly, for PH which is another highly heritable trait, the PV ranged uniformly between 34 and 53% across seasons, environment and stress. However, for GY, highly heritable but polygenic trait, variation between wet and dry season was quite apparent. While PV ranged from 5 to 17% only in WS, it was in range of 12–55% for DS. Similarly, for some yield related traits, NBP (55–63%), PL (at least 80% in LL_N and UL_S and 6–29% in LL_S) and FlgLA (at least 80% in non-stress and WS and less than 15% in DS stress), the model explained significant variance for the traits. However, for traits with either low heritability or narrow range of phenotypic values like BMDW (6–27%), HI (5–17%), TGW (5–16%) and SPFKT (5–30%), very minimal phenotypic variance was captured by markers across environments and seasons.

#### GWAS Identified Significant Genomic Regions Associated with Yield and Yield Components under Different Growing Environments for RS Drought

**Fig. 4:**
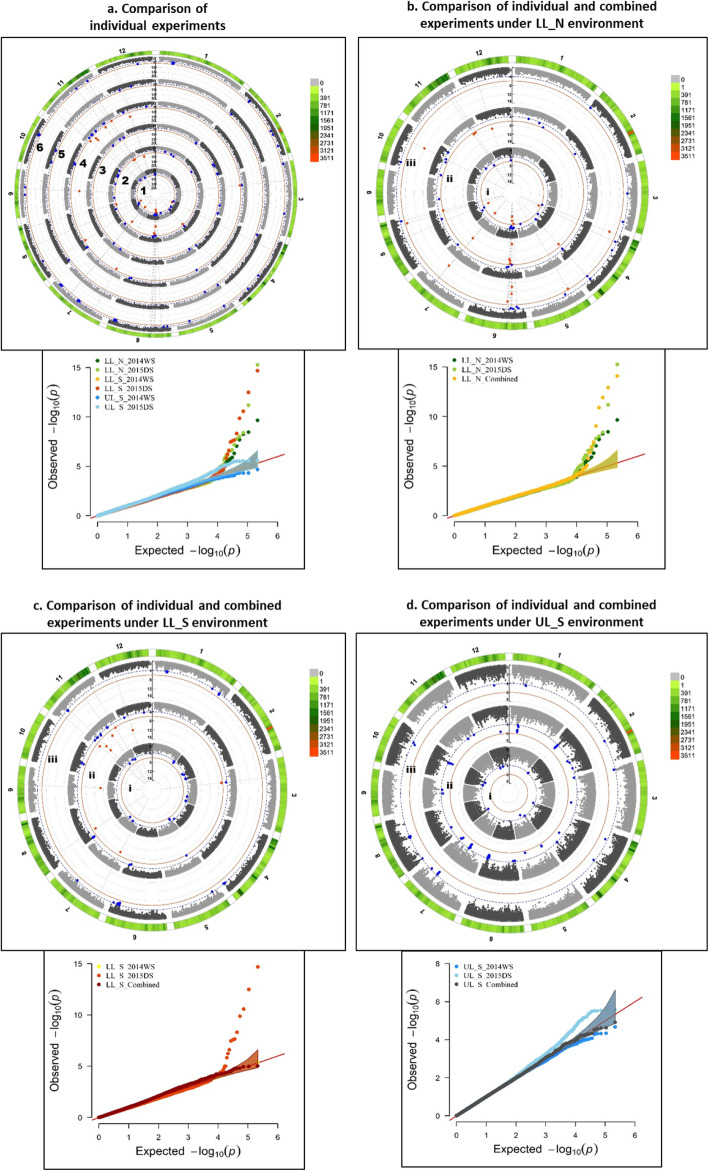
Circular manhattan plot and qq plot for Days to 50% flowering (DTF). **a.** Each of the six experiments (from centre of the plot- 1. lowland non-stress 2014WS(LL_N_WS); 2. lowland non-stress 2015DS (LL_N_DS); 3. lowland stress 2014WS (LL S WS): 4. lowland stress 2015DS (LL_S_DS); 5. upland stress 2014WS (UL_S_WS) and 6. upland stress 2015DS (UL_S_DS) for *p*-values obtained using Farm-CPU method at two significance thresholds of 1e-4 (blue) and 1e-6 (red). The outermost ring depicts the SNP distribution in the 215,250 SNP working set. Individual experimental results are compared to the combined analysis for each of the three environments- **b.** LL_N, **c**. LL_S and **d**. UL_S, to detect seasonal variation between WS and DS, from centre of the plot- i. WS, ii. DS and iii. Combined analysis

**Fig. 5:**
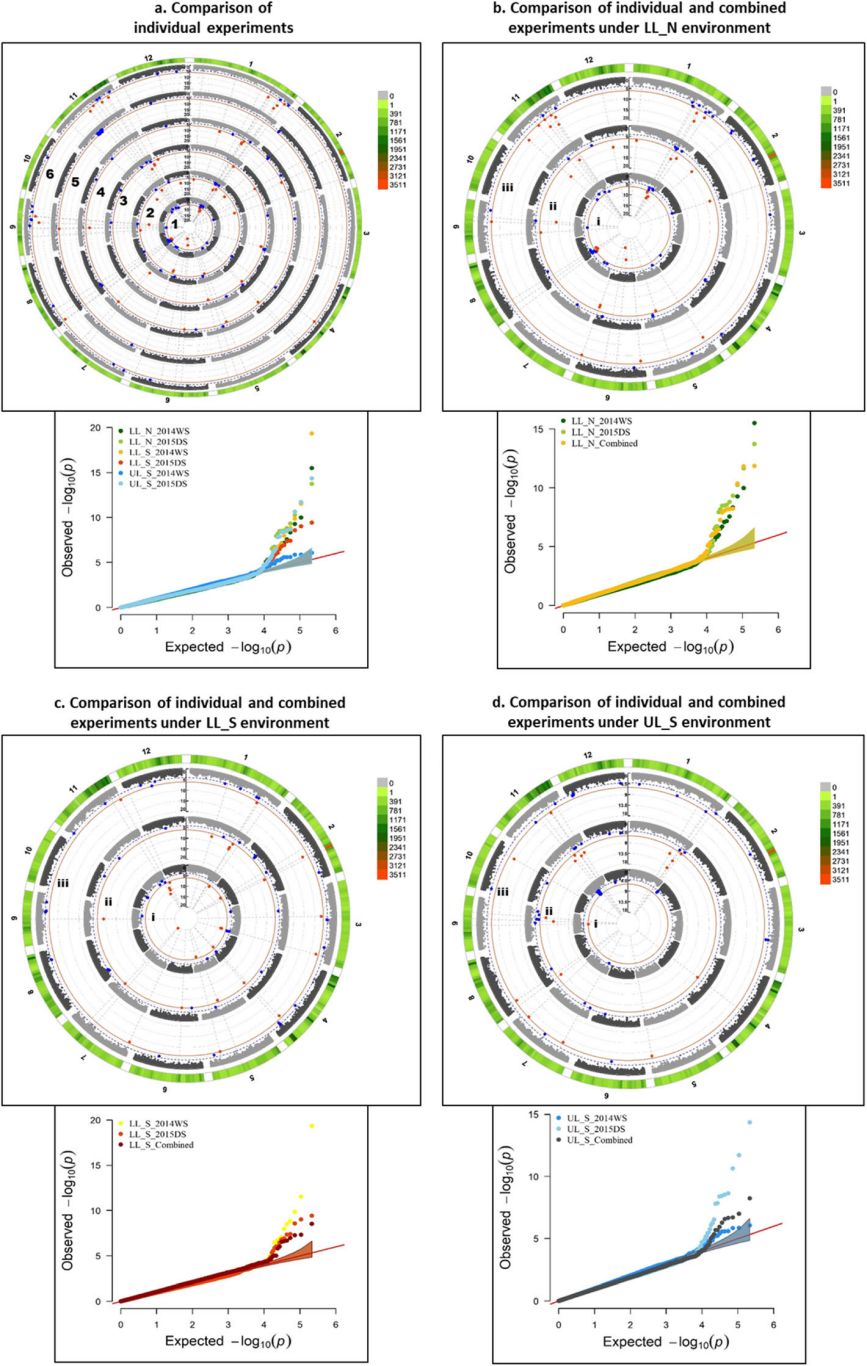
Circular manhattan plot and qq plot for Plant height (PH)- **a**. each of the six experiments (from centre of the plot- 1. lowland non-stress 2014WS (LL_N_WS); 2. lowland non-stress 2015DS (LL_N_DS); 3. lowland stress 2014WS (LL_S_WS); 4. lowland stress 2015DS (LL_S_DS); 5. upland stress 2014(UL_S_WS) and 6. upland stress 2015DS (UL_S_DS) for *p*-values obtained using Farm-CPU method at two significance thresholds of 1e-4 (blue) and 1e-6 (red). The outermost ring depicts the SNP distribution in the 215,250 SNP working set. Individual experimental results are compared to the combined analysis for each of the three environments- **b.** LL_N, **c**. LL_S and **d.** UL_S, to detect seasonal variation between WS and DS, from centre of the plot-i. WS, ii. DS and iii. Combined analysis

**Fig. 6:**
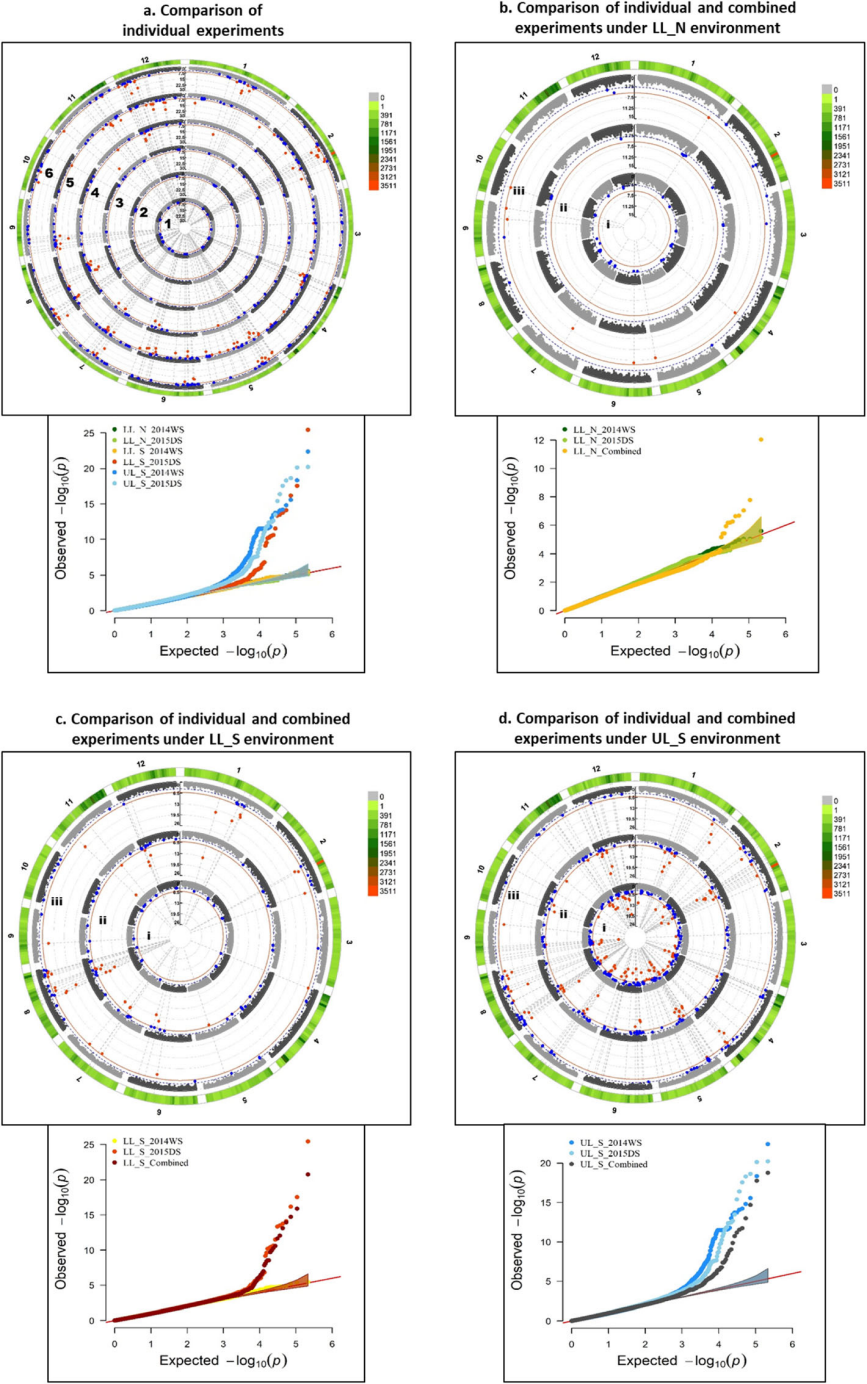
Circular manhattan plot and qq plot for Grain yield (GY)- **a.** each of the six experiments (from centre of the plot- 1. lowland non-stress 2014WS (LL_N_WS); 2. lowland non-stress 2015DS (LL_N_DS); 3. lowland stress 2014WS (LL-S_WS); 4. lowland stress 2015DS (LL_S_DS); 5. upland stress 2014WS (UL_S_WS) and 6. upland stress 2015DS (UL_S_DS) for *p*-values obtained-using Farm-CPU method at two significance thresholds of 1e-4 (blue) and 1e-6 (red). The outermost ring depicts the SNP distribution in the 215,250 SNP working set. Individual experimental results are compared to the combined analysis for each of the three environments- **b.** LL_N, **c**. LL_S and **d**. UL_S, to detect seasonal variation between WS and DS, from centre of the plot-i. WS, ii. DS and iii. Combined analysis

Several significant MTAs and QTLs were identified in the present study for the 10 traits. Among the 219 significant MTAs identified in the study, 95 were associated with grain yield across different environments, seasons and stress levels, 20 with DTF, 34 with PH, 8 with BMDW, 13 with NBP, 25 with HI, 10 with TGW and 20 with SPKFT while no significant association was detected for PL and FlgLA (Additional file 1: Table S4). Circular manhattan plots and qq-plots for MTAs detected using two *p*-value thresholds (1e-6 and 1e-4) to draw out common regions associated with trait across seasons and environments at season-level (WS and DS) and combined-season level are presented in Figs. 4, 5, 6 for DTF, PH, GY, respectively and in Additional file 1: Figure S5 a-g for PL, FlgLA, BMDW, NBP, HI, TGW and SPKFT.

Among the 16 identified QTLs (Additional file 1: Table S4), four QTLs (*qGY*_*1–1*_, *qGY*_*1–2*_, *qPH*_*1–1*_ and *qPH*_*1–2*_) showed consistent effect across both seasons and under different environments; two QTLs showed consistent effect under lowland stress (*qBMDW*_*8–1*_ and *qDTF*_*11–1*_), six QTLs under upland stress (*qGY*_*2–1*_, *qGY*_*2–2*_, *qGY*_*5–1*_, *qGY*_*5–2*_, *qPH*_*9–1*_ and *qGY*_*12–1*_), two QTLs under both lowland non-stress and stress (*qSPKFT*_*9–1*_ and *qBMDW-NBP*_*9–1*_) and two under lowland non-stress across seasons (*qDTF*_*6–1*_ and *qDTF*_*6–2*_). Significant MTAs were reported for GY on chr 1 and 12 under LL_N, on chr 1, 2, 5, 6, 7, 8, 11 and 12 under LL_S, while on chr 1, 2, 4, 5, 6, 7, 8, 11 and 12 under UL_S. Out of these, consistent across experiment level and combined level were on chr 10 for LL_N, on chr 1, 7, 8 and 12 for LL_S and on chr 2, 5, 7 and 8 for UL_S. In about 0.403 Mb interval region on long arm of chr 1 and 4.27 Mb interval region on long arm of chr 2, MTAs were found to be associated with GY under non stress and reproductive stage drought stress conditions for both lowland and upland across seasons, in previously reported major grain yield QTLs under drought– *qDTY*_*1.1*_*and qDTY*_*2.3*_. Three SNPs in a region of 5.06 Mb interval on long arm of chr 11 are reported to be linked with reproductive stage drought stress under lowland and upland conditions across seasons from this study. The 0.941 Mb interval region below centromere on chr 12 showed association with GY under different level of stresses in upland environment (Fig. 6). Under different environmental stresses, the MTAs for DTF were reported on chr 6 (7611279–7,749,410 bp, 9,539,728–10,371,528 bp), chr 7 (19598023–20,159,780 bp), chr 11 (6525213–7,215,940 bp) and chr 12 (7712803–9,203,018 bp). Comparison of experiment level and combined analysis showed consistent effect of MTAs for DTF on chr 6 (7611279–7,749,410 bp) under lowland non-stress and on chr 11 (6525213–6,602,990 bp) for lowland stress (Fig. 4). The long arm of chr 1 (33418648–34,400,345 bp, 37,960,019–39,044,781 bp), chr 3 (33600040–33,600,989 bp), chr 6 (30802585–30,807,826 bp), chr 9 (13423222–16,154,337 bp) and chr 11 (20143839–24,761,315 bp, 25,597,507–28,789,891 bp) was observed to be associated with plant height trait under different environments, stress levels and seasons, with MTAs on chr 1 consistent at both individual experiment and combined levels (Fig. 5). Some SNPs such as S5_352058, S5_4140355, S5_4266313, S8_857745, S9_19316065 and S9_20944019 with very high and almost similar levels of significance were associated with more than one grain yield and yield related traits. However, the SNP S1_3440034 and S12_1642245 were associated with PH and GY, respectively under different environmental stresses.

#### Subpopulation Specific GWAS for Grain Yield under Different Growing Environments and Seasons

**Fig. 7:**
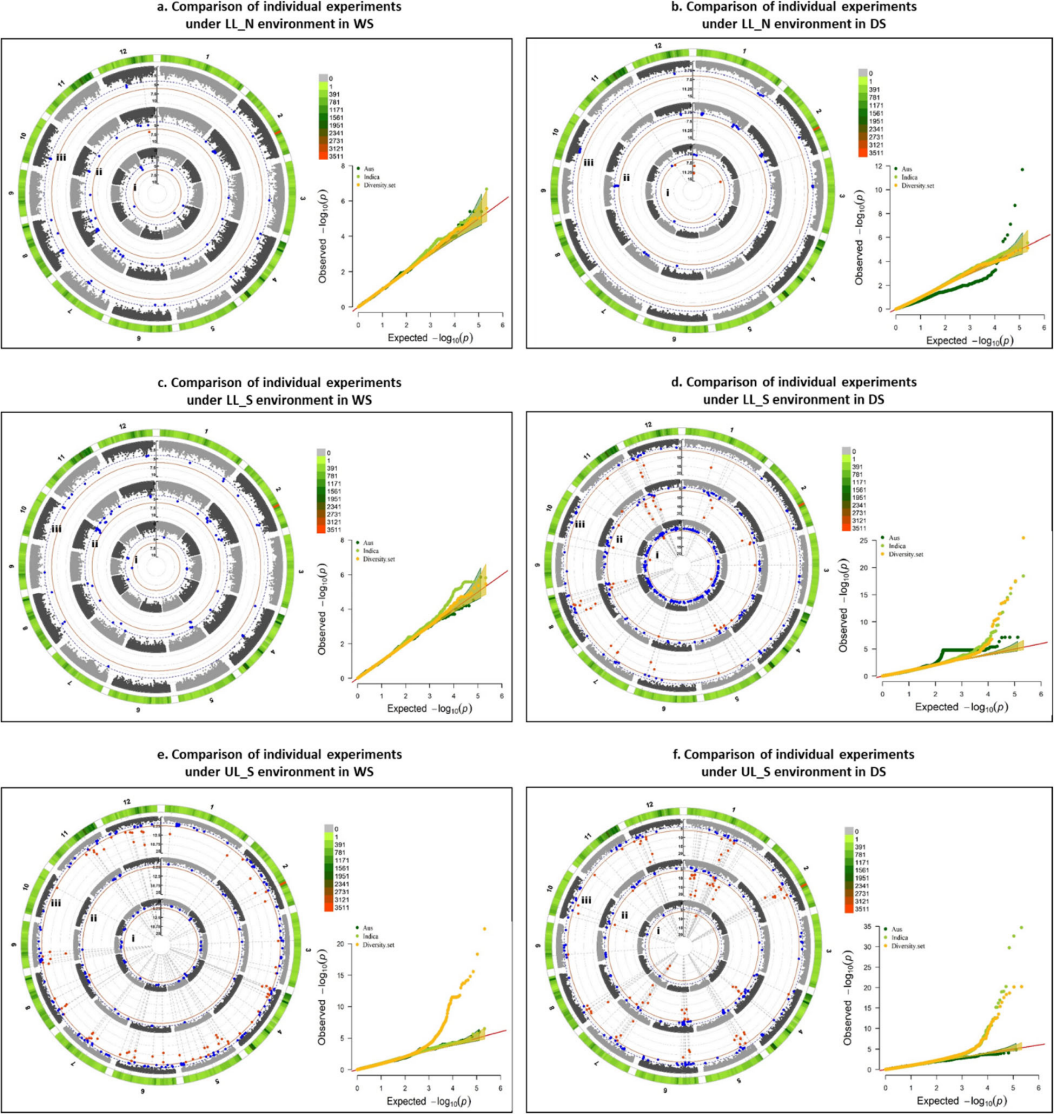
Circular manhattan plot and qq plot for Grain yield (GY) for- **a**. lowland non-stress 2014WS (LL_N_WS); **b**. lowland non-stress 2015DS (LL_N_DS); **c**. lowland stress 2014WS (LL_S_WS); **d**. lowland stress 2015DS (LL_S_DS); **e**. upland stress 2014WS (UL_S_WS); **f**. upland stress 2015DS (UL_S_DS) divided into two levels- subgroup level with **i**. *aus* lines and **ii**. *indica* lines and **iii.** comlpete diversity set of 280 lines for *p*-values obtained using Farm-CPU menthod at two significance thresholds of 1e-4 (blue) and 1e-6 (red). The outermost ring depicts the SNP distribution in the 215,250 SNP working set; to explore the effect of population structure in detection of significant marker trait associations

*Aus*-type rice is closely related to *indica*-type rice but constitutes a distinct genetic group (McNally et al. 2009). The superiority of *aus* accessions over *indica* accessions in the present study was established by the sub-population based GWAS performed for GY wherein upland-adapted *aus* accessions yielded consistent yields under RS drought, especially in DS (Additional file 1: Table S5). While significant loci associated with GY under different environments and seasons at complete diversity set level (280 lines) corresponded to those detected at *aus* subpopulation level (35 accessions) (as shown in Fig.7), 25 additional MTAs were detected at *indica* subpopulation level (245 accessions) in DS- mainly on chr 1 and 11 under LL_S (Fig. 7d) and on chr 1 and 4 under UL_S environment (Fig. 7f).

#### Candidate Gene Analysis for Drought Tolerance under Different Growing Environments

**Fig. 8:**
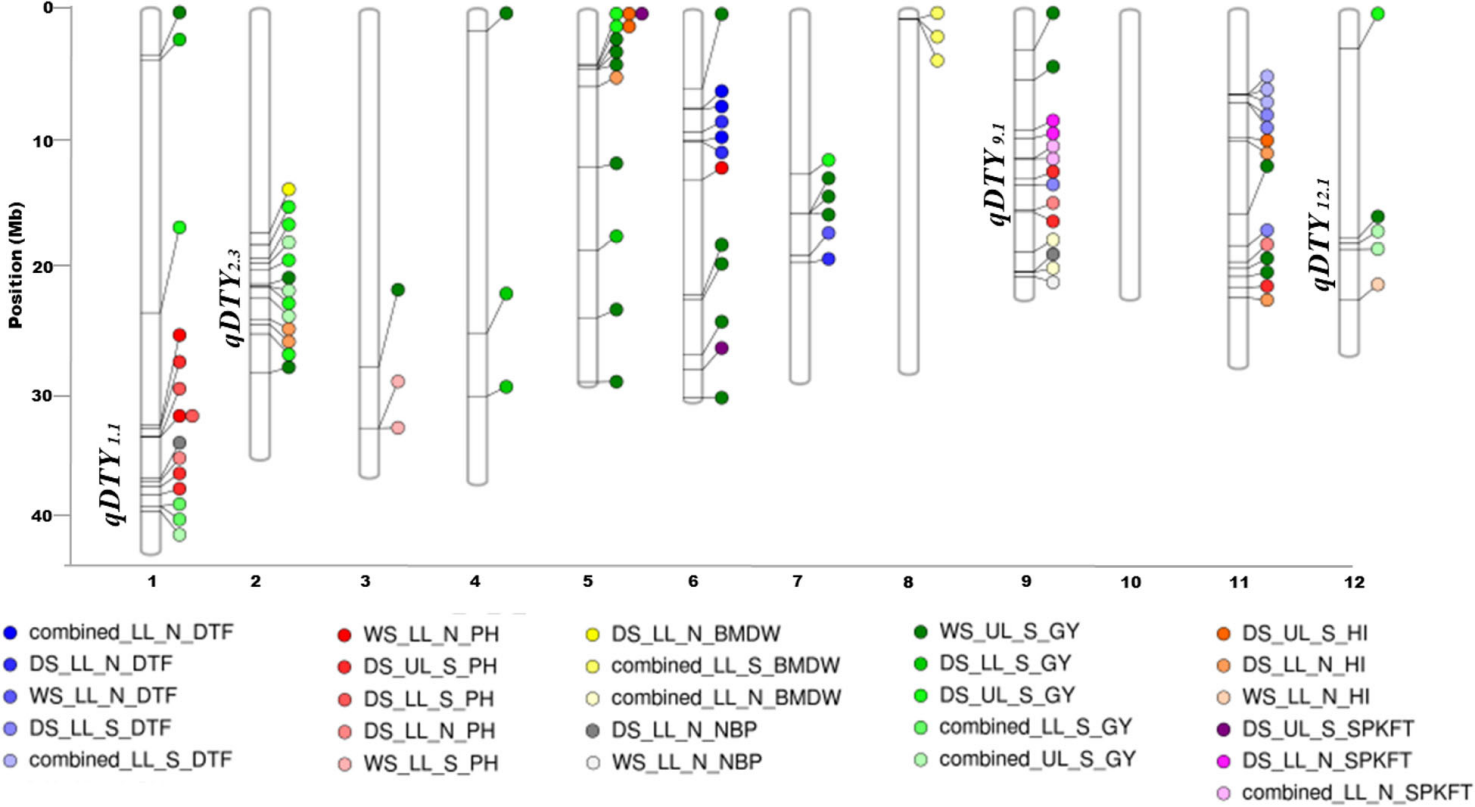
Genomic locations of 101 significant MTAs detected within a 200 kb window in reported genes and drought QTLs (*qDTYs)*. Colored circles show the position of each MTA for 7 traits: Days to 50% flowering-DTF, Plant height-PH, Biomass-BMDW, Number of effective panicles-NBP, Grain yield-GY, Harvest index-HI and Spikelet fertility-SPKFT from each of the six experiments (lowland non stress-LL_N, lowland stress-LL_S and upland stress-UL_S) in the two seasons (WS and DS) and combined season analysis. Exact genomic locations are shown in Table S4

Candidate gene analysis of the 219 MTAs with a 200 kb window centered from the MTA detected 101 of these MTAs within/ in close proximity to 38 genes from MSU database and 4 earlier reported major grain yield QTLs under drought (*qDTY*_*1.1*_, *qDTY*_*2.3*_, *qDTY*_*9.1*_ and *qDTY*_*12.1.*_). Summary of these results is presented in Table 3 wherein we also report 6 novel QTLs of about 0.5–1 Mb for DTF on chr 6, 11; for GY on chr 1, 2, 5 and for BMDW on chr 8 identified with strong peak markers across drought environments, associated mainly with putative retrotransposon proteins. An overview of the results for validation of MTAs for 7 traits out of 10 is presented in Fig. 8 which depicts the genomic locations of 101 MTAs validated from MSU database and literature for previously reported QTLs under drought. The significant MTAs/ QTLs were mainly located on chr 1, 2, 5, 6, 9, 11 and 12 and the percent phenotypic variance captured for these traits ranged from 5 to 88%.

**Table 3:** Summary of candidate gene analysis for seven traits in different seasons and environments validated for genes and qDTY regions

Trait	MTA/ QTL detected	QTL span (Mb)	SNP start (chrom_pos (bp))	SNP end (chrom_pos (bp))	Env	Season	Locus name (MSU 7.0) / Drought QTL (*qDTY*)
BMDW	–	–	S2_17808669	S2_17808669	LL_N	DS	LOC_Os02g30270
BMDW	*qBMDW*_8–1_	0.075	S8_00466752	S8_00541934	LL_S	WS,DS	–
BMDW	*qBMDW-NBP*_9–1_	2.021	S9_19316065	S9_20944019	LL_N	WS,DS	LOC_Os09g36290, LOC_Os09g36330
DTF	*qDTF*_6–1_	0.138	S6_07611279	S6_07749410	LL_N	WS,DS	–
DTF	*qDTF*_6–2_	0.832	S6_09539728	S6_10,371,528	LL_N	WS,DS	–
DTF	–	–	S7_19,598,023	S7_19,598,023	LL_N	WS	LOC_Os07g32800
DTF	–	–	S7_20,159,780	S7_20,159,780	LL_N	DS	LOC_Os07g32820
DTF	*–*	*–*	S9_13934171	S9_13934171	LL_S	DS	*qDTY*_*9.1*_
DTF	*qDTF*_11–1_	0.691	S11_06525213	S11_07215940	LL_S	WS,DS	–
DTF	–	–	S11_18836588	S11_18836588	LL_S	DS	LOC_Os11g31980
GY	*qGY*_1–1_	0.351	S1_03473291	S1_03824622	LL_S,UL_S	WS,DS	–
GY	–	–	S1_24304274	S1_24304274	UL_S	DS	LOC_Os01g43450
GY	*qGY*_1–2_	0.403	S1_39987434	S1_40390527	LL_S,UL_S	WS,DS	*qDTY*_*1.1*_
GY	*qGY*_2–1_	4.271	S2_18794394	S2_23065933	UL_S	WS,DS	LOC_Os02g33620, LOC_Os02g36190
GY	*qGY*_2–2_	3.18	S2_25979458	S2_29167141	UL_S	WS,DS	*qDTY*_*2.3*_
GY	–	–	S3_28646420	S3_28646420	UL_S	WS	LOC_Os03g49650
GY	–	–	S4_01441702	S4_01441702	UL_S	WS	LOC_Os04g25400
GY	–	–	S4_25920763	S4_25920763	LL_S	DS	LOC_Os04g37410
GY	–	–	S4_31088649	S4_31088649	LL_S	DS	LOC_Os04g50150
GY	*qGY*_5–1_	0.365	S5_04502747	S5_04505637	UL_S	WS	–
GY	–	–	S5_12494100	S5_12494100	UL_S	WS	LOC_Os05g20900
GY	–	–	S5_19224697	S5_19224697	LL_S	DS	LOC_Os05g32660
GY	*qGY*_*5–2*_	5.205	S5_24708194	S5_29913640	UL_S	WS	*OsRPK1*, *OsCCaMK*, *OsHAP3B*, *OsTPS1*, *OsSTN8*
GY	–	–	S6_06025083	S6_06025083	UL_S	WS	LOC_Os06g11540
GY	–	–	S6_22794237	S6_22794237	UL_S	WS	LOC_Os06g37850
GY	–	–	S6_23132086	S6_23132086	UL_S	WS	LOC_Os06g39690
GY	–	–	S6_27655258	S6_27655258	UL_S	WS	LOC_Os06g47030
GY	–	–	S6_31179920	S6_31179920	UL_S	WS	LOC_Os06g49910
GY	–	–	S7_13019061	S7_13019061	UL_S	DS	LOC_Os07g23450
GY	–	–	S7_16180595	S7_16180595	UL_S	WS	LOC_Os07g27900
GY	–	–	S7_16181810	S7_16181810	UL_S	WS	
GY	–	–	S7_16183053	S7_16183053	UL_S	WS	
GY	–	–	S9_03032058	S9_03032058	UL_S	WS	LOC_Os09g06650
GY	–	–	S9_05467194	S9_05467194	UL_S	WS	LOC_Os09g10300
GY	–	–	S11_16248710	S11_16248710	UL_S	WS	*qGP-11*, *qGI-11*, *yld11.1*, *gpl11.1*, *gl11.1*
GY	–	–	S11_20668615	S11_20668615	UL_S	WS	LOC_Os11g35310
GY	–	–	S11_21311326	S11_21311326	UL_S	WS	LOC_Os11g36200
GY	–	–	S12_02835650	S12_02835650	UL_S	DS	LOC_Os12g06020
GY	*qGY*_12–1_	0.941	S12_18165164	S12_19106346	UL_S	WS,DS	*qDTY*_*12.1*_
GY / HI	*qGY*_*5–1*_	0.365	S5_04140355	S5_04266313	UL_S	DS	–
HI	–	–	S2_24797737	S2_24797737	LL_N	DS	LOC_Os02g40920
HI	–	–	S2_25205930	S2_25205930	LL_N	DS	LOC_Os02g42020
HI	–	–	S5_05891992	S5_05891992	LL_N	DS	LOC_Os05g10700
HI	–	–	S11_10053723	S11_10053723	UL_S	DS	LOC_Os11g18366
HI	–	–	S11_10351950	S11_10351950	LL_N	DS	LOC_Os11g19230
HI	–	–	S11_23022593	S11_23022593	LL_N	DS	–
HI	–	–	S12_23250434	S12_23250434	LL_N	WS	LOC_Os12g37850
NBP	–	–	S1_37770897	S1_37770897	LL_N	DS	*qDTY*_*1.1*_
NBP	*qBMDW-NBP*_9–1_	2.021	S9_20908000	S9_21337553	LL_N	WS,DS	–
PH	*qPH*_1–1_	0.982	S1_33,418,648	S1_34,400,345	LL_N, LL_S, UL_S	WS,DS	LOC_Os01g53670, LOC_Os01g59760
PH	*qPH*_1–2_	1.085	S1_37,960,019	S1_39,044,781	LL_N, UL_S	WS,DS	*qDTY*_*1.1*_
PH	–	–	S3_33,600,040	S3_33,600,040	LL_S	WS	LOC_Os03g58220
PH	–	–	S3_33,600,989	S3_33,600,989	LL_S	WS	
PH	–	–	S6_13439145	S6_13439145	LL_N	WS	*OsPT9*, *OsPT1055*,*OsGLK1*, *nyc3*
PH	*qPH*_9–1_	2.731	S9_13,423,222	S9_16,154,337	LL_N,UL_S	WS,DS	*qDTY*_*9.1*_
PH	–	–	S11_20,143,839	S11_20,143,839	LL_N	DS	LOC_Os11g34364
PH	–	–	S11_22175365	S11_22175365	UL_S	DS	*qGP-11*,*qGl-11*, *yld11.1*, *gpl11.1*, *gw11.1*
SPKFT	–	–	S6_28876857	S6_28876857	UL_S	DS	LOC_Os06g49060
SPKFT	*qSPKFT*_9–1_	2.411	S9_09426722	S9_11838142	LL_N	WS,DS	–

#### Grain Yield under Drought in Different Environments as Selection Criterion for Promising Accessions to Develop High Yielding Drought Tolerant Varieties

Seven promising accessions viz. Aus 329, Aus 344, Chungur Bali, Dangar, Lalsaita, Para Nellu and Simul Khuri possessing better yield and yield related traits across different seasons under lowland and upland stress in combination of the favourable allele for yield and yield related traits were identified using two parameters- phenotypic performance evaluated using DSI and yield advantage over checks, and genotypic profile characterised by presence of favourable alleles contributing to high yield under drought.

DSI estimated for each genotype based on six important traits (DTF, PH, BMDW, GY, TGW and SPKFT) for each of the two stress conditions (LL_S and UL_S) in both WS and DS (6 traits * 2 stress conditions * 2 seasons) generated maximum of 24 DSI variables for each genotype used as selection criteria, to effectively determine the most tolerant and susceptible genotypes (Additional file 1: Table S2). Significant variability in genotypes exhibiting drought tolerance and susceptibility under different growing conditions was observed. Twelve accessions with DSIs close to zero for at least 20 DSI variables for each genotype were identified, highlighted in green in Additional file 1: Table S2. Subsequently, yield advantage of these 12 selections computed against the traditional varieties used as checks in the experiments narrowed selections down to seven accessions across environments and seasons. The grain yield improvement in these selected accessions ranged from 188 to 508 kg ha^− 1^ under lowland stress, to under 403 to 1645 kg ha^− 1^ lowland non-stress and 846 to 1800 kg ha^− 1^ under upland stress over the best performing checks in DS (Table 4).

**Table 4:** Selected promising accessions on basis of grain yield advantage across seasons and environments

S.No	Taxa Name	DTF		PH		GY					
		LL_N				LL_N		LL_S		UL_S	
		WS	DS	WS	DS	WS	DS	WS	DS	WS	DS
1	AUS 329::IRGC 29116–1	89	83	114	116	1171	4966	616	313	975	1224
2	AUS 344::IRGC 29131–1	87	84	105	125	1379	4943	575	335	994	1907
3	CHUNGUR BALI::IRGC 25855–1	86	77	124	115	1083	7107	592	569	987	1537
4	DANGAR::IRGC 76296–1	86	75	125	105	1785	5865	592	569	980	1059
5	LALSAITA::IRGC 43915–1	88	83	109	112	3465	4899	590	451	965	1778
6	PARA NELLU::IRGC 50009–1	87	75	144	127	3719	5925	582	633	975	953
7	SIMUL KHURI::IRGC 35154–1	91	86	120	118	1174	4844	574	340	994	1399
Check1	IRRI 154	–	93	–	93	–	5462	–	7	–	65
Check2	MTU1010	–	93	–	94	–	4323	–	2	–	60
Check3	Sabitri	–	90	–	101	–	3761	–	124	–	107
Trial Mean		90	89	114	109	1666	5214	573	158	942	336
LSD		1	2	3	3	140	280	82	32	100	62

**Fig. 9:**
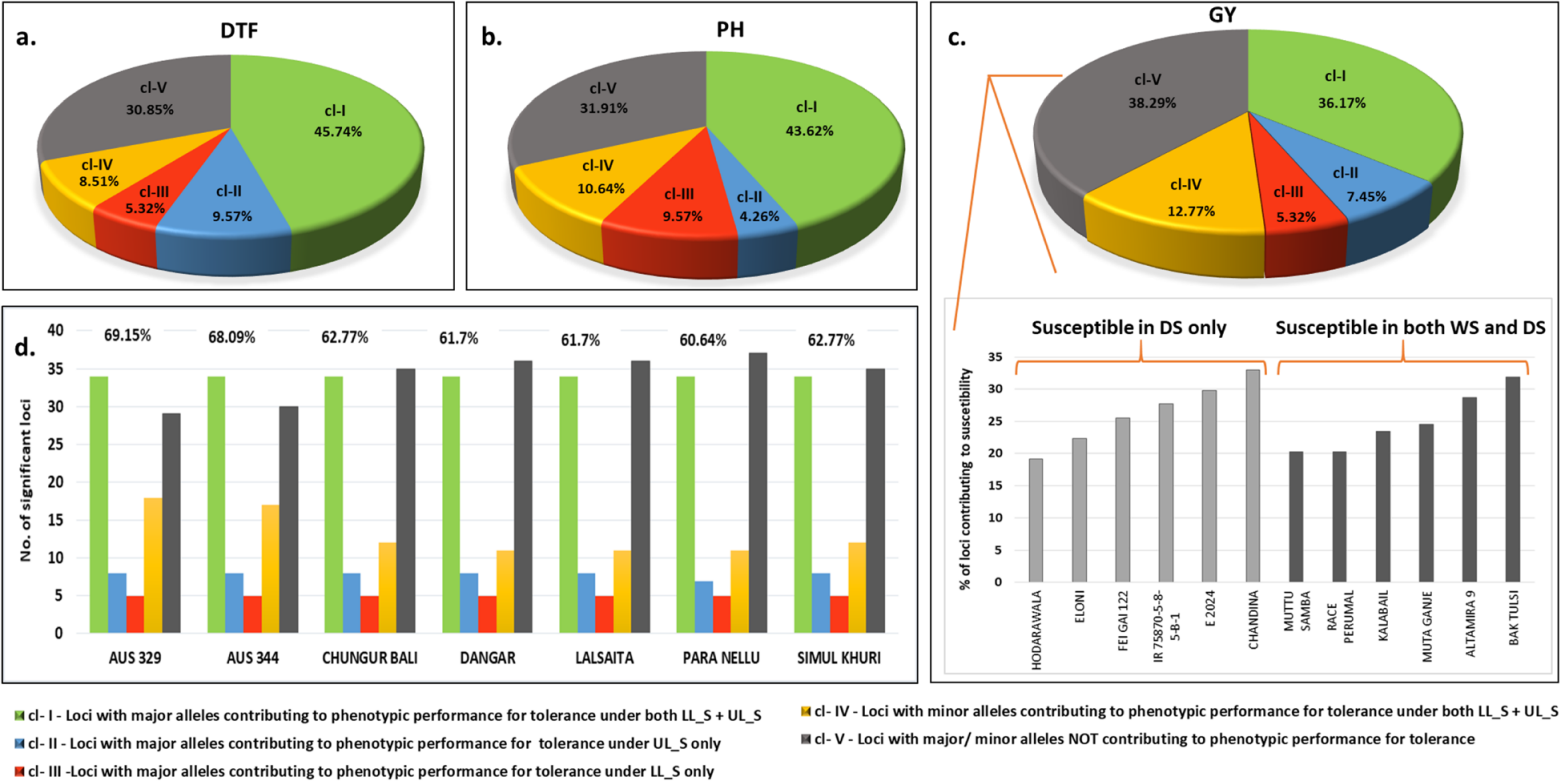
Allelic variation at the significant GWAS-identified loci analysed to detect percentage composition of allelic profile in the selected 7 promising accessions based on presence of major or minor allele contibuting the phenotypic performance for tolerance under reproductive stage drought in lowland- LL_S and upland- UL_S in the two seasons for **a**. DTF; **b**. PH; **c**. GY. The five classes identified include three with loci where major allele present in all selected accessions contributed to trait mean under – LL_S + UL_S(cl-l); only UL_S (cl-ll) and only LL_S (cl-lll) while the fourth class comprises loci with minor allele present in selected accessions contributing to trait mean under both LL_S + UL_S(cl-IV). Class V (cl-V) comprises all the remaining loci out of the analysed 94, with neither major nor minor allele contributing to phenotypic performance for tolerance under drought in the selected accessions. The ClassV loci were analysed for contribution to low yield in 12 susceptible lines across LL_S and UL_S in either or both WS and DS. The variability in allelic variation for GY in the 12 susceptible accessions is depicted in the bar graph, divided in two categories-susceptible in both stress environments in DS only and in both seasons-WS and DS. **d**. Validation of selected accessions based on percentage of allelic variation contributing to grain yield under LL_S and UL_S- loci with favorable allele in each the selected accessions is divided along the first four classes and loci with unfavourable allele in each acession is represented by class V. The cumulative favourable allelic percentage of each accession associated with higher yield under drought is depicted by the percentage value mentioned on top of the bar columns. Details for loci associated with GY are mentioned in Table S6

Validation of phenotype-based selection performed using set of 101 (on 94 unique loci with 4 having co-localization for multiple traits) significant MTAs (validated from database and earlier reported literature for grain yield QTLs) for DTF, PH and GY is presented in Fig.9a, b and c, respectively. Favorable alleles associated with DTF included major alleles in 45.74% loci contributing to both LL_S and UL_S (Class I abbreviated hereby as cl-I), major alleles in 9.57% loci contributing to only UL_S (cl-II) and major alleles in 5.32% loci contributing to only LL_S (cl-III). Interestingly, among favorable minor alleles, 8.5% of loci contributed to DTF under both LL_S and UL_S (cl-IV). Similarly, for PH, favorable alleles comprised 43.6%, 4.26%, 9.57% and 10.64% of 94 loci corresponding to classes I, II, III and IV, respectively while for GY, these figures were 36.17%, 7.45%, 5.32% and 12.7% for the four classes of loci respectively. The results imply that at least 44.67% loci (36.17% with major allele of the panel and 8.5% with minor allele of the panel) of the significant 94 loci can be useful in marker development, haplotype block construction for improving yield and yield related traits under both LL_S and UL_S; at least 4.26% of loci have favorable alleles to target UL_S for trait-based breeding and at least 5.3% of loci from panel have alleles for improving trait performance under LL_S.

Additionally, each selected accession was validated for presence of favorable alleles by computing their percent composition in the set of 94 loci, corresponding to the four classes established (Additional file 1: Table S6). The selected accessions belong predominantly to *aus* genetic sub-group (Aus329, Aus344, Chungur Bali, Dangar, Lalsaita and Simul Khuri) while Para Nellu belongs to *indica* genetic sub-group. The percentage of favorable alleles in their genotypic profile varied from 60 to 70% (Fig. 9d) which establishes usefulness of phenotype-based selection which is validated by a moderate to high percentage of genotypic profile of each accession possessing favorable alleles contributing to improved yield under RS drought.

Interestingly, about 38.29% of loci (cl-V) in the selected seven promising accessions did not contribute to high yield under drought (Fig. 9c). Analysis of these loci in 12 most susceptible accessions (detected having extremes of DSIs, highlighted in red in Additional file 1: Table S2) across lowland and upland stress environments, established variable allelic contribution to susceptibility under RS drought. The 12 accessions were divided into two categories, viz. Hodarawala, Eloni, Fei Gai 122, IR 75870–5–8-5-B-1, E-2024, Chandna (susceptible under different environments in DS only) and Muttu Samba, Race Perumal, Kalabail, Muta Ganje, Altamira-9 and Bak Tulsi (susceptible under different environments in both WS and DS). Validation of allelic contribution in these 12 susceptible accessions using 94 significant loci established a variable range of 19–33% loci with minor allele associated with low yield under drought, as depicted in the Fig. 9c.

## Discussion

### Phenotypic Characterization under Different Environments

In both the environments i.e. lowland and upland and across seasons, the yields of the accessions were lower under reproductive stage drought stress compared to the control non-stress indicating the severity of the drought stress imposed. Numerous studies point towards negative relation between yield potential and yield under drought and this has been used to establish response indices under different levels of stress severity (Raman et al. 2012; Kumar et al. 2014; Palanog et al. 2014). Positive correlation between moderate and severe stress response indices are informative of the genotypes with yield gain under all stress severities. In our study, different levels of stress severity were observed across seasons (Additional file 1: Table S1). Such differential levels of stress were useful in identifying the potential drought tolerant lines under variable growing environments. Verulkar et al. (2010) documented that yield reduction under reproductive stage drought is significant even at moderate stress severity and even lower under severe stress. The premise to use *indica*-*aus* diversity panel in the study was to identify the donors/accessions that can be directly used in breeding programs targeting grain yield improvement under drought for South-Asian and South-East Asian region.

### Effect of Trait Architecture for MTA Validation across Seasons and Environments

PV validation within the diversity panel in our study was affected by trait architecture and seasonal variation, where for example the range of variance captured by Farm-CPU is narrow for simple quantitative traits like flowering time (0.15–0.52) and plant height (0.34–0.63) as compared to that for a complex quantitative trait like GY with range of 0.02–0.55. In our study, the correlation among the two seasons (DS and WS) is of lower magnitude which warrants the variable PV for traits across seasons. Our results draw similar interpretations with recent studies that conclude the effectiveness of multi-locus methods, especially Farm-CPU over single-locus methods (like MLM) for association analysis of traits with either high or low heritability by adequately controlling false positives and negatives, indicated by sharp deviations observed for *p*-value distribution in qq plots (Xu et al. 2018; Kaler and Purcell 2019).

The significant and positive correlation among grain yield and other agronomic traits except for DTF and the colocation of MTAs associated with these traits indicates the contribution of grain yield related traits in contributing to yield improvement under drought stress. Most of the important economic traits such as grain yield, grain quality, biotic and abiotic stresses in different crop species are polygenic in nature. These complex quantitative traits being the focal for the breeding programs, genome wide analysis has proven to be advantageous in capturing the genetic variance of the diverse germplasm, subsequently contributing to improving crop productivity. Identification of marker-trait associations, QTLs, haplotypes, candidate genes and the functional characterization of the identified candidate genes underlying QTLs/genes will help plant breeders to design and develop drought tolerant rice varieties. In the present study, among the detected significant marker-trait associations, some were novel while the others were located near or co-located with the previously reported genes/QTLs.

Recently, GWAS studies conducted on 180 Vietnamese rice landraces identified a total of 17 QTLs associated with vegetative stage drought tolerance under greenhouse conditions (Hoang et al. 2019). Different significant MTAs in the two subpanels of the study, *indica* and *japonica* were detected using mixed model approach with structure control and kinship among the studied landraces. GWAS performed by Subedi et al. (2019) reported 37 highly significant MTAs for 20 traits including plant and root morphological traits, nutrient uptake, yield and its components in MAGIC population of 5 diverse parents for increased adaptability in dry direct seeded rice (DDSR) system.

### MAS Optimization Based on Significant Genomic Regions Identified

The QTLs; *qGY*_*1–2*_ and *qPH*_*1–2*_ and the MTAs (S1_37770897 for NBP) mapped on chromosome 1; *qGY*_*2–2*_ on chromosome 2; *qPH*_*9–1*_ and *qSPKFT*_*9–1*_ on chromosome 9 and qGY_12–1_ mapped on chromosome 12 in both the years and environments were located near the earlier reported major grain yield QTLs namely *qDTY*_*1.1*_, *qDTY*_*2.3*_, *qDTY*_*9.1*_ and *qDTY*_*12.1*_ respectively (Table 3). These findings indicate the consistency of the effects of drought grain yield QTLs across diverse germplasm. It is important to take note here that the *qDTY*_*1.1*_ was reported to have significant effect on the grain yield under control non-stress and reproductive stage drought stress in different genetic backgrounds such as Swarna, IR64, MTU1010 under lowland and upland environments (Vikram et al. 2011; Sandhu et al. 2014; Sandhu et al. 2015). The *qGY*_*2–1*_ and *qGY*_*2–2*_ reported in the present study were found to be present in the upstream and downstream region of earlier reported *qDTY*_*2.3*_, respectively (Sandhu et al. 2014; Palanog et al. 2014). Interestingly, the *qGY*_*5–2*_ reported in the present study was located near the earlier reported genes *OsRPK1* gene (Chen et al. 2013 for root development), *OsCCaMK* (Bao et al. 2014 for microbial symbiosis), *OsHAP3B, OsTPS1* (Miyoshi et al. 2003 for chloroplast biogenesis), *OsSTN8* (Nath et al. 2013) for protein phosphorylation of photosystem II) and MTAs for nutrient uptake (Sandhu et al. 2019). The colocation of identified QTLs in the present study with the earlier reported genes for root development, photosynthetic traits, and the stress-responsive genes further indicate the complex nature of grain yield traits in addition to the contribution of these traits/genomic regions in enhancing yield under drought. After validation, the identified significant marker-trait associations and the selected promising accessions possessing the QTLs/MTAs could be used further in GAB program. The seven selected accessions from this panel may provide novel donors in developing drought tolerant rice varieties for variable growing environment.

### Research Prospective for Breeders

Diversity panels are a valuable source for exploiting genetic variation to potentially raise genetic gain in an integrative pre-breeding approach. Association studies help in detecting genetic variants associated with agronomically important traits and identifying underlying candidate genes and establishing haplotypes to accelerate development of climate-smart cultivars. In our present study, we detected 94 significant loci associated with 38 genes and 4 major grain yield QTLs. Analysing phenotypic performances of various haplotype combinations of these in post-GWAS study and functional characterization of candidate gene expressions can help ascertain superior haplotype combinations for improved grain yield under RS drought in different ecosystems. Exploiting such superior performance haplotypes in different genetic backgrounds; detecting presence of such multiple haplotypes in accessions can aid genomic selection for tailoring development of high-yielding climate-smart varieties. The seven accessions selected based on grain yield and analysed for allelic variation, can serve as potential donors for improving yield under reproductive-stage drought in different ecosystems, as favourable alleles contributing to yield under drought comprised 60–70% genotypic profile in significant loci. Moreover, these selected accessions belong to *aus* and *indica* genetic backgrounds, hence can be exploited to identify consistent, superior haplotypes for yield and yield related traits with potential to strengthen rice production by deployment of tailored climate-smart varieties.

## Conclusions

The diverse *indica-aus* panel possessing wide range of phenotypic variability combined with the already available genomic information was exploited to identify the MTAs/QTLs associated with grain yield improvement under reproductive stage drought. A total of 219 significant MTAs were detected in the present study. Candidate gene analysis within 200 kb window centred from GWAS identified SNP peaks detected 101 of these MTAs within/ in close proximity to 38 reported genes, 4 earlier reported major grain yield QTLs and 6 novel QTLs for 7 traits. Two QTLs each for plant height and grain yield showed consistent effect across seasons and environments under both control non-stress and stress conditions. The significant positive correlation of the grain yield with grain yield related traits was further confirmed with the colocation of QTLs/MTAs associated with these traits. The introgression of the identified QTLs into elite genetic background, functional characterization of candidate genes identified in or near QTLs regions would be the next step in improving grain yield of rice under reproductive stage drought stress conditions. The identified promising accessions may serve as novel donors in drought breeding program targeting grain yield improvement.

## Methods

### Plant Material and Genotypic Data

The study used data evaluated for a diverse *indica-aus* rice panel of 280 accessions, of which 245 represent the four major genetic subgroups belonging to *indica* genetic background and 35 to *aus* genetic background (Additional file 1: Table S7). They were selected from the 3000 accessions recently re-sequenced within the framework of the Rice Genome Project (Li et al. 2014), for their potential to breeding programs targeting rainfed lowland and upland drought environments in South and South-East Asia. In the selected panel, 215 accessions are landraces originating mainly from Asia and 65 accessions are improved lines. Seeds of the accessions were obtained from the IRRI gene bank.

The genotypic data for the 280 accessions were obtained from the International Rice Informatics Consortium (IRIC) database for the 3000 rice genomes project (http://oryzasnp.org/iric-portal). The raw genotypic data extracted from the database contained 962 k SNPs. The filtering for missing data (≤ 20%), minor allele frequency (MAF) ≥ 2% and rate of heterozygosity (Ho) ≤ 5% led to a working set of 215,250 SNPs, referred to as 215 k set. This panel and the associated genotypic data were previously described in Bhandari et al. (2019).

### Phenotyping of Population

#### Experimental Design and Crop Management

Six experiments (Additional file 1: Table S1) were conducted in the 2014 wet season (WS) and 2015 dry season (DS) at IRRI (14.18°N, 121.25°E), Philippines. In each season, the experiment was conducted under control conditions or non-stress experiment (LL_N) in lowland (under flooded, puddled, transplanted and anaerobic conditions) while the reproductive-stage drought-stress experiments were conducted in lowland and upland (under direct-sown, non-puddled, non-flooded and aerobic conditions in leveled fields) environments, referred as LL_S and UL_S, respectively. The LL_N experiments were established in augmented randomized complete block design in single-row plots with 5 m row length. The LL_S and UL_S experiments were established in a α-lattice design with two replications in single or two-row plots with 5 m row length in lowland and 2–3 m row length in upland. The crop management practices were as described in Kumar et al. (2014).

#### Drought Application Procedure

RS-drought phenotyping was as described in Kumar et al. (2014). Briefly, in the LL_S experiments, the field was drained 30 days after transplantation and irrigation was withheld to impose the RS-drought stress. Stress was continued until severe leaf rolling was observed in at least 75% of the accessions and water table depth remained below 100 cm for more than 2 weeks. Fields were thereafter re-irrigated (flash-flooding -WS and sprinklers - DS) and the water was drained after 24 h to impose a subsequent cycle of drought stress. This cyclic pattern was implemented until harvest. In the UL_S experiments, where the crop was established by direct-seeding, RS-drought stress was initiated 45 days after sowing, by withholding sprinkler irrigation until the soil water tension fell below − 50 kPa at 30 cm depth. Thereafter, sprinkler-irrigation and subsequent drainage after 24 h for the imposition of drought stress were done in a cyclic pattern till harvest.

#### Traits Measured

For each experiment, days to 50% flowering (DTF, in days), plant height (PH, in cm, the average for 3 measurements per plot), panicle length (PL, in cm, the average for 3 measurements per plot), flag leaf area (FlgLA, in cm^2^, the average for 3 measurements per plot), dry biomass at maturity (BMDW, in kg ha^− 1^), number of effective panicles (NBP), grain yield (GY, in kg ha^− 1^), 1000-grain weight (TGW, in g) and spikelet fertility (SPKFT, in percentage) were measured in individual plots and harvest index (HI) was calculated as GY/BMDW. Details of measurement procedures of each trait are given in Additional file 1: Table S8.

#### Analysis of Phenotypic Data for each Trait

For each trait from each of the six experiments, best linear unbiased predictors (BLUP) were estimated using the restriction maximum likelihood method (REML) in the PROC MIXED procedure of SAS v9.0 (Statistical Analysis Systems 2002). Within a season, the performance of a genotype was modeled as *Y*_*ij*_ *= μ + ß*_*i*_ *+ c*_*j*_ *+ α*_*i*_ *+ ε*_*ij*_ for augmented randomized complete block design where Y_ij_ is the phenotype of the i^th^ genotype in j^th^ block, μ the overall mean, ß_i_ the block effect which was considered as random, c_j_ the checks effect in j^th^ block which was considered as fixed, α_i_ the random effect of the i^th^ genotype and ε_ij_ is the residual considered as a random effect. We constructed two variables- “checks” and “genotypes” variables in both WS and DS. Checks refer to the control genotypes included additionally in the experiment to compare the performance of genotypes being tested and were used to recover the block effects. For α-lattice design, genotype performance was modeled as *Y*_*ijk*_ *= μ + α*_*i*_ *+ r*_*j*_ *+ b*_*kj*_ *+ ε*_*ijk*_ where Y_ijk_ is the phenotype of the i^th^ genotype in k^th^ block of j^th^ replicate, μ the overall mean, α_i_ is the genotype effect considered as random, r_j_ is the replicate effect considered as fixed, b_kj_ is the random effect of the k^th^ block within j^th^ replicate and ε_ijk_ is the residual considered as a random effect.

The variance components were estimated using the REML method to extract Y_adj_ (*μ* + Y_ij(k)_) values for each genotype which were used in GWAS for analysis at both individual experiment level and combined analysis for each environment- lowland non-stress, lowland stress, and upland stress, to detect genomic regions associated with traits of interest. For each of the studied trait, the broad-sense heritability was estimated using the formula

$$ {\mathrm{H}}^2={\upsigma^2}_{\mathrm{g}}/{\upsigma^2}_{\mathrm{p}} $$

where σ^2^_g_ is the genotypic variance obtained from the experimental data (assuming only additive genetic variance among accessions) and the phenotypic variance is σ^2^_p_ = σ^2^_g_ + σ^2^_e_/r, where σ^2^_e_ is the residual variance obtained from the ANOVA and r is the number of replication.

The corrplot package in R (R. v.1.2.5001) (Wei and Simko 2017) was used to estimate the correlation among the measured traits.

The drought susceptibility index (DSI) was calculated for DTF, PH, BMDW, GY, TGW and SPKFT. Drought intensity (DI) was calculated according to (Lazar et al. 1995) as follows-

$$ \mathrm{DI}=1-{\mathrm{Y}}_{\mathrm{D}}/{\mathrm{Y}}_{\mathrm{N}} $$

Where Y_D_ is the average all genotypes for a given trait under drought stress, while, the Y_N_ is the average of all genotypes for the same trait under normal condition. The drought susceptibility index (DSI) was estimated for each genotype and calculated according to (Lazar et al. 1995) as follows-

$$ \mathrm{DSI}=\frac{1\hbox{-} {\mathrm{X}}_{\mathrm{D}}/{\mathrm{X}}_{\mathrm{N}}}{\mathrm{D}\mathrm{I}} $$

Where X_D_ is the mean performance of each genotype for a given trait under drought stress, while, the X_N_ is the mean performance of each genotype for the same trait under normal condition.

### Methods for Characterizing the Population

#### Experimental Evaluation

Multi-dimensional analysis of the phenotypic data by FDA was performed on phenotypic data (280 accessions × 10 trait variables × 6 experiments) to estimate the pairwise Fisher distance between the experiments using the XLSTAT package [Internet] 2012. (http://www.xlstat.com/en/products-solutions/pro.html) XLSTAT (2012). Using mean grain yield as criterion, each experiment was re-classified based on the grain yield reduction compared to the control-lowland-non-stress experiment (Kumar et al. 2009) (Additional file 1: Table S1).

#### Genetic Structure

The genetic diversity among the 280 accessions was studied with the working set of 215 k markers using the Neighbor-joining (NJ) clustering method in TASSEL 5 (Bradbury et al. 2007) and visualization using FigTree v1.4.3 (Rambaut and Drummond 2016). The population structure was assessed using ADMIXTURE v.1.3.0 (Alexander et al. 2009) and results visualized using R/pophelper (Francis 2017) package for 280 accessions and 215 k SNPs. Series of models for K value ranging from 2 to 8 were run with 5 fold cross-validation to prime the main algorithm- QuasiNewton for convergence acceleration. Accuracy and precision were ensured by performing 20 runs for each value of K and the optimal number of clusters was determined by the K value with the least cross-validation (CV) error. Principal components (PC) explaining genetic variation were estimated using R/GAPIT 3.0 package (Lipka et al. 2012). The estimated population structure covariates (Q) and kinship matrix (K) were used to improve the statistical power of the GWAS models used.

#### Pairwise Linkage Disequilibrium (LD)

LD between SNP loci at the individual chromosomal level was calculated and plotted by computing r^2^ estimators between all pairs of SNP markers using the PopLDdecay (Zhang et al. 2019).

### Methods for Identifying Associations at the Population Level

In our study, we implemented GWAS with MLM, SUPER and Farm-CPU methods using R/GAPIT 3.0 package and visualization of circular manhattan and qq plots using rMVP package (0.99.17) (https://github.com/xiaolei-lab/rMVP)(R/MVP package 2019). The false positives in GWAS study were corrected using “Bonferroni Correction” factor. Using the Bonferroni multiple test correction (0.05/215,250; at 5% level of significance), the calculated threshold value was 2.32 × 10^− 7^. Only the MTAs that exceed the threshold value and which were consistent across multi-locus methods- SUPER and Farm-CPU methods have been reported in this study. To detect seasonal variations, we explored two *p*-value thresholds (1e-6 and 1e-4).

The percent phenotypic variance (PV) explained by all significant SNPs detected in each environment and season was output from all models used in the study. PV explained by each significant SNP was calculated as the squared correlation between the phenotype and genotype of the SNP.

### Candidate Genes Discovery

The candidate genes were searched within the 200-kb region around (100 kb upstream and 100 kb downstream) the detected significant SNP. The literature searches were also performed using QTARO and MSU databases (http://qtaro.abr.affrc.go.jp (QTARO database 2019) and http://rice.plantbiology.msu.edu (MSU database 2019)) to identify the earlier reported QTLs present in the LD region.

### Selection of Accessions as Potential Donors in Breeding Programs

Promising accessions were selected from the population based on yield advantage over non-stress condition in WS for both lowland and upland stress environments and over checks in each environment in DS. The premise was to identify a set of accessions that can be incorporated in breeding programs for drought tolerance under both lowland and upland environments with the advantage of early flowering and short plant type under RS drought.

These selected accessions were analyzed for allelic effect using 101 (on 94 unique loci with 4 having colocalisation for multiple traits) significant MTAs validated from database and earlier reported literature for grain yield QTLs. Allelic variation was studied for effect of allelic contribution to trait mean for DTF, PH and GY under LL_S and UL_S in both seasons. Five classes of loci were established – three based on presence of major allele in all seven accessions contributing to phenotypic performance for tolerance under LL_S + UL_S (class I abbreviated as cl-I); under UL_S only (class II abbreviated as cl-II) and under LL_S only (class III abbreviated as cl-III) while the fourth class (cl-IV) contained loci with minor allele associated to phenotypic performance for tolerance under both LL_S + UL_S. The fifth class (cl-V) consisted of loci with neither the major nor minor allele associated to phenotypic performance for tolerance under RS drought in the selected accessions. Further, validation of phenotypic-based selection of each accession was done by computing the percentage composition of favorable alleles in the set of 94 loci.

## Supplementary information

**Additional file 1. Table S1.** Field experiments conducted at IRRI, Philippines between the 2014 wet season and 2015 dry season. **Table S2.** Drought susceptibility index (DSI) calculated for each genotype for the four stress experiments for selected traits (with green for tolerance and red for susceptibility). **Table S3.** Characterization of the marker set of 215,250 SNPs. **Table S4.** Significant MTAs detected for ten traits in the population and the gene validation results. **Table S5.** Yadj. values used for GWAS of the ten traits measured for the 280 diverse set in three environments and two seasons. **Table S6.** Allelic variation at the GWAS-identified significant loci in the selected 7 accessions for grain yield under lowland and upland drought. **Table S7.** Details of the 280 diversity panel accessions. **Table S8.** Description of phenotypic data recording. **Figure S1.** Projection of 280 lines of the indica-aus diversity panel on the first plane of factorial discriminant analysis using phenotypic data for ten traits. **Figure S2.** Plots of Pearson’s r-values showing correlation between each of the six experiments (LL_N_WS – lowland non-stress 2014WS, LL_S_DS – lowland non-stress 2015DS, LL_S_WS – lowland stress 2014WS, LL_S_DS – lowland stress 2015DS, UL_S_WS – upland stress 2014WS and UL_S_DS – upland stress 2015DS) at trait level for all the ten phenotypic variables. **Figure S3.** Heat map showing the uneven marker distribution along each of the 12 chromosomes using the 215,250 SNP working set. **Figure S4.** Pattern of rapid decay in linkage disequilibrium decay in the population of 280 accessions genotyped with 215,250 SNPs. **Figure S5.** Circular manhattan plots and qq-plots for each of the 6 experiments and comparison of season-wise analysis to combined analysis for each of the 3 growing environments for a. PL, b. FlgLA, c. BMDW, d. NBP, e. HI, f. TGW and g. SPKFT.

## Data Availability

The complete phenotypic data (Y_adj._ values used in the analysis) are provided in Additional file 1: Table S5. The genotypic data for the 280 accessions used in this study (details provided in Additional file 1: Table S7) can be downloaded from the International Rice Informatics Consortium (IRIC) database for the 3,000 rice genomes project (http://oryzasnp.org/iric-portal).

## Abbreviations

BMDW: Dry biomass at maturity
DTF: Days to 50% flowering
Farm-CPU: Fixed and random model for circulating probability unification
FlgLA: Flag leaf area
GWAS: Genome-wide association study
GY: Grain yield
HI: Harvest index
Ho: Observed heterozygosity
TGW: 1000-grain weight
MAF: Minor allele frequency
MLM: Mixed linear model
MTA: Marker-trait association
NBP: Number of effective panicles
PH: Plant height
PL: Panicle length
QTL: Quantitative trait locus
SNP: Single nucleotide polymorphism
SPKFT: Spikelet fertility
SUPER: Settlement of MLM under progressively exclusive relationship
