# Supplementary material for: Genome-Wide Association Study for Yield and Yield Related Traits under Reproductive Stage Drought in a Diverse indica-aus Rice Panel
Source: Rice (N Y). 2020 Aug 6;13:53. doi: 10.1186/s12284-020-00406-3 (PMC7410978; doi:10.1186/s12284-020-00406-3)
Supplement: Supplementary file 1 — Additional file 1. Table S1. Field experiments conducted at IRRI, Philippines between the 2014 wet season and 2015 dry season. Table S2. Drought susceptibility index (DSI) calculated for each genotype for the four stress experiments for selected traits (with green for tolerance and red for susceptibility). Table S3. Characterization of the marker set of 215,250 SNPs. Table S4. Significant MTAs detected for ten traits in the population and the gene validation results. Table S5. Yadj. values used for GWAS of the ten traits measured for the 280 diverse set in three environments and two seasons. Table S6. Allelic variation at the GWAS-identified significant loci in the selected 7 accessions for grain yield under lowland and upland drought. Table S7. Details of the 280 diversity panel accessions. Table S8. Description of phenotypic data recording. Figure S1. Projection of 280 lines of the indica-aus diversity panel on the first plane of factorial discriminant analysis using phenotypic data for ten traits. Figure S2. Plots of Pearson’s r-values showing correlation between each of the six experiments (LL_N_WS – lowland non-stress 2014WS, LL_S_DS – lowland non-stress 2015DS, LL_S_WS – lowland stress 2014WS, LL_S_DS – lowland stress 2015DS, UL_S_WS – upland stress 2014WS and UL_S_DS – upland stress 2015DS) at trait level for all the ten phenotypic variables. Figure S3. Heat map showing the uneven marker distribution along each of the 12 chromosomes using the 215,250 SNP working set. Figure S4. Pattern of rapid decay in linkage disequilibrium decay in the population of 280 accessions genotyped with 215,250 SNPs. Figure S5. Circular manhattan plots and qq-plots for each of the 6 experiments and comparison of season-wise analysis to combined analysis for each of the 3 growing environments for a. PL, b. FlgLA, c. BMDW, d. NBP, e. HI, f. TGW and g. SPKFT. [file 12284_2020_406_MOESM1_ESM.docx]

| **Table S1** Field experiments conducted at IRRI, Philippines between the 2014 wet season and 2015 dry season | | | | | | | | | |
| --- | --- | --- | --- | --- | --- | --- | --- | --- | --- |
| **Set** | **Year** | **Season** | **Condition** | **Water management** | **Sowing date** | **Stress Initiation date** | **Trial Yield Mean (kg/ha)** | **Yield reduction** | **Stress level*** |
| 1 | 2014 | Wet | Transplanted | Irrigated | 27/06/14 | - | 1655 | - | - |
| 2 | 2014 | Wet | Transplanted | Reproductive-Stress | 07/07/14 | 01/09/14 | 595 | 64 | Severe |
| 3 | 2014 | Wet | Direct-seeded | Reproductive-Stress | 07/07/14 | 01/09/14 | 707 | 57 | Moderate |
| 4 | 2015 | Dry | Transplanted | Irrigated | 16/12/14 | - | 5214 | - | - |
| 5 | 2015 | Dry | Transplanted | Reproductive-Stress | 06/01/15 | 27/02/15 | 128 | 98 | Very Severe |
| 6 | 2015 | Dry | Direct-seeded | Reproductive-Stress | 31/01/15 | 27/02/15 | 322 | 94 | Very Severe |
| * Control—continuous irrigation with no yield reduction due to water stress; moderate—yield reduction of 40–60%; severe—yield reduction of 60–85%; very severe—yield reduction of more than 85% as compared with control. | | | | | | | | | |

| **Table S2** Drought susceptibility index (DSI) calculated for each genotype for the four stress experiments for selected traits (with green for tolerance and red for susceptibility)   | **Study designation** | **Condition** | **DTF_DSI** | **PH_DSI** | **BMDW_DSI** | **GY_DSI** | **TGW_DSI** | **SPKFT_DSI** | | --- | --- | --- | --- | --- | --- | --- | --- | | RP002 | LL_S_WS | 1.5437 | 0.6339 | 1.0282 | 1.0121 | 1.0973 | 0.7536 | | RP003 | LL_S_WS | 1.5437 | 3.6840 | 1.2303 | 1.3978 | 2.3165 | 2.1996 | | RP004 | LL_S_WS | 1.5437 | 0.9411 | 0.9174 | 0.9165 | 0.0965 | 0.6703 | | RP005 | LL_S_WS | 0.1532 | 0.1997 | 0.9506 | 1.0080 | 0.7413 | 0.7597 | | RP006 | LL_S_WS | 0.1579 | -0.3276 | 0.8339 | 0.8487 | 1.2473 | 0.8145 | | RP007 | LL_S_WS | 1.5437 | 3.6840 | 1.2303 | 1.3978 | 2.3165 | 2.1996 | | RP008 | LL_S_WS | 1.5437 | 0.6024 | 1.0558 | 0.4359 | 0.3432 | 0.6152 | | RP009 | LL_S_WS | 0.1812 | -0.1165 | 1.0009 | 0.9477 | -0.6515 | 0.6804 | | RP010 | LL_S_WS | 0.1549 | 0.9339 | 1.0046 | 0.8912 | NA | 0.6144 | | RP011 | LL_S_WS | 0.1785 | 0.1586 | 0.8636 | 1.0693 | 0.2896 | 0.6208 | | RP012 | LL_S_WS | 1.5437 | 0.2229 | 0.8628 | 0.3955 | NA | 0.7105 | | RP013 | LL_S_WS | 0.1652 | 0.3819 | 0.9255 | 1.0614 | 0.8803 | 0.7139 | | RP014 | LL_S_WS | 1.5437 | 0.1404 | 0.8477 | 1.0153 | 1.1583 | 0.7216 | | RP015 | LL_S_WS | 0.0870 | 0.3909 | 1.0367 | 1.0659 | 0.1579 | 0.7815 | | RP018 | LL_S_WS | 1.5437 | 1.1393 | 1.0212 | 0.7283 | 0.4170 | 0.4816 | | RP019 | LL_S_WS | 1.5437 | -0.1798 | 0.8999 | 0.8345 | -0.3217 | 0.5936 | | RP020 | LL_S_WS | 1.5437 | 0.2283 | 0.9722 | 1.0417 | 1.3364 | 0.8179 | | RP021 | LL_S_WS | 1.5437 | 0.0935 | 0.9047 | 0.8934 | 0.5486 | 0.8167 | | RP022 | LL_S_WS | 1.5437 | 3.6840 | 1.2303 | 1.3978 | 2.3165 | 2.1996 | | RP023 | LL_S_WS | 0.1089 | 0.2723 | 0.9766 | 1.0077 | 1.1100 | 0.6879 | | RP024 | LL_S_WS | 0.0642 | 0.1970 | 0.9502 | 1.0632 | 0.8057 | 0.8178 | | RP025 | LL_S_WS | 1.5437 | 0.3653 | 0.9632 | 0.9742 | -0.1158 | 0.6686 | | RP026 | LL_S_WS | 1.5437 | 0.4085 | 0.9230 | 0.8305 | 0.3309 | 0.8506 | | RP028 | LL_S_WS | 0.1189 | 1.9444 | 1.0196 | 1.1586 | 1.2590 | 0.8102 | | RP029 | LL_S_WS | 1.5437 | 0.5052 | 0.8954 | 1.1594 | 1.4701 | 0.7789 | | RP030 | LL_S_WS | 0.1700 | 0.6980 | 0.9653 | 1.0064 | 0.2206 | 0.6754 | | **Study designation** | **Condition** | **DTF_DSI** | **PH_DSI** | **BMDW_DSI** | **GY_DSI** | **TGW_DSI** | **SPKFT_DSI** | | RP031 | LL_S_WS | 0.1723 | 0.4717 | 0.9107 | 0.9495 | -5.2121 | 0.8583 | | RP032 | LL_S_WS | 0.0913 | 0.1359 | 1.0381 | 0.6637 | 0.8424 | 0.8884 | | RP033 | LL_S_WS | 0.2367 | -0.3268 | 0.8478 | 1.0057 | -0.3861 | 0.6912 | | RP034 | LL_S_WS | 0.1345 | 0.0758 | 0.9182 | 0.7512 | 0.5539 | 0.6101 | | RP036 | LL_S_WS | 0.1234 | 0.0157 | 0.9150 | 1.0959 | 0.2896 | 0.8809 | | RP037 | LL_S_WS | 0.2466 | 0.4485 | 0.9285 | 0.9143 | 0.3706 | 0.6185 | | RP038 | LL_S_WS | 0.0903 | 0.1768 | 0.9669 | 0.9490 | NA | 0.6936 | | RP039 | LL_S_WS | 0.2257 | -0.1657 | 0.9338 | 0.6631 | 0.3378 | 0.6081 | | RP040 | LL_S_WS | 0.1440 | -0.1520 | 0.9527 | 0.8144 | 0.8019 | 0.8414 | | RP041 | LL_S_WS | 0.1581 | 0.0529 | 0.8662 | 0.5224 | NA | 0.6740 | | RP042 | LL_S_WS | 0.1833 | -0.0357 | 0.7997 | 1.0075 | 1.0656 | 0.8288 | | RP043 | LL_S_WS | 1.5437 | 3.6840 | 1.2303 | 1.3978 | 2.3165 | 2.1996 | | RP044 | LL_S_WS | 0.1159 | 0.4325 | 0.9067 | 0.8541 | 0.0526 | 0.6367 | | RP045 | LL_S_WS | 0.1026 | 0.2698 | 1.0089 | 0.9756 | 0.0000 | 0.6696 | | RP046 | LL_S_WS | 0.0821 | -0.0305 | 0.8925 | 0.9187 | 0.8151 | 0.6316 | | RP047 | LL_S_WS | 0.2367 | 0.3341 | 0.9712 | 0.7834 | 0.2044 | 0.6788 | | RP048 | LL_S_WS | 0.1945 | 0.2670 | 0.9539 | 0.7570 | 0.3022 | 0.5790 | | RP049 | LL_S_WS | 1.5437 | 0.4716 | 0.8208 | 0.7612 | 1.4961 | 0.8407 | | RP050 | LL_S_WS | 1.5437 | 0.4616 | 0.9575 | 1.1282 | 0.6132 | 0.8030 | | RP051 | LL_S_WS | 0.3044 | 0.3295 | 0.9137 | 0.7402 | 0.1158 | 0.6622 | | RP052 | LL_S_WS | 1.5437 | -0.2321 | 0.9750 | 0.8068 | 0.1930 | 0.5400 | | RP053 | LL_S_WS | 0.1593 | 0.2402 | 0.8837 | 0.8959 | 0.7446 | 0.7338 | | RP054 | LL_S_WS | 1.5437 | 0.3717 | 0.9086 | 0.6375 | 0.3309 | 0.5943 | | RP055 | LL_S_WS | 1.5437 | -0.1057 | 0.8038 | 1.0691 | 0.0000 | 0.7950 | | RP056 | LL_S_WS | 1.5437 | 0.0045 | 1.0427 | 0.1791 | 1.6037 | 0.8189 | | RP057 | LL_S_WS | 1.5437 | 3.6840 | 1.2303 | 1.3978 | NA | 2.1996 | | RP058 | LL_S_WS | 1.5437 | -0.2195 | 0.9675 | 0.8829 | 2.1841 | 0.8226 | | RP059 | LL_S_WS | 0.0740 | -0.1350 | 1.0291 | -0.0312 | -2.4323 | 0.5613 | | **Study designation** | **Condition** | **DTF_DSI** | **PH_DSI** | **BMDW_DSI** | **GY_DSI** | **TGW_DSI** | **SPKFT_DSI** | | RP060 | LL_S_WS | 0.1371 | 0.3119 | 0.8670 | 0.9076 | -0.4267 | 0.6719 | | RP061 | LL_S_WS | 0.1553 | 0.0100 | 0.9553 | 1.0825 | 0.6237 | 0.6957 | | RP062 | LL_S_WS | 1.5437 | -1.1182 | 0.9953 | 1.0107 | 0.7371 | 0.8224 | | RP063 | LL_S_WS | 0.2453 | -1.0566 | 0.9769 | 0.7436 | 1.2919 | 0.7414 | | RP065 | LL_S_WS | 0.2067 | -0.0198 | 0.9101 | 0.9167 | NA | 0.7821 | | RP066 | LL_S_WS | 1.5437 | 0.0795 | 0.9166 | 1.0245 | 0.7925 | 0.8060 | | RP067 | LL_S_WS | 1.5437 | 3.6840 | 1.2303 | 1.3978 | 2.3165 | 2.1996 | | RP068 | LL_S_WS | 0.1444 | 0.4457 | 0.9434 | 0.8794 | 0.5791 | 0.6627 | | RP070 | LL_S_WS | 1.5437 | 0.4138 | 0.9658 | 0.8921 | 1.6459 | 0.6554 | | RP071 | LL_S_WS | 1.5437 | 0.4063 | 0.9278 | 1.0522 | 0.7315 | 0.7536 | | RP072 | LL_S_WS | 1.5437 | 1.0152 | 0.9162 | 0.7789 | 0.4412 | 0.5357 | | RP073 | LL_S_WS | 1.5437 | 0.2609 | 0.9289 | 0.7836 | 0.1158 | 0.6066 | | RP074 | LL_S_WS | 1.5437 | -0.0535 | 0.9227 | 0.8854 | 0.2413 | 0.6113 | | RP075 | LL_S_WS | 0.1588 | 0.3885 | 0.9195 | 1.1107 | 0.4738 | 0.7141 | | RP076 | LL_S_WS | 1.5437 | 0.1279 | 0.9889 | 0.9799 | 1.1583 | 0.6745 | | RP077 | LL_S_WS | 1.5437 | 1.8170 | 0.8546 | 1.1646 | 0.8687 | 0.8524 | | RP079 | LL_S_WS | 0.1814 | 0.1389 | 0.9301 | 0.9422 | 0.5515 | 0.5859 | | RP080 | LL_S_WS | 1.5437 | 3.6840 | 1.2303 | 1.3978 | 2.3165 | 2.1996 | | RP081 | LL_S_WS | 1.5437 | -0.1144 | 0.9002 | 0.5364 | -0.3658 | 0.5202 | | RP082 | LL_S_WS | 1.5437 | 3.6840 | 1.2303 | 1.3978 | 2.3165 | 2.1996 | | RP083 | LL_S_WS | 1.5437 | 3.6840 | 1.2303 | 1.3978 | 2.3165 | 2.1996 | | RP084 | LL_S_WS | 1.5437 | 0.6229 | 1.0417 | 0.9678 | 1.1119 | 0.7990 | | RP085 | LL_S_WS | 1.5437 | 0.4837 | 0.8830 | 0.5930 | -0.6515 | 0.6964 | | RP086 | LL_S_WS | 1.5437 | 0.3036 | 0.8789 | 0.9108 | 0.5577 | 0.6378 | | RP087 | LL_S_WS | 1.5437 | 0.7284 | 0.9306 | 0.7266 | 0.1737 | 0.6461 | | RP088 | LL_S_WS | 0.2070 | -0.1206 | 0.8731 | 0.4936 | 0.3159 | 0.5225 | | RP089 | LL_S_WS | 1.5437 | 0.7141 | 0.9387 | 1.1226 | 1.0193 | 0.6896 | | RP090 | LL_S_WS | 0.1199 | 0.0577 | 0.9360 | 1.1152 | 1.3030 | 0.8372 | | **Study designation** | **Condition** | **DTF_DSI** | **PH_DSI** | **BMDW_DSI** | **GY_DSI** | **TGW_DSI** | **SPKFT_DSI** | | RP091 | LL_S_WS | 1.5437 | 0.5961 | 0.8483 | 0.8910 | 0.4009 | 0.6856 | | RP092 | LL_S_WS | 0.1109 | -0.0633 | 0.7979 | 0.8031 | 1.9640 | 1.0275 | | RP093 | LL_S_WS | 0.0535 | 0.4442 | 0.9650 | 0.7446 | 0.4826 | 0.6749 | | RP094 | LL_S_WS | 0.1834 | 0.0137 | 0.9707 | 0.8862 | 0.1511 | 0.7037 | | RP095 | LL_S_WS | 0.2920 | -0.0874 | 0.9293 | 0.6339 | 0.0579 | 0.7856 | | RP096 | LL_S_WS | 1.5437 | 3.6840 | 1.2303 | 1.3978 | 2.3165 | 2.1996 | | RP097 | LL_S_WS | 0.1020 | 0.4000 | 0.9354 | 0.6920 | 0.0000 | 0.6800 | | RP098 | LL_S_WS | 0.2701 | 0.3593 | 0.8736 | 0.4821 | NA | 0.7281 | | RP099 | LL_S_WS | 1.5437 | 0.9718 | 0.9556 | 0.8217 | 0.5036 | 0.6402 | | RP100 | LL_S_WS | 0.2460 | 0.4931 | 0.9307 | 0.8228 | NA | 0.6349 | | RP101 | LL_S_WS | -0.1428 | 0.7703 | 1.0081 | 0.6904 | 1.0810 | 0.6246 | | RP102 | LL_S_WS | 0.0228 | 0.9570 | 0.8813 | 0.6345 | 0.5515 | 0.8080 | | RP103 | LL_S_WS | 1.5437 | 0.4889 | 1.0238 | 0.3966 | 0.1363 | 0.6186 | | RP104 | LL_S_WS | 1.5437 | 3.6840 | 1.2303 | 1.3978 | 2.3165 | 2.1996 | | RP105 | LL_S_WS | 1.5437 | 3.6840 | 1.2303 | 1.3978 | 2.3165 | 2.1996 | | RP106 | LL_S_WS | 0.0730 | 0.6238 | 0.9244 | 1.0208 | NA | 0.6896 | | RP107 | LL_S_WS | 0.1765 | 0.6336 | 0.9119 | 0.7285 | 1.2192 | 0.8818 | | RP108 | LL_S_WS | 1.5437 | 0.3597 | 0.8805 | 0.8100 | 0.1103 | 0.6438 | | RP109 | LL_S_WS | 0.1260 | 0.1625 | 0.8926 | 0.9345 | 0.1053 | 0.6540 | | RP110 | LL_S_WS | 1.5437 | 0.8910 | 0.8308 | 1.0252 | NA | 0.7998 | | RP111 | LL_S_WS | 1.5437 | -0.0703 | 0.7995 | 0.9509 | 0.5346 | 0.7699 | | RP112 | LL_S_WS | 0.1996 | 0.0796 | 0.9409 | 1.0252 | 2.2238 | 1.0030 | | RP113 | LL_S_WS | 1.5437 | 0.6048 | 0.8580 | 0.5510 | 1.5057 | 0.8534 | | RP114 | LL_S_WS | 0.2614 | 0.6339 | 0.9976 | 0.4308 | 0.1287 | 0.6437 | | RP115 | LL_S_WS | 1.5437 | 3.6840 | 1.2303 | 1.3978 | 2.3165 | 2.1996 | | RP116 | LL_S_WS | 1.5437 | 0.7816 | 0.9548 | 0.8123 | 0.2758 | 0.7900 | | RP117 | LL_S_WS | 1.5437 | 0.3833 | 0.9129 | 0.1170 | 0.0681 | 0.7347 | | RP118 | LL_S_WS | 1.5437 | 3.6840 | 1.2303 | 1.3978 | 2.3165 | 2.1996 | | **Study designation** | **Condition** | **DTF_DSI** | **PH_DSI** | **BMDW_DSI** | **GY_DSI** | **TGW_DSI** | **SPKFT_DSI** | | RP119 | LL_S_WS | 1.5437 | 0.5436 | 0.9862 | 0.7608 | 0.9376 | 0.6824 | | RP120 | LL_S_WS | 1.5437 | 0.5538 | 0.9621 | 0.9161 | NA | 0.7944 | | RP121 | LL_S_WS | 1.5437 | 1.4655 | 0.8469 | 0.7090 | -0.1007 | 0.5286 | | RP122 | LL_S_WS | 1.5437 | 0.3891 | 0.9725 | 0.9074 | NA | 0.5207 | | RP123 | LL_S_WS | 1.5437 | 3.6840 | 1.2303 | 1.3978 | 2.3165 | 2.1996 | | RP124 | LL_S_WS | 0.3055 | 0.4884 | 0.8634 | 0.5688 | NA | 0.6668 | | RP125 | LL_S_WS | 1.5437 | 0.4837 | 1.0325 | 0.6756 | 1.2590 | 0.7814 | | RP126 | LL_S_WS | 1.5437 | 3.6840 | 1.2303 | 1.3978 | 2.3165 | 2.1996 | | RP127 | LL_S_WS | 1.5437 | 3.6840 | 1.2303 | 1.3978 | 2.3165 | 2.1996 | | RP128 | LL_S_WS | 0.2365 | 0.4534 | 0.8454 | 0.4051 | 1.0135 | 0.7702 | | RP130 | LL_S_WS | -0.0087 | 0.3954 | 1.0109 | 1.0735 | 0.9928 | 0.8278 | | RP131 | LL_S_WS | 1.5437 | -0.0047 | 0.8513 | 1.1381 | 0.3861 | 0.6749 | | RP132 | LL_S_WS | 0.2435 | 0.3444 | 0.9957 | 0.4766 | 0.0000 | 0.8214 | | RP133 | LL_S_WS | 0.0952 | 0.5113 | 0.9120 | 1.0213 | 0.8687 | 0.7966 | | RP134 | LL_S_WS | 0.0733 | 0.4538 | 0.9998 | 0.9047 | 0.5791 | 0.8435 | | RP135 | LL_S_WS | 1.5437 | -0.3726 | 1.0031 | 0.9277 | 1.4892 | 0.7002 | | RP136 | LL_S_WS | 1.5437 | 3.6840 | 1.2303 | 1.3978 | NA | 2.1996 | | RP137 | LL_S_WS | 0.0825 | -0.5773 | 0.9245 | 0.7815 | 1.5636 | 0.8331 | | RP138 | LL_S_WS | 1.5437 | 0.1229 | 0.9105 | 0.6549 | 0.0927 | 0.8506 | | RP139 | LL_S_WS | 1.5437 | 0.2385 | 0.8668 | 0.6656 | 1.0424 | 0.8332 | | RP140 | LL_S_WS | 1.5437 | 0.7056 | 0.9103 | -0.0748 | 1.0939 | 0.5654 | | RP141 | LL_S_WS | 1.5437 | 0.6487 | 0.9892 | 0.7140 | 0.8561 | 0.6268 | | RP142 | LL_S_WS | 1.5437 | 0.7157 | 0.8566 | 0.8446 | NA | 0.8874 | | RP143 | LL_S_WS | 1.5437 | 0.6212 | 1.0403 | 0.3422 | 0.7315 | 0.5944 | | RP144 | LL_S_WS | 0.3214 | 0.4123 | 0.8007 | 0.7038 | 0.8534 | 0.6949 | | RP146 | LL_S_WS | 1.5437 | 0.8023 | 1.0539 | 0.1730 | NA | 0.6798 | | RP147 | LL_S_WS | 0.0496 | 0.3735 | 0.8927 | 0.7873 | 0.0000 | 0.6148 | | RP148 | LL_S_WS | 1.5437 | 1.0972 | 0.9133 | 0.9116 | 0.9652 | 0.7191 | | **Study designation** | **Condition** | **DTF_DSI** | **PH_DSI** | **BMDW_DSI** | **GY_DSI** | **TGW_DSI** | **SPKFT_DSI** | | RP149 | LL_S_WS | 1.5437 | 3.6840 | 1.2303 | 1.3978 | 2.3165 | 2.1996 | | RP150 | LL_S_WS | 1.5437 | 0.4992 | 0.8803 | 0.7976 | 0.8424 | 0.5914 | | RP151 | LL_S_WS | 0.0561 | 0.6818 | 0.9446 | 0.5547 | 0.2518 | 0.6836 | | RP152 | LL_S_WS | 1.5437 | 0.3300 | 0.9467 | 0.7471 | 2.2062 | 1.0302 | | RP153 | LL_S_WS | 1.5437 | 0.8253 | 0.9817 | 0.8471 | 0.4826 | 0.5775 | | RP154 | LL_S_WS | 0.1470 | 0.4176 | 0.9460 | 0.8925 | 1.1583 | 0.5498 | | RP155 | LL_S_WS | 1.5437 | 3.6840 | 1.2303 | 1.3978 | 2.3165 | 2.1996 | | RP156 | LL_S_WS | 1.5437 | 0.8436 | 0.9356 | 0.4192 | 0.4212 | 0.7542 | | RP157 | LL_S_WS | 1.5437 | 0.8365 | 0.9350 | 0.3519 | 0.6682 | 0.6320 | | RP158 | LL_S_WS | 1.5437 | 3.6840 | 1.2303 | 1.3978 | 2.3165 | 2.1996 | | RP159 | LL_S_WS | 0.2205 | -0.3990 | 0.9017 | 0.8613 | 0.3118 | 0.6213 | | RP160 | LL_S_WS | 0.2100 | -0.0995 | 0.8788 | 1.1961 | 1.6847 | 0.6258 | | RP161 | LL_S_WS | 0.2328 | -0.3538 | 0.9666 | 0.7597 | 0.8424 | 0.7602 | | RP162 | LL_S_WS | 1.5437 | 1.1769 | 0.9560 | -0.0316 | 0.3861 | 0.7100 | | RP164 | LL_S_WS | 1.5437 | 1.4511 | 0.9935 | 0.8456 | 0.6318 | 0.5726 | | RP165 | LL_S_WS | 0.3080 | 0.1003 | 0.8892 | 0.4906 | 1.7953 | 1.0282 | | RP166 | LL_S_WS | 1.5437 | 3.6840 | 1.2303 | 1.3978 | 2.3165 | 2.1996 | | RP167 | LL_S_WS | 1.5437 | 3.6840 | 1.2303 | 1.3978 | 2.3165 | 2.1996 | | RP168 | LL_S_WS | 0.1345 | 0.1998 | 0.9293 | 0.5722 | NA | 0.8154 | | RP169 | LL_S_WS | 1.5437 | 3.6840 | 1.2303 | 1.3978 | 2.3165 | 2.1996 | | RP170 | LL_S_WS | 1.5437 | -1.1227 | 0.9588 | 0.9747 | 1.5592 | 0.8679 | | RP171 | LL_S_WS | 1.5437 | 3.6840 | 1.2303 | 1.3978 | 2.3165 | 2.1996 | | RP172 | LL_S_WS | 0.1998 | 0.1421 | 0.8902 | 0.9315 | 0.2518 | 0.6568 | | RP173 | LL_S_WS | 1.5437 | 1.1249 | 0.9990 | 0.8948 | -0.1103 | 0.7873 | | RP174 | LL_S_WS | 1.5437 | 0.7983 | 0.9741 | 0.5951 | 1.9546 | 0.7348 | | RP175 | LL_S_WS | 1.5437 | 0.1869 | 0.8550 | 0.9783 | 1.2509 | 0.8808 | | RP176 | LL_S_WS | 1.5437 | 3.6840 | 1.2303 | 1.3978 | NA | 2.1996 | | RP177 | LL_S_WS | 1.5437 | -0.1603 | 0.8831 | 0.3071 | 0.5791 | 0.5168 | | **Study designation** | **Condition** | **DTF_DSI** | **PH_DSI** | **BMDW_DSI** | **GY_DSI** | **TGW_DSI** | **SPKFT_DSI** | | RP178 | LL_S_WS | 0.2399 | 0.7664 | 0.8829 | 0.7150 | 0.1007 | 0.5368 | | RP179 | LL_S_WS | 1.5437 | 3.6840 | 1.2303 | 1.3978 | 2.3165 | 2.1996 | | RP180 | LL_S_WS | 0.0665 | 0.6453 | 0.9465 | 1.0993 | 0.7446 | 0.8558 | | RP181 | LL_S_WS | 1.5437 | 0.1897 | 1.0633 | 0.5685 | 1.9601 | 1.0121 | | RP182 | LL_S_WS | 0.2061 | -0.0310 | 0.9679 | 0.9466 | 1.4701 | 0.6421 | | RP183 | LL_S_WS | 0.1334 | -0.6946 | 0.8060 | 1.1317 | 0.9266 | 0.8579 | | RP184 | LL_S_WS | 1.5437 | 0.4898 | 1.0156 | 1.2042 | 0.4343 | 0.6790 | | RP185 | LL_S_WS | 1.5437 | 3.6840 | 1.2303 | 1.3978 | NA | 2.1996 | | RP186 | LL_S_WS | 0.0500 | -0.4077 | 0.9847 | 0.6384 | -0.9009 | 0.9136 | | RP188 | LL_S_WS | 1.5437 | 3.6840 | 1.2303 | 1.3978 | 2.3165 | 2.1996 | | RP189 | LL_S_WS | 1.5437 | 3.6840 | 1.2303 | 1.3978 | 2.3165 | 2.1996 | | RP190 | LL_S_WS | 1.5437 | 0.5628 | 1.0294 | 0.5229 | 0.1007 | 0.6622 | | RP191 | LL_S_WS | 0.0749 | -0.0230 | 0.9771 | 1.0090 | NA | 0.6477 | | RP192 | LL_S_WS | 1.5437 | 3.6840 | 1.2303 | 1.3978 | 2.3165 | 2.1996 | | RP193 | LL_S_WS | 0.1903 | -0.1712 | 0.8414 | 0.1600 | 1.2972 | 0.8910 | | RP194 | LL_S_WS | 0.2577 | 0.7375 | 0.9487 | 1.0516 | 1.2686 | 0.8659 | | RP196 | LL_S_WS | 0.1963 | 0.2721 | 0.9726 | 0.4232 | -0.1511 | 0.5958 | | RP197 | LL_S_WS | 1.5437 | 1.6533 | 1.2303 | 0.2581 | 0.2896 | 0.7102 | | RP198 | LL_S_WS | 0.3422 | -0.1672 | 0.8655 | 0.8792 | 1.4604 | 0.7157 | | RP199 | LL_S_WS | 1.5437 | 3.6840 | 1.2303 | 1.3978 | NA | 2.1996 | | RP200 | LL_S_WS | 1.5437 | -0.6418 | 0.9219 | 1.1107 | 1.2590 | 1.0253 | | RP201 | LL_S_WS | 1.5437 | 0.3823 | 0.9669 | 1.0952 | 1.3810 | 0.7690 | | RP202 | LL_S_WS | 1.5437 | 0.4205 | 0.9998 | 0.6824 | 0.0579 | 0.8132 | | RP203 | LL_S_WS | 0.1512 | 0.4957 | 0.8871 | 1.0658 | 1.0246 | 0.7407 | | RP204 | LL_S_WS | 1.5437 | 0.8549 | 0.8949 | 1.1917 | 0.0552 | 0.5815 | | RP205 | LL_S_WS | 1.5437 | 1.3130 | 0.9473 | 1.1040 | 0.5309 | 0.4714 | | RP206 | LL_S_WS | 1.5437 | 3.6840 | 1.2303 | 1.3978 | 2.3165 | 2.1996 | | RP207 | LL_S_WS | 1.5437 | 0.1646 | 0.8870 | 0.8936 | -1.0810 | 0.6070 | | **Study designation** | **Condition** | **DTF_DSI** | **PH_DSI** | **BMDW_DSI** | **GY_DSI** | **TGW_DSI** | **SPKFT_DSI** | | RP208 | LL_S_WS | 0.2379 | 0.4318 | 0.8594 | 0.8429 | 1.0220 | 0.6399 | | RP209 | LL_S_WS | 0.2344 | 0.2480 | 0.9370 | 0.2408 | -0.3089 | 0.7328 | | RP210 | LL_S_WS | 0.2012 | -0.0396 | 1.0428 | 0.6579 | 1.0003 | 0.5579 | | RP211 | LL_S_WS | 1.5437 | 1.2318 | 1.0363 | 0.9125 | 0.7189 | 0.6804 | | RP212 | LL_S_WS | 1.5437 | 3.6840 | 1.2303 | 1.3978 | 2.3165 | 2.1996 | | RP213 | LL_S_WS | 1.5437 | 0.3729 | 0.9098 | 0.9270 | NA | 0.6093 | | RP214 | LL_S_WS | 1.5437 | 3.6840 | 1.2303 | 1.3978 | 2.3165 | 2.1996 | | RP215 | LL_S_WS | 1.5437 | 0.5213 | 0.9804 | 0.5160 | 0.9652 | 0.9702 | | RP216 | LL_S_WS | 0.2134 | 0.1893 | 0.8702 | 1.0105 | 0.2758 | 0.6614 | | RP217 | LL_S_WS | 1.5437 | 0.1603 | 0.9723 | 0.9264 | 0.8967 | 0.9115 | | RP218 | LL_S_WS | 1.5437 | 3.6840 | 1.2303 | 1.3978 | 2.3165 | 2.1996 | | RP219 | LL_S_WS | 0.0916 | 0.5862 | 1.0239 | 0.9578 | 1.3597 | 0.8705 | | RP220 | LL_S_WS | 0.0914 | -0.1582 | 0.9130 | 1.0415 | 0.9376 | 0.8891 | | RP222 | LL_S_WS | 1.5437 | 3.6840 | 1.2303 | 1.3978 | 2.3165 | 2.1996 | | RP223 | LL_S_WS | 1.5437 | 0.5827 | 0.9854 | 0.8437 | 0.7925 | 0.6428 | | RP224 | LL_S_WS | 0.1879 | -0.2949 | 0.8234 | 1.0187 | 0.5265 | 0.6951 | | RP225 | LL_S_WS | 1.5437 | -0.1280 | 0.9420 | 0.7854 | 0.6237 | 0.7511 | | RP226 | LL_S_WS | 0.2302 | 0.0068 | 0.8494 | 1.0725 | 1.4701 | 0.8729 | | RP228 | LL_S_WS | 1.5437 | -0.5338 | 0.8496 | 0.3127 | 0.9477 | 0.8180 | | RP229 | LL_S_WS | 0.1246 | 0.2533 | 0.8813 | 1.1254 | 0.3309 | 0.6303 | | RP230 | LL_S_WS | 1.5437 | 3.6840 | 1.2303 | 1.3978 | 2.3165 | 2.1996 | | RP231 | LL_S_WS | 0.1175 | 0.6089 | 1.0073 | 0.9558 | 0.1219 | 0.7474 | | RP232 | LL_S_WS | 0.1758 | -0.1421 | 0.9928 | 0.9779 | 0.3022 | 0.6140 | | RP233 | LL_S_WS | 0.3035 | 0.3042 | 0.8520 | 1.1719 | 0.8561 | 0.7219 | | RP234 | LL_S_WS | 0.2118 | 0.1732 | 0.9300 | 0.1790 | 0.5991 | 0.7446 | | RP235 | LL_S_WS | 1.5437 | 3.6840 | 1.2303 | 1.3978 | 2.3165 | 2.1996 | | RP237 | LL_S_WS | 0.1431 | 0.3591 | 0.7676 | 1.0094 | 0.9169 | 0.6457 | | RP238 | LL_S_WS | 1.5437 | 3.6840 | 1.2303 | 1.3978 | 2.3165 | 2.1996 | | **Study designation** | **Condition** | **DTF_DSI** | **PH_DSI** | **BMDW_DSI** | **GY_DSI** | **TGW_DSI** | **SPKFT_DSI** | | RP239 | LL_S_WS | 1.5437 | 3.6840 | 1.2303 | 1.3978 | 2.3165 | 2.1996 | | RP240 | LL_S_WS | 1.5437 | 0.6713 | 0.9463 | 0.8058 | 2.1621 | 0.9913 | | RP241 | LL_S_WS | 1.5437 | 0.6363 | 0.9441 | 0.7862 | 1.2919 | 0.7075 | | RP242 | LL_S_WS | 1.5437 | 0.3801 | 0.8892 | 0.9555 | 0.4633 | 0.5961 | | RP243 | LL_S_WS | 1.5437 | 3.6840 | 1.2303 | 1.3978 | 2.3165 | 2.1996 | | RP244 | LL_S_WS | 1.5437 | 3.6840 | 1.2303 | 1.3978 | NA | 2.1996 | | RP245 | LL_S_WS | 1.5437 | 3.6840 | 1.2303 | 1.3978 | 2.3165 | 2.1996 | | RP247 | LL_S_WS | 1.5437 | 3.6840 | 1.2303 | 1.3978 | 2.3165 | 2.1996 | | RP248 | LL_S_WS | 0.2375 | 0.5291 | 0.9718 | 0.7504 | NA | 0.7064 | | RP250 | LL_S_WS | 1.5437 | 3.6840 | 1.2303 | 1.3978 | 2.3165 | 2.1996 | | RP251 | LL_S_WS | 1.5437 | 0.1701 | 0.8515 | 0.8997 | -2.3165 | 0.4682 | | RP252 | LL_S_WS | 1.5437 | 3.6840 | 1.2303 | 1.3978 | NA | 2.1996 | | RP253 | LL_S_WS | 1.5437 | 1.1822 | 0.9560 | 0.4329 | 1.8953 | 0.8616 | | RP254 | LL_S_WS | 1.5437 | 0.5122 | 0.9256 | 0.5445 | 0.9266 | 0.6609 | | RP255 | LL_S_WS | 1.5437 | 0.3844 | 0.9229 | 1.1123 | -0.1287 | 0.6730 | | RP256 | LL_S_WS | 1.5437 | -0.4474 | 0.8813 | 0.9815 | -0.1930 | 0.6882 | | RP257 | LL_S_WS | 1.5437 | 0.8295 | 0.9658 | 0.2442 | 0.2172 | 0.7603 | | RP258 | LL_S_WS | 1.5437 | 1.0147 | 0.9951 | 0.9276 | 0.4170 | 0.7088 | | RP259 | LL_S_WS | 1.5437 | 3.6840 | 1.2303 | 1.3978 | 2.3165 | 2.1996 | | RP260 | LL_S_WS | 0.0979 | 0.9471 | 0.9523 | 0.6387 | 0.2317 | 0.7492 | | RP261 | LL_S_WS | 1.5437 | 3.6840 | 1.2303 | 1.3978 | 2.3165 | 2.1996 | | RP262 | LL_S_WS | 1.5437 | 3.6840 | 1.2303 | 1.3978 | NA | 2.1996 | | RP263 | LL_S_WS | 0.1692 | 0.0675 | 0.9506 | 0.7143 | 1.3093 | 0.7657 | | RP264 | LL_S_WS | 1.5437 | 1.4138 | 0.9483 | 0.8002 | -0.0643 | 0.7907 | | RP266 | LL_S_WS | 1.5437 | 3.6840 | 1.2303 | 1.3978 | 2.3165 | 2.1996 | | RP267 | LL_S_WS | 1.5437 | 0.5058 | 1.0172 | 0.2133 | 2.0269 | 0.8761 | | RP268 | LL_S_WS | 1.5437 | 1.1318 | 0.8831 | 1.0992 | 0.1737 | 0.7805 | | RP269 | LL_S_WS | 1.5437 | 0.3490 | 0.9987 | 0.2460 | 2.1510 | 1.0229 | | **Study designation** | **Condition** | **DTF_DSI** | **PH_DSI** | **BMDW_DSI** | **GY_DSI** | **TGW_DSI** | **SPKFT_DSI** | | RP270 | LL_S_WS | 0.1490 | 0.6298 | 0.8095 | 0.7510 | 0.7925 | 0.7916 | | RP272 | LL_S_WS | 0.0558 | 0.1420 | 0.9842 | 1.1172 | 0.7239 | 0.8352 | | RP273 | LL_S_WS | 0.1334 | 0.3849 | 0.9390 | 0.7256 | 0.5451 | 0.5788 | | RP274 | LL_S_WS | 1.5437 | 0.6515 | 0.9821 | 0.2388 | 1.3789 | 0.7978 | | RP275 | LL_S_WS | 1.5437 | 1.2746 | 0.7695 | 0.9508 | 1.1583 | 0.6303 | | RP276 | LL_S_WS | 1.5437 | 3.6840 | 1.2303 | 1.3978 | 2.3165 | 2.1996 | | RP277 | LL_S_WS | 1.5437 | 3.6840 | 1.2303 | 1.3978 | 2.3165 | 2.1996 | | RP278 | LL_S_WS | 1.5437 | 0.8092 | 0.9297 | 0.0660 | NA | 0.7098 | | RP279 | LL_S_WS | 0.1243 | 0.3635 | 0.9470 | 0.5067 | -1.3364 | 0.4785 | | RP280 | LL_S_WS | 1.5437 | 0.9566 | 0.9883 | 1.1207 | 2.2044 | 1.0319 | | RP281 | LL_S_WS | 1.5437 | 0.4711 | 0.9472 | 0.6577 | 0.7371 | 0.7176 | | RP282 | LL_S_WS | 0.2648 | 0.4109 | 1.0103 | 0.7913 | 1.8129 | 1.0013 | | RP283 | LL_S_WS | 0.0955 | 1.2051 | 0.9618 | 1.1193 | 0.6067 | 0.8831 | | RP284 | LL_S_WS | 1.5437 | -0.1608 | 0.8359 | 0.7138 | 0.3243 | 0.6722 | | RP285 | LL_S_WS | 0.1064 | -0.2807 | 0.9605 | 0.9232 | NA | 0.6566 | | RP286 | LL_S_WS | 1.5437 | -1.1529 | 0.9454 | 0.6469 | 1.4478 | 0.8865 | | RP287 | LL_S_WS | 1.5437 | 1.8919 | 0.9805 | 0.6367 | 0.6756 | 0.7150 | | RP288 | LL_S_WS | 0.1220 | 1.0594 | 0.9294 | 0.7373 | -0.9009 | 0.6422 | | RP289 | LL_S_WS | 0.1265 | 0.2849 | 0.8785 | 0.8558 | 1.2802 | 0.6459 | | RP290 | LL_S_WS | 1.5437 | 0.4638 | 0.9326 | 0.8368 | 0.2780 | 0.6775 | | RP291 | LL_S_WS | 0.0869 | 0.6201 | 0.9702 | 0.9481 | -0.3475 | 0.6015 | | RP292 | LL_S_WS | 1.5437 | 0.8280 | 1.0381 | 0.8772 | 0.8825 | 0.8329 | | RP293 | LL_S_WS | 1.5437 | 0.3785 | 0.8636 | 0.6586 | 0.6205 | 0.6341 | | RP294 | LL_S_WS | 0.2990 | -0.1393 | 0.8511 | 0.7807 | 0.6950 | 0.7176 | | RP295 | LL_S_WS | 0.3653 | -0.2732 | 0.8071 | 0.6248 | -0.7722 | 0.5802 | | RP296 | LL_S_WS | 1.5437 | -0.1914 | 0.7241 | 1.1816 | 0.8424 | 0.9640 | | RP297 | LL_S_WS | 1.5437 | 0.7571 | 0.9123 | 0.3732 | -0.5515 | 0.5113 | | RP298 | LL_S_WS | 0.1635 | 0.0688 | 0.9694 | 1.2215 | 0.9186 | 0.6371 | | **Study designation** | **Condition** | **DTF_DSI** | **PH_DSI** | **BMDW_DSI** | **GY_DSI** | **TGW_DSI** | **SPKFT_DSI** | | RP299 | LL_S_WS | 0.2229 | 0.1411 | 1.0167 | 0.7571 | NA | 0.7794 | | RP300 | LL_S_WS | 1.5437 | 0.7738 | 0.9144 | 1.0969 | 1.3899 | 0.8054 | | RP002 | UL_S_WS | 0.1293 | 1.9925 | 1.3254 | 1.0956 | 0.2780 | 1.3322 | | RP003 | UL_S_WS | 0.5071 | -1.8276 | 1.1117 | -0.0128 | 0.7361 | 1.4077 | | RP004 | UL_S_WS | 0.8898 | 0.7173 | 0.5383 | 0.8253 | 0.8008 | 0.0130 | | RP005 | UL_S_WS | 0.3095 | 2.2931 | 1.1133 | 1.0903 | 1.0920 | 1.1849 | | RP006 | UL_S_WS | 0.0385 | 1.7954 | 0.9384 | 0.6896 | 1.0974 | 0.0916 | | RP007 | UL_S_WS | 0.6486 | -0.1150 | 1.3686 | -0.5659 | 0.4573 | 0.9352 | | RP008 | UL_S_WS | 4.7029 | 7.2068 | 1.4942 | 2.0312 | 4.1150 | 2.7002 | | RP009 | UL_S_WS | 1.1448 | -0.1395 | 0.9983 | 0.9475 | -0.7481 | 0.3626 | | RP010 | UL_S_WS | 0.3461 | 2.0052 | NA | 0.8808 | NA | 1.8351 | | RP011 | UL_S_WS | 0.4841 | 0.7324 | -0.3573 | 1.2399 | 0.1819 | 0.3600 | | RP012 | UL_S_WS | 0.7378 | 1.6991 | 0.7989 | -0.4581 | NA | -0.1126 | | RP013 | UL_S_WS | 0.4281 | 1.7864 | 1.3442 | 1.2233 | 1.1628 | 1.0901 | | RP014 | UL_S_WS | 0.7487 | 1.3417 | 1.1424 | 1.1233 | 0.5582 | 0.8726 | | RP015 | UL_S_WS | 4.7029 | 7.2068 | 1.4942 | 2.0312 | 4.1150 | 2.7002 | | RP018 | UL_S_WS | 0.0555 | 2.3606 | 1.1388 | 0.4695 | 1.1737 | 0.8299 | | RP019 | UL_S_WS | 0.5297 | -0.0565 | 1.2607 | 0.7651 | -0.2413 | 1.4200 | | RP020 | UL_S_WS | -0.0561 | 0.9231 | 0.4868 | 1.1670 | 1.0697 | 0.1143 | | RP021 | UL_S_WS | 0.1774 | 0.5443 | 1.3564 | 0.8618 | -0.0419 | 0.4727 | | RP022 | UL_S_WS | 0.3568 | 1.2579 | 0.3693 | -0.3760 | 0.2533 | 0.5018 | | RP023 | UL_S_WS | 0.8147 | 1.2184 | 0.9049 | 1.0753 | 0.8054 | 0.1182 | | RP024 | UL_S_WS | 4.7029 | 7.2068 | 1.4942 | 2.0312 | 4.1150 | 2.7002 | | RP025 | UL_S_WS | 0.4937 | 1.1869 | 1.2068 | 1.0389 | 0.1672 | 1.1181 | | RP026 | UL_S_WS | 4.7029 | 7.2068 | 1.4942 | 2.0312 | 4.1150 | 2.7002 | | RP028 | UL_S_WS | 1.1749 | 0.8353 | 1.1407 | 1.4238 | 0.6354 | 0.2435 | | RP029 | UL_S_WS | 0.4755 | 1.0013 | 0.8905 | 1.4394 | 1.1432 | 0.4485 | | RP030 | UL_S_WS | 0.4600 | 0.8604 | 1.2063 | 1.0673 | 0.3773 | 1.4248 | | **Study designation** | **Condition** | **DTF_DSI** | **PH_DSI** | **BMDW_DSI** | **GY_DSI** | **TGW_DSI** | **SPKFT_DSI** | | RP031 | UL_S_WS | 0.8432 | 0.4287 | 0.7592 | 0.8441 | -8.3688 | 1.0665 | | RP032 | UL_S_WS | 1.0742 | 0.0169 | 1.4049 | 0.2837 | 0.8593 | 1.3750 | | RP033 | UL_S_WS | 0.2459 | 0.2974 | 1.4021 | 1.1035 | 0.8849 | 1.6606 | | RP034 | UL_S_WS | 0.1732 | 1.5244 | 1.2783 | 0.5823 | 0.7904 | 0.8518 | | RP036 | UL_S_WS | 0.4717 | 1.1021 | 1.0369 | 1.3057 | 0.8399 | 0.1986 | | RP037 | UL_S_WS | 0.5459 | 1.0835 | 1.0313 | 0.8840 | 0.9781 | -0.0118 | | RP038 | UL_S_WS | 0.8143 | 0.7908 | 1.2624 | 0.9536 | NA | 0.4910 | | RP039 | UL_S_WS | 4.7029 | 7.2068 | 1.4942 | 0.0312 | 4.1150 | 2.7002 | | RP040 | UL_S_WS | 0.4507 | -0.6699 | 0.1685 | 0.5663 | 1.0268 | 0.6993 | | RP041 | UL_S_WS | 0.6160 | -0.4828 | 0.9924 | -0.0562 | NA | 1.3929 | | RP042 | UL_S_WS | -0.2366 | -1.5920 | 0.1521 | 1.0701 | 0.8464 | 0.7187 | | RP043 | UL_S_WS | 0.3097 | -0.6877 | 1.2871 | 0.2196 | 0.6950 | 0.7254 | | RP044 | UL_S_WS | 0.8881 | -1.1723 | 0.5106 | 0.6703 | 0.7223 | 0.1576 | | RP045 | UL_S_WS | 0.6247 | -1.1413 | 0.9290 | 1.0978 | 0.6361 | 0.3756 | | RP046 | UL_S_WS | 4.7029 | 7.2068 | 1.4942 | 2.0312 | 4.1150 | 2.7002 | | RP047 | UL_S_WS | 0.5658 | 0.7473 | 1.1799 | 0.5839 | -0.2548 | 0.4742 | | RP048 | UL_S_WS | 0.4311 | 0.1281 | 0.8718 | 0.4639 | 0.6345 | 1.6174 | | RP049 | UL_S_WS | 0.1330 | 2.2014 | 0.8399 | 0.5006 | 0.9160 | 0.3285 | | RP050 | UL_S_WS | 0.6829 | 1.5965 | 0.6664 | 1.3668 | -0.5985 | 0.5543 | | RP051 | UL_S_WS | 1.1218 | 0.1551 | 0.1542 | 0.2709 | 0.1681 | 0.0535 | | RP052 | UL_S_WS | 0.5595 | -0.9543 | 0.8036 | 0.6517 | 0.8230 | 0.0632 | | RP053 | UL_S_WS | 0.3249 | -0.1500 | NA | 2.0312 | 1.3063 | 1.1297 | | RP054 | UL_S_WS | 0.3627 | 0.6823 | 1.3927 | 2.0312 | 0.8336 | 2.2778 | | RP055 | UL_S_WS | 0.7668 | -0.8329 | 0.7008 | 1.2330 | 0.9179 | 1.4399 | | RP056 | UL_S_WS | 0.5874 | -2.6481 | 1.0687 | -0.9164 | 1.0688 | 0.4599 | | RP057 | UL_S_WS | NA | 0.5739 | 1.4696 | -1.3599 | NA | 1.4776 | | RP058 | UL_S_WS | 0.9024 | 0.7424 | 0.5942 | 0.7040 | 1.8531 | 0.0726 | | RP059 | UL_S_WS | 0.4210 | -1.2927 | 0.7478 | -1.3198 | -3.8692 | 0.6153 | | **Study designation** | **Condition** | **DTF_DSI** | **PH_DSI** | **BMDW_DSI** | **GY_DSI** | **TGW_DSI** | **SPKFT_DSI** | | RP060 | UL_S_WS | 0.2829 | 1.7208 | 1.1963 | 0.8972 | 0.0468 | 1.4452 | | RP061 | UL_S_WS | -0.0585 | 0.5658 | NA | 1.2720 | 1.1825 | 0.8951 | | RP062 | UL_S_WS | 4.7029 | 7.2068 | 1.4942 | 2.0312 | 4.1150 | 2.7002 | | RP063 | UL_S_WS | -0.1859 | -1.8448 | NA | 0.4380 | 1.2398 | 1.8865 | | RP065 | UL_S_WS | 0.4565 | 0.6140 | 1.2147 | 0.8604 | NA | 1.7037 | | RP066 | UL_S_WS | 0.3919 | 0.0683 | 0.7318 | 1.1156 | -0.1448 | -0.1024 | | RP067 | UL_S_WS | 0.4155 | 1.6300 | 1.0560 | 1.5040 | 1.3747 | 1.7477 | | RP068 | UL_S_WS | 4.7029 | 7.2068 | 1.4942 | 2.0312 | 4.1150 | 2.7002 | | RP070 | UL_S_WS | 0.5501 | 0.2763 | 0.7541 | 0.7803 | -0.0119 | 0.2478 | | RP071 | UL_S_WS | 0.6620 | 0.9619 | 1.1697 | 1.2440 | 0.1035 | 1.1573 | | RP072 | UL_S_WS | 0.7881 | -0.2109 | 0.6401 | 0.5722 | 0.4089 | 0.4060 | | RP073 | UL_S_WS | 0.7925 | -1.3097 | 0.6233 | 0.5581 | 0.1290 | 0.1492 | | RP074 | UL_S_WS | 0.4155 | -1.9435 | 0.8829 | 0.8450 | 0.8666 | 1.7943 | | RP075 | UL_S_WS | 0.7925 | 0.9754 | 0.6165 | 1.3426 | 0.4515 | 0.5004 | | RP076 | UL_S_WS | 1.0305 | 0.2280 | 0.8865 | 1.0095 | 0.9339 | 0.0450 | | RP077 | UL_S_WS | 4.7029 | 7.2068 | 1.4942 | 2.0312 | 4.1150 | 2.7002 | | RP079 | UL_S_WS | -0.4163 | -1.7042 | 1.2511 | 0.9532 | 0.5943 | 1.3609 | | RP080 | UL_S_WS | 0.4084 | -0.7260 | 0.4849 | -0.3457 | -0.2266 | 0.3256 | | RP081 | UL_S_WS | 0.7592 | -4.5913 | 1.0903 | -0.0096 | 0.0701 | 0.1393 | | RP082 | UL_S_WS | 4.7029 | 7.2068 | 1.4942 | 2.0312 | 4.1150 | 2.7002 | | RP083 | UL_S_WS | 0.1330 | 0.5069 | 0.7911 | -1.4385 | -2.4306 | 0.3085 | | RP084 | UL_S_WS | 0.1745 | 2.1153 | 1.3958 | 1.0075 | 0.9443 | 0.5407 | | RP085 | UL_S_WS | 0.3535 | -3.9711 | 1.2588 | 0.1742 | -0.6277 | 1.2615 | | RP086 | UL_S_WS | NA | 0.5100 | 1.1591 | 0.8949 | 1.3926 | NA | | RP087 | UL_S_WS | 4.7029 | 7.2068 | 1.4942 | 2.0312 | 4.1150 | 2.7002 | | RP088 | UL_S_WS | 0.3168 | -3.5423 | 1.0289 | 0.0621 | 0.7894 | 1.4668 | | RP089 | UL_S_WS | 4.7029 | 7.2068 | 1.4942 | 2.0312 | 4.1150 | 2.7002 | | RP090 | UL_S_WS | 0.2562 | 0.7811 | 1.3475 | 1.3676 | 0.9079 | 0.7084 | | **Study designation** | **Condition** | **DTF_DSI** | **PH_DSI** | **BMDW_DSI** | **GY_DSI** | **TGW_DSI** | **SPKFT_DSI** | | RP091 | UL_S_WS | 0.1864 | 1.3185 | NA | 0.7998 | 1.2434 | 1.9129 | | RP092 | UL_S_WS | 0.0831 | 1.9546 | 0.8377 | 0.5874 | 0.9187 | 0.8550 | | RP093 | UL_S_WS | 0.6626 | 1.0287 | -0.1370 | 0.4721 | 0.7603 | 0.4872 | | RP094 | UL_S_WS | -0.3366 | 1.6638 | NA | 0.8215 | 0.6879 | 1.1744 | | RP095 | UL_S_WS | 0.5490 | 0.4481 | 1.1110 | 0.1799 | 0.4339 | 1.6459 | | RP096 | UL_S_WS | 0.6482 | -0.4128 | 1.2653 | 1.1937 | 1.0439 | 1.3710 | | RP097 | UL_S_WS | 0.7580 | -1.6894 | 0.7311 | 0.4454 | -0.3499 | 0.4074 | | RP098 | UL_S_WS | -0.0020 | -2.0308 | 0.7798 | -0.1636 | NA | 0.3085 | | RP099 | UL_S_WS | 0.3784 | -1.9498 | 0.9226 | 0.6657 | 0.7420 | 0.0876 | | RP100 | UL_S_WS | 0.3994 | 1.4575 | 1.1251 | 2.0312 | NA | 1.2073 | | RP101 | UL_S_WS | 0.8891 | -0.4479 | 0.6137 | 0.1041 | 1.4456 | -0.1500 | | RP102 | UL_S_WS | 1.1220 | -2.0673 | 0.0809 | 0.0593 | 0.3821 | -0.1529 | | RP103 | UL_S_WS | 0.4617 | -1.4698 | 1.1472 | -0.3212 | -0.5769 | 0.3233 | | RP104 | UL_S_WS | 0.7096 | -1.8926 | 0.6804 | -1.1493 | -0.0702 | 1.0958 | | RP105 | UL_S_WS | 0.4507 | -0.5614 | 1.2169 | -0.0186 | -0.8224 | 0.3851 | | RP106 | UL_S_WS | 0.7179 | 1.9052 | 0.8222 | 1.1644 | NA | 0.6436 | | RP107 | UL_S_WS | 0.3168 | 1.2438 | 0.8737 | 0.3454 | 0.0749 | 1.5773 | | RP108 | UL_S_WS | 0.5501 | -1.3280 | -0.8292 | 0.5677 | 0.3423 | 0.2095 | | RP109 | UL_S_WS | 0.7684 | 0.9348 | 0.9649 | 0.9155 | 0.5197 | -0.0858 | | RP110 | UL_S_WS | 0.6953 | 1.1175 | 0.3633 | 1.1393 | NA | 0.6285 | | RP111 | UL_S_WS | 0.4382 | 1.4190 | 0.4322 | 0.9928 | 1.1075 | 0.0482 | | RP112 | UL_S_WS | 4.7029 | 7.2068 | 1.4942 | 2.0312 | 4.1150 | 2.7002 | | RP113 | UL_S_WS | 4.7029 | 7.2068 | 1.4942 | 2.0312 | 4.1150 | 2.7002 | | RP114 | UL_S_WS | 0.1847 | -1.8370 | 1.2246 | -0.1932 | -0.1971 | 1.6831 | | RP115 | UL_S_WS | 0.5353 | 1.5214 | 0.9192 | 0.4504 | 1.5248 | 1.5075 | | RP116 | UL_S_WS | 0.1291 | -0.5747 | NA | 2.0312 | 0.8726 | 1.9649 | | RP117 | UL_S_WS | 0.4789 | -2.4551 | 1.0011 | -1.0093 | -0.4456 | 1.3733 | | RP118 | UL_S_WS | 0.3420 | 1.7314 | 1.3814 | -1.5261 | 0.6780 | 0.6289 | | **Study designation** | **Condition** | **DTF_DSI** | **PH_DSI** | **BMDW_DSI** | **GY_DSI** | **TGW_DSI** | **SPKFT_DSI** | | RP119 | UL_S_WS | 0.8658 | -0.3962 | 0.7294 | 0.2881 | 0.3571 | -0.1356 | | RP120 | UL_S_WS | 0.2907 | -1.7152 | 1.2262 | 0.8715 | NA | 1.3061 | | RP121 | UL_S_WS | -0.1390 | 1.3038 | 0.6055 | 0.5750 | 0.6697 | 0.7832 | | RP122 | UL_S_WS | 0.1577 | -2.8352 | 0.8336 | 0.8862 | NA | 0.3198 | | RP123 | UL_S_WS | 0.9792 | 0.1418 | 0.9551 | 1.0448 | 1.4294 | 1.2773 | | RP124 | UL_S_WS | 0.7935 | -1.2179 | 1.0093 | 0.1207 | NA | 2.4004 | | RP125 | UL_S_WS | 0.9647 | 0.9249 | 0.6698 | 0.1076 | 0.6950 | -0.0356 | | RP126 | UL_S_WS | 4.7029 | 7.2068 | 1.4942 | 2.0312 | 4.1150 | 2.7002 | | RP127 | UL_S_WS | 1.1337 | -4.6534 | 1.2076 | 1.5299 | 1.6487 | 1.5102 | | RP128 | UL_S_WS | 0.2206 | 0.3426 | 0.4864 | -0.3248 | 0.8325 | 1.4017 | | RP130 | UL_S_WS | 0.9185 | 1.8815 | 0.8599 | 1.1778 | 0.3837 | 0.2890 | | RP131 | UL_S_WS | 0.6250 | 1.8277 | 0.3687 | 1.4371 | 0.7645 | 0.4899 | | RP132 | UL_S_WS | 0.2893 | 1.0002 | 1.1647 | -0.2752 | 0.1889 | 0.3164 | | RP133 | UL_S_WS | 4.7029 | 7.2068 | 1.4942 | 2.0312 | 4.1150 | 2.7002 | | RP134 | UL_S_WS | 0.5687 | 0.5884 | 0.7704 | 0.8610 | 0.4731 | 0.3436 | | RP135 | UL_S_WS | 0.4441 | 1.4630 | 1.2337 | 0.9378 | 1.6592 | 1.7319 | | RP136 | UL_S_WS | 0.4728 | 1.3220 | 1.1667 | 0.9107 | NA | 1.4452 | | RP137 | UL_S_WS | 0.5219 | -1.3876 | 1.0953 | 0.5046 | 0.4129 | 1.2681 | | RP138 | UL_S_WS | 0.4155 | -2.1616 | 1.0606 | 0.3183 | 1.1646 | 1.4519 | | RP139 | UL_S_WS | 0.4211 | -4.8637 | 1.1455 | 0.2580 | 0.2375 | 2.0162 | | RP140 | UL_S_WS | 0.6341 | -2.4955 | 0.6404 | -1.5138 | -0.2576 | 0.1545 | | RP141 | UL_S_WS | 0.4719 | -1.3079 | 1.1817 | 0.4511 | 0.6480 | 0.6779 | | RP142 | UL_S_WS | 1.4342 | -1.6914 | 1.1186 | 0.6627 | NA | 1.3996 | | RP143 | UL_S_WS | 0.6433 | -1.2642 | 1.3574 | -0.4805 | -0.0300 | 0.6927 | | RP144 | UL_S_WS | 0.0986 | 1.2097 | 0.8149 | 0.3650 | -0.0275 | 0.2752 | | RP146 | UL_S_WS | 0.9843 | -0.5525 | 0.8854 | -0.8774 | NA | 0.9280 | | RP147 | UL_S_WS | 0.8485 | -1.1455 | 0.4303 | 0.5452 | 0.3441 | 0.0688 | | RP148 | UL_S_WS | 0.6156 | -0.5551 | 0.2828 | 0.8390 | -0.2755 | 0.5434 | | **Study designation** | **Condition** | **DTF_DSI** | **PH_DSI** | **BMDW_DSI** | **GY_DSI** | **TGW_DSI** | **SPKFT_DSI** | | RP149 | UL_S_WS | 0.9152 | -1.3681 | 1.0536 | 0.1361 | 2.0674 | 1.1984 | | RP150 | UL_S_WS | 0.6433 | 0.3628 | 0.4217 | 0.6473 | 0.5178 | 0.4009 | | RP151 | UL_S_WS | 0.5805 | -0.2406 | 1.0787 | 0.0636 | 1.2829 | 0.8480 | | RP152 | UL_S_WS | 0.9029 | -3.8408 | 0.8971 | 0.3075 | 0.5925 | 1.4169 | | RP153 | UL_S_WS | 0.6160 | -2.0546 | 0.8946 | 0.7481 | 0.9573 | 0.2147 | | RP154 | UL_S_WS | 0.2609 | -2.2589 | 0.6828 | 0.8061 | 0.7909 | 0.2682 | | RP155 | UL_S_WS | 1.0169 | -1.3563 | -0.0751 | 0.4032 | 1.0086 | 1.3393 | | RP156 | UL_S_WS | 0.8350 | -2.9409 | 0.8660 | -0.4542 | 0.5407 | 0.5140 | | RP157 | UL_S_WS | 1.0199 | -2.5473 | 0.6921 | -0.6918 | 1.1627 | 0.2514 | | RP158 | UL_S_WS | 0.5670 | 0.4632 | 0.8768 | 1.0407 | 0.9941 | 1.1632 | | RP159 | UL_S_WS | 0.2190 | 0.6779 | 1.0069 | 0.8447 | 1.2036 | 0.4549 | | RP160 | UL_S_WS | 0.0942 | 0.4207 | 0.7305 | 1.5390 | 0.5308 | 0.1233 | | RP161 | UL_S_WS | 0.1460 | -0.6601 | NA | 0.3887 | 0.5164 | 1.2561 | | RP162 | UL_S_WS | 4.7029 | 7.2068 | 1.4942 | 2.0312 | 4.1150 | 2.7002 | | RP164 | UL_S_WS | 0.2907 | 0.6300 | 1.3087 | 0.7505 | 0.5761 | 0.6548 | | RP165 | UL_S_WS | 4.7029 | 7.2068 | 1.4942 | 2.0312 | 4.1150 | 2.7002 | | RP166 | UL_S_WS | 0.5156 | -1.5460 | 0.9632 | -0.8907 | 0.8150 | 0.7458 | | RP167 | UL_S_WS | 0.4569 | 0.5986 | 0.5498 | 1.2527 | 0.3552 | -0.0181 | | RP168 | UL_S_WS | 0.7283 | 0.2137 | 1.2176 | 0.1407 | NA | 0.0561 | | RP169 | UL_S_WS | 1.3007 | -1.0297 | 0.9630 | 1.3750 | 1.4580 | 0.4923 | | RP170 | UL_S_WS | 0.7423 | -0.9606 | 1.0594 | 0.9981 | 1.1257 | 1.5414 | | RP171 | UL_S_WS | 0.3079 | 1.1343 | 0.9397 | 0.4532 | 0.8837 | 1.3141 | | RP172 | UL_S_WS | 0.1524 | 1.2642 | NA | 0.9514 | 0.6931 | 1.5252 | | RP173 | UL_S_WS | 0.1881 | 1.9801 | 1.1331 | 0.8813 | 0.3513 | 0.4846 | | RP174 | UL_S_WS | 0.4211 | 1.5967 | 1.2339 | 0.0474 | 1.7595 | 1.4371 | | RP175 | UL_S_WS | 0.7962 | -2.4199 | 0.5101 | 0.9383 | 0.8829 | 0.1063 | | RP176 | UL_S_WS | 4.7029 | 7.2068 | 1.4942 | 2.0312 | NA | 2.7002 | | RP177 | UL_S_WS | 0.1380 | -1.2659 | 0.8660 | -0.5686 | 0.0765 | 0.4070 | | **Study designation** | **Condition** | **DTF_DSI** | **PH_DSI** | **BMDW_DSI** | **GY_DSI** | **TGW_DSI** | **SPKFT_DSI** | | RP178 | UL_S_WS | -0.0020 | -0.1506 | -1.8788 | 0.5359 | 0.6075 | 0.5776 | | RP179 | UL_S_WS | 0.1005 | 2.0485 | 1.4420 | 0.9891 | 1.4905 | 0.6142 | | RP180 | UL_S_WS | 0.3741 | 0.4797 | 0.9042 | 1.3350 | 1.3531 | 0.5169 | | RP181 | UL_S_WS | -0.2730 | 2.5705 | 1.3557 | 0.0039 | 1.2005 | 1.2645 | | RP182 | UL_S_WS | 1.0824 | 1.5146 | 1.3164 | 0.9918 | 1.0573 | 0.8094 | | RP183 | UL_S_WS | 0.1294 | -0.5902 | 0.7880 | 1.3709 | 0.2089 | 2.0521 | | RP184 | UL_S_WS | 1.0349 | 1.1182 | 0.9277 | 1.5231 | 0.7983 | 0.1123 | | RP185 | UL_S_WS | 1.1503 | -2.2587 | 0.4869 | 0.8025 | NA | 0.0616 | | RP186 | UL_S_WS | 0.4238 | -0.8929 | 0.6747 | 0.1666 | -4.6653 | 0.8235 | | RP188 | UL_S_WS | 0.3754 | 1.2723 | 1.1032 | 1.0614 | 0.5296 | 1.3446 | | RP189 | UL_S_WS | 0.0831 | 1.9253 | 0.8369 | 0.7278 | 0.4751 | 1.3018 | | RP190 | UL_S_WS | -0.1960 | 0.4024 | NA | -0.0559 | 0.5986 | 0.5171 | | RP191 | UL_S_WS | 0.4593 | 0.5540 | 1.0788 | 1.1276 | NA | 1.0665 | | RP192 | UL_S_WS | 0.7660 | 1.0245 | 1.0136 | 1.2915 | 1.3835 | 0.3375 | | RP193 | UL_S_WS | 0.6953 | 0.6102 | 1.2208 | 0.4657 | 1.0195 | 0.4704 | | RP194 | UL_S_WS | 1.0199 | 0.6725 | 0.9209 | 1.1670 | 0.5994 | 1.3706 | | RP196 | UL_S_WS | 0.1813 | 0.5160 | 1.0265 | -0.1341 | 0.6680 | 0.4749 | | RP197 | UL_S_WS | 0.6709 | 1.4727 | 0.8969 | -0.7992 | 0.7232 | -0.0038 | | RP198 | UL_S_WS | 0.3332 | 1.0885 | 1.3301 | 0.7932 | 0.9450 | 1.5488 | | RP199 | UL_S_WS | 0.7365 | -3.2919 | 1.0233 | 0.8462 | NA | 1.3678 | | RP200 | UL_S_WS | 0.1790 | 0.1575 | 1.0766 | 1.3211 | 0.6904 | 0.2162 | | RP201 | UL_S_WS | 0.5397 | 1.3080 | 1.0547 | 1.2857 | 1.0575 | 0.7950 | | RP202 | UL_S_WS | 0.5241 | 0.8706 | 1.2614 | 0.2911 | 0.4397 | 1.5458 | | RP203 | UL_S_WS | 4.7029 | 7.2068 | 1.4942 | 2.0312 | 4.1150 | 2.7002 | | RP204 | UL_S_WS | 1.3033 | -1.1436 | 0.8514 | 1.5277 | 0.4148 | 1.2773 | | RP205 | UL_S_WS | 0.5402 | 1.2847 | 1.1053 | 1.3473 | 0.8249 | 0.2622 | | RP206 | UL_S_WS | 0.6806 | 1.9383 | 1.0134 | 0.4836 | -0.2855 | 0.7760 | | RP207 | UL_S_WS | 0.6620 | -6.4030 | 1.2067 | 0.8891 | -1.0454 | 0.1011 | | **Study designation** | **Condition** | **DTF_DSI** | **PH_DSI** | **BMDW_DSI** | **GY_DSI** | **TGW_DSI** | **SPKFT_DSI** | | RP208 | UL_S_WS | 0.4677 | -3.1990 | 0.0537 | 0.6009 | -0.5640 | -0.0865 | | RP209 | UL_S_WS | 0.8766 | -1.7304 | 1.0612 | -0.8502 | -1.1805 | 0.5130 | | RP210 | UL_S_WS | 0.5892 | -2.5512 | 1.0813 | 0.3933 | 0.6209 | -0.1807 | | RP211 | UL_S_WS | 0.9679 | 1.4874 | 0.7410 | 0.8415 | 1.3723 | 0.3214 | | RP212 | UL_S_WS | 0.8305 | 1.3525 | 0.9897 | 1.1037 | 0.9261 | 0.3269 | | RP213 | UL_S_WS | 0.4942 | 2.2460 | 1.0572 | 0.8961 | NA | 0.0268 | | RP214 | UL_S_WS | 0.4937 | 0.4963 | 1.2373 | 0.7532 | 0.3803 | 1.4118 | | RP215 | UL_S_WS | 0.8175 | 1.9709 | 1.3259 | -0.1231 | -2.4491 | 1.5877 | | RP216 | UL_S_WS | 0.6528 | 0.2816 | 0.5098 | 1.1316 | 0.6158 | 0.7783 | | RP217 | UL_S_WS | 0.2723 | 1.1258 | 1.1919 | 0.9000 | 1.6884 | 0.4030 | | RP218 | UL_S_WS | 4.7029 | 7.2068 | 1.4942 | 2.0312 | 4.1150 | 2.7002 | | RP219 | UL_S_WS | 0.8442 | 1.0480 | 1.0116 | 0.9532 | 0.6709 | 0.7744 | | RP220 | UL_S_WS | 0.6496 | 0.1966 | 0.2935 | 1.0738 | 0.2482 | -0.1253 | | RP222 | UL_S_WS | NA | -0.2963 | 1.3939 | 0.8945 | 0.1430 | 0.9012 | | RP223 | UL_S_WS | 0.6883 | 2.2419 | 1.2564 | 0.7050 | 0.2783 | 1.6538 | | RP224 | UL_S_WS | 0.0328 | -0.0721 | 0.2443 | 1.1459 | 0.5646 | 0.7643 | | RP225 | UL_S_WS | 0.1790 | 0.5995 | NA | 0.5785 | 1.3005 | 1.8165 | | RP226 | UL_S_WS | 0.4220 | 0.0254 | 0.8323 | 1.2388 | 1.2275 | 2.0567 | | RP228 | UL_S_WS | 1.1209 | -5.7047 | -0.1861 | -0.6713 | 0.4761 | -0.0846 | | RP229 | UL_S_WS | 0.5365 | 2.1303 | 0.4301 | 1.3368 | 0.2791 | 0.0840 | | RP230 | UL_S_WS | 0.2908 | -1.9415 | 1.3029 | -1.3541 | 0.8456 | -0.2234 | | RP231 | UL_S_WS | 0.3636 | -1.6034 | 1.2492 | 0.9532 | -0.0860 | 0.9011 | | RP232 | UL_S_WS | 0.5625 | -1.2968 | 1.1779 | 1.0233 | 0.6723 | 0.7083 | | RP233 | UL_S_WS | 0.0987 | 0.7837 | 0.9431 | 1.5013 | 0.8868 | 1.0242 | | RP234 | UL_S_WS | 0.6129 | 1.8656 | NA | 0.0312 | 1.6338 | 2.2391 | | RP235 | UL_S_WS | 0.6314 | -2.7587 | 0.6661 | 1.3788 | -1.5193 | 0.0679 | | RP237 | UL_S_WS | 0.2609 | 1.3156 | 0.8892 | 1.1004 | 0.9511 | 1.6347 | | RP238 | UL_S_WS | 0.3271 | -2.6240 | 1.3103 | 1.1445 | 1.1938 | 0.3029 | | **Study designation** | **Condition** | **DTF_DSI** | **PH_DSI** | **BMDW_DSI** | **GY_DSI** | **TGW_DSI** | **SPKFT_DSI** | | RP239 | UL_S_WS | 0.6979 | 1.7501 | 0.8914 | 0.8989 | 1.0264 | -0.0718 | | RP240 | UL_S_WS | 0.2734 | 0.6270 | 1.2586 | 0.5815 | -1.1692 | 0.9308 | | RP241 | UL_S_WS | 0.4792 | 0.3195 | 1.0268 | 0.6321 | 1.1525 | 0.5736 | | RP242 | UL_S_WS | 0.8589 | 0.1104 | 1.1708 | 0.9760 | 0.9728 | 1.2793 | | RP243 | UL_S_WS | 4.7029 | 7.2068 | 1.4942 | 2.0312 | 4.1150 | 2.7002 | | RP244 | UL_S_WS | 0.7596 | -0.0215 | 0.7622 | 0.8685 | NA | 0.4538 | | RP245 | UL_S_WS | 0.9021 | 1.5931 | -0.2711 | 0.8046 | 0.1463 | 0.8182 | | RP247 | UL_S_WS | -0.1739 | 0.6335 | NA | 0.4943 | 0.9918 | 1.6524 | | RP248 | UL_S_WS | 0.7058 | 0.1758 | 0.5221 | 0.3957 | NA | 0.3340 | | RP250 | UL_S_WS | 0.6830 | -5.3432 | 1.0584 | -0.6999 | -0.5120 | 0.4232 | | RP251 | UL_S_WS | 0.1971 | -1.7479 | 0.3328 | 0.8772 | -2.3925 | 0.1135 | | RP252 | UL_S_WS | 0.6732 | 1.0154 | 1.2101 | 0.8666 | NA | 0.3957 | | RP253 | UL_S_WS | 0.4601 | -3.0879 | 0.7734 | -0.3259 | 0.5411 | 0.3616 | | RP254 | UL_S_WS | 4.7029 | 7.2068 | 1.4942 | 2.0312 | 4.1150 | 2.7002 | | RP255 | UL_S_WS | 0.5208 | -1.1980 | 0.7002 | 1.3679 | -0.2517 | 0.2552 | | RP256 | UL_S_WS | 0.8891 | -0.4735 | 0.4453 | 0.9817 | -0.3225 | -0.0218 | | RP257 | UL_S_WS | 1.0488 | -3.1979 | 0.7947 | -0.7368 | -0.8318 | 0.6389 | | RP258 | UL_S_WS | 0.4539 | 0.7458 | 1.3494 | 0.9323 | 1.0449 | 1.2147 | | RP259 | UL_S_WS | 0.2908 | 2.2079 | 1.0403 | 1.3552 | 0.6612 | 0.7586 | | RP260 | UL_S_WS | 0.1406 | 1.3997 | 1.2921 | 0.2703 | 0.5075 | 1.7617 | | RP261 | UL_S_WS | 4.7029 | 7.2068 | 1.4942 | 2.0312 | 4.1150 | 2.7002 | | RP262 | UL_S_WS | 0.8457 | -1.9104 | 1.1116 | 0.8267 | NA | 1.3709 | | RP263 | UL_S_WS | 0.5874 | 0.1827 | 0.6807 | 0.3101 | 0.5881 | 0.0327 | | RP264 | UL_S_WS | 0.4899 | 1.0705 | 1.2633 | 0.6213 | 0.0218 | 1.3191 | | RP266 | UL_S_WS | 4.7029 | 7.2068 | 1.4942 | 2.0312 | 4.1150 | 2.7002 | | RP267 | UL_S_WS | 0.4568 | -2.0337 | 1.3043 | -0.8651 | 1.0669 | 2.0830 | | RP268 | UL_S_WS | 0.9790 | 1.1192 | 0.8636 | 1.2573 | 0.2879 | 0.6636 | | RP269 | UL_S_WS | 0.3880 | -3.1711 | 0.9127 | -0.8386 | -1.6005 | 0.9237 | | **Study designation** | **Condition** | **DTF_DSI** | **PH_DSI** | **BMDW_DSI** | **GY_DSI** | **TGW_DSI** | **SPKFT_DSI** | | RP270 | UL_S_WS | 0.3723 | 0.0913 | 1.0856 | 0.4842 | -0.0149 | 1.4415 | | RP272 | UL_S_WS | 0.4926 | 1.4165 | 1.1444 | 1.3247 | 1.0419 | 1.5482 | | RP273 | UL_S_WS | 0.6906 | 1.5798 | 0.8385 | 0.3750 | -0.5657 | -0.1665 | | RP274 | UL_S_WS | 0.1599 | -2.2606 | NA | -0.7543 | 0.5280 | 1.9935 | | RP275 | UL_S_WS | 0.3699 | 1.8333 | 0.6670 | 0.9439 | 1.2419 | 0.5254 | | RP276 | UL_S_WS | 0.3994 | 1.9058 | 1.2713 | 0.7289 | 0.0316 | 1.5314 | | RP277 | UL_S_WS | 0.8324 | 1.1596 | 0.6797 | 0.0694 | 0.0650 | -0.0122 | | RP278 | UL_S_WS | 0.4435 | 1.1825 | 1.3627 | -1.0908 | NA | 1.2067 | | RP279 | UL_S_WS | 0.6677 | -0.0217 | 0.5909 | -0.0697 | -1.6991 | -0.3134 | | RP280 | UL_S_WS | 0.7378 | -0.2950 | 1.0687 | 1.3277 | 1.5876 | 0.3370 | | RP281 | UL_S_WS | 0.4755 | -1.8927 | 0.8368 | 0.3072 | 0.5007 | 0.2652 | | RP282 | UL_S_WS | 0.7816 | 0.5594 | 1.2951 | 0.5448 | 0.6926 | 1.4412 | | RP283 | UL_S_WS | 0.5191 | 0.9196 | 0.8821 | 1.3492 | 0.4308 | 1.8116 | | RP284 | UL_S_WS | 4.7029 | 7.2068 | 1.4942 | 2.0312 | 4.1150 | 2.7002 | | RP285 | UL_S_WS | 4.7029 | 7.2068 | 1.4942 | 2.0312 | NA | 2.7002 | | RP286 | UL_S_WS | 1.0668 | -3.9570 | 0.9141 | 0.2307 | 0.8475 | 0.4497 | | RP287 | UL_S_WS | 0.8514 | -0.3155 | 0.9345 | 0.1986 | 0.9173 | 0.1831 | | RP288 | UL_S_WS | 0.3754 | 2.2325 | 1.2138 | 0.5497 | -0.2536 | 0.8079 | | RP289 | UL_S_WS | 0.9879 | 0.6508 | 0.9329 | 0.7027 | -0.1853 | 0.3240 | | RP290 | UL_S_WS | 0.7816 | 0.1815 | 1.0405 | 0.6303 | 1.0032 | 0.8112 | | RP291 | UL_S_WS | -0.1584 | 1.6892 | 1.3786 | 1.0026 | 0.1270 | 0.6644 | | RP292 | UL_S_WS | 4.7029 | 7.2068 | 1.4942 | 2.0312 | 4.1150 | 2.7002 | | RP293 | UL_S_WS | 0.5620 | -1.7108 | 1.2292 | 0.3013 | 1.3329 | 0.2987 | | RP294 | UL_S_WS | 4.7029 | 7.2068 | 1.4942 | 2.0312 | 4.1150 | 2.7002 | | RP295 | UL_S_WS | 0.0650 | -2.3298 | 0.1322 | 0.0245 | -1.2072 | -0.0902 | | RP296 | UL_S_WS | 4.7029 | 7.2068 | 1.4942 | 2.0312 | 4.1150 | 2.7002 | | RP297 | UL_S_WS | 0.6293 | -2.4698 | 0.9440 | -0.2889 | 0.4470 | 1.7724 | | RP298 | UL_S_WS | 4.7029 | 7.2068 | 1.4942 | 2.0312 | 4.1150 | 2.7002 | | **Study designation** | **Condition** | **DTF_DSI** | **PH_DSI** | **BMDW_DSI** | **GY_DSI** | **TGW_DSI** | **SPKFT_DSI** | | RP299 | UL_S_WS | 0.8669 | -2.1445 | 0.9738 | 0.4809 | NA | 0.4692 | | RP300 | UL_S_WS | 0.3627 | 1.3514 | 0.9226 | 1.2838 | 1.1059 | 0.8700 | | RP002 | LL_S_DS | NA | 1.0715 | 1.0300 | 1.0529 | 0.5924 | 1.0695 | | RP003 | LL_S_DS | 3.8347 | 2.0001 | 1.2554 | 1.0903 | 1.9033 | 1.6752 | | RP004 | LL_S_DS | NA | 0.7801 | 0.9186 | 1.0565 | 1.9033 | NA | | RP005 | LL_S_DS | 0.5070 | 0.7142 | 0.8161 | 0.7243 | 0.7479 | 0.5530 | | RP006 | LL_S_DS | -0.2610 | 0.7131 | 0.8079 | 1.0497 | 0.7923 | 0.8221 | | RP007 | LL_S_DS | 3.8347 | 2.0001 | 1.2554 | 1.0903 | 1.9033 | 1.6752 | | RP008 | LL_S_DS | -0.2157 | 0.7621 | 1.0679 | 1.0168 | 0.9342 | 0.8731 | | RP009 | LL_S_DS | -0.5450 | 0.8720 | 1.0461 | 1.0512 | 0.4378 | 1.1099 | | RP010 | LL_S_DS | -0.2458 | 0.9061 | 1.0288 | 1.0545 | NA | 0.6255 | | RP011 | LL_S_DS | -0.0274 | 0.7112 | 0.8632 | 1.0693 | 0.5134 | 1.0701 | | RP012 | LL_S_DS | 1.0271 | 0.8976 | 0.8460 | 0.4120 | NA | 0.5668 | | RP013 | LL_S_DS | -0.3090 | 0.9123 | 0.9306 | 1.0641 | 0.9219 | 0.8190 | | RP014 | LL_S_DS | 0.2612 | 0.8137 | 0.8338 | 0.7009 | 0.5656 | 0.8774 | | RP015 | LL_S_DS | 0.1318 | 1.1092 | 1.0507 | 1.0456 | 0.8128 | 0.7406 | | RP018 | LL_S_DS | 0.4301 | 0.7369 | 1.0235 | 1.0116 | 0.7479 | 0.7598 | | RP019 | LL_S_DS | NA | 0.6645 | 0.8878 | 1.0534 | 0.5402 | 1.0648 | | RP020 | LL_S_DS | 0.1919 | 0.6595 | 0.9855 | 0.9967 | 0.7923 | 0.7405 | | RP021 | LL_S_DS | NA | 0.6307 | 0.8923 | 1.0572 | 1.9033 | NA | | RP022 | LL_S_DS | -0.6870 | 0.8189 | 0.7348 | 1.0176 | 0.5406 | 1.1258 | | RP023 | LL_S_DS | 0.8396 | 0.8934 | 0.9732 | 0.9720 | 0.6091 | 0.5685 | | RP024 | LL_S_DS | 0.0752 | 0.7690 | 0.9527 | 1.0592 | 0.6711 | 0.8519 | | RP025 | LL_S_DS | -0.7676 | 0.7133 | 0.9502 | 1.0363 | 0.6307 | 1.0999 | | RP026 | LL_S_DS | 0.9811 | 1.0263 | 0.9294 | 1.0006 | 0.6314 | 0.9837 | | RP028 | LL_S_DS | -0.2022 | 1.0465 | 1.0481 | 1.0634 | 1.9033 | 0.7638 | | RP029 | LL_S_DS | -0.5715 | 0.8632 | 0.8887 | 1.0668 | 0.7745 | 0.7207 | | RP030 | LL_S_DS | -0.1501 | 0.9957 | 0.9689 | 1.0595 | 0.6055 | 1.0829 | | **Study designation** | **Condition** | **DTF_DSI** | **PH_DSI** | **BMDW_DSI** | **GY_DSI** | **TGW_DSI** | **SPKFT_DSI** | | RP031 | LL_S_DS | -0.6557 | 0.9325 | 0.9261 | 1.0595 | -2.1859 | 1.2406 | | RP032 | LL_S_DS | NA | 0.8675 | 1.0587 | 1.0359 | 0.8288 | 0.5234 | | RP033 | LL_S_DS | 0.6852 | 0.4908 | 0.8326 | 0.5432 | 0.6998 | 0.4757 | | RP034 | LL_S_DS | 0.5853 | 0.5806 | 0.9113 | 0.5866 | 0.7183 | 0.6119 | | RP036 | LL_S_DS | -0.7852 | 0.9397 | 0.8977 | 1.0543 | 0.7451 | 0.8379 | | RP037 | LL_S_DS | 0.6287 | 0.6211 | 0.9305 | 0.9135 | 0.7044 | 0.4426 | | RP038 | LL_S_DS | -0.1359 | 0.9252 | 0.9811 | 1.0389 | NA | 0.7649 | | RP039 | LL_S_DS | 0.3731 | 0.7051 | 0.9379 | 0.7964 | 0.7224 | 0.6767 | | RP040 | LL_S_DS | 0.2945 | 0.6052 | 0.9542 | 0.8226 | 0.8342 | 0.6007 | | RP041 | LL_S_DS | 0.0047 | 0.8201 | 0.8769 | 0.9371 | NA | 0.6516 | | RP042 | LL_S_DS | 0.5516 | 0.5020 | 0.7850 | 0.8685 | 0.7261 | 0.5398 | | RP043 | LL_S_DS | 3.8347 | 2.0001 | 1.2554 | 1.0903 | 1.9033 | 1.6752 | | RP044 | LL_S_DS | -0.4278 | 0.7572 | 0.9115 | 1.0427 | 0.6398 | 1.0262 | | RP045 | LL_S_DS | 0.2189 | 0.7971 | 1.0116 | 1.0513 | 0.7893 | 1.0727 | | RP046 | LL_S_DS | 0.1889 | 0.8246 | 0.8849 | 0.9898 | 0.8133 | 0.7415 | | RP047 | LL_S_DS | 0.9183 | 0.8183 | 0.9788 | 0.7221 | 0.3641 | 0.7058 | | RP048 | LL_S_DS | -0.4275 | 0.7017 | 1.0151 | 1.0262 | 0.6947 | 0.6196 | | RP049 | LL_S_DS | 0.0939 | 0.8031 | 0.8079 | 0.9646 | 0.6771 | 0.8903 | | RP050 | LL_S_DS | -0.6122 | 1.0213 | 0.9680 | 1.0672 | 0.3321 | 1.1534 | | RP051 | LL_S_DS | -0.1863 | 0.9940 | 0.9280 | 1.0129 | 0.7037 | 0.7132 | | RP052 | LL_S_DS | -0.6870 | 0.5533 | 0.9651 | 1.0331 | 0.7371 | 0.6132 | | RP053 | LL_S_DS | 0.2394 | 0.8732 | 0.8904 | 0.9503 | 0.8523 | 0.6829 | | RP054 | LL_S_DS | NA | 0.7627 | 0.8566 | 1.0249 | 0.7868 | 1.1476 | | RP055 | LL_S_DS | -0.4744 | 0.4753 | 0.7778 | 1.0648 | 0.8243 | 1.2198 | | RP056 | LL_S_DS | 0.2369 | 0.4355 | 1.0541 | 0.9601 | 0.7087 | 1.0231 | | RP057 | LL_S_DS | 3.8347 | 2.0001 | 1.2554 | 1.0903 | NA | 1.6752 | | RP058 | LL_S_DS | -0.1011 | 0.6411 | 0.9796 | 1.0425 | 1.0469 | 0.9614 | | RP059 | LL_S_DS | 0.6977 | 0.4747 | 1.0508 | 0.8382 | -0.4958 | 0.9065 | | **Study designation** | **Condition** | **DTF_DSI** | **PH_DSI** | **BMDW_DSI** | **GY_DSI** | **TGW_DSI** | **SPKFT_DSI** | | RP060 | LL_S_DS | 0.8130 | 0.7175 | 0.8693 | 0.8051 | 0.4975 | 0.6848 | | RP061 | LL_S_DS | 0.3229 | 0.7299 | 0.9719 | 1.0302 | 0.7714 | 1.0121 | | RP062 | LL_S_DS | -0.2473 | 0.6742 | 1.0111 | 1.0580 | 0.7464 | 0.8963 | | RP063 | LL_S_DS | NA | 0.6892 | 0.9488 | 1.0163 | 1.9033 | 1.1061 | | RP065 | LL_S_DS | 0.7547 | 0.6767 | 0.9020 | 0.8736 | NA | 0.6317 | | RP066 | LL_S_DS | -0.2949 | 0.7223 | 0.9256 | 1.0494 | 0.4969 | NA | | RP067 | LL_S_DS | 3.8347 | 2.0001 | 1.2554 | 1.0903 | 1.9033 | 1.6752 | | RP068 | LL_S_DS | 0.7865 | 0.8488 | 0.9425 | 0.7731 | 0.6398 | 0.5816 | | RP070 | LL_S_DS | NA | 0.8861 | 0.9905 | 1.0433 | 0.3830 | 0.7610 | | RP071 | LL_S_DS | -0.3262 | 0.9206 | 0.9193 | 1.0458 | 0.4588 | 0.7510 | | RP072 | LL_S_DS | 0.1284 | 0.7709 | 0.9712 | 1.0260 | 0.5360 | 0.6858 | | RP073 | LL_S_DS | -0.3097 | 0.5961 | 0.9272 | 1.0105 | 0.4862 | 0.9420 | | RP074 | LL_S_DS | -0.3655 | 0.6472 | 0.9243 | 1.0493 | 0.8810 | 0.8682 | | RP075 | LL_S_DS | -0.2471 | 0.9007 | 0.9284 | 1.0603 | 0.7634 | 1.0524 | | RP076 | LL_S_DS | NA | 0.9477 | 1.0171 | 1.0466 | 1.9033 | NA | | RP077 | LL_S_DS | -0.3724 | 0.7044 | 0.8167 | 1.0458 | 0.2332 | 1.0131 | | RP079 | LL_S_DS | -0.2127 | 0.6610 | 0.9468 | 1.0447 | 0.6055 | 1.0993 | | RP080 | LL_S_DS | 3.8347 | 2.0001 | 1.2554 | 1.0903 | 1.9033 | 1.6752 | | RP081 | LL_S_DS | NA | 0.4667 | 0.8644 | 0.9963 | 1.9033 | NA | | RP082 | LL_S_DS | 3.8347 | 2.0001 | 1.2554 | 1.0903 | 1.9033 | 1.6752 | | RP083 | LL_S_DS | NA | 0.9873 | 0.8991 | 0.9235 | 1.9033 | NA | | RP084 | LL_S_DS | 0.0859 | 0.9260 | 1.0536 | 1.0507 | 0.7696 | 1.1748 | | RP085 | LL_S_DS | NA | 0.6401 | 0.8814 | 1.0294 | 0.3522 | 1.2837 | | RP086 | LL_S_DS | 0.6548 | 0.7535 | 0.8798 | 0.9594 | 0.7932 | 0.5390 | | RP087 | LL_S_DS | 0.1126 | 0.4427 | 0.9472 | 1.0269 | 0.5406 | 1.0405 | | RP088 | LL_S_DS | 0.5657 | 0.3862 | 0.8810 | 0.7618 | 0.6558 | 0.5574 | | RP089 | LL_S_DS | -0.5511 | 0.9758 | 0.9423 | 1.0685 | 0.7837 | 0.8919 | | RP090 | LL_S_DS | NA | 0.8152 | 0.9491 | 1.0636 | 0.6544 | 0.9148 | | **Study designation** | **Condition** | **DTF_DSI** | **PH_DSI** | **BMDW_DSI** | **GY_DSI** | **TGW_DSI** | **SPKFT_DSI** | | RP091 | LL_S_DS | 0.6216 | 0.9778 | 0.8478 | 0.9535 | 0.7714 | 0.8441 | | RP092 | LL_S_DS | 0.1685 | 0.9600 | 0.7707 | 1.0382 | 0.8129 | 1.0436 | | RP093 | LL_S_DS | -0.3749 | 0.7949 | 0.9764 | 1.0199 | 0.8357 | 1.1214 | | RP094 | LL_S_DS | 0.6488 | 0.8110 | 0.9719 | 1.0039 | 0.6947 | 0.7576 | | RP095 | LL_S_DS | 0.7167 | 0.7190 | 0.9269 | 0.5143 | 0.5678 | 0.4793 | | RP096 | LL_S_DS | 3.8347 | 2.0001 | 1.2554 | 1.0903 | 1.9033 | 1.6752 | | RP097 | LL_S_DS | -0.6381 | 0.6002 | 0.9356 | 1.0395 | 0.5402 | 0.8582 | | RP098 | LL_S_DS | 1.0541 | 0.6566 | 0.8768 | 0.4010 | NA | 0.6386 | | RP099 | LL_S_DS | NA | 0.9022 | 0.9817 | 1.0399 | 0.8129 | 0.9203 | | RP100 | LL_S_DS | 0.2156 | 1.0844 | 0.9469 | 1.0489 | NA | 1.1563 | | RP101 | LL_S_DS | -0.4419 | 1.0522 | 1.0246 | 1.0432 | 1.0126 | 0.9732 | | RP102 | LL_S_DS | NA | 0.9376 | 0.8913 | 1.0439 | 0.8079 | 1.2856 | | RP103 | LL_S_DS | NA | 0.8861 | 1.0324 | 1.0088 | 0.2681 | 1.0949 | | RP104 | LL_S_DS | 3.8347 | 2.0001 | 1.2554 | 1.0903 | 1.9033 | 1.6752 | | RP105 | LL_S_DS | 3.8347 | 2.0001 | 1.2554 | 1.0903 | 1.9033 | 1.6752 | | RP106 | LL_S_DS | 0.8476 | 0.9608 | 0.9281 | 0.9052 | NA | 0.8253 | | RP107 | LL_S_DS | -0.4802 | 1.0742 | 0.9189 | 1.0345 | 0.4689 | 0.9544 | | RP108 | LL_S_DS | NA | 0.4818 | 0.8943 | 1.0281 | 1.9033 | 1.1321 | | RP109 | LL_S_DS | 0.7167 | 0.6521 | 0.8892 | 0.7411 | 0.5656 | 0.5209 | | RP110 | LL_S_DS | -0.6906 | 0.8640 | 0.8262 | 1.0416 | NA | 0.5330 | | RP111 | LL_S_DS | NA | 0.8593 | 0.7203 | 1.0449 | 1.9033 | NA | | RP112 | LL_S_DS | -0.6589 | 1.0081 | 0.9636 | 1.0602 | 0.9437 | 1.1105 | | RP113 | LL_S_DS | -0.3256 | 0.5443 | 0.8620 | 1.0153 | 0.6494 | 0.9202 | | RP114 | LL_S_DS | 0.0639 | 0.8542 | 1.0504 | 0.7042 | 0.4798 | 0.9356 | | RP115 | LL_S_DS | 3.8347 | 2.0001 | 1.2554 | 1.0903 | 1.9033 | 1.6752 | | RP116 | LL_S_DS | -0.1705 | 0.8705 | 0.9633 | 1.0253 | 0.6055 | 0.7462 | | RP117 | LL_S_DS | NA | 0.8901 | 0.9240 | 0.9902 | 1.9033 | 1.1841 | | RP118 | LL_S_DS | 0.4206 | 0.7293 | 0.8966 | 0.6189 | 0.5170 | 0.4339 | | **Study designation** | **Condition** | **DTF_DSI** | **PH_DSI** | **BMDW_DSI** | **GY_DSI** | **TGW_DSI** | **SPKFT_DSI** | | RP119 | LL_S_DS | NA | 0.5761 | 0.9865 | 1.0345 | 0.6568 | 0.8901 | | RP120 | LL_S_DS | NA | 0.8415 | 0.9701 | 1.0512 | NA | 0.7488 | | RP121 | LL_S_DS | NA | 0.9738 | 0.8008 | 1.0163 | 0.6864 | 0.7975 | | RP122 | LL_S_DS | 0.2422 | 0.4621 | 0.9877 | 1.0305 | NA | 0.9134 | | RP123 | LL_S_DS | 3.8347 | 2.0001 | 1.2554 | 1.0903 | 1.9033 | 1.6752 | | RP124 | LL_S_DS | 1.1061 | 0.5854 | 0.8655 | 0.3796 | NA | 0.5114 | | RP125 | LL_S_DS | -0.1195 | 0.9117 | 1.0570 | 1.0157 | 0.6711 | 0.4739 | | RP126 | LL_S_DS | 3.8347 | 2.0001 | 1.2554 | 1.0903 | 1.9033 | 1.6752 | | RP127 | LL_S_DS | 3.8347 | 2.0001 | 1.2554 | 1.0903 | 1.9033 | 1.6752 | | RP128 | LL_S_DS | 0.9904 | 0.7588 | 0.8410 | 0.3020 | 0.6771 | 0.5197 | | RP130 | LL_S_DS | -0.5972 | 1.0474 | 1.0198 | 1.0658 | 0.8079 | 1.1168 | | RP131 | LL_S_DS | NA | 0.8502 | 0.8457 | 1.0768 | 1.0170 | 0.8936 | | RP132 | LL_S_DS | 0.8238 | 0.7651 | 1.0033 | 0.4624 | 0.3830 | 0.5502 | | RP133 | LL_S_DS | 0.2642 | 0.8698 | 0.9168 | 1.0074 | 0.6222 | 0.8806 | | RP134 | LL_S_DS | NA | 1.0520 | 1.0230 | 1.0400 | 0.6887 | 0.9236 | | RP135 | LL_S_DS | 0.7450 | 0.6606 | 1.0049 | 1.0103 | 0.8911 | 0.7365 | | RP136 | LL_S_DS | 3.8347 | 2.0001 | 1.2554 | 1.0903 | NA | 1.6752 | | RP137 | LL_S_DS | -0.3885 | 0.8060 | 0.9278 | 1.0421 | 0.7259 | 1.2694 | | RP138 | LL_S_DS | NA | 0.7113 | 0.8990 | 1.0249 | 0.7294 | 0.8154 | | RP139 | LL_S_DS | -0.0865 | 0.5741 | 0.8689 | 0.8870 | 0.6765 | 0.7841 | | RP140 | LL_S_DS | -0.1719 | 0.5708 | 0.9043 | 0.9283 | 0.3836 | 1.1327 | | RP141 | LL_S_DS | NA | 0.9041 | 0.9650 | 1.0088 | 0.7183 | 1.1493 | | RP142 | LL_S_DS | -0.0097 | 0.7301 | 0.8564 | 1.0270 | NA | 0.7893 | | RP143 | LL_S_DS | -0.3097 | 0.4097 | 1.0533 | 0.8690 | 0.4975 | 0.7986 | | RP144 | LL_S_DS | 0.9255 | 0.6324 | 0.7764 | 0.2905 | 0.4689 | 0.5255 | | RP146 | LL_S_DS | NA | 0.8706 | 1.0758 | 1.0105 | NA | 1.1485 | | RP147 | LL_S_DS | -0.3380 | 0.7542 | 0.8992 | 1.0380 | 0.5537 | 1.0967 | | RP148 | LL_S_DS | NA | 0.8001 | 0.9150 | 1.0172 | 0.3786 | 0.6517 | | **Study designation** | **Condition** | **DTF_DSI** | **PH_DSI** | **BMDW_DSI** | **GY_DSI** | **TGW_DSI** | **SPKFT_DSI** | | RP149 | LL_S_DS | 3.8347 | 2.0001 | 1.2554 | 1.0903 | 1.9033 | 1.6752 | | RP150 | LL_S_DS | -0.4278 | 0.9582 | 0.8953 | 1.0221 | 0.7634 | 0.8143 | | RP151 | LL_S_DS | -0.3179 | 0.9312 | 0.9635 | 1.0090 | 0.6825 | 0.7523 | | RP152 | LL_S_DS | NA | 0.6272 | 0.9619 | 1.0467 | 0.7609 | 1.2005 | | RP153 | LL_S_DS | 0.4277 | 0.8010 | 1.0234 | 0.9941 | 0.7224 | 1.0454 | | RP154 | LL_S_DS | NA | 0.7517 | 0.9504 | 1.0446 | 0.8366 | 0.9858 | | RP155 | LL_S_DS | 3.8347 | 2.0001 | 1.2554 | 1.0903 | 1.9033 | 1.6752 | | RP156 | LL_S_DS | 0.4165 | 0.7112 | 0.9622 | 1.0129 | 0.8870 | 0.9023 | | RP157 | LL_S_DS | -0.6605 | 0.6940 | 1.0016 | 0.9681 | 1.9033 | 0.9143 | | RP158 | LL_S_DS | 3.8347 | 2.0001 | 1.2554 | 1.0903 | 1.9033 | 1.6752 | | RP159 | LL_S_DS | -0.0774 | 0.7218 | 0.9078 | 1.0355 | 0.8551 | 0.9367 | | RP160 | LL_S_DS | 0.0142 | 0.9011 | 0.8670 | 1.0710 | 0.7634 | 0.8267 | | RP161 | LL_S_DS | -0.1079 | 0.6954 | 0.9743 | 0.9416 | 0.7139 | 0.5533 | | RP162 | LL_S_DS | NA | 0.7263 | 0.9577 | 0.9777 | 0.8655 | NA | | RP164 | LL_S_DS | -0.5032 | 0.9046 | 0.9913 | 1.0466 | 0.7386 | 1.1417 | | RP165 | LL_S_DS | 1.2332 | 0.7522 | 0.8885 | 0.1940 | 0.5134 | 0.4954 | | RP166 | LL_S_DS | 3.8347 | 2.0001 | 1.2554 | 1.0903 | 1.9033 | 1.6752 | | RP167 | LL_S_DS | 3.8347 | 2.0001 | 1.2554 | 1.0903 | 1.9033 | 1.6752 | | RP168 | LL_S_DS | 0.3950 | 0.6644 | 0.9375 | 0.8466 | NA | 0.6104 | | RP169 | LL_S_DS | 3.8347 | 2.0001 | 1.2554 | 1.0903 | 1.9033 | 1.6752 | | RP170 | LL_S_DS | NA | 0.5158 | 0.9750 | 1.0383 | 0.9806 | 0.8564 | | RP171 | LL_S_DS | 3.8347 | 2.0001 | 1.2554 | 1.0903 | 1.9033 | 1.6752 | | RP172 | LL_S_DS | 0.0192 | 0.9631 | 0.8834 | 1.0372 | 0.7183 | 0.8403 | | RP173 | LL_S_DS | NA | 1.2346 | 0.9907 | 1.0378 | 0.8121 | NA | | RP174 | LL_S_DS | NA | 1.0392 | 0.9774 | 1.0431 | 1.9033 | NA | | RP175 | LL_S_DS | 0.4921 | -0.0339 | 0.7122 | 1.0256 | 0.7330 | 1.1041 | | RP176 | LL_S_DS | 3.8347 | 2.0001 | 1.2554 | 1.0903 | NA | 1.6752 | | RP177 | LL_S_DS | 0.4510 | 0.1653 | 0.8111 | 0.8326 | 0.3231 | 0.5268 | | **Study designation** | **Condition** | **DTF_DSI** | **PH_DSI** | **BMDW_DSI** | **GY_DSI** | **TGW_DSI** | **SPKFT_DSI** | | RP178 | LL_S_DS | 1.0541 | 0.7189 | 0.8934 | 0.7480 | 0.6474 | 0.5550 | | RP179 | LL_S_DS | 3.8347 | 2.0001 | 1.2554 | 1.0903 | 1.9033 | 1.6752 | | RP180 | LL_S_DS | -0.6757 | 0.9115 | 0.9594 | 1.0660 | 1.0076 | 0.9608 | | RP181 | LL_S_DS | NA | 1.0548 | 1.0837 | 1.0232 | 1.0433 | 0.9118 | | RP182 | LL_S_DS | 0.7224 | 0.7022 | 0.9658 | 0.7614 | 0.7714 | 0.5418 | | RP183 | LL_S_DS | 0.4400 | 0.6389 | 0.7740 | 1.0503 | 0.5950 | 0.9457 | | RP184 | LL_S_DS | NA | 0.9223 | 1.0011 | 1.0685 | 1.9033 | 0.8895 | | RP185 | LL_S_DS | 3.8347 | 2.0001 | 1.2554 | 1.0903 | NA | 1.6752 | | RP186 | LL_S_DS | 0.5422 | 0.4758 | 0.9947 | 0.8326 | -0.7020 | 0.7566 | | RP188 | LL_S_DS | 3.8347 | 2.0001 | 1.2554 | 1.0903 | 1.9033 | 1.6752 | | RP189 | LL_S_DS | 3.8347 | 2.0001 | 1.2554 | 1.0903 | 1.9033 | 1.6752 | | RP190 | LL_S_DS | NA | 0.7958 | 1.0376 | 0.9949 | 0.7420 | 1.1628 | | RP191 | LL_S_DS | -0.5343 | 0.9644 | 0.9914 | 1.0682 | NA | 0.9755 | | RP192 | LL_S_DS | 3.8347 | 2.0001 | 1.2554 | 1.0903 | 1.9033 | 1.6752 | | RP193 | LL_S_DS | 0.5982 | 0.6131 | 0.8343 | 0.9477 | 0.7261 | 0.6025 | | RP194 | LL_S_DS | 0.5296 | 0.9393 | 0.9626 | 1.0245 | 0.6055 | 0.6716 | | RP196 | LL_S_DS | 0.7031 | 0.8029 | 0.9536 | 0.9319 | 0.7656 | 1.0337 | | RP197 | LL_S_DS | NA | 0.9739 | 0.9043 | 0.9736 | 1.9033 | 1.1064 | | RP198 | LL_S_DS | 1.1722 | 0.7370 | 0.8699 | 0.8446 | 0.6238 | 0.5692 | | RP199 | LL_S_DS | 3.8347 | 2.0001 | 1.2554 | 1.0903 | NA | 1.6752 | | RP200 | LL_S_DS | -0.1374 | 0.6194 | 0.9294 | 1.0102 | 0.7656 | 0.7159 | | RP201 | LL_S_DS | NA | 0.7747 | 0.9505 | 1.0663 | 1.0186 | NA | | RP202 | LL_S_DS | NA | 0.6856 | 0.9924 | 1.0110 | 0.6494 | NA | | RP203 | LL_S_DS | 0.2802 | 0.6315 | 0.8972 | 0.9835 | 0.6250 | 0.5913 | | RP204 | LL_S_DS | NA | 0.4878 | 0.8710 | 1.0615 | 0.5402 | NA | | RP205 | LL_S_DS | NA | 0.8312 | 0.9436 | 1.0600 | 1.9033 | NA | | RP206 | LL_S_DS | 3.8347 | 2.0001 | 1.2554 | 1.0903 | 1.9033 | 1.6752 | | RP207 | LL_S_DS | -0.4664 | 0.4798 | 0.9086 | 1.0587 | 0.2005 | 1.1600 | | **Study designation** | **Condition** | **DTF_DSI** | **PH_DSI** | **BMDW_DSI** | **GY_DSI** | **TGW_DSI** | **SPKFT_DSI** | | RP208 | LL_S_DS | 0.4969 | 0.6398 | 0.8528 | 1.0185 | 0.3001 | 0.7739 | | RP209 | LL_S_DS | 0.6732 | 0.5963 | 0.9346 | -0.1940 | 0.1226 | 0.5584 | | RP210 | LL_S_DS | 0.0088 | 0.7532 | 1.0662 | 1.0256 | 0.7881 | 0.5167 | | RP211 | LL_S_DS | -0.5462 | 0.7706 | 0.9689 | 1.0453 | 0.8510 | 0.9407 | | RP212 | LL_S_DS | 3.8347 | 2.0001 | 1.2554 | 1.0903 | 1.9033 | 1.6752 | | RP213 | LL_S_DS | NA | 0.9557 | 0.8499 | 1.0390 | NA | 1.1247 | | RP214 | LL_S_DS | 3.8347 | 2.0001 | 1.2554 | 1.0903 | 1.9033 | 1.6752 | | RP215 | LL_S_DS | -0.4445 | 0.7121 | 0.9909 | 1.0314 | -0.1866 | 0.5672 | | RP216 | LL_S_DS | -0.1865 | 0.9147 | 0.8631 | 1.0032 | 0.7609 | 1.0824 | | RP217 | LL_S_DS | NA | 0.7342 | 0.9954 | 1.0371 | 1.9033 | NA | | RP218 | LL_S_DS | 3.8347 | 2.0001 | 1.2554 | 1.0903 | 1.9033 | 1.6752 | | RP219 | LL_S_DS | -0.4261 | 1.0170 | 1.0276 | 1.0572 | 0.8480 | 1.1209 | | RP220 | LL_S_DS | NA | 0.8741 | 0.9187 | 1.0504 | 0.5662 | 1.1412 | | RP222 | LL_S_DS | 3.8347 | 2.0001 | 1.2554 | 1.0903 | 1.9033 | 1.6752 | | RP223 | LL_S_DS | 0.1244 | 1.2012 | 0.9945 | 1.0451 | 0.6692 | 0.8896 | | RP224 | LL_S_DS | 0.5018 | 0.4589 | 0.8050 | 0.8802 | 0.5656 | 0.6728 | | RP225 | LL_S_DS | 0.5649 | 0.5666 | 0.9378 | 0.9044 | 0.7296 | 0.7067 | | RP226 | LL_S_DS | 0.9018 | 0.1896 | 0.8371 | 0.9540 | 0.6878 | 0.7067 | | RP228 | LL_S_DS | 0.5088 | 0.4952 | 0.8522 | 0.9453 | 0.7386 | 0.8354 | | RP229 | LL_S_DS | 0.7098 | 0.8997 | 0.8745 | 0.9891 | 0.5278 | 0.7103 | | RP230 | LL_S_DS | 3.8347 | 2.0001 | 1.2554 | 1.0903 | 1.9033 | 1.6752 | | RP231 | LL_S_DS | -0.5356 | 0.8222 | 1.0097 | 1.0327 | 0.6120 | 0.6724 | | RP232 | LL_S_DS | 0.3088 | 0.7293 | 1.0068 | 1.0496 | 0.7101 | 0.5029 | | RP233 | LL_S_DS | 0.5516 | 0.5901 | 0.8292 | 0.9868 | 0.6474 | 0.7523 | | RP234 | LL_S_DS | 0.8170 | 0.7549 | 0.9332 | 0.9041 | 0.9260 | 0.8295 | | RP235 | LL_S_DS | 3.8347 | 2.0001 | 1.2554 | 1.0903 | 1.9033 | 1.6752 | | RP237 | LL_S_DS | 0.5569 | 0.7702 | 0.7385 | 0.9814 | 0.6544 | 0.6795 | | RP238 | LL_S_DS | 3.8347 | 2.0001 | 1.2554 | 1.0903 | 1.9033 | 1.6752 | | **Study designation** | **Condition** | **DTF_DSI** | **PH_DSI** | **BMDW_DSI** | **GY_DSI** | **TGW_DSI** | **SPKFT_DSI** | | RP239 | LL_S_DS | 3.8347 | 2.0001 | 1.2554 | 1.0903 | 1.9033 | 1.6752 | | RP240 | LL_S_DS | 0.2118 | 0.9240 | 0.9360 | 0.9693 | 0.1951 | 0.7135 | | RP241 | LL_S_DS | NA | 1.0187 | 0.9602 | 1.0582 | 1.9033 | 1.1119 | | RP242 | LL_S_DS | -0.2784 | 0.7969 | 0.8883 | 0.9616 | 0.7696 | 0.9771 | | RP243 | LL_S_DS | 3.8347 | 2.0001 | 1.2554 | 1.0903 | 1.9033 | 1.6752 | | RP244 | LL_S_DS | 3.8347 | 2.0001 | 1.2554 | 1.0903 | NA | 1.6752 | | RP245 | LL_S_DS | 3.8347 | 2.0001 | 1.2554 | 1.0903 | 1.9033 | 1.6752 | | RP247 | LL_S_DS | 3.8347 | 2.0001 | 1.2554 | 1.0903 | 1.9033 | 1.6752 | | RP248 | LL_S_DS | 0.6915 | 0.9065 | 0.9866 | 0.8937 | NA | 0.8153 | | RP250 | LL_S_DS | 3.8347 | 2.0001 | 1.2554 | 1.0903 | 1.9033 | 1.6752 | | RP251 | LL_S_DS | 0.0187 | 0.3881 | 0.8963 | 1.0382 | -0.5945 | 0.7497 | | RP252 | LL_S_DS | 3.8347 | 2.0001 | 1.2554 | 1.0903 | NA | 1.6752 | | RP253 | LL_S_DS | NA | 0.8267 | 0.9954 | 1.0132 | 0.8577 | 0.5353 | | RP254 | LL_S_DS | -0.5250 | 0.9632 | 0.9544 | 1.0250 | 1.9033 | 0.8367 | | RP255 | LL_S_DS | -0.2944 | 0.9714 | 0.9460 | 1.0525 | 0.6007 | 1.0391 | | RP256 | LL_S_DS | 0.3741 | 0.6625 | 0.8877 | 1.0370 | 0.6309 | 1.0874 | | RP257 | LL_S_DS | NA | 0.7636 | 0.9494 | 0.9627 | 0.3466 | 1.0855 | | RP258 | LL_S_DS | -0.5513 | 0.7399 | 1.0066 | 1.0322 | 0.7801 | 1.0512 | | RP259 | LL_S_DS | 3.8347 | 2.0001 | 1.2554 | 1.0903 | 1.9033 | 1.6752 | | RP260 | LL_S_DS | -0.1547 | 1.0750 | 0.9676 | 1.0312 | 0.7576 | 0.9958 | | RP261 | LL_S_DS | 3.8347 | 2.0001 | 1.2554 | 1.0903 | 1.9033 | 1.6752 | | RP262 | LL_S_DS | 3.8347 | 2.0001 | 1.2554 | 1.0903 | NA | 1.6752 | | RP263 | LL_S_DS | 0.6141 | 0.6812 | 0.9603 | 0.7710 | 0.7183 | 0.5055 | | RP264 | LL_S_DS | NA | 0.7609 | 0.9673 | 1.0466 | 0.4798 | 0.8535 | | RP266 | LL_S_DS | 3.8347 | 2.0001 | 1.2554 | 1.0903 | 1.9033 | 1.6752 | | RP267 | LL_S_DS | -0.2327 | 0.8047 | 1.0081 | 0.9633 | 0.8391 | 1.0980 | | RP268 | LL_S_DS | NA | 0.8359 | 0.8743 | 1.0667 | 0.5355 | 1.1595 | | RP269 | LL_S_DS | NA | 0.5674 | 0.9854 | 0.9707 | 1.9033 | 1.1509 | | **Study designation** | **Condition** | **DTF_DSI** | **PH_DSI** | **BMDW_DSI** | **GY_DSI** | **TGW_DSI** | **SPKFT_DSI** | | RP270 | LL_S_DS | 0.7850 | 0.5001 | 0.7704 | 0.6239 | 0.4975 | 0.5945 | | RP272 | LL_S_DS | -0.3736 | 0.8962 | 0.9755 | 1.0580 | 0.8391 | 1.0128 | | RP273 | LL_S_DS | 0.3769 | 0.8246 | 0.9415 | 0.9547 | 0.3001 | 0.6448 | | RP274 | LL_S_DS | -0.1116 | 0.7836 | 1.0043 | 0.9754 | 0.6055 | 0.7906 | | RP275 | LL_S_DS | 0.1406 | 0.7999 | 0.7387 | 1.0071 | 0.8335 | 0.7231 | | RP276 | LL_S_DS | 3.8347 | 2.0001 | 1.2554 | 1.0903 | 1.9033 | 1.6752 | | RP277 | LL_S_DS | 3.8347 | 2.0001 | 1.2554 | 1.0903 | 1.9033 | 1.6752 | | RP278 | LL_S_DS | NA | 1.0961 | 0.9421 | 0.9892 | NA | 1.0391 | | RP279 | LL_S_DS | -0.3561 | 1.0191 | 0.9990 | 1.0367 | -0.0546 | 1.2817 | | RP280 | LL_S_DS | -0.4326 | 0.8985 | 0.9939 | 1.0684 | 1.0004 | 1.1803 | | RP281 | LL_S_DS | 0.1124 | 0.0662 | 0.8869 | 0.9842 | 0.6270 | 0.7355 | | RP282 | LL_S_DS | 0.9041 | 0.7115 | 1.0204 | 0.7292 | 0.6947 | 0.5183 | | RP283 | LL_S_DS | NA | 1.1225 | 0.9784 | 1.0574 | 0.6314 | 0.7386 | | RP284 | LL_S_DS | -0.5087 | 0.8722 | 0.8138 | 0.9996 | 0.7696 | 0.6637 | | RP285 | LL_S_DS | 0.4890 | 0.4689 | 0.9714 | 0.9061 | NA | 0.6664 | | RP286 | LL_S_DS | NA | 0.6381 | 0.9451 | 1.0380 | 1.9033 | 1.0981 | | RP287 | LL_S_DS | NA | 0.5350 | 0.9665 | 1.0078 | 1.9033 | 1.0868 | | RP288 | LL_S_DS | 0.6739 | 0.8648 | 0.9191 | 0.9283 | 0.3288 | 0.7329 | | RP289 | LL_S_DS | 0.4988 | 0.6505 | 0.8568 | 0.8296 | 0.4116 | 0.4949 | | RP290 | LL_S_DS | -0.6396 | 0.8564 | 0.9293 | 1.0335 | 0.7801 | 0.8831 | | RP291 | LL_S_DS | 0.2591 | 0.8307 | 0.9922 | 1.0545 | 0.5134 | 1.0794 | | RP292 | LL_S_DS | -0.1479 | 0.5317 | 1.0155 | 1.0335 | 0.6870 | 1.0334 | | RP293 | LL_S_DS | NA | 0.8708 | 0.8930 | 1.0025 | 1.9033 | NA | | RP294 | LL_S_DS | 1.1912 | 0.5532 | 0.8457 | 0.6541 | 0.3775 | 0.5744 | | RP295 | LL_S_DS | 1.0478 | 0.3328 | 0.7912 | 0.4954 | 0.1589 | 0.4671 | | RP296 | LL_S_DS | 0.3964 | 0.7093 | 0.6687 | 1.0376 | 0.9950 | 0.8570 | | RP297 | LL_S_DS | NA | 0.6932 | 0.9151 | 1.0040 | 0.5662 | 1.1061 | | RP298 | LL_S_DS | 1.0419 | 0.8189 | 0.9685 | 0.9241 | 0.8135 | 0.5759 | | **Study designation** | **Condition** | **DTF_DSI** | **PH_DSI** | **BMDW_DSI** | **GY_DSI** | **TGW_DSI** | **SPKFT_DSI** | | RP299 | LL_S_DS | 0.5649 | 0.6461 | 1.0274 | 0.8009 | NA | 0.5947 | | RP300 | LL_S_DS | NA | 0.7344 | 0.8789 | 1.0599 | 1.0307 | 0.9008 | | RP002 | UL_S_DS | -0.6256 | 0.9974 | 0.9425 | 1.1779 | 1.0423 | 1.1255 | | RP003 | UL_S_DS | -1.0402 | 0.6575 | 0.8795 | 1.1012 | 0.8203 | 0.9728 | | RP004 | UL_S_DS | NA | 1.1479 | 0.8237 | 1.2386 | 0.9024 | NA | | RP005 | UL_S_DS | -0.4806 | 0.7299 | 0.7926 | 1.1553 | 0.9167 | 1.2135 | | RP006 | UL_S_DS | NA | 1.1323 | 0.8319 | 1.2386 | 0.9718 | NA | | RP007 | UL_S_DS | -0.9297 | 1.1057 | 0.8693 | 1.1425 | 0.7735 | 1.1669 | | RP008 | UL_S_DS | -0.2058 | 0.5988 | 1.0908 | 1.1053 | 1.0035 | 1.2304 | | RP009 | UL_S_DS | -0.0611 | 0.6041 | 0.9583 | 1.1618 | 0.4734 | 1.2567 | | RP010 | UL_S_DS | -0.7100 | 0.8627 | 1.0253 | 1.1664 | NA | 1.2459 | | RP011 | UL_S_DS | 0.4226 | 0.6604 | 0.6311 | 1.1773 | 0.5968 | 0.7886 | | RP012 | UL_S_DS | 0.5891 | 0.8151 | 0.7566 | 0.9467 | NA | 1.0186 | | RP013 | UL_S_DS | -0.8782 | 0.8276 | 0.8320 | 1.1680 | 0.8778 | 1.1569 | | RP014 | UL_S_DS | -1.2861 | 0.8675 | 0.7373 | 1.1813 | 1.2599 | 0.9069 | | RP015 | UL_S_DS | 0.0974 | 0.8564 | 1.0381 | 1.1832 | 0.5481 | 1.1287 | | RP018 | UL_S_DS | 4.9726 | 2.0739 | 1.4415 | 1.2386 | 1.8048 | 1.3527 | | RP019 | UL_S_DS | NA | 0.5868 | 0.8924 | 1.1213 | 0.8516 | 1.1491 | | RP020 | UL_S_DS | 4.9726 | 2.0739 | 1.4415 | 1.2386 | 1.8048 | 1.3527 | | RP021 | UL_S_DS | 4.9726 | 2.0739 | 1.4415 | 1.2386 | 1.8048 | 1.3527 | | RP022 | UL_S_DS | -1.7217 | 0.9661 | 0.6454 | 1.0883 | 0.7219 | 1.2222 | | RP023 | UL_S_DS | 0.4476 | 0.8586 | 1.0452 | 1.1664 | 0.7645 | 1.1828 | | RP024 | UL_S_DS | -1.0693 | 1.0088 | 0.9010 | 1.1873 | 1.3620 | 1.2286 | | RP025 | UL_S_DS | NA | 0.6620 | 0.9142 | 1.1668 | 0.7219 | NA | | RP026 | UL_S_DS | 0.5726 | 0.7177 | 0.8130 | 1.1349 | 0.7190 | 0.8942 | | RP028 | UL_S_DS | 4.9726 | 2.0739 | 1.4415 | 1.2386 | 1.8048 | 1.3527 | | RP029 | UL_S_DS | 4.9726 | 2.0739 | 1.4415 | 1.2386 | 1.8048 | 1.3527 | | RP030 | UL_S_DS | -0.9338 | 0.8254 | 0.8964 | 1.1660 | 1.1628 | 1.2282 | | **Study designation** | **Condition** | **DTF_DSI** | **PH_DSI** | **BMDW_DSI** | **GY_DSI** | **TGW_DSI** | **SPKFT_DSI** | | RP031 | UL_S_DS | 0.1738 | 0.7765 | 0.8114 | 1.0736 | -2.8847 | 0.7091 | | RP032 | UL_S_DS | 0.5144 | 0.7871 | 1.0279 | 1.1089 | 1.1920 | 1.1732 | | RP033 | UL_S_DS | 1.2755 | 0.3549 | 0.8483 | 0.2414 | 0.5726 | 0.7363 | | RP034 | UL_S_DS | 1.4228 | 0.4648 | 0.8304 | -0.8913 | 0.5190 | 0.1159 | | RP036 | UL_S_DS | 0.7384 | 0.6968 | 0.8694 | 0.6691 | 0.6324 | 0.1820 | | RP037 | UL_S_DS | 1.0892 | 0.4292 | 0.7607 | -0.4875 | 0.3921 | 0.1074 | | RP038 | UL_S_DS | 0.4022 | 0.6411 | 0.7695 | 0.5659 | NA | 0.1877 | | RP039 | UL_S_DS | 1.3363 | 0.4826 | 0.8192 | -0.5349 | 0.2141 | 0.0810 | | RP040 | UL_S_DS | 1.1270 | 0.3495 | 0.8676 | 0.4882 | 0.6125 | 0.0781 | | RP041 | UL_S_DS | -0.2333 | 0.4800 | 0.7354 | 0.5107 | NA | 0.6852 | | RP042 | UL_S_DS | 1.2844 | 0.4154 | 0.6564 | 0.4212 | 0.7372 | 0.2388 | | RP043 | UL_S_DS | -1.0402 | 0.8131 | 0.8802 | 1.1228 | 0.8631 | 1.2209 | | RP044 | UL_S_DS | 0.0736 | 0.5354 | 0.7773 | 1.1134 | 1.0095 | 1.0052 | | RP045 | UL_S_DS | 0.0482 | 0.5080 | 1.0703 | 1.1309 | 0.7320 | 1.1634 | | RP046 | UL_S_DS | -0.3731 | 0.9061 | 0.8392 | 1.1675 | 0.8122 | 1.2149 | | RP047 | UL_S_DS | 1.2339 | 0.7826 | 0.8808 | 0.4318 | 0.5050 | 0.3572 | | RP048 | UL_S_DS | 1.0201 | 0.6327 | 0.9646 | 0.7592 | 0.3833 | 0.4304 | | RP049 | UL_S_DS | 4.9726 | 2.0739 | 1.4415 | 1.2386 | 1.8048 | 1.3527 | | RP050 | UL_S_DS | -1.1803 | 1.0306 | 0.8979 | 1.1996 | 0.5308 | 1.2266 | | RP051 | UL_S_DS | 1.2521 | 0.8035 | 0.7069 | 0.5968 | 0.3854 | 0.2835 | | RP052 | UL_S_DS | 4.9726 | 2.0739 | 1.4415 | 1.2386 | 1.8048 | 1.3527 | | RP053 | UL_S_DS | 0.5566 | 0.5035 | 0.7234 | 0.0225 | 0.5952 | 0.2126 | | RP054 | UL_S_DS | -1.1963 | 0.8828 | 0.7392 | 1.1267 | 0.7735 | 1.2670 | | RP055 | UL_S_DS | NA | 0.6382 | 0.6588 | 1.2120 | 1.2657 | 1.2106 | | RP056 | UL_S_DS | -0.9423 | 0.7189 | 1.0714 | 1.0549 | 0.9718 | 1.2388 | | RP057 | UL_S_DS | 4.9726 | 2.0739 | 1.4415 | 1.2386 | NA | 1.3527 | | RP058 | UL_S_DS | -0.9061 | 0.7117 | 0.8766 | 1.1600 | 1.1860 | 1.2641 | | RP059 | UL_S_DS | 0.4394 | 0.4541 | 0.9889 | 0.7998 | 0.5455 | 0.9886 | | **Study designation** | **Condition** | **DTF_DSI** | **PH_DSI** | **BMDW_DSI** | **GY_DSI** | **TGW_DSI** | **SPKFT_DSI** | | RP060 | UL_S_DS | 0.2737 | 0.7347 | 0.5932 | 1.1357 | 0.9645 | 0.9354 | | RP061 | UL_S_DS | 1.6616 | 0.5173 | 0.7489 | -0.1999 | 0.6513 | 0.2111 | | RP062 | UL_S_DS | -0.3029 | 0.3221 | 0.9540 | 1.1574 | 0.6872 | 1.0257 | | RP063 | UL_S_DS | -1.1055 | 0.8156 | 0.9638 | 1.1409 | 0.9718 | 1.2385 | | RP065 | UL_S_DS | 1.6750 | 0.4864 | 0.8539 | -0.1409 | NA | 0.0875 | | RP066 | UL_S_DS | 4.9726 | 2.0739 | 1.4415 | 1.2386 | 1.8048 | 1.3527 | | RP067 | UL_S_DS | -1.0806 | 0.9312 | 0.7524 | 0.9734 | 0.8390 | 1.0454 | | RP068 | UL_S_DS | -0.5530 | 0.9385 | 0.9402 | 1.1583 | 1.2372 | 1.2137 | | RP070 | UL_S_DS | -1.0947 | 0.8644 | 0.9325 | 1.1623 | 1.3227 | 1.2554 | | RP071 | UL_S_DS | -1.2217 | 0.9235 | 0.9225 | 1.1881 | 0.6649 | 1.2143 | | RP072 | UL_S_DS | NA | 0.5432 | 0.8567 | 1.2299 | 0.7735 | NA | | RP073 | UL_S_DS | 0.4546 | 0.5826 | 0.9237 | 1.1257 | 0.3854 | 1.1338 | | RP074 | UL_S_DS | -0.9311 | 0.6593 | 0.8248 | 1.1653 | 1.3052 | 1.2179 | | RP075 | UL_S_DS | -0.1804 | 0.7393 | 0.7205 | 1.1444 | 0.8203 | 1.2173 | | RP076 | UL_S_DS | -1.0806 | 0.9061 | 0.9193 | 1.1740 | 1.3053 | 1.2184 | | RP077 | UL_S_DS | 4.9726 | 2.0739 | 1.4415 | 1.2386 | 1.8048 | 1.3527 | | RP079 | UL_S_DS | 0.2521 | 0.6442 | 0.8485 | 1.1535 | 1.2333 | 1.2155 | | RP080 | UL_S_DS | 0.0159 | 0.6212 | 0.7900 | 1.0711 | 1.0469 | 0.9617 | | RP081 | UL_S_DS | -0.8563 | 0.3643 | 0.8102 | 1.1118 | 0.6649 | 1.2060 | | RP082 | UL_S_DS | 4.9726 | 2.0739 | 1.4415 | 1.2386 | 1.8048 | 1.3527 | | RP083 | UL_S_DS | NA | 0.7487 | 0.9074 | 1.1145 | 0.1902 | 1.2240 | | RP084 | UL_S_DS | -1.3303 | 0.8986 | 1.0542 | 1.1720 | 1.3901 | 1.1102 | | RP085 | UL_S_DS | -1.3298 | 0.5951 | 0.8731 | 1.1190 | 0.4512 | 1.1312 | | RP086 | UL_S_DS | 1.6613 | 0.7685 | 0.8600 | 0.6073 | 0.6817 | 0.1716 | | RP087 | UL_S_DS | -0.0683 | 0.2837 | 0.7889 | 1.1047 | 0.9175 | 1.0514 | | RP088 | UL_S_DS | 0.2843 | 0.4882 | 0.8649 | 1.1164 | 1.0228 | 1.2388 | | RP089 | UL_S_DS | -1.0925 | 1.1403 | 0.8896 | 1.2166 | 0.9385 | 1.1978 | | RP090 | UL_S_DS | 0.6016 | 0.7116 | 0.8451 | 1.0652 | 0.8261 | 0.6419 | | **Study designation** | **Condition** | **DTF_DSI** | **PH_DSI** | **BMDW_DSI** | **GY_DSI** | **TGW_DSI** | **SPKFT_DSI** | | RP091 | UL_S_DS | 0.3150 | 1.0768 | 0.6530 | 1.1090 | 0.7529 | 0.9551 | | RP092 | UL_S_DS | -1.0784 | 1.0734 | 0.8069 | 1.1463 | 0.8631 | 1.2193 | | RP093 | UL_S_DS | NA | 0.9429 | 0.8774 | 1.1399 | 1.3760 | 1.2235 | | RP094 | UL_S_DS | -0.6652 | 0.7863 | 0.9499 | 1.1700 | 1.1251 | 1.2550 | | RP095 | UL_S_DS | 1.2188 | 0.5096 | 0.8517 | 0.6316 | 0.6531 | 0.2131 | | RP096 | UL_S_DS | -0.8684 | 0.9136 | 0.9503 | 1.1854 | 1.0968 | 1.2176 | | RP097 | UL_S_DS | 0.6864 | 0.3509 | 0.9200 | 0.9473 | 0.6044 | 0.5880 | | RP098 | UL_S_DS | 0.9191 | 0.3871 | 0.7642 | 0.4197 | NA | 1.0114 | | RP099 | UL_S_DS | -1.6790 | 0.8997 | 0.8215 | 1.1247 | 0.5422 | 1.2380 | | RP100 | UL_S_DS | 0.0473 | 0.7059 | 0.8312 | 0.9732 | NA | 0.5437 | | RP101 | UL_S_DS | -1.2038 | 0.8142 | 0.9502 | 1.1320 | 1.0829 | 1.2188 | | RP102 | UL_S_DS | -0.8401 | 1.0350 | 0.8414 | 1.1286 | 0.7735 | 1.2305 | | RP103 | UL_S_DS | -0.0153 | 0.8022 | 1.0931 | 0.6672 | 0.7872 | 0.8970 | | RP104 | UL_S_DS | 0.2632 | 0.7553 | 1.0091 | 0.5753 | 1.1386 | 0.6346 | | RP105 | UL_S_DS | 4.9726 | 2.0739 | 1.4415 | 1.2386 | 1.8048 | 1.3527 | | RP106 | UL_S_DS | 0.2768 | 0.9004 | 0.7698 | 1.1901 | NA | 1.0590 | | RP107 | UL_S_DS | 0.3862 | 0.8711 | 0.7026 | 0.7443 | 0.9565 | 0.8128 | | RP108 | UL_S_DS | -1.5265 | 0.6170 | 0.7804 | 1.1529 | 0.7735 | 1.2148 | | RP109 | UL_S_DS | 1.3798 | 0.4692 | 0.7493 | 0.4358 | 0.3662 | 0.6601 | | RP110 | UL_S_DS | 4.9726 | 2.0739 | 1.4415 | 1.2386 | NA | 1.3527 | | RP111 | UL_S_DS | NA | 0.9818 | 0.6346 | 1.2386 | 0.9718 | NA | | RP112 | UL_S_DS | -0.1117 | 0.7758 | 0.7726 | 1.1183 | 0.8908 | 1.0159 | | RP113 | UL_S_DS | 0.5565 | 0.2987 | 0.6266 | 0.8183 | 0.7836 | 0.8765 | | RP114 | UL_S_DS | 0.9028 | 0.5897 | 0.9815 | 0.1806 | 0.4308 | 0.4250 | | RP115 | UL_S_DS | 4.9726 | 2.0739 | 1.4415 | 1.2386 | 1.8048 | 1.3527 | | RP116 | UL_S_DS | -0.8812 | 0.9028 | 0.9604 | 1.1518 | 0.7735 | 1.2162 | | RP117 | UL_S_DS | -0.9911 | 0.8601 | 0.8829 | 1.0743 | 0.5308 | 1.2187 | | RP118 | UL_S_DS | 4.9726 | 2.0739 | 1.4415 | 1.2386 | 1.8048 | 1.3527 | | **Study designation** | **Condition** | **DTF_DSI** | **PH_DSI** | **BMDW_DSI** | **GY_DSI** | **TGW_DSI** | **SPKFT_DSI** | | RP119 | UL_S_DS | -1.1803 | 0.8101 | 1.0198 | 1.1386 | 1.3560 | 1.2378 | | RP120 | UL_S_DS | 0.4265 | 0.7086 | 0.9522 | 1.0975 | NA | 0.9777 | | RP121 | UL_S_DS | 4.9726 | 2.0739 | 1.4415 | 1.2386 | 1.8048 | 1.3527 | | RP122 | UL_S_DS | -0.5791 | 0.7526 | 1.0299 | 1.1725 | NA | 1.1042 | | RP123 | UL_S_DS | 4.9726 | 2.0739 | 1.4415 | 1.2386 | 1.8048 | 1.3527 | | RP124 | UL_S_DS | 1.3731 | 0.2597 | 0.7284 | -0.9729 | NA | 0.1867 | | RP125 | UL_S_DS | 1.1766 | 0.8422 | 0.9247 | 0.5573 | 0.5157 | 0.4955 | | RP126 | UL_S_DS | 4.9726 | 2.0739 | 1.4415 | 1.2386 | 1.8048 | 1.3527 | | RP127 | UL_S_DS | 4.9726 | 2.0739 | 1.4415 | 1.2386 | 1.8048 | 1.3527 | | RP128 | UL_S_DS | 1.2785 | 0.6327 | 0.7053 | -0.1944 | 0.6890 | 0.2478 | | RP130 | UL_S_DS | NA | 1.0432 | 0.9528 | 1.1713 | 1.1225 | 1.1284 | | RP131 | UL_S_DS | -1.2566 | 1.0131 | 0.6592 | 1.2013 | 0.9024 | 1.2688 | | RP132 | UL_S_DS | 1.2072 | 0.5423 | 1.0607 | 0.4759 | 0.3954 | 0.3699 | | RP133 | UL_S_DS | -0.6454 | 0.8805 | 0.7720 | 1.1708 | 0.8318 | 1.2018 | | RP134 | UL_S_DS | -0.8153 | 0.6996 | 0.9964 | 1.1664 | 0.8203 | 1.2321 | | RP135 | UL_S_DS | -0.1336 | 0.6974 | 1.0820 | 1.1622 | 1.1240 | 0.8031 | | RP136 | UL_S_DS | -0.4326 | 0.9734 | 0.8993 | 1.1513 | NA | 1.2348 | | RP137 | UL_S_DS | 0.5137 | 0.3751 | 0.9001 | 0.9450 | 0.7798 | 0.7457 | | RP138 | UL_S_DS | -1.0806 | 0.5588 | 0.9592 | 1.1168 | 0.5818 | 1.2168 | | RP139 | UL_S_DS | 0.0736 | 0.3145 | 0.7467 | 0.9375 | 0.6115 | 0.7391 | | RP140 | UL_S_DS | -1.2612 | 0.7338 | 0.8471 | 1.0211 | 0.6016 | 1.2059 | | RP141 | UL_S_DS | -1.0402 | 0.7563 | 0.9596 | 1.1093 | 1.3822 | 1.2152 | | RP142 | UL_S_DS | 0.2224 | 0.5993 | 0.6452 | 0.7922 | NA | 0.5158 | | RP143 | UL_S_DS | -0.9931 | 0.7677 | 1.0782 | 1.0723 | 1.3016 | 1.2132 | | RP144 | UL_S_DS | 0.5211 | 0.8360 | 0.8477 | 0.8388 | 0.4355 | 0.6699 | | RP146 | UL_S_DS | -0.9255 | 0.7418 | 1.0972 | 1.0545 | NA | 1.2150 | | RP147 | UL_S_DS | -0.5359 | 0.7122 | 0.7705 | 1.1437 | 1.2377 | 1.2141 | | RP148 | UL_S_DS | -0.9255 | 0.9465 | 0.8310 | 1.1463 | 0.3463 | 1.2235 | | **Study designation** | **Condition** | **DTF_DSI** | **PH_DSI** | **BMDW_DSI** | **GY_DSI** | **TGW_DSI** | **SPKFT_DSI** | | RP149 | UL_S_DS | -0.8872 | 0.7121 | 0.9179 | 1.1245 | 1.2032 | 1.2093 | | RP150 | UL_S_DS | -0.4344 | 0.8721 | 0.7833 | 1.0988 | 1.0677 | 1.1518 | | RP151 | UL_S_DS | 0.0293 | 0.5368 | 0.8035 | 0.8168 | 0.6262 | 0.8906 | | RP152 | UL_S_DS | 0.0168 | 0.1680 | 0.9353 | 0.8938 | 0.5028 | 0.8847 | | RP153 | UL_S_DS | 1.3617 | 0.6502 | 0.8508 | 0.7455 | 0.6753 | 0.3554 | | RP154 | UL_S_DS | -0.4573 | 0.5263 | 0.9521 | 0.9842 | 0.3140 | 0.8523 | | RP155 | UL_S_DS | 0.9131 | 0.7185 | 0.9224 | 1.1697 | 1.4433 | 1.2337 | | RP156 | UL_S_DS | 0.9191 | 0.5115 | 0.7196 | 0.5534 | 0.5700 | 0.4379 | | RP157 | UL_S_DS | -0.4806 | 0.7284 | 0.7825 | 1.0604 | 0.9851 | 1.1255 | | RP158 | UL_S_DS | 4.9726 | 2.0739 | 1.4415 | 1.2386 | 1.8048 | 1.3527 | | RP159 | UL_S_DS | 1.3528 | 0.4941 | 0.6880 | -0.3484 | 0.6937 | 0.0565 | | RP160 | UL_S_DS | 0.1694 | 0.5851 | 0.6814 | 1.1176 | 0.7713 | 0.2294 | | RP161 | UL_S_DS | 0.9621 | 0.5942 | 0.9228 | -0.1988 | 0.5191 | 0.1981 | | RP162 | UL_S_DS | 4.9726 | 2.0739 | 1.4415 | 1.2386 | 1.8048 | 1.3527 | | RP164 | UL_S_DS | 4.9726 | 2.0739 | 1.4415 | 1.2386 | 1.8048 | 1.3527 | | RP165 | UL_S_DS | 2.0443 | 0.5623 | 0.7230 | -2.9008 | 0.4470 | 0.0545 | | RP166 | UL_S_DS | -1.1325 | 0.9856 | 0.9358 | 1.0680 | 1.1102 | 1.2192 | | RP167 | UL_S_DS | 4.9726 | 2.0739 | 1.4415 | 1.2386 | 1.8048 | 1.3527 | | RP168 | UL_S_DS | 0.9160 | 0.6299 | 0.7218 | -1.0750 | NA | 0.1455 | | RP169 | UL_S_DS | 4.9726 | 2.0739 | 1.4415 | 1.2386 | 1.8048 | 1.3527 | | RP170 | UL_S_DS | -1.0157 | 0.3062 | 0.9345 | 1.1743 | 1.3992 | 1.2373 | | RP171 | UL_S_DS | 4.9726 | 2.0739 | 1.4415 | 1.2386 | 1.8048 | 1.3527 | | RP172 | UL_S_DS | -0.2003 | 0.7569 | 0.7926 | 0.9585 | 0.7489 | 0.6632 | | RP173 | UL_S_DS | 4.9726 | 2.0739 | 1.4415 | 1.2386 | 1.8048 | 1.3527 | | RP174 | UL_S_DS | 4.9726 | 2.0739 | 1.4415 | 1.2386 | 1.8048 | 1.3527 | | RP175 | UL_S_DS | -0.0727 | 0.0417 | 0.9545 | 1.1373 | 0.7182 | 0.8635 | | RP176 | UL_S_DS | 4.9726 | 2.0739 | 1.4415 | 1.2386 | NA | 1.3527 | | RP177 | UL_S_DS | 4.9726 | 2.0739 | 1.4415 | 1.2386 | 1.8048 | 1.3527 | | **Study designation** | **Condition** | **DTF_DSI** | **PH_DSI** | **BMDW_DSI** | **GY_DSI** | **TGW_DSI** | **SPKFT_DSI** | | RP178 | UL_S_DS | 1.9796 | 0.5436 | 0.7512 | -0.6872 | 0.3877 | 0.0794 | | RP179 | UL_S_DS | -1.1554 | 1.0410 | 1.1243 | 1.1737 | 1.0580 | 1.2297 | | RP180 | UL_S_DS | 0.2371 | 0.8828 | 0.9218 | 1.1218 | 0.8859 | 0.6290 | | RP181 | UL_S_DS | 4.9726 | 2.0739 | 1.4415 | 1.2386 | 1.8048 | 1.3527 | | RP182 | UL_S_DS | 0.6345 | 0.7427 | 0.9862 | 0.8231 | 0.7031 | 0.2921 | | RP183 | UL_S_DS | NA | 0.7187 | 0.7916 | 1.2055 | 1.3238 | 1.2897 | | RP184 | UL_S_DS | -1.4260 | 0.9417 | 1.0045 | 1.2029 | 0.7098 | 1.2330 | | RP185 | UL_S_DS | 4.9726 | 2.0739 | 1.4415 | 1.2386 | NA | 1.3527 | | RP186 | UL_S_DS | 1.3313 | 0.3929 | 0.9312 | -0.1619 | -0.0618 | 0.1951 | | RP188 | UL_S_DS | 4.9726 | 2.0739 | 1.4415 | 1.2386 | 1.8048 | 1.3527 | | RP189 | UL_S_DS | NA | 1.0805 | 0.7720 | 1.2137 | 0.7735 | 1.1718 | | RP190 | UL_S_DS | NA | 0.8676 | 1.0902 | 1.0256 | 0.8631 | NA | | RP191 | UL_S_DS | 0.5137 | 0.6374 | 0.9705 | 1.0411 | NA | 0.8475 | | RP192 | UL_S_DS | 4.9726 | 2.0739 | 1.4415 | 1.2386 | 1.8048 | 1.3527 | | RP193 | UL_S_DS | 1.2785 | 0.4307 | 0.5296 | 0.3750 | 0.5585 | 0.4896 | | RP194 | UL_S_DS | -0.2001 | 0.7900 | 0.8671 | 1.1447 | 0.7826 | 1.1126 | | RP196 | UL_S_DS | 1.4781 | 0.8657 | 0.9654 | 1.0728 | 0.8631 | NA | | RP197 | UL_S_DS | 4.9726 | 2.0739 | 1.4415 | 1.2386 | 1.8048 | 1.3527 | | RP198 | UL_S_DS | 1.7823 | 0.4164 | 0.7013 | -0.1970 | 0.5648 | 0.1548 | | RP199 | UL_S_DS | 4.9726 | 2.0739 | 1.4415 | 1.2386 | NA | 1.3527 | | RP200 | UL_S_DS | 4.9726 | 2.0739 | 1.4415 | 1.2386 | 1.8048 | 1.3527 | | RP201 | UL_S_DS | -1.3150 | 0.7838 | 0.9261 | 1.1927 | 0.9718 | 1.2236 | | RP202 | UL_S_DS | -0.1211 | 0.5524 | 0.8601 | 1.1529 | 0.4002 | 1.1635 | | RP203 | UL_S_DS | 0.2796 | 0.6576 | 0.8324 | 1.1624 | 0.3575 | 0.8858 | | RP204 | UL_S_DS | 4.9726 | 2.0739 | 1.4415 | 1.2386 | 1.8048 | 1.3527 | | RP205 | UL_S_DS | NA | 0.9108 | 0.9097 | 1.2159 | 0.9024 | 1.1996 | | RP206 | UL_S_DS | 4.9726 | 2.0739 | 1.4415 | 1.2386 | 1.8048 | 1.3527 | | RP207 | UL_S_DS | -1.2217 | 0.5647 | 0.7835 | 1.1664 | 0.3609 | 1.2111 | | **Study designation** | **Condition** | **DTF_DSI** | **PH_DSI** | **BMDW_DSI** | **GY_DSI** | **TGW_DSI** | **SPKFT_DSI** | | RP208 | UL_S_DS | -0.2152 | 0.6087 | 0.8615 | 1.1482 | 0.8590 | 1.0329 | | RP209 | UL_S_DS | 0.0433 | 0.6679 | 0.9384 | 1.0035 | 1.1158 | 1.2205 | | RP210 | UL_S_DS | -0.4003 | 0.7038 | 1.0301 | 1.0914 | 0.6824 | 0.9467 | | RP211 | UL_S_DS | NA | 0.8285 | 1.0292 | 1.2275 | 1.0580 | 1.2323 | | RP212 | UL_S_DS | -0.9555 | 0.8986 | 0.7549 | 1.2051 | 0.9385 | 1.2126 | | RP213 | UL_S_DS | 4.9726 | 2.0739 | 1.4415 | 1.2386 | NA | 1.3527 | | RP214 | UL_S_DS | 4.9726 | 2.0739 | 1.4415 | 1.2386 | 1.8048 | 1.3527 | | RP215 | UL_S_DS | NA | 0.9654 | 0.9460 | 1.1674 | 0.7636 | 1.2035 | | RP216 | UL_S_DS | 0.5049 | 0.6503 | 0.8368 | 0.7405 | 0.6246 | 0.6746 | | RP217 | UL_S_DS | 4.9726 | 2.0739 | 1.4415 | 1.2386 | 1.8048 | 1.3527 | | RP218 | UL_S_DS | 4.9726 | 2.0739 | 1.4415 | 1.2386 | 1.8048 | 1.3527 | | RP219 | UL_S_DS | -1.0415 | 0.9367 | 1.0930 | 1.1578 | 0.1245 | 1.2220 | | RP220 | UL_S_DS | -1.1403 | 1.0151 | 0.8724 | 1.2063 | 0.7735 | 1.1995 | | RP222 | UL_S_DS | -0.5943 | 0.6164 | 0.8601 | 1.1670 | 0.7728 | 1.0270 | | RP223 | UL_S_DS | NA | 1.0685 | 0.8984 | 1.1452 | 0.4255 | 1.2240 | | RP224 | UL_S_DS | 0.3745 | 0.5313 | 0.7129 | 0.9358 | 0.6204 | 0.4276 | | RP225 | UL_S_DS | NA | 0.5965 | 0.9400 | 1.1916 | 0.9718 | NA | | RP226 | UL_S_DS | 1.5670 | 0.1146 | 0.7230 | 0.1025 | 0.3303 | 0.1397 | | RP228 | UL_S_DS | 0.1383 | -0.0746 | 0.6789 | 1.0213 | 0.4816 | 1.0279 | | RP229 | UL_S_DS | -1.1304 | 1.0460 | 0.7339 | 1.1981 | 0.7735 | 1.2183 | | RP230 | UL_S_DS | 4.9726 | 2.0739 | 1.4415 | 1.2386 | 1.8048 | 1.3527 | | RP231 | UL_S_DS | 0.0228 | 0.6599 | 1.1153 | 1.1748 | 0.5248 | 1.1481 | | RP232 | UL_S_DS | 0.9721 | 0.4586 | 0.9839 | 0.9252 | 0.5470 | 0.0898 | | RP233 | UL_S_DS | 1.0689 | 0.5546 | 0.7412 | 0.6045 | 0.3088 | 0.1036 | | RP234 | UL_S_DS | 0.9827 | 0.7267 | 0.8230 | 0.8633 | 0.6183 | 0.3640 | | RP235 | UL_S_DS | 4.9726 | 2.0739 | 1.4415 | 1.2386 | 1.8048 | 1.3527 | | RP237 | UL_S_DS | -0.0934 | 0.6414 | 0.6243 | 0.6468 | 0.4318 | 0.3847 | | RP238 | UL_S_DS | 4.9726 | 2.0739 | 1.4415 | 1.2386 | 1.8048 | 1.3527 | | **Study designation** | **Condition** | **DTF_DSI** | **PH_DSI** | **BMDW_DSI** | **GY_DSI** | **TGW_DSI** | **SPKFT_DSI** | | RP239 | UL_S_DS | NA | 0.8808 | 0.7814 | 1.1393 | 1.4218 | 1.1362 | | RP240 | UL_S_DS | -0.4525 | 0.8159 | 0.9955 | 1.0851 | 0.8361 | 1.2104 | | RP241 | UL_S_DS | 0.1586 | 0.8503 | 0.8422 | 1.1483 | 0.7142 | 1.2418 | | RP242 | UL_S_DS | -1.0439 | 0.7665 | 0.8821 | 1.1747 | 1.3132 | 1.1559 | | RP243 | UL_S_DS | -0.9033 | 0.9435 | 0.9513 | 1.1832 | 1.2492 | 1.2393 | | RP244 | UL_S_DS | 4.9726 | 2.0739 | 1.4415 | 1.2386 | NA | 1.3527 | | RP245 | UL_S_DS | 4.9726 | 2.0739 | 1.4415 | 1.2386 | 1.8048 | 1.3527 | | RP247 | UL_S_DS | NA | 0.9749 | 1.0643 | 1.1987 | 0.9385 | 1.2120 | | RP248 | UL_S_DS | 0.8330 | 0.7596 | 0.8475 | 0.7857 | NA | 0.6953 | | RP250 | UL_S_DS | 4.9726 | 2.0739 | 1.4415 | 1.2386 | 1.8048 | 1.3527 | | RP251 | UL_S_DS | 4.9726 | 2.0739 | 1.4415 | 1.2386 | 1.8048 | 1.3527 | | RP252 | UL_S_DS | -1.2551 | 0.8510 | 1.0262 | 1.1682 | NA | 1.2213 | | RP253 | UL_S_DS | 0.1090 | 0.7313 | 0.9062 | 1.0913 | 1.3717 | 1.2569 | | RP254 | UL_S_DS | -0.9033 | 0.8490 | 0.7434 | 1.1060 | 0.7219 | 1.2259 | | RP255 | UL_S_DS | -0.2915 | 0.9041 | 0.8124 | 1.1904 | 0.3609 | 1.2587 | | RP256 | UL_S_DS | 4.9726 | 2.0739 | 1.4415 | 1.2386 | 1.8048 | 1.3527 | | RP257 | UL_S_DS | -1.0157 | 0.7104 | 0.8488 | 1.0678 | 0.4512 | 1.2217 | | RP258 | UL_S_DS | NA | 0.7005 | 0.9848 | 1.1125 | 1.2162 | 1.1129 | | RP259 | UL_S_DS | -0.0472 | 0.8940 | 0.7652 | 1.1472 | 1.1605 | 0.8889 | | RP260 | UL_S_DS | 0.1724 | 0.8453 | 0.8584 | 0.9778 | 0.2194 | 0.8116 | | RP261 | UL_S_DS | 4.9726 | 2.0739 | 1.4415 | 1.2386 | 1.8048 | 1.3527 | | RP262 | UL_S_DS | 4.9726 | 2.0739 | 1.4415 | 1.2386 | NA | 1.3527 | | RP263 | UL_S_DS | 1.5213 | 0.4670 | 0.9012 | 0.3238 | 0.4416 | 0.1946 | | RP264 | UL_S_DS | NA | 0.9743 | 0.8110 | 1.1900 | 0.6016 | 1.2366 | | RP266 | UL_S_DS | 4.9726 | 2.0739 | 1.4415 | 1.2386 | 1.8048 | 1.3527 | | RP267 | UL_S_DS | -1.0784 | 0.7411 | 0.9919 | 1.0579 | 0.9024 | 1.2309 | | RP268 | UL_S_DS | -1.2471 | 0.8707 | 0.8476 | 1.1939 | 1.2972 | 1.2200 | | RP269 | UL_S_DS | -0.9338 | 0.8063 | 0.9412 | 1.0627 | 0.2578 | 1.2410 | | **Study designation** | **Condition** | **DTF_DSI** | **PH_DSI** | **BMDW_DSI** | **GY_DSI** | **TGW_DSI** | **SPKFT_DSI** | | RP270 | UL_S_DS | 1.6218 | 0.3855 | 0.7440 | -0.2694 | 0.5942 | 0.1223 | | RP272 | UL_S_DS | 0.1568 | 0.8370 | 0.9592 | 1.1771 | 1.0684 | 1.0686 | | RP273 | UL_S_DS | 0.9417 | 0.7956 | 0.8936 | 0.2678 | 0.3828 | 0.1235 | | RP274 | UL_S_DS | 0.8032 | 0.6301 | 0.9105 | 0.9854 | 0.3478 | 0.9226 | | RP275 | UL_S_DS | -1.3192 | 0.8805 | 0.7031 | 1.1739 | 1.3561 | 1.2210 | | RP276 | UL_S_DS | -0.3529 | 0.9019 | 0.8388 | 1.1378 | 1.0257 | 0.9765 | | RP277 | UL_S_DS | -0.1809 | 1.0559 | 0.9919 | 1.1138 | 1.3044 | 1.2223 | | RP278 | UL_S_DS | 0.5908 | 0.7691 | 0.8867 | 0.7089 | NA | 0.7334 | | RP279 | UL_S_DS | 0.4726 | 0.5980 | 0.7346 | 0.7956 | 0.4662 | 0.5168 | | RP280 | UL_S_DS | -0.3080 | 0.9904 | 1.0494 | 1.1963 | 1.1061 | 1.2253 | | RP281 | UL_S_DS | -1.1455 | 0.6241 | 0.9251 | 1.1258 | 1.3277 | 1.2244 | | RP282 | UL_S_DS | 0.2828 | 0.7253 | 0.9611 | 1.0688 | 0.7867 | 0.8295 | | RP283 | UL_S_DS | -0.0860 | 0.8092 | 0.8330 | 1.1627 | 0.8743 | 0.8312 | | RP284 | UL_S_DS | -0.8314 | 0.8755 | 0.7986 | 1.1445 | 0.9385 | 1.2341 | | RP285 | UL_S_DS | 0.0650 | 0.4559 | 0.9739 | 1.1487 | NA | 1.0688 | | RP286 | UL_S_DS | -0.6719 | 0.4525 | 0.9991 | 1.1227 | 1.3606 | 1.1689 | | RP287 | UL_S_DS | 0.1724 | 0.0599 | 0.8639 | 1.0492 | 1.0118 | 1.0929 | | RP288 | UL_S_DS | 0.3098 | 0.8442 | 0.9193 | 1.1453 | 0.4823 | 1.2178 | | RP289 | UL_S_DS | 0.7013 | 0.6404 | 0.8906 | 1.0485 | 0.5220 | 0.9159 | | RP290 | UL_S_DS | NA | 0.9286 | 0.8790 | 1.1575 | 0.9385 | 1.1822 | | RP291 | UL_S_DS | -0.9395 | 0.9727 | 0.9168 | 1.1727 | 0.7219 | 1.2287 | | RP292 | UL_S_DS | 4.9726 | 2.0739 | 1.4415 | 1.2386 | 1.8048 | 1.3527 | | RP293 | UL_S_DS | -1.0648 | 0.8385 | 0.8007 | 1.1320 | 1.0313 | 1.2459 | | RP294 | UL_S_DS | 1.7287 | 0.4525 | 0.7552 | 0.0363 | 0.4494 | 0.2291 | | RP295 | UL_S_DS | 1.3731 | 0.2820 | 0.6575 | -0.8160 | 0.3714 | 0.0150 | | RP296 | UL_S_DS | NA | 0.8379 | 0.7762 | 1.2154 | 1.1487 | 1.1713 | | RP297 | UL_S_DS | -0.0277 | 0.7661 | 0.8602 | 0.9962 | 0.8493 | 1.0526 | | RP298 | UL_S_DS | -1.2950 | 0.8826 | 0.9799 | 1.2103 | 1.3122 | 1.1365 | | **Study designation** | **Condition** | **DTF_DSI** | **PH_DSI** | **BMDW_DSI** | **GY_DSI** | **TGW_DSI** | **SPKFT_DSI** | | RP299 | UL_S_DS | 0.8163 | 0.5035 | 1.0595 | 1.0153 | NA | 0.8418 | | RP300 | UL_S_DS | -1.2471 | 0.8253 | 0.7102 | 1.1930 | 0.9385 | 1.2659 |   **Table S3** Characterisation of the marker set of 215,250 SNPs | | | | | | | | | | | | | | |
| --- | --- | --- | --- | --- | --- | --- | --- | --- | --- | --- | --- | --- | --- | --- |
|  |  | **Rice 12 chromosomes** | | | | | | | | | | | |  |
| **Table S2** | **Chr** | Chr1 | Chr2 | Chr3 | Chr4 | Chr5 | Chr6 | Chr7 | Chr8 | Chr9 | Chr10 | Chr11 | Chr12 | All |
|  | **Size (bp)** | 43,254,181 | 35,933,118 | 36,411,206 | 35,498,858 | 29,956,679 | 31,236,551 | 29,671,737 | 28,438,543 | 22,936,631 | 23,202,713 | 29,017,568 | 27,530,374 | 373,088,159 |
| **Distribution of distance (kb) between two adjacent SNP loci** | **Number** | 22,346 | 21,868 | 15,483 | 23,830 | 14,719 | 16,291 | 15,204 | 19,330 | 12,224 | 11,215 | 26,015 | 16,725 | 215,250 |
|  | **density** | 1,936 | 1,643 | 2,352 | 1,490 | 2,035 | 1,917 | 1,952 | 1,471 | 1,876 | 2,069 | 1,115 | 1,646 | 1,733 |
|  | **Minimum** | 137 | 211 | 236 | 179 | 302 | 37 | 263 | 179 | 253 | 48 | 8 | 274 | 177 |
|  | **1st Quartile** | 373 | 337 | 360 | 300 | 354 | 407 | 404 | 413 | 397 | 344 | 462 | 391 | 378 |
|  | **Median** | 472 | 493 | 387 | 438 | 456 | 476 | 457 | 650 | 530 | 412 | 773 | 497 | 503 |
|  | **3rd Quartile** | 574 | 688 | 440 | 743 | 572 | 581 | 578 | 904 | 628 | 576 | 1,231 | 695 | 684 |
|  | **Maximum** | 1,077 | 3,549 | 821 | 3,089 | 1,066 | 1,092 | 1,077 | 1,216 | 1,103 | 893 | 1,916 | 1,690 | 1,549 |
| **Distribution of the minor allele frequency (MAF)** | **Minimum** | 0.02 | 0.02 | 0.02 | 0.02 | 0.02 | 0.02 | 0.02 | 0.02 | 0.02 | 0.02 | 0.02 | 0.02 | 0.02 |
|  | **1st Quartile** | 0.04 | 0.04 | 0.04 | 0.04 | 0.04 | 0.04 | 0.04 | 0.04 | 0.04 | 0.04 | 0.04 | 0.04 | 0.04 |
|  | **Median** | 0.11 | 0.10 | 0.10 | 0.09 | 0.08 | 0.10 | 0.08 | 0.10 | 0.09 | 0.10 | 0.11 | 0.10 | 0.09 |
|  | **3rd Quartile** | 0.24 | 0.24 | 0.22 | 0.21 | 0.19 | 0.22 | 0.18 | 0.23 | 0.21 | 0.24 | 0.24 | 0.22 | 0.22 |
|  | **Maximum** | 0.50 | 0.50 | 0.50 | 0.50 | 0.50 | 0.50 | 0.50 | 0.50 | 0.50 | 0.50 | 0.50 | 0.50 | 0.50 |
| **Distribution of heterozygosity across chromosomes/ sites** | **Minimum** | 0.00% | 0.00% | 0.00% | 0.00% | 0.00% | 0.00% | 0.00% | 0.00% | 0.00% | 0.00% | 0.00% | 0.00% | 0.00% |
|  | **1st Quartile** | 0.00% | 0.00% | 0.00% | 0.00% | 0.00% | 0.00% | 0.00% | 0.00% | 0.00% | 0.00% | 0.00% | 0.00% | 0.00% |
|  | **Median** | 0.36% | 0.36% | 0.36% | 0.36% | 0.36% | 0.36% | 0.36% | 0.36% | 0.36% | 0.36% | 0.36% | 0.36% | 0.36% |
|  | **3rd Quartile** | 1.07% | 1.07% | 1.07% | 1.07% | 1.07% | 1.07% | 1.43% | 1.07% | 1.43% | 1.43% | 1.07% | 1.43% | 1.19% |
|  | **Maximum** | 5.00% | 5.00% | 5.00% | 5.00% | 5.00% | 5.00% | 5.00% | 5.00% | 5.00% | 5.00% | 5.00% | 5.00% | 5.00% |

**Table S4** Significant MTAs detected for ten traits in the population and the gene validation results

| **Trait** | **QTL** | **Env** | **Season** | **Chrom** | **Position** | **p-value** | **Locus name (MSU 7.0) / Drought QTL (qDTY)** | **Gene product description** |
| --- | --- | --- | --- | --- | --- | --- | --- | --- |
| HI | - | LL_N | 2015DS | 1 | 1670618 | 1.30E-09 | - | - |
| GY | *qGY*_1-1_ | UL_S | 2014WS | 1 | 3473291 | 3.21E-12 | - | - |
| GY |  | LL_S | 2015DS | 1 | 3824622 | 8.65E-13 | - | - |
| HI | - | UL_S | 2015DS | 1 | 4520401 | 2.40E-08 | - | - |
| HI | - | UL_S | 2015DS | 1 | 5997070 | 4.19E-10 | - | - |
| HI | - | UL_S | 2015DS | 1 | 7448895 | 4.85E-18 | - | - |
| SPKFT | - | LL_N | 2015DS | 1 | 14729531 | 1.97E-08 | - | - |
| SPKFT | - | LL_N | 2015DS | 1 | 15696481 | 2.59E-08 | - | - |
| GY | - | LL_S | 2015DS | 1 | 17515001 | 4.26E-08 | - | - |
| PH | - | LL_S | 2015DS | 1 | 17986986 | 4.60E-08 | - | - |
| GY | - | UL_S | 2015DS | 1 | 24237011 | 4.13E-16 | - | - |
| GY | - | UL_S | 2015DS | 1 | 24304274 | 2.33E-19 | LOC_Os01g43450 | expressed protein |
| GY | - | UL_S | 2015DS | 1 | 27277198 | 1.34E-12 | - | - |
| PH | *qPH*_1-1_ | LL_N | 2014WS | 1 | 33418648 | 5.31E-10 | LOC_Os01g53670 | ENTH domain containing protein C expressed |
| PH |  | UL_S | 2015DS | 1 | 33689947 | 2.29E-09 | - | - |
| PH |  | LL_S | 2015DS | 1 | 34359106 | 2.70E-09 | - | - |
| PH |  | LL_N | 2014WS / 2015DS | 1 | 34400345 | 4.66E-09 / 5.57E-11 | - | - |
| PH |  | LL_S | 2015DS | 1 | 34400345 | 3.71E-10 | LOC_Os01g59760 | bZIP transcription factor C putative C expressed |
|  |  |  |  |  |  |  |  |  |
|  |  |  |  |  |  |  |  |  |
|  |  |  |  |  |  |  |  |  |
| **Trait** | **QTL** | **Env** | **Season** | **Chrom** | **Position** | **p-value** | **Locus name (MSU 7.0) / Drought QTL (qDTY)** | **Gene product description** |
| NBP | - | LL_N | 2015DS | 1 | 37770897 | 1.63E-10 | *qDTY_1.1_* | *qDTY_1.1_* |
| PH | *qPH*_1-2_ | LL_N | 2015DS | 1 | 37960019 | 2.85E-09 |  |  |
| PH |  | UL_S | 2015DS | 1 | 38418739 | 4.45E-15 |  |  |
| PH |  | UL_S | 2015DS | 1 | 39044781 | 2.26E-11 |  |  |
| GY | *qGY*_1-2_ | LL_S | 2015DS/ 2014WS/ combined | 1 | 39987434 | 4.60E-08 |  |  |
| GY |  | LL_S | 2015DS/ 2014WS/ combined | 1 | 40017809 | 2.47E-10 |  |  |
| GY |  | UL_S | 2015DS/ 2014WS/ combined | 1 | 40390527 | 3.21E-12 |  |  |
| NBP | - | LL_N | 2015DS | 1 | 42056650 | 2.97E-09 | - | - |
| PH | - | LL_N | 2015DS | 2 | 7074179 | 3.26E-09 | - | - |
| TGW | - | LL_N | 2015DS | 2 | 8191777 | 2.77E-08 | - | - |
| GY | - | UL_S | 2014WS | 2 | 12985560 | 3.21E-12 | - | - |
| TGW | - | LL_N | 2015DS | 2 | 13051747 | 2.47E-10 | - | - |
| BMDW | - | LL_N | 2015DS | 2 | 13987526 | 4.13E-09 | - | - |
| BMDW | - | LL_N | 2015DS | 2 | 15644298 | 6.47E-11 | - | - |
| BMDW | - | LL_N | 2015DS | 2 | 17808669 | 1.21E-10 | LOC_Os02g30270 | expressed protein |
|  |  |  |  |  |  |  |  |  |
|  |  |  |  |  |  |  |  |  |
|  |  |  |  |  |  |  |  |  |
| **Trait** | **QTL** | **Env** | **Season** | **Chrom** | **Position** | **p-value** | **Locus name (MSU 7.0) / Drought QTL (qDTY)** | **Gene product description** |
| GY | *qGY*_2-1_ | UL_S | 2015DS | 2 | 18794394 | 2.42E-08 | - | - |
| GY |  | UL_S | 2015DS | 2 | 19821147 | 2.80E-18 | - | - |
| GY |  | UL_S | 2015DS/ 2014WS/ combined | 2 | 20248668 | 4.19E-08 | LOC_Os02g33620 | retrotransposon protein C putative C unclassified C expressed |
| GY |  | UL_S | 2015DS | 2 | 20774488 | 2.22E-08 | - | - |
| GY |  | UL_S | 2014WS | 2 | 21953460 | 6.13E-15 | LOC_Os02g36190 | cytochrome P450 C putative C expressed |
| GY |  | UL_S | 2015DS/ 2014WS/ combined | 2 | 22142784 | 3.27E-14 | - | - |
| GY |  | UL_S | 2015DS | 2 | 22201226 | 4.25E-13 | - | - |
| GY |  | UL_S | 2015DS/ combined | 2 | 23065933 | 2.19E-13 | - | - |
| SPKFT | - | UL_S | 2015DS | 2 | 24695278 | 1.13E-12 | - | - |
| HI | - | LL_N | 2015DS | 2 | 24797737 | 1.96E-11 | LOC_Os02g40920 | transposon protein C putative C CACTA C En FSpm sub-class C expressed |
| HI | - | LL_N | 2015DS | 2 | 25205930 | 1.20E-08 | LOC_Os02g42020 | expressed protein |
| GY | *qGY*_2-2_ | UL_S | 2015DS | 2 | 25979458 | 7.03E-09 | *qDTY_2.3_* | *qDTY_2.3_* |
| GY |  | UL_S | 2014WS | 2 | 29167141 | 3.80E-11 |  |  |
| NBP | - | LL_S | 2015DS | 2 | 34406178 | 9.19E-12 | - | - |
| PH | - | LL_N | 2015DS | 2 | 34710697 | 6.94E-09 | - | - |
| **Trait** | **QTL** | **Env** | **Season** | **Chrom** | **Position** | **p-value** | **Locus name (MSU 7.0) / Drought QTL (qDTY)** | **Gene product description** |
| TGW | - | LL_S | 2015DS | 3 | 4775424 | 6.08E-29 | - | - |
| HI | - | LL_N | 2014WS | 3 | 12571062 | 5.12E-10 | - | - |
| GY | - | UL_S | 2014WS | 3 | 13543585 | 3.61E-08 | - | - |
| NBP | - | LL_N | 2015DS | 3 | 14390997 | 1.14E-08 | - | - |
| GY | - | UL_S | 2014WS | 3 | 28646420 | 1.85E-08 | LOC_Os03g49650 | retrotransposon protein C putative C Ty1-copia subclass |
| PH | - | LL_S | 2014WS | 3 | 33600040 | 1.08E-08 | LOC_Os03g58220 | transposon protein C putative C unclassified C expressed |
| PH | - | LL_S | 2014WS | 3 | 33600989 | 3.30E-09 | LOC_Os03g58220 | transposon protein C putative C unclassified C expressed |
| GY | - | UL_S | 2014WS | 4 | 445488 | 4.55E-11 | - | - |
| GY | - | UL_S | 2014WS | 4 | 801040 | 3.79E-08 | - | - |
| GY | - | UL_S | 2014WS | 4 | 1441702 | 4.15E-08 | LOC_Os04g25400 | uncharacterized protein all1601 C putative C expressed |
| GY | - | UL_S | 2014WS | 4 | 1598504 | 1.03E-13 | - | - |
| SPKFT | - | LL_N | 2015DS | 4 | 6528566 | 2.90E-09 | - | - |
| SPKFT | - | LL_N | 2015DS | 4 | 7496029 | 1.52E-08 | - | - |
| SPKFT | - | UL_S | 2015DS | 4 | 15860075 | 3.15E-11 | - | - |
| TGW | - | LL_S | 2015DS | 4 | 19096486 | 2.21E-12 | - | - |
| NBP | - | LL_N | 2014WS | 4 | 22161787 | 9.59E-11 | - | - |
| GY | - | LL_S | 2015DS | 4 | 25920763 | 6.20E-09 | LOC_Os04g37410 | expressed protein |
| GY | - | LL_S | 2015DS | 4 | 31088649 | 1.68E-10 | LOC_Os04g50150 | retrotransposon protein C putative C unclassified C expressed |
| **Trait** | **QTL** | **Env** | **Season** | **Chrom** | **Position** | **p-value** | **Locus name (MSU 7.0) / Drought QTL (qDTY)** | **Gene product description** |
| PH | - | LL_S | 2015DS | 4 | 31375912 | 3.93E-08 | - | - |
| GY / HI | - | UL_S | 2015DS | 5 | 352058 | 2.89E-15 | - | - |
| GY | - | UL_S | 2014WS | 5 | 594868 | 2.84E-08 | - | - |
| GY | - | UL_S | 2015DS | 5 | 722162 | 1.84E-11 | - | - |
| GY | - | UL_S | 2014WS | 5 | 2683697 | 4.87E-12 | - | - |
| GY / HI / SPKFT | *qGY*_5-1_ | UL_S | 2015DS | 5 | 4140355 | 1.96E-19 | - | - |
| GY / HI |  | UL_S | 2015DS | 5 | 4266313 | 3.03E-21 | - | - |
| GY |  | UL_S | 2014WS | 5 | 4502747 | 1.85E-14 | - | - |
| GY |  | UL_S | 2014WS | 5 | 4502961 | 2.68E-16 | - | - |
| GY |  | UL_S | 2014WS | 5 | 4505637 | 1.85E-14 | - | - |
| HI | - | UL_S | 2015DS | 5 | 5357669 | 4.27E-08 | - | - |
| GY | - | UL_S | 2015DS | 5 | 5522232 | 4.02E-13 | - | - |
| GY | - | UL_S | 2014WS | 5 | 5824772 | 2.44E-10 | - | - |
| HI | - | LL_N | 2015DS | 5 | 5891992 | 1.03E-17 | LOC_Os05g10700 | retrotransposon protein C putative C unclassified C expressed |
| GY | - | UL_S | 2015DS | 5 | 6254266 | 3.15E-08 | - | - |
| HI | - | UL_S | 2015DS | 5 | 6523238 | 4.45E-13 | - | - |
| SPKFT | - | UL_S | 2015DS | 5 | 6747589 | 4.02E-09 | - | - |
| GY | - | UL_S | 2015DS | 5 | 7777995 | 8.82E-12 | - | - |
| HI | - | UL_S | 2015DS | 5 | 8338589 | 2.73E-08 | - | - |
| HI | - | UL_S | 2015DS | 5 | 8505783 | 1.79E-09 | - | - |
| GY | - | UL_S | 2014WS | 5 | 12494100 | 7.19E-12 | LOC_Os05g20900 | retrotransposon protein C putative C unclassified |
| GY | - | UL_S | 2014WS | 5 | 14325590 | 3.21E-12 | - | - |
| **Trait** | **QTL** | **Env** | **Season** | **Chrom** | **Position** | **p-value** | **Locus name (MSU 7.0) / Drought QTL (qDTY)** | **Gene product description** |
| GY | - | LL_S | 2015DS | 5 | 19224697 | 1.71E-09 | LOC_Os05g32660 | leucine-rich repeat family protein C putative C expressed |
| GY | *qGY_5-2_* | UL_S | 2014WS | 5 | 24708194 | 1.85E-14 | OsRPK1, OsCCaMK, OsHAP3B, OsTPS1, OsSTN8 | OsRPK1, OsCCaMK, OsHAP3B, OsTPS1, OsSTN8 |
| GY |  | UL_S | 2014WS | 5 | 29913640 | 3.21E-12 |  |  |
| GY | - | UL_S | 2015DS | 6 | 3233993 | 3.21E-12 | - | - |
| GY | - | UL_S | 2015DS | 6 | 3723575 | 3.44E-13 | - | - |
| GY | - | UL_S | 2014WS | 6 | 5883556 | 8.47E-15 | - | - |
| GY | - | UL_S | 2014WS | 6 | 6025083 | 2.02E-08 | LOC_Os06g11540 | retrotransposon protein C putative C unclassified C expressed |
| GY | - | UL_S | 2015DS | 6 | 7472450 | 2.43E-09 | - | - |
| DTF | *qDTF*_6-1_ | LL_N | 2014WS/ combined | 6 | 7611279 | 2.23E-10 | - | - |
| DTF |  | LL_N | 2015DS/ 2014WS/ combined | 6 | 7749410 | 5.38E-16 / 2.002E-08 | - | - |
| DTF | *qDTF*_6-2_ | LL_N | 2015DS | 6 | 9539728 | 1.66E-08 | - | - |
| DTF |  | LL_N | combined | 6 | 10279310 | 3.85E-08 | - | - |
| DTF |  | LL_N | 2015DS | 6 | 10371528 | 3.94E-09 | - | - |
| GY | - | UL_S | 2014WS | 6 | 12392812 | 3.21E-12 | - | - |
| PH | - | LL_N | 2014WS | 6 | 13439145 | 1.02E-10 | OsPT9, OsPT1055,OsGLK1, nyc3 | OsPT9, OsPT1055,OsGLK1, nyc3 |
| TGW | - | LL_S | 2015DS | 6 | 16493040 | 3.59E-08 | - | - |
| **Trait** | **QTL** | **Env** | **Season** | **Chrom** | **Position** | **p-value** | **Locus name (MSU 7.0) / Drought QTL (qDTY)** | **Gene product description** |
| PH | - | LL_N | 2014WS | 6 | 19486804 | 3.17E-16 | - | - |
| GY | - | UL_S | 2014WS | 6 | 22794237 | 1.20E-09 | LOC_Os06g37850 | transposon protein C putative C CACTA C En FSpm sub-class C expressed |
| GY | - | UL_S | 2014WS | 6 | 23132086 | 3.53E-14 | LOC_Os06g39690 | SOUL heme-binding protein C putative C expressed |
| TGW | - | LL_S | 2015DS | 6 | 23403566 | 6.22E-13 | - | - |
| GY | - | UL_S | 2014WS | 6 | 26672678 | 3.21E-12 | - | - |
| GY | - | UL_S | 2014WS | 6 | 27655258 | 2.80E-11 | LOC_Os06g47030 | expressed protein |
| SPKFT | - | UL_S | 2015DS | 6 | 28876857 | 3.26E-08 | LOC_Os06g49060 | ternary complex factor MIP1 C putative C expressed |
| HI | - | UL_S | 2015DS | 6 | 29380888 | 8.34E-09 | - | - |
| PH | - | LL_N | 2015DS | 6 | 30802585 | 1.17E-08 | - | - |
| PH | - | LL_N | 2015DS | 6 | 30807826 | 1.56E-09 | - | - |
| GY | - | UL_S | 2014WS | 6 | 31179920 | 1.20E-08 | LOC_Os06g49910 | retrotransposon protein C putative C unclassified |
| DTF | - | LL_S | 2015DS | 7 | 5093247 | 2.26E-08 | - | - |
| GY | - | UL_S | 2015DS | 7 | 11494665 | 7.47E-21 | - | - |
| GY | - | UL_S | 2014WS | 7 | 12116041 | 3.86E-11 | - | - |
| HI | - | LL_S | 2015DS | 7 | 12460909 | 5.98E-12 | - | - |
| GY | - | UL_S | 2015DS | 7 | 13019061 | 1.90E-10 | LOC_Os07g23450 | ZOS7-04 - C2H2 zinc finger protein C expressed |
| GY | - | UL_S | 2015DS | 7 | 14683350 | 2.68E-08 | - | - |
| NBP | - | LL_S | 2015DS | 7 | 15048319 | 5.42E-08 | - | - |
| GY | - | UL_S | 2014WS | 7 | 15125997 | 5.94E-09 | - | - |
| **Trait** | **QTL** | **Env** | **Season** | **Chrom** | **Position** | **p-value** | **Locus name (MSU 7.0) / Drought QTL (qDTY)** | **Gene product description** |
| GY | - | UL_S | 2014WS | 7 | 16180595 | 3.42E-08 | LOC_Os07g27900 | retrotransposon protein C putative C Ty1-copia subclass |
| GY | - | UL_S | 2014WS | 7 | 16181810 | 1.66E-12 | LOC_Os07g27900 | retrotransposon protein C putative C Ty1-copia subclass |
| GY | - | UL_S | 2014WS | 7 | 16183053 | 1.17E-11 | LOC_Os07g27900 | retrotransposon protein C putative C Ty1-copia subclass |
| SPKFT | - | LL_N | 2014WS | 7 | 16855834 | 2.65E-08 | - | - |
| SPKFT | - | LL_N | 2014WS | 7 | 18448562 | 5.00E-16 | - | - |
| DTF | - | LL_N | 2014WS | 7 | 19598023 | 5.53E-09 | LOC_Os07g32800 | autophagy-related protein C putative C expressed |
| PH | - | LL_S | 2014WS | 7 | 19689644 | 4.48E-20 | - | - |
| DTF | - | LL_N | 2015DS | 7 | 20159780 | 6.62E-12 | LOC_Os07g32820 | expressed protein |
| SPKFT | - | LL_N | 2014WS | 7 | 27596132 | 4.53E-08 | - | - |
| BMDW | *qBMDW*_8-1_ | LL_S | 2015DS/ 2014WS/ combined | 8 | 466752 | 1.25E-05 | - | - |
| BMDW |  | LL_S | 2015DS/ 2014WS/ combined | 8 | 500943 | 3.82E-06 | - | - |
| BMDW |  | LL_S | 2015DS/ 2014WS/ combined | 8 | 541934 | 1.81E-05 | - | - |
| **Trait** | **QTL** | **Env** | **Season** | **Chrom** | **Position** | **p-value** | **Locus name (MSU 7.0) / Drought QTL (qDTY)** | **Gene product description** |
| PH | - | UL_S | 2015DS | 8 | 654862 | 1.57E-08 | - | - |
| GY / HI | - | UL_S | 2015DS | 8 | 857745 | 1.61E-23 | - | - |
| GY | - | UL_S | 2015DS | 8 | 2601016 | 3.30E-08 | - | - |
| PH | - | LL_N | 2014WS | 8 | 3543841 | 2.77E-08 | - | - |
| GY | - | UL_S | 2015DS | 8 | 4420926 | 2.70E-08 | - | - |
| DTF | - | LL_S | 2015DS | 8 | 5367927 | 3.49E-08 | - | - |
| GY | - | UL_S | 2014WS | 8 | 5755212 | 6.96E-11 | - | - |
| GY | - | UL_S | 2014WS | 8 | 6119419 | 2.85E-12 | - | - |
| GY | - | UL_S | 2015DS | 8 | 6560107 | 2.59E-08 | - | - |
| DTF | - | LL_N | 2014WS | 8 | 17075677 | 3.42E-09 | - | - |
| TGW | - | LL_S | 2015DS | 8 | 19133569 | 9.75E-13 | - | - |
| NBP | - | LL_S | 2015DS | 8 | 20972048 | 3.71E-09 | - | - |
| NBP | - | LL_N | 2014WS | 8 | 22460760 | 4.44E-09 | - | - |
| HI | - | LL_S | 2015DS | 8 | 24471764 | 5.04E-10 | - | - |
| GY | - | UL_S | 2014WS | 9 | 366317 | 1.29E-10 | - | - |
| NBP | - | LL_S | 2015DS | 9 | 990034 | 5.54E-08 | - | - |
| SPKFT | - | LL_N | 2015DS | 9 | 1671524 | 1.72E-10 | - | - |
| GY | - | UL_S | 2014WS | 9 | 3032058 | 3.21E-12 | LOC_Os09g06650 | retrotransposon C putative C centromere-specific C expressed |
| SPKFT | - | LL_N | 2015DS | 9 | 4622275 | 4.69E-09 | - | - |
| GY | - | UL_S | 2014WS | 9 | 5467194 | 4.27E-23 | LOC_Os09g10300 | Skp1 family C dimerisation domain containing protein C expressed |
| GY | - | UL_S | 2014WS | 9 | 7290176 | 2.44E-12 | - | - |
| **Trait** | **QTL** | **Env** | **Season** | **Chrom** | **Position** | **p-value** | **Locus name (MSU 7.0) / Drought QTL (qDTY)** | **Gene product description** |
| SPKFT | *qSPKFT*_9-1_ | LL_N | 2015DS | 9 | 9426722 | 3.66E-11 | - | - |
| SPKFT |  | LL_N | 2015DS | 9 | 10118145 | 8.13E-11 | - | - |
| PH |  | LL_S | 2015DS / combined | 9 | 11072513 | 9.79E-10 | - | - |
| SPKFT |  | LL_N | 2015DS/ 2014WS/ combined | 9 | 11791517 | 0.000158 | - | - |
| SPKFT |  | LL_N | 2015DS/ 2014WS/ combined | 9 | 11838142 | 0.000118 | - | - |
| PH | *qPH*_9-1_ | UL_S | 2015DS | 9 | 13423222 | 1.94E-12 | *qDTY_9.1_* | *qDTY_9.1_* |
| DTF |  | LL_S | 2015DS | 9 | 13934171 | 2.06E-15 |  |  |
| PH |  | LL_N | 2015DS | 9 | 15970207 | 1.81E-14 |  |  |
| PH |  | UL_S | 2015DS | 9 | 16154337 | 3.63E-09 |  |  |
| BMDW / NBP | *qBMDW-NBP*_9-1_ | LL_N | 2015DS/ 2014WS/ combined | 9 | 19316065 | 3.69E-08 / 1.6E-13 | LOC_Os09g36290 | Ser FThr protein phosphatase family protein C putative C expressed |
| NBP |  | LL_N | 2015DS | 9 | 20908000 | 7.08E-09 | - | - |
| BMDW / NBP |  | LL_N | 2015DS/ 2014WS/ combined | 9 | 20944019 | 2.3E-12 / 2.46E-11 | LOC_Os09g36330 | expressed protein |
| NBP |  | LL_N | 2014WS | 9 | 21337553 | 4.43E-13 | - | - |
| **Trait** | **QTL** | **Env** | **Season** | **Chrom** | **Position** | **p-value** | **Locus name (MSU 7.0) / Drought QTL (qDTY)** | **Gene product description** |
| GY | - | UL_S | 2015DS | 10 | 4716948 | 5.91E-10 | - | - |
| SPKFT | - | LL_N | 2014WS | 10 | 5092509 | 4.88E-15 | - | - |
| GY | - | UL_S | 2015DS | 10 | 9297675 | 2.44E-08 | - | - |
| DTF | - | LL_S | 2015DS | 11 | 2557438 | 2.73E-08 | - | - |
| TGW | - | LL_N | 2015DS | 11 | 2678930 | 3.33E-11 | - | - |
| PH | - | LL_N | 2015DS | 11 | 5630523 | 2.18E-12 | - | - |
| DTF | *qDTF*_11-1_ | LL_S | 2015DS/ 2014WS/ combined | 11 | 6525213 | 2.45E-05 | - | - |
| DTF |  | LL_S | 2015DS/ 2014WS/ combined | 11 | 6604428 | 3.34E-05 | - | - |
| DTF |  | LL_S | 2015DS/ 2014WS/ combined | 11 | 6602990 | 5.28E-05 | - | - |
| DTF |  | LL_S | 2015DS | 11 | 7215923 | 1.37E-10 | LOC_Os11g12760 | O-methyltransferase C putative C expressed |
| DTF |  | LL_S | 2015DS | 11 | 7215940 | 3.24E-13 | LOC_Os11g12760 | O-methyltransferase C putative C expressed |
| **Trait** | **QTL** | **Env** | **Season** | **Chrom** | **Position** | **p-value** | **Locus name (MSU 7.0) / Drought QTL (qDTY)** | **Gene product description** |
| HI | - | UL_S | 2015DS | 11 | 10053723 | 5.67E-14 | LOC_Os11g18366 | cycloartenol synthase C putative C expressed |
| HI | - | LL_N | 2015DS | 11 | 10351950 | 1.50E-08 | LOC_Os11g19230 | retrotransposon protein C putative C Ty1-copia subclass C expressed |
| SPKFT | - | UL_S | 2015DS | 11 | 11165817 | 2.61E-08 | - | - |
| GY | - | LL_S | 2015DS | 11 | 11817831 | 1.86E-11 | - | - |
| PH | - | UL_S | 2015DS | 11 | 15756517 | 2.86E-09 | - | - |
| GY | - | UL_S | 2014WS | 11 | 16248710 | 3.08E-08 | qGP-11, qGI-11, yld11.1, gpl11.1, gl11.1 | qGP-11, qGI-11, yld11.1, gpl11.1, gl11.1 |
| DTF | - | LL_S | 2015DS | 11 | 18836588 | 4.86E-09 | LOC_Os11g31980 | OsSCP63 - Putative Serine Carboxypeptidase homologue C expressed |
| PH | - | LL_N | 2015DS | 11 | 20143839 | 3.31E-09 | LOC_Os11g34364 | armadillo Fbeta-catenin-like repeat containing protein C expressed |
| GY | - | UL_S | 2014WS | 11 | 20668615 | 4.67E-10 | LOC_Os11g35310 | phytosulfokines precursor C putative C expressed |
| GY | - | UL_S | 2014WS | 11 | 21092857 | 5.75E-09 | - | - |
| **Trait** | **QTL** | **Env** | **Season** | **Chrom** | **Position** | **p-value** | **Locus name (MSU 7.0) / Drought QTL (qDTY)** | **Gene product description** |
| GY | - | UL_S | 2014WS | 11 | 21311326 | 2.84E-12 | LOC_Os11g36200 | receptor-like protein kinase 2 precursor C putative C expressed |
| PH | - | UL_S | 2015DS | 11 | 22175365 | 4.18E-09 | qGP-11,qGl-11, yld11.1, gpl11.1, gw11.1 | qGP-11,qGl-11, yld11.1, gpl11.1, gw11.1 |
| HI | - | LL_N | 2015DS | 11 | 23022593 | 2.02E-12 |  |  |
| SPKFT | - | UL_S | 2015DS | 11 | 24636744 | 5.49E-16 | - | - |
| PH | - | LL_S | 2014WS | 11 | 24761315 | 2.98E-12 | - | - |
| PH | - | UL_S | 2015DS | 11 | 25597507 | 1.37E-08 | - | - |
| GY | - | UL_S | 2014WS | 11 | 26464313 | 3.21E-12 | - | - |
| PH | - | LL_S | 2014WS | 11 | 28779480 | 1.50E-09 | - | - |
| PH | - | LL_S | 2014WS | 11 | 28789891 | 1.39E-10 | - | - |
| GY | - | LL_S / UL_S | 2015DS | 12 | 1642245 | 2.72E-25 | - | - |
| GY | - | UL_S | 2015DS | 12 | 2835650 | 1.82E-09 | LOC_Os12g06020 | mRNA-decapping enzyme C putative C expressed |
| GY | - | UL_S | 2015DS | 12 | 3848827 | 4.24E-08 | - | - |
| GY | - | UL_S | 2015DS | 12 | 4340342 | 6.06E-21 | - | - |
| GY | - | UL_S | 2015DS | 12 | 7226659 | 3.28E-08 | - | - |
| DTF | - | LL_S | 2015DS | 12 | 7712803 | 2.65E-11 | - | - |
| GY | - | UL_S | 2015DS | 12 | 7778770 | 4.13E-17 | - | - |
| DTF | - | LL_N | 2015DS | 12 | 9203018 | 9.95E-09 | - | - |
| HI | - | UL_S | 2015DS | 12 | 10391392 | 1.66E-19 | - | - |
| TGW | - | LL_S | 2015DS | 12 | 11404416 | 2.22E-13 | - | - |
| **Trait** | **QTL** | **Env** | **Season** | **Chrom** | **Position** | **p-value** | **Locus name (MSU 7.0) / Drought QTL (qDTY)** | **Gene product description** |
| GY | - | UL_S | 2014WS | 12 | 11591239 | 2.05E-08 | - | - |
| GY | - | UL_S | 2014WS | 12 | 12177156 | 1.58E-15 | - | - |
| GY | - | UL_S | 2014WS | 12 | 12704766 | 4.26E-09 | - | - |
| PH | - | LL_N | 2015DS | 12 | 16502509 | 4.67E-10 | - | - |
| HI | - | LL_S | 2015DS | 12 | 17641543 | 2.65E-12 | - | - |
| GY | *qGY*_12-1_ | UL_S | 2014WS | 12 | 18165164 | 1.34E-09 | *qDTY_12.1_* | *qDTY_12.1_* |
| GY |  | UL_S | 2015DS/ 2014WS/ combined | 12 | 18597220 | 4.69E-19 |  |  |
| GY |  | UL_S | 2015DS/ 2014WS/ combined | 12 | 19106346 | 2.96E-10 |  |  |
| HI | - | LL_N | 2014WS | 12 | 23250434 | 2.74E-08 | LOC_Os12g37850 | expressed protein |
| TGW | - | LL_S | 2015DS | 12 | 24105210 | 5.64E-09 | - | - |

**Table S5** Yadj. values used for GWAS of the ten traits measured for the 280 diverse set in three environments and two seasons

| **Study designation** | **3KG database designation** | **Env** | **Season** | **DTF** | **PH (cm)** | **PL (cm)** | **FlgLA (cm^2^)** | **NBP** | **BMDW (kgha^-1^)** | **GY (kgha^-1^)** | **HI** | **TGW (g)** | **SPKFT (%)** |
| --- | --- | --- | --- | --- | --- | --- | --- | --- | --- | --- | --- | --- | --- |
| RP002 | 19::IRGC 70786-1 | LL_S | WS | 0 | 119.20 | 19.25 | 42.72 | 31 | 3059.24 | 574.33 | 0.16 | 100.00 | 53.74 |
| RP003 | 3210::IRGC 116950-1 | LL_S | WS | 0 | 0.00 | 0.00 | 0.00 | 0 | 0.00 | 0.00 | 0.00 | 0.00 | 0.00 |
| RP004 | 498-2A BR 8::IRGC 5891-1 | LL_S | WS | 0 | 93.10 | 18.70 | 27.46 | 25 | 3686.99 | 586.82 | 0.16 | 230.00 | 59.75 |
| RP005 | ADT 12::IRGC 6254-1 | LL_S | WS | 82 | 149.76 | 27.65 | 56.10 | 35 | 3571.89 | 589.05 | 0.16 | 170.00 | 57.79 |
| RP006 | AE NOUA::IRGC 89308-1 | LL_S | WS | 79 | 142.67 | 20.70 | 39.38 | 20 | 4589.56 | 571.83 | 0.16 | 120.00 | 52.42 |
| RP007 | ALTAMIRA 9::IRGC 116953-1 | LL_S | WS | 0 | 0.00 | 0.00 | 0.00 | 0 | 0.00 | 0.00 | 0.00 | 0.00 | 0.00 |
| RP008 | ARAURE 1::IRGC 116956-1 | LL_S | WS | 0 | 84.15 | 23.41 | 30.64 | 28 | 3565.77 | 609.83 | 0.16 | 230.00 | 62.78 |
| RP009 | ARC 10100::IRGC 20709-1 | LL_S | WS | 78 | 118.11 | 21.98 | 60.35 | 21 | 2878.95 | 599.40 | 0.16 | 205.00 | 60.45 |
| RP010 | ARC 10594::IRGC 12524-1 | LL_S | WS | 82 | 108.64 | 23.03 | 42.67 | 30 | 3289.27 | 613.60 | 0.16 | 170.00 | 64.33 |
| RP011 | ARC 10754::IRGC 12603-1 | LL_S | WS | 82 | 121.20 | 23.75 | 35.71 | 31 | 3515.68 | 611.51 | 0.16 | 175.00 | 64.63 |
| RP012 | ARC 10812::IRGC 21074-1 | LL_S | WS | 0 | 141.86 | 22.50 | 41.87 | 15 | 4135.12 | 576.79 | 0.16 | 135.00 | 59.43 |
| RP013 | ARC 11524::IRGC 42672-1 | LL_S | WS | 83 | 127.15 | 21.85 | 53.65 | 21 | 3448.33 | 578.35 | 0.16 | 155.00 | 59.16 |
| RP014 | ARC 11857::IRGC 40972-1 | LL_S | WS | 0 | 133.40 | 22.43 | 33.49 | 15 | 4127.92 | 598.45 | 0.16 | 110.00 | 58.44 |
| RP015 | ARC 11901::IRGC 21727-1 | LL_S | WS | 87 | 137.62 | 23.16 | 40.00 | 22 | 3563.87 | 577.37 | 0.16 | 205.00 | 57.00 |
| RP018 | ARC 13778::IRGC 41216-1 | LL_S | WS | 0 | 99.24 | 20.57 | 42.27 | 20 | 4137.85 | 580.77 | 0.16 | 205.00 | 62.52 |
| RP019 | ARC 14060::IRGC 41374-1 | LL_S | WS | 0 | 116.32 | 19.96 | 45.12 | 34 | 4166.95 | 619.59 | 0.16 | 205.00 | 63.63 |
| RP020 | ARC 14064::IRGC 41377-1 | LL_S | WS | 0 | 133.01 | 21.95 | 35.87 | 17 | 3264.78 | 571.36 | 0.16 | 110.00 | 54.54 |
| RP021 | ARC 14654::IRGC 41663-1 | LL_S | WS | 0 | 106.57 | 20.80 | 36.53 | 35 | 4258.07 | 593.50 | 0.16 | 145.00 | 55.43 |
| RP022 | ARC 15873::IRGC 43250-1 | LL_S | WS | 0 | 0.00 | 0.00 | 0.00 | 0 | 0.00 | 0.00 | 0.00 | 0.00 | 0.00 |
| RP023 | ARC 18092::IRGC 42256-1 | LL_S | WS | 86 | 133.90 | 22.67 | 37.46 | 18 | 4147.63 | 596.17 | 0.16 | 125.00 | 60.95 |
| RP024 | ARC 18112::IRGC 42274-1 | LL_S | WS | 87 | 124.90 | 23.32 | 49.82 | 25 | 3735.78 | 575.05 | 0.16 | 150.00 | 56.16 |
| RP025 | ARC 18202::IRGC 42328-1 | LL_S | WS | 0 | 111.72 | 21.74 | 35.49 | 24 | 3664.47 | 597.27 | 0.16 | 210.00 | 61.37 |
| RP026 | ARC 18597::IRGC 43299-1 | LL_S | WS | 0 | 126.76 | 21.38 | 34.53 | 7 | 3299.94 | 571.43 | 0.16 | 180.00 | 54.22 |
| RP028 | ASHI BINNI::IRGC 77216-1 | LL_S | WS | 87 | 62.10 | 17.81 | 20.97 | 21 | 3794.55 | 574.45 | 0.16 | 105.00 | 57.40 |
| **Study designation** | **3KG database designation** | **Env** | **Season** | **DTF** | **PH (cm)** | **PL (cm)** | **FlgLA (cm^2^)** | **NBP** | **BMDW (kgha^-1^)** | **GY (kgha^-1^)** | **HI** | **TGW (g)** | **SPKFT (%)** |
| RP029 | ASHMBER::IRGC 27522-1 | LL_S | WS | 0 | 105.11 | 21.02 | 44.85 | 16 | 3868.58 | 570.88 | 0.16 | 95.00 | 57.18 |
| RP030 | ASU::IRGC 62154-1 | LL_S | WS | 80 | 106.34 | 21.20 | 44.44 | 26 | 3596.22 | 591.09 | 0.16 | 190.00 | 61.74 |
| RP031 | AUS 171::IRGC 29004-1 | LL_S | WS | 79 | 105.42 | 20.55 | 33.55 | 12 | 3164.16 | 572.79 | 0.16 | 195.00 | 55.05 |
| RP032 | AUS 177::IRGC 29009-1 | LL_S | WS | 87 | 103.82 | 21.48 | 40.54 | 21 | 2993.67 | 587.23 | 0.16 | 140.00 | 51.03 |
| RP033 | AUS 219::IRGC 29031-1 | LL_S | WS | 74 | 115.81 | 18.33 | 39.46 | 10 | 4213.82 | 586.99 | 0.16 | 280.00 | 61.42 |
| RP034 | AUS 233::IRGC 29036-1 | LL_S | WS | 80 | 122.47 | 22.55 | 43.35 | 41 | 4106.24 | 599.09 | 0.16 | 175.00 | 63.78 |
| RP036 | AUS 295::IRGC 29083-1 | LL_S | WS | 82 | 131.55 | 21.63 | 46.53 | 26 | 4614.97 | 586.32 | 0.16 | 210.00 | 52.48 |
| RP037 | AUS 301::IRGC 29089-1 | LL_S | WS | 73 | 105.16 | 20.28 | 37.10 | 13 | 3641.42 | 580.97 | 0.16 | 210.00 | 58.77 |
| RP038 | AUS 308::IRGC 29096-1 | LL_S | WS | 85 | 120.28 | 22.70 | 43.79 | 10 | 3218.80 | 580.58 | 0.16 | 210.00 | 60.28 |
| RP039 | AUS 329::IRGC 29116-1 | LL_S | WS | 76 | 119.59 | 21.27 | 50.52 | 23 | 3589.27 | 615.61 | 0.16 | 205.00 | 63.77 |
| RP040 | AUS 344::IRGC 29131-1 | LL_S | WS | 78 | 109.41 | 19.50 | 44.35 | 11 | 3735.64 | 575.38 | 0.16 | 170.00 | 55.65 |
| RP041 | AUS 439::IRGC 29221-1 | LL_S | WS | 83 | 108.08 | 21.27 | 41.77 | 27 | 3288.79 | 608.71 | 0.16 | 230.00 | 60.15 |
| RP042 | AUS PADDY (RED)::IRGC 44978-1 | LL_S | WS | 76 | 106.77 | 20.71 | 44.80 | 19 | 3841.85 | 571.50 | 0.16 | 135.00 | 55.02 |
| RP043 | B 4414 F-MR-6-3::IRGC 117310-1 | LL_S | WS | 0 | 0.00 | 0.00 | 0.00 | 0 | 0.00 | 0.00 | 0.00 | 0.00 | 0.00 |
| RP044 | B 6136 E-3-TB-0-1-5::IRGC 117311-1 | LL_S | WS | 84 | 89.63 | 19.25 | 40.98 | 34 | 3399.23 | 614.12 | 0.16 | 215.00 | 62.94 |
| RP045 | B 6136-3-TB-0-1-5::IRGC 117312-1 | LL_S | WS | 85 | 92.47 | 16.63 | 37.82 | 28 | 3939.07 | 637.98 | 0.16 | 230.00 | 62.46 |
| RP046 | B 6149 F-MR-7::IRGC 117314-1 | LL_S | WS | 87 | 139.65 | 21.77 | 39.31 | 34 | 4005.78 | 609.42 | 0.16 | 175.00 | 60.97 |
| RP047 | BA BAI GU::IRGC 79580-1 | LL_S | WS | 73 | 118.57 | 19.37 | 44.67 | 27 | 3567.44 | 598.57 | 0.16 | 155.00 | 61.81 |
| RP048 | BA SHI ZAO::IRGC 67903-1 | LL_S | WS | 76 | 112.14 | 21.89 | 50.98 | 33 | 3915.20 | 596.65 | 0.16 | 200.00 | 64.88 |
| RP049 | BADAL 1163::IRGC 32796-1 | LL_S | WS | 0 | 126.37 | 21.48 | 52.97 | 8 | 3916.32 | 568.88 | 0.16 | 85.00 | 52.69 |
| RP050 | BADUIE::IRGC 53715-1 | LL_S | WS | 0 | 131.94 | 22.67 | 34.61 | 12 | 3396.64 | 573.30 | 0.16 | 125.00 | 55.86 |
| RP051 | BAI HE::IRGC 76438-1 | LL_S | WS | 70 | 109.32 | 23.07 | 35.35 | 26 | 3196.66 | 598.86 | 0.16 | 190.00 | 61.54 |
| RP052 | BAIANG 6::IRGC 6129-1 | LL_S | WS | 0 | 103.97 | 20.83 | 39.34 | 50 | 4547.48 | 628.90 | 0.16 | 220.00 | 64.29 |
| RP053 | BAK TULSI::IRGC 34831-1 | LL_S | WS | 79 | 107.31 | 21.02 | 65.29 | 14 | 3392.53 | 573.44 | 0.16 | 190.00 | 59.03 |
| RP054 | BAKASI::IRGC 27074-1 | LL_S | WS | 0 | 98.44 | 19.91 | 41.88 | 47 | 3607.25 | 604.29 | 0.16 | 180.00 | 63.61 |
| RP055 | BALASURIYA A::IRGC 66509-1 | LL_S | WS | 0 | 101.90 | 18.19 | 38.67 | 24 | 4263.49 | 593.22 | 0.16 | 230.00 | 55.59 |
| RP056 | BAMOA A 75::IRGC 51101-1 | LL_S | WS | 0 | 78.82 | 20.76 | 47.06 | 17 | 3644.80 | 570.47 | 0.16 | 80.00 | 54.52 |
| RP057 | BANDI::IRGC 17214-1 | LL_S | WS | 0 | 0.00 | 0.00 | 0.00 | 0 | 0.00 | 0.00 | 0.00 | 0.00 | 0.00 |
| **Study designation** | **3KG database designation** | **Env** | **Season** | **DTF** | **PH (cm)** | **PL (cm)** | **FlgLA (cm^2^)** | **NBP** | **BMDW (kgha^-1^)** | **GY (kgha^-1^)** | **HI** | **TGW (g)** | **SPKFT (%)** |
| RP058 | BANGKOUY::IRGC 94037-1 | LL_S | WS | 0 | 135.47 | 22.86 | 51.83 | 2 | 3261.72 | 568.11 | 0.16 | 20.00 | 52.80 |
| RP059 | BARIK KUDI::IRGC 52807-1 | LL_S | WS | 81 | 100.74 | 18.33 | 35.15 | 35 | 3093.70 | 599.91 | 0.16 | 205.00 | 63.48 |
| RP060 | BAT DO::IRGC 7014-1 | LL_S | WS | 82 | 124.37 | 20.18 | 38.36 | 22 | 3490.84 | 599.33 | 0.16 | 225.00 | 61.06 |
| RP061 | BATHURI::IRGC 25838-1 | LL_S | WS | 77 | 115.11 | 21.22 | 50.45 | 28 | 3202.37 | 586.70 | 0.16 | 190.00 | 61.01 |
| RP062 | BAZAIL::IRGC 27526-1 | LL_S | WS | 0 | 118.73 | 20.04 | 44.85 | 18 | 3205.37 | 575.29 | 0.16 | 150.00 | 54.99 |
| RP063 | BENGALY MORIMO::IRGC 10976-1 | LL_S | WS | 78 | 120.62 | 20.33 | 41.44 | 21 | 3268.29 | 570.83 | 0.16 | 115.00 | 57.42 |
| RP065 | BHADOIA 303::IRGC 6588-1 | LL_S | WS | 75 | 124.05 | 18.03 | 32.56 | 25 | 4009.13 | 582.67 | 0.16 | 165.00 | 55.97 |
| RP066 | BK 26::IRGC 45197-1 | LL_S | WS | 0 | 118.27 | 17.42 | 48.88 | 46 | 3968.17 | 590.01 | 0.16 | 125.00 | 56.09 |
| RP067 | BKN BR 1031-78-5-4::IRGC 55927-1 | LL_S | WS | 0 | 0.00 | 0.00 | 0.00 | 0 | 0.00 | 0.00 | 0.00 | 0.00 | 0.00 |
| RP068 | BONG SEN::IRGC 7011-1 | LL_S | WS | 84 | 124.45 | 21.01 | 42.78 | 20 | 3791.10 | 578.76 | 0.16 | 165.00 | 60.64 |
| RP070 | BPI 76 NON SENSITIVE (GREEN)::IRGC 9790-1 | LL_S | WS | 0 | 106.49 | 21.13 | 44.43 | 23 | 3390.27 | 569.67 | 0.16 | 55.00 | 62.54 |
| RP071 | BR 51-115-4::IRGC 43999-1 | LL_S | WS | 0 | 100.19 | 19.45 | 35.18 | 29 | 4152.69 | 608.04 | 0.16 | 130.00 | 57.29 |
| RP072 | BR 5230-46-4::IRGC 117318-1 | LL_S | WS | 0 | 68.82 | 20.77 | 32.81 | 5 | 3344.74 | 579.42 | 0.16 | 170.00 | 60.20 |
| RP073 | BR IRGA 409::IRGC 55915-1 | LL_S | WS | 0 | 84.92 | 19.51 | 42.90 | 33 | 3764.22 | 588.33 | 0.16 | 190.00 | 60.71 |
| RP074 | BW 295-5::IRGC 63098-1 | LL_S | WS | 0 | 93.84 | 19.45 | 36.43 | 35 | 3634.89 | 601.18 | 0.16 | 215.00 | 63.15 |
| RP075 | C 1016-1::IRGC 50368-1 | LL_S | WS | 82 | 112.05 | 21.02 | 43.03 | 24 | 3370.77 | 592.77 | 0.16 | 175.00 | 60.16 |
| RP076 | C 166-135::IRGC 50633-1 | LL_S | WS | 0 | 116.69 | 20.53 | 42.56 | 14 | 3023.05 | 599.48 | 0.16 | 125.00 | 62.28 |
| RP077 | C 662083::IRGC 62101-1 | LL_S | WS | 0 | 74.62 | 18.92 | 39.54 | 26 | 4440.24 | 574.93 | 0.16 | 100.00 | 54.77 |
| RP079 | CAUVERY::IRGC 45255-1 | LL_S | WS | 78 | 75.20 | 17.54 | 27.49 | 22 | 3103.46 | 586.79 | 0.16 | 160.00 | 62.40 |
| RP080 | CE IN TSAN::IRGC 4362-1 | LL_S | WS | 0 | 0.00 | 0.00 | 0.00 | 0 | 0.00 | 0.00 | 0.00 | 0.00 | 0.00 |
| RP081 | CEA 3::IRGC 116965-1 | LL_S | WS | 0 | 70.06 | 19.88 | 34.83 | 23 | 3442.73 | 594.06 | 0.16 | 220.00 | 61.35 |
| RP082 | CEMPO MANGGAR::IRGC 27107-1 | LL_S | WS | 0 | 0.00 | 0.00 | 0.00 | 0 | 0.00 | 0.00 | 0.00 | 0.00 | 0.00 |
| RP083 | CHAKOL::IRGC 77226-1 | LL_S | WS | 0 | 0.00 | 0.00 | 0.00 | 0 | 0.00 | 0.00 | 0.00 | 0.00 | 0.00 |
| RP084 | CHAM LEK::IRGC 89387-1 | LL_S | WS | 0 | 123.61 | 21.38 | 34.32 | 23 | 3531.41 | 578.47 | 0.16 | 130.00 | 56.00 |
| RP085 | CHAMA (DWARF)::IRGC 69487-1 | LL_S | WS | 0 | 66.53 | 18.79 | 30.47 | 45 | 3708.30 | 603.50 | 0.16 | 205.00 | 61.61 |
| RP086 | CHANDARHAT::IRGC 25845-1 | LL_S | WS | 0 | 110.06 | 19.71 | 41.43 | 23 | 3670.77 | 589.86 | 0.16 | 205.00 | 61.55 |
| RP087 | CHANDINA::IRGC 36420-1 | LL_S | WS | 0 | 54.05 | 17.87 | 26.88 | 8 | 3036.83 | 571.61 | 0.16 | 185.00 | 61.79 |
| RP088 | CHANG LE SAN SHU ZAO::IRGC 63561-1 | LL_S | WS | 79 | 70.39 | 18.75 | 34.47 | 26 | 3445.74 | 636.45 | 0.16 | 190.00 | 62.85 |
| **Study designation** | **3KG database designation** | **Env** | **Season** | **DTF** | **PH (cm)** | **PL (cm)** | **FlgLA (cm^2^)** | **NBP** | **BMDW (kgha^-1^)** | **GY (kgha^-1^)** | **HI** | **TGW (g)** | **SPKFT (%)** |
| RP089 | CHAO PEUAK DENG::IRGC 11602-1 | LL_S | WS | 0 | 127.65 | 21.34 | 40.16 | 20 | 3520.00 | 584.42 | 0.16 | 140.00 | 60.73 |
| RP090 | CHI SHENG TAO::IRGC 4606-1 | LL_S | WS | 80 | 105.93 | 19.79 | 36.59 | 25 | 3211.95 | 576.99 | 0.16 | 105.00 | 53.96 |
| RP091 | CHIAYI WU-K'O::IRGC 64974-1 | LL_S | WS | 0 | 124.86 | 19.82 | 39.47 | 13 | 3583.73 | 575.84 | 0.16 | 215.00 | 60.74 |
| RP092 | CHIH SHEN LI::IRGC 1306-1 | LL_S | WS | 86 | 136.29 | 21.88 | 40.27 | 6 | 4316.30 | 568.24 | 0.16 | 35.00 | 46.62 |
| RP093 | CHNNOR::IRGC 67485-1 | LL_S | WS | 86 | 121.12 | 23.45 | 53.86 | 32 | 3263.35 | 598.23 | 0.16 | 190.00 | 62.63 |
| RP094 | CHUA DAU::IRGC 4785-1 | LL_S | WS | 78 | 135.46 | 21.37 | 42.25 | 33 | 3868.41 | 586.26 | 0.16 | 215.00 | 60.34 |
| RP095 | CHUNGUR BALI::IRGC 25855-1 | LL_S | WS | 70 | 126.64 | 22.34 | 38.94 | 40 | 3800.82 | 591.93 | 0.16 | 195.00 | 58.33 |
| RP096 | CICA 9::IRGC 53079-1 | LL_S | WS | 0 | 0.00 | 0.00 | 0.00 | 0 | 0.00 | 0.00 | 0.00 | 0.00 | 0.00 |
| RP097 | CN 44-40-7::IRGC 45368-1 | LL_S | WS | 83 | 73.84 | 18.10 | 31.31 | 34 | 3692.10 | 632.33 | 0.16 | 180.00 | 61.95 |
| RP098 | CO 39::IRGC 51231-1 | LL_S | WS | 72 | 71.74 | 17.83 | 26.53 | 24 | 3408.59 | 574.33 | 0.16 | 180.00 | 59.41 |
| RP099 | CR 60-10::IRGC 15777-1 | LL_S | WS | 0 | 63.73 | 18.30 | 33.20 | 33 | 2827.40 | 588.11 | 0.16 | 180.00 | 61.97 |
| RP100 | CRILLO LA FRIA::IRGC 10793-1 | LL_S | WS | 78 | 110.25 | 20.46 | 42.92 | 21 | 3206.89 | 595.81 | 0.16 | 125.00 | 61.85 |
| RP101 | CSR-90 IR-2::IRGC 117327-1 | LL_S | WS | 96 | 88.46 | 18.19 | 35.45 | 16 | 3236.57 | 580.30 | 0.16 | 160.00 | 57.23 |
| RP102 | CT 9737-6-1-1-2-2P-M::IRGC 117330-1 | LL_S | WS | 91 | 66.92 | 19.49 | 31.04 | 23 | 3279.48 | 586.74 | 0.16 | 160.00 | 55.41 |
| RP103 | CUN GU NUO::IRGC 63576-1 | LL_S | WS | 0 | 80.22 | 20.11 | 49.51 | 24 | 3683.72 | 579.02 | 0.16 | 160.00 | 61.07 |
| RP104 | CUYAMEL 3820::IRGC 116975-1 | LL_S | WS | 0 | 0.00 | 0.00 | 0.00 | 0 | 0.00 | 0.00 | 0.00 | 0.00 | 0.00 |
| RP105 | DA 11::IRGC 6046-1 | LL_S | WS | 0 | 0.00 | 0.00 | 0.00 | 0 | 0.00 | 0.00 | 0.00 | 0.00 | 0.00 |
| RP106 | DA GANG ZHAN::IRGC 67103-1 | LL_S | WS | 87 | 124.78 | 19.54 | 39.79 | 23 | 3535.31 | 593.15 | 0.16 | 215.00 | 61.28 |
| RP107 | DA NUO (ZHAN)::IRGC 72025-1 | LL_S | WS | 80 | 118.19 | 20.07 | 38.26 | 11 | 3428.45 | 573.42 | 0.16 | 90.00 | 53.12 |
| RP108 | DAA MANSA::IRGC 67559-1 | LL_S | WS | 0 | 82.91 | 16.30 | 33.70 | 20 | 3448.92 | 584.71 | 0.16 | 200.00 | 63.41 |
| RP109 | DANGAR::IRGC 76296-1 | LL_S | WS | 79 | 119.83 | 21.23 | 39.82 | 29 | 3711.51 | 591.68 | 0.16 | 210.00 | 59.04 |
| RP110 | DHANE BURWA::IRGC 10105-1 | LL_S | WS | 0 | 95.31 | 20.42 | 44.49 | 2 | 3539.82 | 568.21 | 0.16 | 15.00 | 55.82 |
| RP111 | DISSI::IRGC 101346-1 | LL_S | WS | 0 | 125.10 | 20.09 | 60.43 | 35 | 3736.00 | 606.04 | 0.16 | 200.00 | 55.68 |
| RP112 | DUDH KADAR::IRGC 67707-1 | LL_S | WS | 78 | 128.96 | 21.69 | 31.51 | 0 | 2950.28 | 567.92 | 0.16 | 10.00 | 46.63 |
| RP113 | E 2024::IRGC 67958-1 | LL_S | WS | 0 | 55.48 | 15.38 | 23.77 | 30 | 3357.52 | 571.98 | 0.16 | 70.00 | 52.96 |
| RP114 | E 2040::IRGC 67968-1 | LL_S | WS | 72 | 67.11 | 18.59 | 30.06 | 42 | 3263.74 | 609.75 | 0.16 | 170.00 | 63.85 |
| RP115 | E DAW HAWM::IRGC 47938-1 | LL_S | WS | 0 | 0.00 | 0.00 | 0.00 | 0 | 0.00 | 0.00 | 0.00 | 0.00 | 0.00 |
| RP116 | ELONI::IRGC 116980-1 | LL_S | WS | 0 | 68.52 | 18.28 | 31.87 | 26 | 3490.52 | 585.05 | 0.16 | 185.00 | 56.86 |
| **Study designation** | **3KG database designation** | **Env** | **Season** | **DTF** | **PH (cm)** | **PL (cm)** | **FlgLA (cm^2^)** | **NBP** | **BMDW (kgha^-1^)** | **GY (kgha^-1^)** | **HI** | **TGW (g)** | **SPKFT (%)** |
| RP117 | EPEAL 102::IRGC 78698-1 | LL_S | WS | 0 | 75.33 | 15.51 | 42.71 | 20 | 3231.67 | 584.28 | 0.16 | 165.00 | 55.90 |
| RP118 | ES 21::IRGC 56171-1 | LL_S | WS | 0 | 0.00 | 0.00 | 0.00 | 0 | 0.00 | 0.00 | 0.00 | 0.00 | 0.00 |
| RP119 | EX FOILAEIN (NAPUTO)::IRGC 81675-1 | LL_S | WS | 0 | 88.78 | 19.78 | 32.03 | 8 | 4086.78 | 571.02 | 0.16 | 125.00 | 60.18 |
| RP120 | FEI GAI 122::IRGC 63599-1 | LL_S | WS | 0 | 72.22 | 16.43 | 43.01 | 16 | 3358.34 | 583.37 | 0.16 | 100.00 | 55.48 |
| RP121 | FONAIAP 2::IRGC 116985-1 | LL_S | WS | 0 | 75.05 | 18.69 | 34.22 | 27 | 4415.33 | 645.63 | 0.16 | 240.00 | 64.32 |
| RP122 | FU ZAO XIAN::IRGC 63619-1 | LL_S | WS | 0 | 65.27 | 19.24 | 29.77 | 19 | 3228.06 | 593.89 | 0.16 | 220.00 | 64.97 |
| RP123 | G 25::IRGC 45733-1 | LL_S | WS | 0 | 0.00 | 0.00 | 0.00 | 0 | 0.00 | 0.00 | 0.00 | 0.00 | 0.00 |
| RP124 | GAO JIAO BAI::IRGC 68047-1 | LL_S | WS | 67 | 85.90 | 19.42 | 27.41 | 25 | 3479.53 | 599.98 | 0.16 | 150.00 | 61.41 |
| RP125 | GENIT::IRGC 3272-1 | LL_S | WS | 0 | 110.97 | 17.07 | 31.03 | 18 | 2878.19 | 576.48 | 0.16 | 105.00 | 56.47 |
| RP126 | GOJOL GORIA::IRGC 26629-1 | LL_S | WS | 0 | 0.00 | 0.00 | 0.00 | 0 | 0.00 | 0.00 | 0.00 | 0.00 | 0.00 |
| RP127 | GOKULGANJA::IRGC 45701-1 | LL_S | WS | 0 | 0.00 | 0.00 | 0.00 | 0 | 0.00 | 0.00 | 0.00 | 0.00 | 0.00 |
| RP128 | GUI HUA ZAO::IRGC 68060-1 | LL_S | WS | 74 | 112.86 | 20.60 | 34.74 | 30 | 3706.95 | 597.23 | 0.16 | 135.00 | 55.69 |
| RP130 | HAWM KRUA::IRGC 64333-1 | LL_S | WS | 88 | 141.47 | 23.41 | 47.08 | 17 | 3551.04 | 595.40 | 0.16 | 120.00 | 55.41 |
| RP131 | HD 10::IRGC 6638-1 | LL_S | WS | 0 | 150.82 | 22.78 | 41.43 | 29 | 3679.12 | 619.30 | 0.16 | 200.00 | 62.68 |
| RP132 | HE GU TSAO::IRGC 51302-1 | LL_S | WS | 73 | 116.66 | 23.74 | 38.04 | 16 | 3676.88 | 575.67 | 0.16 | 190.00 | 54.00 |
| RP133 | HODARAWALA::IRGC 67631-1 | LL_S | WS | 86 | 113.37 | 22.94 | 41.32 | 12 | 3383.63 | 571.74 | 0.16 | 125.00 | 57.27 |
| RP134 | HOLOI BASH (SOLOI BASH)::IRGC 64778-1 | LL_S | WS | 87 | 109.41 | 19.45 | 34.34 | 35 | 3390.91 | 597.17 | 0.16 | 165.00 | 54.67 |
| RP135 | HTA 22::IRGC 45827-1 | LL_S | WS | 0 | 149.95 | 21.64 | 39.93 | 21 | 4075.88 | 592.40 | 0.16 | 100.00 | 58.49 |
| RP136 | HUA LI ZAO::IRGC 80950-1 | LL_S | WS | 0 | 0.00 | 0.00 | 0.00 | 0 | 0.00 | 0.00 | 0.00 | 0.00 | 0.00 |
| RP137 | I KUNG PAO::IRGC 114-1 | LL_S | WS | 86 | 114.93 | 20.30 | 55.84 | 14 | 3556.79 | 569.65 | 0.16 | 65.00 | 53.45 |
| RP138 | IA CUBA 17::IRGC 116990-1 | LL_S | WS | 0 | 81.97 | 19.67 | 42.84 | 36 | 4285.98 | 609.83 | 0.16 | 240.00 | 54.64 |
| RP139 | ICTA CRISPO 38::IRGC 116994-1 | LL_S | WS | 0 | 67.97 | 17.64 | 38.22 | 6 | 3412.16 | 573.65 | 0.16 | 110.00 | 53.40 |
| RP140 | ICTA MOTAGUA::IRGC 116995-1 | LL_S | WS | 0 | 71.51 | 17.16 | 31.43 | 11 | 3855.53 | 582.26 | 0.16 | 95.00 | 61.27 |
| RP141 | INIAP 6::IRGC 117002-1 | LL_S | WS | 0 | 75.30 | 16.82 | 37.47 | 22 | 3120.45 | 588.13 | 0.16 | 145.00 | 63.94 |
| RP142 | IR 13429-109-2-2-1::IRGC 63491-1 | LL_S | WS | 0 | 70.50 | 19.46 | 27.82 | 15 | 3566.96 | 573.22 | 0.16 | 105.00 | 52.55 |
| RP143 | IR 21015-72-3-3-3-1::IRGC 117004-1 | LL_S | WS | 0 | 69.50 | 17.73 | 32.00 | 9 | 3532.58 | 573.90 | 0.16 | 130.00 | 63.34 |
| RP144 | IR 2344-P1 PB-9-3-2B::IRGC 39317-C1 | LL_S | WS | 68 | 112.99 | 19.45 | 26.81 | 19 | 4271.97 | 574.85 | 0.16 | 120.00 | 61.21 |
| RP146 | IR 63295-AC 209-7::IRGC 117365-1 | LL_S | WS | 0 | 77.72 | 17.15 | 32.15 | 21 | 3113.37 | 611.59 | 0.16 | 195.00 | 61.69 |
| **Study designation** | **3KG database designation** | **Env** | **Season** | **DTF** | **PH (cm)** | **PL (cm)** | **FlgLA (cm^2^)** | **NBP** | **BMDW (kgha^-1^)** | **GY (kgha^-1^)** | **HI** | **TGW (g)** | **SPKFT (%)** |
| RP147 | IR 69502-6-SRN-3-UBN-1-B::IRGC 117290-1 | LL_S | WS | 88 | 86.19 | 17.72 | 33.73 | 28 | 3411.36 | 610.44 | 0.16 | 210.00 | 62.95 |
| RP148 | IR 75870-5-8-5-B-1::IRGC 117297-1 | LL_S | WS | 0 | 73.05 | 19.78 | 32.43 | 7 | 3522.40 | 581.42 | 0.16 | 105.00 | 58.99 |
| RP149 | IR 77390-1-6-4-19-1-B::IRGC 117303-1 | LL_S | WS | 0 | 0.00 | 0.00 | 0.00 | 0 | 0.00 | 0.00 | 0.00 | 0.00 | 0.00 |
| RP150 | IR 80310-12-B-1-3-B::IRGC 117307-1 | LL_S | WS | 0 | 104.52 | 23.06 | 46.36 | 29 | 3173.44 | 613.09 | 0.16 | 140.00 | 63.66 |
| RP151 | IR 80340-23-B-12-6-B::IRGC 117309-1 | LL_S | WS | 87 | 87.58 | 18.46 | 33.13 | 26 | 3363.81 | 607.30 | 0.16 | 205.00 | 61.85 |
| RP152 | IRGA 318-11-6-9-2B::IRGC 117339-1 | LL_S | WS | 0 | 67.45 | 18.23 | 32.05 | 1 | 3223.23 | 567.78 | 0.16 | 10.00 | 46.61 |
| RP153 | IRGA 370-38-1-1F-C4-2::IRGC 117342-1 | LL_S | WS | 0 | 71.04 | 17.32 | 38.72 | 48 | 3112.94 | 608.23 | 0.16 | 190.00 | 64.47 |
| RP154 | IRGA 370-42-1-1F-C-1::IRGC 117343-1 | LL_S | WS | 81 | 72.04 | 21.35 | 38.47 | 7 | 3559.00 | 572.70 | 0.16 | 115.00 | 65.71 |
| RP155 | IRGA 659-1-2-2-2::IRGC 117345-1 | LL_S | WS | 0 | 0.00 | 0.00 | 0.00 | 0 | 0.00 | 0.00 | 0.00 | 0.00 | 0.00 |
| RP156 | IRGA 959-1-2-2F-4-1-4A-6-CA-6X::IRGC 117006-1 | LL_S | WS | 0 | 64.22 | 18.14 | 25.53 | 17 | 2844.25 | 573.17 | 0.16 | 180.00 | 56.23 |
| RP157 | IRI 339::IRGC 46956-1 | LL_S | WS | 0 | 63.05 | 15.07 | 30.47 | 24 | 2915.49 | 587.05 | 0.16 | 185.00 | 62.37 |
| RP158 | IRRIBINI::IRGC 49094-1 | LL_S | WS | 0 | 0.00 | 0.00 | 0.00 | 0 | 0.00 | 0.00 | 0.00 | 0.00 | 0.00 |
| RP159 | JABOR SAIL::IRGC 66831-1 | LL_S | WS | 77 | 126.38 | 20.83 | 52.23 | 29 | 3430.39 | 620.21 | 0.16 | 225.00 | 61.45 |
| RP160 | JAGLI BORO::IRGC 27516-2 | LL_S | WS | 76 | 128.74 | 19.25 | 35.36 | 2 | 4111.72 | 568.11 | 0.16 | 60.00 | 63.67 |
| RP161 | JAMBALI::IRGC 73101-1 | LL_S | WS | 76 | 119.49 | 20.91 | 51.41 | 14 | 3619.42 | 573.87 | 0.16 | 140.00 | 58.10 |
| RP162 | JAO LEUANG::IRGC 65866-1 | LL_S | WS | 0 | 90.83 | 16.76 | 31.31 | 4 | 3818.86 | 582.22 | 0.16 | 200.00 | 52.28 |
| RP164 | JHODI BIRUN::IRGC 31812-1 | LL_S | WS | 0 | 68.92 | 15.97 | 34.70 | 32 | 4168.47 | 598.85 | 0.16 | 160.00 | 63.24 |
| RP165 | JIN JUN DAO::IRGC 59710-1 | LL_S | WS | 70 | 106.96 | 22.22 | 31.86 | 2 | 3652.39 | 568.47 | 0.16 | 45.00 | 46.62 |
| RP166 | JUMA 62::IRGC 117011-1 | LL_S | WS | 0 | 0.00 | 0.00 | 0.00 | 0 | 0.00 | 0.00 | 0.00 | 0.00 | 0.00 |
| RP167 | KALABAIL::IRGC 25877-1 | LL_S | WS | 0 | 0.00 | 0.00 | 0.00 | 0 | 0.00 | 0.00 | 0.00 | 0.00 | 0.00 |
| RP168 | KALIA::IRGC 34699-1 | LL_S | WS | 82 | 106.06 | 21.84 | 48.57 | 23 | 3366.97 | 599.88 | 0.16 | 135.00 | 55.86 |
| RP169 | KALO CHAKOL::IRGC 77258-1 | LL_S | WS | 0 | 0.00 | 0.00 | 0.00 | 0 | 0.00 | 0.00 | 0.00 | 0.00 | 0.00 |
| RP170 | KALU ILANKALAYAN::IRGC 36270-1 | LL_S | WS | 0 | 130.46 | 20.87 | 46.69 | 12 | 4060.45 | 570.18 | 0.16 | 85.00 | 54.05 |
| RP171 | KAM PAI::IRGC 78245-1 | LL_S | WS | 0 | 0.00 | 0.00 | 0.00 | 0 | 0.00 | 0.00 | 0.00 | 0.00 | 0.00 |
| RP172 | KEERIPALA CHILL PADDY::IRGC 49790-1 | LL_S | WS | 76 | 122.23 | 19.72 | 51.37 | 42 | 3726.33 | 610.38 | 0.16 | 205.00 | 63.70 |
| RP173 | KETAN SERANG::IRGC 14615-1 | LL_S | WS | 0 | 107.38 | 16.81 | 40.06 | 39 | 3248.69 | 612.62 | 0.16 | 220.00 | 54.91 |
| RP174 | KHAO DAWK MALI 105::IRGC 27748-2 | LL_S | WS | 0 | 106.54 | 20.35 | 38.99 | 15 | 3744.20 | 570.44 | 0.16 | 50.00 | 57.83 |
| **Study designation** | **3KG database designation** | **Env** | **Season** | **DTF** | **PH (cm)** | **PL (cm)** | **FlgLA (cm^2^)** | **NBP** | **BMDW (kgha^-1^)** | **GY (kgha^-1^)** | **HI** | **TGW (g)** | **SPKFT (%)** |
| RP175 | KHAO GRADOOK CHAHNG::IRGC 17111-1 | LL_S | WS | 0 | 79.21 | 20.06 | 34.10 | 13 | 4597.00 | 570.27 | 0.16 | 115.00 | 53.41 |
| RP176 | KHAO' HAWM::IRGC 78257-1 | LL_S | WS | 0 | 0.00 | 0.00 | 0.00 | 0 | 0.00 | 0.00 | 0.00 | 0.00 | 0.00 |
| RP177 | KHAO THI RATE::IRGC 58041-1 | LL_S | WS | 0 | 100.21 | 21.36 | 40.68 | 18 | 3373.85 | 586.75 | 0.16 | 150.00 | 60.17 |
| RP178 | KHARSU 80::IRGC 28016-1 | LL_S | WS | 74 | 77.95 | 18.58 | 39.25 | 38 | 3291.90 | 621.39 | 0.16 | 220.00 | 64.22 |
| RP179 | KITRANA 1007::IRGC 68517-1 | LL_S | WS | 0 | 0.00 | 0.00 | 0.00 | 0 | 0.00 | 0.00 | 0.00 | 0.00 | 0.00 |
| RP180 | KN 1 B 361-1-8-6-9::IRGC 46974-1 | LL_S | WS | 85 | 110.86 | 20.09 | 35.68 | 22 | 3443.83 | 587.68 | 0.16 | 190.00 | 54.47 |
| RP181 | KORASISI::IRGC 5285-1 | LL_S | WS | 0 | 144.10 | 22.86 | 46.79 | 8 | 3099.28 | 568.19 | 0.16 | 40.00 | 46.63 |
| RP182 | KOTTEYARAN::IRGC 47383-1 | LL_S | WS | 77 | 129.13 | 21.23 | 55.86 | 22 | 4222.70 | 592.09 | 0.16 | 95.00 | 60.69 |
| RP183 | KOYRA::IRGC 77267-1 | LL_S | WS | 82 | 130.08 | 18.86 | 42.63 | 11 | 4687.05 | 571.47 | 0.16 | 120.00 | 54.46 |
| RP184 | KULA KARUPPAN::IRGC 55328-1 | LL_S | WS | 0 | 122.95 | 21.31 | 53.72 | 35 | 3558.24 | 589.10 | 0.16 | 195.00 | 62.12 |
| RP185 | KUNENG::IRGC 71545-1 | LL_S | WS | 0 | 0.00 | 0.00 | 0.00 | 0 | 0.00 | 0.00 | 0.00 | 0.00 | 0.00 |
| RP186 | KURULU WEE (WHITE)::IRGC 66518-1 | LL_S | WS | 85 | 119.72 | 21.54 | 45.75 | 15 | 3329.87 | 571.23 | 0.16 | 125.00 | 51.50 |
| RP188 | KUSHIARA::IRGC 34709-1 | LL_S | WS | 0 | 0.00 | 0.00 | 0.00 | 0 | 0.00 | 0.00 | 0.00 | 0.00 | 0.00 |
| RP189 | KUTTA::IRGC 52184-1 | LL_S | WS | 0 | 0.00 | 0.00 | 0.00 | 0 | 0.00 | 0.00 | 0.00 | 0.00 | 0.00 |
| RP190 | LABRA::IRGC 74757-1 | LL_S | WS | 0 | 109.56 | 16.31 | 47.82 | 32 | 3735.19 | 589.00 | 0.16 | 220.00 | 60.52 |
| RP191 | LAI YIP ZIM::IRGC 4955-1 | LL_S | WS | 86 | 125.41 | 20.62 | 37.84 | 26 | 3330.58 | 585.16 | 0.16 | 235.00 | 61.47 |
| RP192 | LALKA (LAL DHAN)::IRGC 64946-1 | LL_S | WS | 0 | 0.00 | 0.00 | 0.00 | 0 | 0.00 | 0.00 | 0.00 | 0.00 | 0.00 |
| RP193 | LALSAITA::IRGC 43915-1 | LL_S | WS | 77 | 114.43 | 19.22 | 44.64 | 18 | 3716.74 | 589.64 | 0.16 | 110.00 | 53.14 |
| RP194 | LARHA MUGAD::IRGC 52339-1 | LL_S | WS | 76 | 102.19 | 20.77 | 34.24 | 23 | 3221.42 | 586.46 | 0.16 | 95.00 | 54.60 |
| RP196 | LENJA MURALI::IRGC 66815-1 | LL_S | WS | 79 | 118.03 | 20.34 | 41.67 | 33 | 3525.43 | 614.00 | 0.16 | 245.00 | 62.87 |
| RP197 | LEUANG YAI 29-12-2::IRGC 881-1 | LL_S | WS | 0 | 70.59 | 15.08 | 41.10 | 11 | 0.00 | 580.13 | 0.16 | 210.00 | 58.81 |
| RP198 | LU MAO ZHAN::IRGC 68159-1 | LL_S | WS | 67 | 123.55 | 21.29 | 38.26 | 21 | 3400.28 | 582.48 | 0.16 | 85.00 | 59.33 |
| RP199 | LUA CHAN HUONG::IRGC 16800-1 | LL_S | WS | 0 | 0.00 | 0.00 | 0.00 | 0 | 0.00 | 0.00 | 0.00 | 0.00 | 0.00 |
| RP200 | MAKALIOKA::IRGC 376-1 | LL_S | WS | 0 | 127.41 | 23.13 | 38.80 | 3 | 3387.16 | 568.65 | 0.16 | 105.00 | 46.64 |
| RP201 | MAKRO::IRGC 74763-1 | LL_S | WS | 0 | 115.89 | 20.79 | 45.00 | 5 | 3508.86 | 571.38 | 0.16 | 105.00 | 57.43 |
| RP202 | MAMORIAKA::IRGC 68672-1 | LL_S | WS | 0 | 106.39 | 21.29 | 40.58 | 20 | 3088.65 | 575.93 | 0.16 | 195.00 | 56.13 |
| RP203 | MEI FENG 9::IRGC 63735-1 | LL_S | WS | 78 | 67.17 | 17.83 | 31.89 | 17 | 3262.25 | 577.56 | 0.16 | 145.00 | 58.87 |
| RP204 | MEKENZIE SMALL::IRGC 49895-1 | LL_S | WS | 0 | 70.39 | 20.98 | 32.53 | 27 | 3033.13 | 597.82 | 0.16 | 205.00 | 59.85 |
| **Study designation** | **3KG database designation** | **Env** | **Season** | **DTF** | **PH (cm)** | **PL (cm)** | **FlgLA (cm^2^)** | **NBP** | **BMDW (kgha^-1^)** | **GY (kgha^-1^)** | **HI** | **TGW (g)** | **SPKFT (%)** |
| RP205 | MELEKE::IRGC 56823-1 | LL_S | WS | 0 | 77.61 | 23.40 | 47.20 | 18 | 3614.90 | 600.99 | 0.16 | 185.00 | 63.49 |
| RP206 | MENTIK TJERE BELUT::IRGC 18254-1 | LL_S | WS | 0 | 0.00 | 0.00 | 0.00 | 0 | 0.00 | 0.00 | 0.00 | 0.00 | 0.00 |
| RP207 | MILYANG 30::IRGC 46977-1 | LL_S | WS | 0 | 58.85 | 16.16 | 31.54 | 30 | 2969.13 | 610.70 | 0.16 | 220.00 | 59.52 |
| RP208 | MILYANG 77::IRGC 69340-1 | LL_S | WS | 74 | 70.21 | 15.98 | 26.55 | 17 | 3741.27 | 573.15 | 0.16 | 95.00 | 60.44 |
| RP209 | MIN KE ZHAN::IRGC 72230-1 | LL_S | WS | 75 | 82.78 | 21.51 | 30.08 | 22 | 3815.90 | 575.79 | 0.16 | 170.00 | 58.72 |
| RP210 | MIN ZAO 6::IRGC 63772-1 | LL_S | WS | 77 | 78.97 | 18.46 | 33.12 | 18 | 2876.11 | 614.15 | 0.16 | 125.00 | 65.20 |
| RP211 | MODDAI KARUPPAN::IRGC 15465-1 | LL_S | WS | 0 | 95.83 | 20.69 | 36.97 | 10 | 3603.81 | 582.34 | 0.16 | 200.00 | 57.92 |
| RP212 | MUKKALA BAZAL::IRGC 77279-1 | LL_S | WS | 0 | 0.00 | 0.00 | 0.00 | 0 | 0.00 | 0.00 | 0.00 | 0.00 | 0.00 |
| RP213 | MULLIKURUVA::IRGC 77529-1 | LL_S | WS | 0 | 136.83 | 22.76 | 36.82 | 10 | 3648.60 | 570.18 | 0.16 | 55.00 | 62.79 |
| RP214 | MUTA GANJE::IRGC 26744-1 | LL_S | WS | 0 | 0.00 | 0.00 | 0.00 | 0 | 0.00 | 0.00 | 0.00 | 0.00 | 0.00 |
| RP215 | MUTTU SAMBA::IRGC 36333-1 | LL_S | WS | 0 | 123.16 | 21.22 | 38.72 | 6 | 3556.09 | 568.09 | 0.16 | 70.00 | 46.60 |
| RP216 | N 22::IRGC 46459-1 | LL_S | WS | 78 | 111.43 | 20.92 | 55.21 | 36 | 3877.23 | 593.37 | 0.16 | 185.00 | 62.08 |
| RP217 | Na souan::IRGC 11889-1 | LL_S | WS | 0 | 114.44 | 18.85 | 36.25 | 3 | 2958.96 | 570.88 | 0.16 | 190.00 | 49.07 |
| RP218 | NAZIRA SAIL::IRGC 77284-1 | LL_S | WS | 0 | 0.00 | 0.00 | 0.00 | 0 | 0.00 | 0.00 | 0.00 | 0.00 | 0.00 |
| RP219 | NCS 194::IRGC 51932-1 | LL_S | WS | 85 | 113.45 | 19.57 | 42.58 | 22 | 4008.14 | 596.94 | 0.16 | 95.00 | 53.86 |
| RP220 | NCS 237::IRGC 62202-1 | LL_S | WS | 85 | 130.10 | 24.04 | 48.88 | 12 | 3769.40 | 577.71 | 0.16 | 125.00 | 53.87 |
| RP222 | NCS 964 C::IRGC 62604-1 | LL_S | WS | 0 | 0.00 | 0.00 | 0.00 | 0 | 0.00 | 0.00 | 0.00 | 0.00 | 0.00 |
| RP223 | NIAO YAO::IRGC 5496-1 | LL_S | WS | 0 | 123.05 | 22.15 | 36.27 | 12 | 3334.33 | 580.17 | 0.16 | 125.00 | 62.26 |
| RP224 | NIBARI::IRGC 67742-1 | LL_S | WS | 78 | 126.86 | 21.42 | 46.03 | 24 | 4006.13 | 584.80 | 0.16 | 170.00 | 59.43 |
| RP225 | NONA BOKRA::IRGC 22710-C1 | LL_S | WS | 0 | 117.50 | 21.61 | 39.42 | 20 | 4026.34 | 583.77 | 0.16 | 190.00 | 58.84 |
| RP226 | NOROI::IRGC 31611-1 | LL_S | WS | 73 | 105.72 | 23.13 | 43.39 | 9 | 3957.37 | 572.96 | 0.16 | 95.00 | 53.68 |
| RP228 | NX 3533::IRGC 63796-1 | LL_S | WS | 0 | 74.10 | 18.01 | 36.29 | 15 | 3454.31 | 584.37 | 0.16 | 130.00 | 53.60 |
| RP229 | O. SATIVA::IRGC 17083-1 | LL_S | WS | 85 | 138.44 | 23.15 | 41.17 | 30 | 3860.86 | 593.31 | 0.16 | 180.00 | 61.82 |
| RP230 | PAH WEAN::IRGC 78276-1 | LL_S | WS | 0 | 0.00 | 0.00 | 0.00 | 0 | 0.00 | 0.00 | 0.00 | 0.00 | 0.00 |
| RP231 | PAI CHUEH CHIU LIU::IRGC 34259-1 | LL_S | WS | 84 | 77.03 | 18.46 | 43.96 | 30 | 4032.81 | 574.76 | 0.16 | 180.00 | 58.71 |
| RP232 | PAI YI PING::IRGC 1368-1 | LL_S | WS | 77 | 105.29 | 17.81 | 36.31 | 22 | 3279.15 | 584.27 | 0.16 | 200.00 | 59.77 |
| RP233 | PALEPYU::IRGC 33549-1 | LL_S | WS | 69 | 108.33 | 21.15 | 42.36 | 42 | 4414.97 | 601.68 | 0.16 | 145.00 | 59.71 |
| RP234 | PARA NELLU::IRGC 50009-1 | LL_S | WS | 75 | 137.50 | 22.24 | 29.54 | 30 | 3556.31 | 582.33 | 0.16 | 215.00 | 58.94 |
| **Study designation** | **3KG database designation** | **Env** | **Season** | **DTF** | **PH (cm)** | **PL (cm)** | **FlgLA (cm^2^)** | **NBP** | **BMDW (kgha^-1^)** | **GY (kgha^-1^)** | **HI** | **TGW (g)** | **SPKFT (%)** |
| RP235 | PATISAIL::IRGC 37562-1 | LL_S | WS | 0 | 0.00 | 0.00 | 0.00 | 0 | 0.00 | 0.00 | 0.00 | 0.00 | 0.00 |
| RP237 | PERUNEL::IRGC 63113-1 | LL_S | WS | 81 | 133.41 | 22.80 | 41.16 | 23 | 4348.54 | 588.07 | 0.16 | 145.00 | 64.08 |
| RP238 | PICO NEGRO::IRGC 55849-1 | LL_S | WS | 0 | 0.00 | 0.00 | 0.00 | 0 | 0.00 | 0.00 | 0.00 | 0.00 | 0.00 |
| RP239 | PLI KHAO::IRGC 64596-1 | LL_S | WS | 0 | 0.00 | 0.00 | 0.00 | 0 | 0.00 | 0.00 | 0.00 | 0.00 | 0.00 |
| RP240 | PODI HEENATI::IRGC 36345-1 | LL_S | WS | 0 | 95.03 | 17.11 | 36.14 | 10 | 4361.06 | 568.09 | 0.16 | 10.00 | 48.67 |
| RP241 | PSBRC 50::IRGC 99706-1 | LL_S | WS | 0 | 88.40 | 20.25 | 31.06 | 27 | 3172.25 | 597.23 | 0.16 | 115.00 | 60.35 |
| RP242 | PSBRC 68::IRGC 99711-1 | LL_S | WS | 0 | 101.23 | 21.90 | 47.51 | 39 | 4266.94 | 600.07 | 0.16 | 200.00 | 62.97 |
| RP243 | PSBRC 88::IRGC 99717-1 | LL_S | WS | 0 | 0.00 | 0.00 | 0.00 | 0 | 0.00 | 0.00 | 0.00 | 0.00 | 0.00 |
| RP244 | PULUT BARAYA::IRGC 27393-1 | LL_S | WS | 0 | 0.00 | 0.00 | 0.00 | 0 | 0.00 | 0.00 | 0.00 | 0.00 | 0.00 |
| RP245 | PURA BINNI::IRGC 26772-1 | LL_S | WS | 0 | 0.00 | 0.00 | 0.00 | 0 | 0.00 | 0.00 | 0.00 | 0.00 | 0.00 |
| RP247 | QUERO ASSAN::IRGC 28860-1 | LL_S | WS | 0 | 0.00 | 0.00 | 0.00 | 0 | 0.00 | 0.00 | 0.00 | 0.00 | 0.00 |
| RP248 | RACE PERUMAL::IRGC 55347-1 | LL_S | WS | 73 | 108.95 | 19.32 | 29.55 | 27 | 3236.54 | 578.90 | 0.16 | 175.00 | 59.82 |
| RP250 | RELLY::IRGC 14623-1 | LL_S | WS | 0 | 0.00 | 0.00 | 0.00 | 0 | 0.00 | 0.00 | 0.00 | 0.00 | 0.00 |
| RP251 | RIZ TYPE SORGHO::IRGC 69015-1 | LL_S | WS | 0 | 79.30 | 19.20 | 35.06 | 32 | 3652.02 | 603.30 | 0.16 | 240.00 | 61.41 |
| RP252 | ROJOFOTSY::IRGC 69402-1 | LL_S | WS | 0 | 0.00 | 0.00 | 0.00 | 0 | 0.00 | 0.00 | 0.00 | 0.00 | 0.00 |
| RP253 | RPA 5929 (K 45)::IRGC 33963-1 | LL_S | WS | 0 | 60.13 | 16.77 | 38.48 | 2 | 3326.85 | 577.72 | 0.16 | 40.00 | 51.95 |
| RP254 | RPW 9-4 (SS 1)::IRGC 50690-1 | LL_S | WS | 0 | 81.78 | 19.06 | 33.33 | 16 | 2819.86 | 572.51 | 0.16 | 120.00 | 62.95 |
| RP255 | RR 272-17-829::IRGC 117354-1 | LL_S | WS | 0 | 93.47 | 19.65 | 46.19 | 27 | 2984.82 | 605.76 | 0.16 | 190.00 | 62.01 |
| RP256 | RTS 16::IRGC 8235-1 | LL_S | WS | 0 | 120.18 | 19.82 | 44.54 | 22 | 3321.44 | 584.37 | 0.16 | 195.00 | 60.45 |
| RP257 | RUSTIC::IRGC 117026-1 | LL_S | WS | 0 | 61.77 | 15.72 | 26.36 | 21 | 2967.65 | 576.05 | 0.16 | 145.00 | 57.01 |
| RP258 | SADA RUPA::IRGC 77299-1 | LL_S | WS | 0 | 80.01 | 17.15 | 38.74 | 29 | 3545.50 | 593.05 | 0.16 | 205.00 | 60.50 |
| RP259 | SAN CHIAO TSWEN::IRGC 1565-1 | LL_S | WS | 0 | 0.00 | 0.00 | 0.00 | 0 | 0.00 | 0.00 | 0.00 | 0.00 | 0.00 |
| RP260 | SAN SHIH TSI::IRGC 1038-1 | LL_S | WS | 87 | 94.45 | 21.82 | 35.12 | 23 | 3183.19 | 594.23 | 0.16 | 180.00 | 59.46 |
| RP261 | SIGARDIS::IRGC 15555-1 | LL_S | WS | 0 | 0.00 | 0.00 | 0.00 | 0 | 0.00 | 0.00 | 0.00 | 0.00 | 0.00 |
| RP262 | SIMET 2::IRGC 25734-1 | LL_S | WS | 0 | 0.00 | 0.00 | 0.00 | 0 | 0.00 | 0.00 | 0.00 | 0.00 | 0.00 |
| RP263 | SIMUL KHURI::IRGC 35154-1 | LL_S | WS | 81 | 117.75 | 20.51 | 42.28 | 17 | 3419.95 | 573.89 | 0.16 | 100.00 | 58.67 |
| RP264 | SINNA SITHIRA KALI::IRGC 51064-1 | LL_S | WS | 0 | 69.10 | 15.97 | 26.53 | 33 | 3001.92 | 587.01 | 0.16 | 185.00 | 55.45 |
| RP266 | SITHAIYAN KOTTAI SAMBA::IRGC 50155-1 | LL_S | WS | 0 | 0.00 | 0.00 | 0.00 | 0 | 0.00 | 0.00 | 0.00 | 0.00 | 0.00 |
| **Study designation** | **3KG database designation** | **Env** | **Season** | **DTF** | **PH (cm)** | **PL (cm)** | **FlgLA (cm^2^)** | **NBP** | **BMDW (kgha^-1^)** | **GY (kgha^-1^)** | **HI** | **TGW (g)** | **SPKFT (%)** |
| RP267 | SML AWINI::IRGC 13391-1 | LL_S | WS | 0 | 77.64 | 17.72 | 27.17 | 16 | 2778.26 | 568.59 | 0.16 | 30.00 | 52.64 |
| RP268 | SOLOMON RED RICE::IRGC 50950-1 | LL_S | WS | 0 | 90.66 | 16.86 | 37.17 | 18 | 3910.23 | 578.47 | 0.16 | 185.00 | 57.67 |
| RP269 | SONA::IRGC 26971-C1 | LL_S | WS | 0 | 74.79 | 19.58 | 38.18 | 1 | 2746.95 | 567.93 | 0.16 | 10.00 | 46.60 |
| RP270 | SONAMUKHI::IRGC 46693-1 | LL_S | WS | 79 | 94.53 | 21.42 | 33.82 | 7 | 3947.60 | 588.69 | 0.16 | 125.00 | 56.77 |
| RP272 | SSANGDUJO::IRGC 55632-1 | LL_S | WS | 87 | 132.57 | 19.69 | 45.24 | 22 | 3647.63 | 571.09 | 0.16 | 165.00 | 54.79 |
| RP273 | SUFAID 246::IRGC 28303-1 | LL_S | WS | 82 | 121.96 | 23.18 | 43.91 | 18 | 3673.70 | 577.04 | 0.16 | 130.00 | 63.87 |
| RP274 | SUWEON 311::IRGC 61890-1 | LL_S | WS | 0 | 64.44 | 15.67 | 35.90 | 31 | 2951.74 | 576.12 | 0.16 | 85.00 | 54.32 |
| RP275 | T 315::IRGC 54792-1 | LL_S | WS | 0 | 92.33 | 20.56 | 35.13 | 18 | 4331.87 | 593.80 | 0.16 | 135.00 | 64.23 |
| RP276 | TAIPEI WOO CO::IRGC 112-1 | LL_S | WS | 0 | 0.00 | 0.00 | 0.00 | 0 | 0.00 | 0.00 | 0.00 | 0.00 | 0.00 |
| RP277 | TAITUNG WOO LI::IRGC 111-1 | LL_S | WS | 0 | 0.00 | 0.00 | 0.00 | 0 | 0.00 | 0.00 | 0.00 | 0.00 | 0.00 |
| RP278 | TAK SUFAID::IRGC 73127-1 | LL_S | WS | 0 | 94.42 | 16.83 | 41.49 | 22 | 3278.50 | 589.36 | 0.16 | 150.00 | 59.91 |
| RP279 | TAK::IRGC 73124-1 | LL_S | WS | 85 | 101.79 | 20.72 | 40.13 | 15 | 2928.55 | 597.87 | 0.16 | 205.00 | 62.38 |
| RP280 | TIKAL 3::IRGC 50649-1 | LL_S | WS | 0 | 77.02 | 19.42 | 42.40 | 9 | 3693.85 | 567.97 | 0.16 | 15.00 | 46.65 |
| RP281 | TOC 5430::IRGC 70487-1 | LL_S | WS | 0 | 75.36 | 18.74 | 30.44 | 32 | 3349.77 | 597.85 | 0.16 | 150.00 | 58.81 |
| RP282 | TONG GU HONG::IRGC 81026-1 | LL_S | WS | 72 | 106.11 | 23.04 | 35.24 | 5 | 3643.82 | 568.21 | 0.16 | 50.00 | 46.61 |
| RP283 | TSAI YUAN CHON::IRGC 126-1 | LL_S | WS | 84 | 87.65 | 19.62 | 33.24 | 28 | 3215.74 | 575.29 | 0.16 | 155.00 | 53.69 |
| RP284 | TSAO SHENG LI 1::IRGC 1309-1 | LL_S | WS | 0 | 128.43 | 21.23 | 46.66 | 31 | 4296.85 | 614.36 | 0.16 | 215.00 | 62.39 |
| RP285 | UPRH 233::IRGC 61667-1 | LL_S | WS | 80 | 73.70 | 18.66 | 34.43 | 14 | 3377.84 | 574.85 | 0.16 | 100.00 | 60.92 |
| RP286 | UQUIHUA::IRGC 117037-1 | LL_S | WS | 0 | 99.74 | 23.45 | 45.29 | 20 | 3796.14 | 571.66 | 0.16 | 90.00 | 51.22 |
| RP287 | URAIBOOL::IRGC 52785-1 | LL_S | WS | 0 | 44.53 | 12.37 | 29.19 | 14 | 3052.72 | 585.90 | 0.16 | 170.00 | 57.90 |
| RP288 | VEN THAP::IRGC 56138-1 | LL_S | WS | 84 | 99.09 | 20.52 | 36.23 | 30 | 4180.28 | 603.38 | 0.16 | 250.00 | 62.59 |
| RP289 | WANGA BARUGULU::IRGC 52261-1 | LL_S | WS | 83 | 108.51 | 21.76 | 35.19 | 29 | 4430.81 | 584.16 | 0.16 | 85.00 | 61.24 |
| RP290 | WAR 72-2-1-1::IRGC 117361-1 | LL_S | WS | 0 | 100.76 | 19.98 | 50.30 | 29 | 3832.81 | 589.91 | 0.16 | 220.00 | 61.41 |
| RP291 | WI BIR SHUN::IRGC 4602-1 | LL_S | WS | 86 | 112.30 | 20.41 | 44.35 | 35 | 3598.15 | 591.39 | 0.16 | 230.00 | 62.47 |
| RP292 | WP 36::IRGC 55278-1 | LL_S | WS | 0 | 71.09 | 17.58 | 26.91 | 7 | 3250.39 | 582.32 | 0.16 | 130.00 | 53.96 |
| RP293 | WP 65::IRGC 36526-1 | LL_S | WS | 0 | 84.66 | 20.28 | 30.11 | 20 | 3645.49 | 586.22 | 0.16 | 205.00 | 61.58 |
| RP294 | XI GAN JING REN::IRGC 60035-1 | LL_S | WS | 70 | 106.14 | 21.81 | 34.66 | 21 | 3706.27 | 577.04 | 0.16 | 140.00 | 61.08 |
| RP295 | XIA ZHI BAI::IRGC 53437-1 | LL_S | WS | 64 | 100.69 | 18.95 | 25.59 | 36 | 3913.73 | 587.99 | 0.16 | 200.00 | 60.95 |
| **Study designation** | **3KG database designation** | **Env** | **Season** | **DTF** | **PH (cm)** | **PL (cm)** | **FlgLA (cm^2^)** | **NBP** | **BMDW (kgha^-1^)** | **GY (kgha^-1^)** | **HI** | **TGW (g)** | **SPKFT (%)** |
| RP296 | XITTO::IRGC 6671-1 | LL_S | WS | 0 | 144.89 | 21.67 | 61.38 | 10 | 5134.07 | 573.75 | 0.16 | 210.00 | 49.36 |
| RP297 | YA NONG ZAO 4::IRGC 63908-1 | LL_S | WS | 0 | 67.29 | 16.92 | 36.88 | 27 | 2976.55 | 609.98 | 0.16 | 260.00 | 62.83 |
| RP298 | YEBAWYIN::IRGC 33885-1 | LL_S | WS | 81 | 135.62 | 26.40 | 65.59 | 32 | 4016.10 | 599.05 | 0.16 | 175.00 | 63.11 |
| RP299 | YONG JIN ZAO 3::IRGC 70441-1 | LL_S | WS | 74 | 77.95 | 19.29 | 32.73 | 20 | 3587.24 | 572.87 | 0.16 | 180.00 | 56.82 |
| RP300 | H 6::IRGC 157-1 | LL_S | WS | 0 | 110.77 | 21.61 | 40.60 | 19 | 3437.46 | 577.08 | 0.16 | 100.00 | 55.42 |
| RP002 | 19::IRGC 70786-1 | LL_S | DS | NA | 66.84 | 16.13 | 28.08 | 5 | 3343.33 | 71.32 | 0.03 | 130.86 | 29.56 |
| RP003 | 3210::IRGC 116950-1 | LL_S | DS | 0 | 0.00 | 0.00 | 0.00 | 0 | 0.00 | 0.00 | 0.00 | 0.00 | 0.00 |
| RP004 | 498-2A BR 8::IRGC 5891-1 | LL_S | DS | NA | 76.27 | 16.98 | 29.71 | 4 | 3889.19 | 52.82 | 0.02 | 0.00 | NA |
| RP005 | ADT 12::IRGC 6254-1 | LL_S | DS | 79 | 101.80 | 22.06 | 37.04 | 28 | 5497.25 | 709.02 | 0.15 | 151.77 | 59.14 |
| RP006 | AE NOUA::IRGC 89308-1 | LL_S | DS | 94 | 84.31 | 19.58 | 36.88 | 14 | 5076.79 | 54.15 | 0.03 | 151.77 | 42.39 |
| RP007 | ALTAMIRA 9::IRGC 116953-1 | LL_S | DS | 0 | 0.00 | 0.00 | 0.00 | 0 | 0.00 | 0.00 | 0.00 | 0.00 | 0.00 |
| RP008 | ARAURE 1::IRGC 116956-1 | LL_S | DS | 96 | 62.27 | 17.53 | 19.73 | 14 | 3754.68 | 59.71 | 0.04 | 137.48 | 41.73 |
| RP009 | ARC 10100::IRGC 20709-1 | LL_S | DS | 101 | 64.57 | 17.22 | 30.48 | 7 | 2574.00 | 66.68 | 0.02 | 123.19 | 29.53 |
| RP010 | ARC 10594::IRGC 12524-1 | LL_S | DS | 97 | 79.60 | 19.21 | 37.19 | 10 | 3235.95 | 55.64 | 0.05 | 137.48 | 55.94 |
| RP011 | ARC 10754::IRGC 12603-1 | LL_S | DS | 93 | 81.62 | 21.23 | 35.50 | 12 | 3685.11 | 50.15 | 0.03 | 146.05 | 32.53 |
| RP012 | ARC 10812::IRGC 21074-1 | LL_S | DS | 68 | 83.23 | 20.91 | 35.27 | 28 | 4513.87 | 500.41 | 0.12 | 154.62 | 58.09 |
| RP013 | ARC 11524::IRGC 42672-1 | LL_S | DS | 100 | 77.15 | 19.25 | 27.47 | 6 | 3600.69 | 57.70 | 0.02 | 128.91 | 44.77 |
| RP014 | ARC 11857::IRGC 40972-1 | LL_S | DS | 80 | 82.27 | 18.83 | 30.54 | 24 | 4457.75 | 780.96 | 0.14 | 154.62 | 41.42 |
| RP015 | ARC 11901::IRGC 21727-1 | LL_S | DS | 89 | 68.57 | 18.26 | 30.68 | 16 | 3692.93 | 99.78 | 0.04 | 126.05 | 49.32 |
| RP018 | ARC 13778::IRGC 41216-1 | LL_S | DS | 78 | 90.74 | 19.77 | 40.12 | 16 | 4495.93 | 87.52 | 0.03 | 151.77 | 43.74 |
| RP019 | ARC 14060::IRGC 41374-1 | LL_S | DS | NA | 74.06 | 19.46 | 32.93 | 7 | 4543.33 | 51.98 | 0.02 | 128.91 | 31.76 |
| RP020 | ARC 14064::IRGC 41377-1 | LL_S | DS | 80 | 95.04 | 20.60 | 42.37 | 12 | 3345.49 | 192.57 | 0.06 | 151.77 | 48.45 |
| RP021 | ARC 14654::IRGC 41663-1 | LL_S | DS | NA | 74.87 | 19.97 | 26.77 | 4 | 4653.64 | 49.89 | 0.02 | 0.00 | NA |
| RP022 | ARC 15873::IRGC 43250-1 | LL_S | DS | 101 | 82.27 | 17.06 | 27.32 | 22 | 4830.30 | 53.81 | 0.02 | 143.19 | 27.57 |
| RP023 | ARC 18092::IRGC 42256-1 | LL_S | DS | 72 | 80.01 | 21.43 | 31.54 | 24 | 4521.30 | 231.77 | 0.10 | 163.19 | 58.59 |
| RP024 | ARC 18112::IRGC 42274-1 | LL_S | DS | 89 | 81.22 | 18.31 | 29.78 | 24 | 3955.17 | 68.47 | 0.04 | 148.91 | 43.94 |
| RP025 | ARC 18202::IRGC 42328-1 | LL_S | DS | 105 | 79.79 | 18.91 | 26.03 | 10 | 4102.33 | 97.63 | 0.03 | 133.73 | 30.28 |
| RP026 | ARC 18597::IRGC 43299-1 | LL_S | DS | 69 | 69.41 | 17.27 | 30.18 | 15 | 3430.43 | 115.84 | 0.04 | 140.34 | 36.50 |
| **Study designation** | **3KG database designation** | **Env** | **Season** | **DTF** | **PH (cm)** | **PL (cm)** | **FlgLA (cm^2^)** | **NBP** | **BMDW (kgha^-1^)** | **GY (kgha^-1^)** | **HI** | **TGW (g)** | **SPKFT (%)** |
| RP028 | ASHI BINNI::IRGC 77216-1 | LL_S | DS | 99 | 62.70 | 17.77 | 21.80 | 5 | 3659.14 | 82.72 | 0.04 | 0.00 | 49.44 |
| RP029 | ASHMBER::IRGC 27522-1 | LL_S | DS | 105 | 69.25 | 18.78 | 30.10 | 9 | 4150.54 | 72.13 | 0.04 | 154.20 | 50.44 |
| RP030 | ASU::IRGC 62154-1 | LL_S | DS | 94 | 65.88 | 17.76 | 26.78 | 7 | 3810.02 | 59.59 | 0.02 | 143.19 | 31.50 |
| RP031 | AUS 171::IRGC 29004-1 | LL_S | DS | 104 | 64.53 | 15.82 | 25.77 | 11 | 3194.70 | 50.39 | 0.02 | 128.91 | 23.42 |
| RP032 | AUS 177::IRGC 29009-1 | LL_S | DS | NA | 61.04 | 16.56 | 21.14 | 6 | 3002.92 | 55.75 | 0.03 | 124.20 | 58.86 |
| RP033 | AUS 219::IRGC 29031-1 | LL_S | DS | 71 | 80.27 | 20.08 | 32.96 | 21 | 4564.60 | 1049.96 | 0.20 | 151.77 | 64.13 |
| RP034 | AUS 233::IRGC 29036-1 | LL_S | DS | 74 | 88.74 | 20.60 | 33.46 | 24 | 4436.61 | 598.31 | 0.08 | 143.19 | 56.02 |
| RP036 | AUS 295::IRGC 29083-1 | LL_S | DS | 107 | 70.05 | 18.33 | 36.38 | 16 | 5130.34 | 89.69 | 0.07 | 146.05 | 43.76 |
| RP037 | AUS 301::IRGC 29089-1 | LL_S | DS | 73 | 82.56 | 17.94 | 35.95 | 30 | 3842.18 | 272.35 | 0.14 | 157.48 | 60.16 |
| RP038 | AUS 308::IRGC 29096-1 | LL_S | DS | 93 | 67.89 | 16.28 | 29.89 | 12 | 3284.63 | 85.18 | 0.03 | 146.05 | 47.84 |
| RP039 | AUS 329::IRGC 29116-1 | LL_S | DS | 80 | 74.09 | 18.54 | 30.56 | 24 | 3766.08 | 315.70 | 0.07 | 148.91 | 52.53 |
| RP040 | AUS 344::IRGC 29131-1 | LL_S | DS | 80 | 73.29 | 19.09 | 33.18 | 25 | 3971.70 | 338.44 | 0.14 | 146.05 | 57.80 |
| RP041 | AUS 439::IRGC 29221-1 | LL_S | DS | 93 | 64.69 | 16.02 | 25.81 | 13 | 3349.97 | 136.57 | 0.07 | 140.34 | 53.00 |
| RP042 | AUS PADDY (RED)::IRGC 44978-1 | LL_S | DS | 74 | 79.21 | 18.36 | 34.60 | 25 | 4112.88 | 416.27 | 0.14 | 154.62 | 59.84 |
| RP043 | B 4414 F-MR-6-3::IRGC 117310-1 | LL_S | DS | 0 | 0.00 | 0.00 | 0.00 | 0 | 0.00 | 0.00 | 0.00 | 0.00 | 0.00 |
| RP044 | B 6136 E-3-TB-0-1-5::IRGC 117311-1 | LL_S | DS | 101 | 63.11 | 18.07 | 26.98 | 25 | 3539.71 | 68.97 | 0.03 | 146.05 | 34.32 |
| RP045 | B 6136-3-TB-0-1-5::IRGC 117312-1 | LL_S | DS | 86 | 60.01 | 16.61 | 33.15 | 26 | 4252.13 | 75.51 | 0.03 | 134.62 | 32.30 |
| RP046 | B 6149 F-MR-7::IRGC 117314-1 | LL_S | DS | 88 | 81.40 | 19.45 | 31.66 | 24 | 4304.79 | 163.86 | 0.08 | 154.62 | 47.67 |
| RP047 | BA BAI GU::IRGC 79580-1 | LL_S | DS | 66 | 77.05 | 19.09 | 34.45 | 30 | 3731.36 | 459.89 | 0.09 | 137.48 | 51.73 |
| RP048 | BA SHI ZAO::IRGC 67903-1 | LL_S | DS | 96 | 78.48 | 17.73 | 34.29 | 9 | 3335.62 | 76.52 | 0.04 | 146.05 | 55.49 |
| RP049 | BADAL 1163::IRGC 32796-1 | LL_S | DS | 85 | 86.73 | 20.70 | 35.66 | 13 | 4193.72 | 143.99 | 0.04 | 154.62 | 39.96 |
| RP050 | BADUIE::IRGC 53715-1 | LL_S | DS | 108 | 73.82 | 17.93 | 25.61 | 11 | 3507.68 | 62.98 | 0.03 | 140.34 | 27.41 |
| RP051 | BAI HE::IRGC 76438-1 | LL_S | DS | 92 | 60.39 | 15.91 | 25.24 | 13 | 3239.16 | 90.37 | 0.03 | 126.05 | 50.56 |
| RP052 | BAIANG 6::IRGC 6129-1 | LL_S | DS | 103 | 70.75 | 18.05 | 26.77 | 10 | 5067.18 | 78.06 | 0.03 | 147.06 | 54.02 |
| RP053 | BAK TULSI::IRGC 34831-1 | LL_S | DS | 82 | 64.68 | 18.10 | 33.44 | 17 | 3500.89 | 205.11 | 0.09 | 154.62 | 52.47 |
| RP054 | BAKASI::IRGC 27074-1 | LL_S | DS | NA | 67.74 | 16.74 | 28.08 | 5 | 4382.83 | 66.62 | 0.03 | 123.19 | 27.45 |
| RP055 | BALASURIYA A::IRGC 66509-1 | LL_S | DS | 98 | 75.52 | 18.59 | 23.71 | 9 | 4678.11 | 59.06 | 0.02 | 130.39 | 23.66 |
| RP056 | BAMOA A 75::IRGC 51101-1 | LL_S | DS | 85 | 61.74 | 19.30 | 27.75 | 23 | 3833.21 | 78.14 | 0.04 | 163.19 | 33.81 |
| **Study designation** | **3KG database designation** | **Env** | **Season** | **DTF** | **PH (cm)** | **PL (cm)** | **FlgLA (cm^2^)** | **NBP** | **BMDW (kgha^-1^)** | **GY (kgha^-1^)** | **HI** | **TGW (g)** | **SPKFT (%)** |
| RP057 | BANDI::IRGC 17214-1 | LL_S | DS | 0 | 0.00 | 0.00 | 0.00 | 0 | 0.00 | 0.00 | 0.00 | 0.00 | 0.00 |
| RP058 | BANGKOUY::IRGC 94037-1 | LL_S | DS | 95 | 86.87 | 20.91 | 27.04 | 15 | 3354.33 | 67.66 | 0.05 | 157.48 | 35.94 |
| RP059 | BARIK KUDI::IRGC 52807-1 | LL_S | DS | 70 | 74.11 | 18.98 | 30.40 | 18 | 3082.86 | 135.70 | 0.03 | 126.05 | 39.11 |
| RP060 | BAT DO::IRGC 7014-1 | LL_S | DS | 71 | 87.14 | 19.61 | 30.23 | 39 | 3634.89 | 446.97 | 0.11 | 140.34 | 51.98 |
| RP061 | BATHURI::IRGC 25838-1 | LL_S | DS | 79 | 73.30 | 17.27 | 37.18 | 15 | 3235.44 | 143.44 | 0.03 | 154.62 | 35.32 |
| RP062 | BAZAIL::IRGC 27526-1 | LL_S | DS | 98 | 60.38 | 19.15 | 28.27 | 12 | 3265.03 | 61.56 | 0.03 | 133.73 | 40.84 |
| RP063 | BENGALY MORIMO::IRGC 10976-1 | LL_S | DS | NA | 61.44 | 16.89 | 27.07 | 4 | 3874.88 | 82.82 | 0.03 | 0.00 | 29.43 |
| RP065 | BHADOIA 303::IRGC 6588-1 | LL_S | DS | 69 | 81.64 | 18.52 | 28.97 | 25 | 4336.05 | 336.47 | 0.11 | 148.91 | 54.10 |
| RP066 | BK 26::IRGC 45197-1 | LL_S | DS | 98 | 77.23 | 19.32 | 32.39 | 4 | 4088.29 | 82.82 | 0.03 | 140.39 | NA |
| RP067 | BKN BR 1031-78-5-4::IRGC 55927-1 | LL_S | DS | 0 | 0.00 | 0.00 | 0.00 | 0 | 0.00 | 0.00 | 0.00 | 0.00 | 0.00 |
| RP068 | BONG SEN::IRGC 7011-1 | LL_S | DS | 74 | 81.50 | 20.18 | 34.34 | 17 | 4052.41 | 453.96 | 0.10 | 146.05 | 56.66 |
| RP070 | BPI 76 NON SENSITIVE (GREEN)::IRGC 9790-1 | LL_S | DS | NA | 66.82 | 16.89 | 29.37 | 8 | 3327.14 | 67.92 | 0.04 | 151.77 | 48.62 |
| RP071 | BR 51-115-4::IRGC 43999-1 | LL_S | DS | 99 | 60.78 | 17.04 | 25.05 | 22 | 4521.68 | 100.41 | 0.05 | 144.20 | 48.08 |
| RP072 | BR 5230-46-4::IRGC 117318-1 | LL_S | DS | 83 | 58.38 | 18.10 | 27.51 | 13 | 2965.25 | 77.19 | 0.04 | 150.86 | 47.01 |
| RP073 | BR IRGA 409::IRGC 55915-1 | LL_S | DS | 98 | 64.16 | 17.21 | 21.57 | 12 | 4015.90 | 97.95 | 0.04 | 148.91 | 36.69 |
| RP074 | BW 295-5::IRGC 63098-1 | LL_S | DS | 102 | 62.57 | 16.48 | 20.35 | 10 | 3833.34 | 61.68 | 0.03 | 128.91 | 42.13 |
| RP075 | C 1016-1::IRGC 50368-1 | LL_S | DS | 97 | 68.85 | 19.60 | 36.91 | 10 | 3474.50 | 79.46 | 0.03 | 131.77 | 33.12 |
| RP076 | C 166-135::IRGC 50633-1 | LL_S | DS | NA | 63.60 | 17.92 | 28.41 | 5 | 2923.83 | 80.35 | 0.03 | 0.00 | NA |
| RP077 | C 662083::IRGC 62101-1 | LL_S | DS | 100 | 95.39 | 20.11 | 34.87 | 7 | 5081.02 | 140.55 | 0.03 | 140.39 | 35.34 |
| RP079 | CAUVERY::IRGC 45255-1 | LL_S | DS | 94 | 52.32 | 17.06 | 29.77 | 22 | 3126.03 | 75.34 | 0.03 | 143.19 | 29.24 |
| RP080 | CE IN TSAN::IRGC 4362-1 | LL_S | DS | 0 | 0.00 | 0.00 | 0.00 | 0 | 0.00 | 0.00 | 0.00 | 0.00 | 0.00 |
| RP081 | CEA 3::IRGC 116965-1 | LL_S | DS | NA | 52.09 | 16.21 | 24.67 | 4 | 3996.05 | 83.14 | 0.03 | 0.00 | NA |
| RP082 | CEMPO MANGGAR::IRGC 27107-1 | LL_S | DS | 0 | 0.00 | 0.00 | 0.00 | 0 | 0.00 | 0.00 | 0.00 | 0.00 | 0.00 |
| RP083 | CHAKOL::IRGC 77226-1 | LL_S | DS | NA | 58.85 | 15.76 | 46.38 | 5 | 4544.82 | 84.56 | 0.03 | 0.00 | NA |
| RP084 | CHAM LEK::IRGC 89387-1 | LL_S | DS | 90 | 79.88 | 18.93 | 31.49 | 15 | 3702.28 | 68.24 | 0.02 | 148.91 | 26.27 |
| RP085 | CHAMA (DWARF)::IRGC 69487-1 | LL_S | DS | NA | 52.08 | 15.60 | 22.77 | 18 | 3913.02 | 58.54 | 0.02 | 130.39 | 21.07 |
| RP086 | CHANDARHAT::IRGC 25845-1 | LL_S | DS | 74 | 74.76 | 19.14 | 29.99 | 27 | 3845.76 | 203.28 | 0.14 | 157.48 | 58.80 |
| RP087 | CHANDINA::IRGC 36420-1 | LL_S | DS | 85 | 52.46 | 18.05 | 23.47 | 18 | 3060.31 | 69.26 | 0.03 | 143.19 | 33.14 |
| **Study designation** | **3KG database designation** | **Env** | **Season** | **DTF** | **PH (cm)** | **PL (cm)** | **FlgLA (cm^2^)** | **NBP** | **BMDW (kgha^-1^)** | **GY (kgha^-1^)** | **HI** | **TGW (g)** | **SPKFT (%)** |
| RP088 | CHANG LE SAN SHU ZAO::IRGC 63561-1 | LL_S | DS | 77 | 55.00 | 17.89 | 30.43 | 42 | 3540.09 | 296.39 | 0.11 | 144.20 | 55.00 |
| RP089 | CHAO PEUAK DENG::IRGC 11602-1 | LL_S | DS | 103 | 81.09 | 20.50 | 27.53 | 7 | 3703.69 | 59.27 | 0.02 | 147.06 | 41.36 |
| RP090 | CHI SHENG TAO::IRGC 4606-1 | LL_S | DS | NA | 63.75 | 17.06 | 27.80 | 6 | 3275.67 | 69.85 | 0.03 | 157.48 | 39.55 |
| RP091 | CHIAYI WU-K'O::IRGC 64974-1 | LL_S | DS | 78 | 76.14 | 20.08 | 34.78 | 17 | 3746.73 | 199.30 | 0.06 | 154.62 | 43.78 |
| RP092 | CHIH SHEN LI::IRGC 1306-1 | LL_S | DS | 88 | 69.67 | 18.32 | 34.53 | 13 | 4740.50 | 63.84 | 0.02 | 131.77 | 32.99 |
| RP093 | CHNNOR::IRGC 67485-1 | LL_S | DS | 98 | 82.99 | 17.99 | 27.54 | 7 | 3363.81 | 82.61 | 0.02 | 134.62 | 29.87 |
| RP094 | CHUA DAU::IRGC 4785-1 | LL_S | DS | 74 | 80.84 | 19.45 | 30.74 | 27 | 4139.01 | 126.87 | 0.06 | 146.05 | 48.60 |
| RP095 | CHUNGUR BALI::IRGC 25855-1 | LL_S | DS | 70 | 79.24 | 19.40 | 30.27 | 37 | 4064.45 | 572.18 | 0.19 | 140.34 | 64.77 |
| RP096 | CICA 9::IRGC 53079-1 | LL_S | DS | 0 | 0.00 | 0.00 | 0.00 | 0 | 0.00 | 0.00 | 0.00 | 0.00 | 0.00 |
| RP097 | CN 44-40-7::IRGC 45368-1 | LL_S | DS | 104 | 57.98 | 16.33 | 23.00 | 15 | 3924.17 | 58.30 | 0.03 | 128.91 | 43.73 |
| RP098 | CO 39::IRGC 51231-1 | LL_S | DS | 63 | 53.39 | 18.31 | 22.48 | 35 | 3545.09 | 554.26 | 0.23 | 140.34 | 54.95 |
| RP099 | CR 60-10::IRGC 15777-1 | LL_S | DS | NA | 47.51 | 15.86 | 22.20 | 12 | 2760.79 | 65.97 | 0.03 | 131.77 | 39.39 |
| RP100 | CRILLO LA FRIA::IRGC 10793-1 | LL_S | DS | 87 | 58.27 | 15.39 | 24.14 | 10 | 3235.95 | 55.01 | 0.02 | 140.39 | 26.93 |
| RP101 | CSR-90 IR-2::IRGC 117327-1 | LL_S | DS | 98 | 53.01 | 15.88 | 22.68 | 10 | 3293.99 | 49.55 | 0.02 | 140.39 | 33.50 |
| RP102 | CT 9737-6-1-1-2-2P-M::IRGC 117330-1 | LL_S | DS | NA | 48.03 | 16.75 | 22.90 | 9 | 3352.41 | 45.71 | 0.02 | 120.86 | 20.37 |
| RP103 | CUN GU NUO::IRGC 63576-1 | LL_S | DS | NA | 51.52 | 15.70 | 25.49 | 11 | 3899.31 | 60.40 | 0.02 | 146.05 | 29.43 |
| RP104 | CUYAMEL 3820::IRGC 116975-1 | LL_S | DS | 0 | 0.00 | 0.00 | 0.00 | 0 | 0.00 | 0.00 | 0.00 | 0.00 | 0.00 |
| RP105 | DA 11::IRGC 6046-1 | LL_S | DS | 0 | 0.00 | 0.00 | 0.00 | 0 | 0.00 | 0.00 | 0.00 | 0.00 | 0.00 |
| RP106 | DA GANG ZHAN::IRGC 67103-1 | LL_S | DS | 71 | 78.05 | 20.29 | 28.89 | 30 | 3706.76 | 373.20 | 0.07 | 137.48 | 45.29 |
| RP107 | DA NUO (ZHAN)::IRGC 72025-1 | LL_S | DS | 102 | 66.08 | 16.75 | 24.73 | 12 | 3551.11 | 61.27 | 0.03 | 143.19 | 38.15 |
| RP108 | DAA MANSA::IRGC 67559-1 | LL_S | DS | NA | 69.75 | 17.58 | 30.40 | 5 | 3489.22 | 79.37 | 0.03 | 0.00 | 29.06 |
| RP109 | DANGAR::IRGC 76296-1 | LL_S | DS | 70 | 84.48 | 18.05 | 31.96 | 24 | 3943.64 | 571.72 | 0.15 | 154.62 | 57.90 |
| RP110 | DHANE BURWA::IRGC 10105-1 | LL_S | DS | 103 | 71.41 | 19.51 | 27.49 | 10 | 3726.75 | 95.12 | 0.06 | 143.19 | 59.80 |
| RP111 | DISSI::IRGC 101346-1 | LL_S | DS | NA | 70.02 | 17.69 | 40.56 | 4 | 4547.92 | 78.85 | 0.03 | 0.00 | NA |
| RP112 | DUDH KADAR::IRGC 67707-1 | LL_S | DS | 105 | 65.38 | 17.76 | 33.69 | 9 | 2915.42 | 58.88 | 0.02 | 126.05 | 28.89 |
| RP113 | E 2024::IRGC 67958-1 | LL_S | DS | 97 | 48.32 | 15.65 | 21.28 | 25 | 3476.93 | 64.99 | 0.03 | 131.77 | 39.00 |
| RP114 | E 2040::IRGC 67968-1 | LL_S | DS | 85 | 46.44 | 14.94 | 24.62 | 14 | 2817.16 | 312.17 | 0.10 | 134.62 | 39.85 |
| RP115 | E DAW HAWM::IRGC 47938-1 | LL_S | DS | 0 | 0.00 | 0.00 | 0.00 | 0 | 0.00 | 0.00 | 0.00 | 0.00 | 0.00 |
| **Study designation** | **3KG database designation** | **Env** | **Season** | **DTF** | **PH (cm)** | **PL (cm)** | **FlgLA (cm^2^)** | **NBP** | **BMDW (kgha^-1^)** | **GY (kgha^-1^)** | **HI** | **TGW (g)** | **SPKFT (%)** |
| RP116 | ELONI::IRGC 116980-1 | LL_S | DS | 97 | 49.12 | 16.52 | 23.90 | 18 | 3627.46 | 83.30 | 0.03 | 143.19 | 49.21 |
| RP117 | EPEAL 102::IRGC 78698-1 | LL_S | DS | NA | 46.66 | 15.13 | 22.27 | 11 | 3306.16 | 58.54 | 0.02 | 0.00 | 24.61 |
| RP118 | ES 21::IRGC 56171-1 | LL_S | DS | 78 | 86.22 | 20.91 | 42.29 | 12 | 5250.43 | 235.86 | 0.08 | 167.53 | 58.36 |
| RP119 | EX FOILAEIN (NAPUTO)::IRGC 81675-1 | LL_S | DS | NA | 74.15 | 17.70 | 24.46 | 6 | 4410.99 | 64.14 | 0.04 | 137.53 | 40.89 |
| RP120 | FEI GAI 122::IRGC 63599-1 | LL_S | DS | NA | 49.23 | 16.14 | 27.41 | 13 | 3500.89 | 60.71 | 0.04 | 151.77 | 48.03 |
| RP121 | FONAIAP 2::IRGC 116985-1 | LL_S | DS | NA | 63.95 | 20.12 | 26.10 | 7 | 5129.59 | 88.90 | 0.04 | 147.06 | 44.36 |
| RP122 | FU ZAO XIAN::IRGC 63619-1 | LL_S | DS | 81 | 56.12 | 18.31 | 28.26 | 14 | 3285.02 | 92.89 | 0.03 | 151.77 | 38.71 |
| RP123 | G 25::IRGC 45733-1 | LL_S | DS | 0 | 0.00 | 0.00 | 0.00 | 0 | 0.00 | 0.00 | 0.00 | 0.00 | 0.00 |
| RP124 | GAO JIAO BAI::IRGC 68047-1 | LL_S | DS | 60 | 70.05 | 19.09 | 27.84 | 34 | 3623.23 | 659.45 | 0.20 | 157.48 | 61.23 |
| RP125 | GENIT::IRGC 3272-1 | LL_S | DS | 94 | 69.51 | 18.26 | 31.04 | 7 | 2828.81 | 76.36 | 0.05 | 148.91 | 62.81 |
| RP126 | GOJOL GORIA::IRGC 26629-1 | LL_S | DS | 0 | 0.00 | 0.00 | 0.00 | 0 | 0.00 | 0.00 | 0.00 | 0.00 | 0.00 |
| RP127 | GOKULGANJA::IRGC 45701-1 | LL_S | DS | 0 | 0.00 | 0.00 | 0.00 | 0 | 0.00 | 0.00 | 0.00 | 0.00 | 0.00 |
| RP128 | GUI HUA ZAO::IRGC 68060-1 | LL_S | DS | 65 | 79.87 | 18.83 | 31.97 | 33 | 3910.59 | 608.03 | 0.19 | 154.62 | 59.11 |
| RP130 | HAWM KRUA::IRGC 64333-1 | LL_S | DS | 101 | 75.49 | 18.10 | 28.85 | 7 | 3735.08 | 57.59 | 0.02 | 120.86 | 29.62 |
| RP131 | HD 10::IRGC 6638-1 | LL_S | DS | NA | 86.60 | 20.16 | 34.64 | 5 | 3896.88 | 41.34 | 0.02 | 111.77 | 42.19 |
| RP132 | HE GU TSAO::IRGC 51302-1 | LL_S | DS | 68 | 79.47 | 20.91 | 38.59 | 23 | 3872.54 | 503.05 | 0.13 | 151.77 | 57.88 |
| RP133 | HODARAWALA::IRGC 67631-1 | LL_S | DS | 85 | 74.39 | 20.21 | 33.72 | 16 | 3527.66 | 161.42 | 0.04 | 134.62 | 42.59 |
| RP134 | HOLOI BASH (SOLOI BASH)::IRGC 64778-1 | LL_S | DS | NA | 59.15 | 15.87 | 30.49 | 5 | 3350.48 | 78.07 | 0.04 | 140.39 | 39.79 |
| RP135 | HTA 22::IRGC 45827-1 | LL_S | DS | 75 | 91.20 | 18.36 | 37.24 | 12 | 4403.82 | 129.26 | 0.03 | 148.91 | 48.08 |
| RP136 | HUA LI ZAO::IRGC 80950-1 | LL_S | DS | 0 | 0.00 | 0.00 | 0.00 | 0 | 0.00 | 0.00 | 0.00 | 0.00 | 0.00 |
| RP137 | I KUNG PAO::IRGC 114-1 | LL_S | DS | 100 | 59.32 | 14.82 | 30.04 | 7 | 3733.28 | 57.13 | 0.02 | 123.73 | 20.84 |
| RP138 | IA CUBA 17::IRGC 116990-1 | LL_S | DS | NA | 54.64 | 16.12 | 28.16 | 15 | 4681.05 | 68.83 | 0.04 | 154.20 | 45.73 |
| RP139 | ICTA CRISPO 38::IRGC 116994-1 | LL_S | DS | 93 | 51.81 | 17.39 | 26.21 | 29 | 3555.33 | 204.19 | 0.09 | 128.91 | 45.72 |
| RP140 | ICTA MOTAGUA::IRGC 116995-1 | LL_S | DS | 98 | 63.21 | 16.93 | 24.47 | 12 | 4145.90 | 82.09 | 0.03 | 143.73 | 26.70 |
| RP141 | INIAP 6::IRGC 117002-1 | LL_S | DS | NA | 50.08 | 16.70 | 26.32 | 10 | 3683.08 | 89.86 | 0.03 | 143.19 | 28.07 |
| RP142 | IR 13429-109-2-2-1::IRGC 63491-1 | LL_S | DS | 93 | 55.55 | 17.01 | 25.74 | 21 | 3731.87 | 84.05 | 0.04 | 143.19 | 46.58 |
| RP143 | IR 21015-72-3-3-3-1::IRGC 117004-1 | LL_S | DS | 98 | 66.48 | 16.80 | 28.22 | 20 | 3681.52 | 154.25 | 0.06 | 140.34 | 45.42 |
| RP144 | IR 2344-P1 PB-9-3-2B::IRGC 39317-C1 | LL_S | DS | 65 | 87.00 | 20.29 | 28.86 | 41 | 4667.98 | 849.28 | 0.20 | 143.19 | 61.41 |
| **Study designation** | **3KG database designation** | **Env** | **Season** | **DTF** | **PH (cm)** | **PL (cm)** | **FlgLA (cm^2^)** | **NBP** | **BMDW (kgha^-1^)** | **GY (kgha^-1^)** | **HI** | **TGW (g)** | **SPKFT (%)** |
| RP146 | IR 63295-AC 209-7::IRGC 117365-1 | LL_S | DS | NA | 56.11 | 17.87 | 23.74 | 6 | 3106.18 | 51.08 | 0.02 | 133.73 | 28.07 |
| RP147 | IR 69502-6-SRN-3-UBN-1-B::IRGC 117290-1 | LL_S | DS | 99 | 59.75 | 16.07 | 21.08 | 17 | 3526.38 | 67.00 | 0.03 | 148.91 | 30.17 |
| RP148 | IR 75870-5-8-5-B-1::IRGC 117297-1 | LL_S | DS | NA | 62.42 | 17.32 | 25.30 | 5 | 3706.63 | 112.14 | 0.02 | 144.20 | 53.54 |
| RP149 | IR 77390-1-6-4-19-1-B::IRGC 117303-1 | LL_S | DS | 0 | 0.00 | 0.00 | 0.00 | 0 | 0.00 | 0.00 | 0.00 | 0.00 | 0.00 |
| RP150 | IR 80310-12-B-1-3-B::IRGC 117307-1 | LL_S | DS | 101 | 62.98 | 17.53 | 24.95 | 13 | 3199.57 | 89.26 | 0.05 | 131.77 | 44.75 |
| RP151 | IR 80340-23-B-12-6-B::IRGC 117309-1 | LL_S | DS | 98 | 57.44 | 15.60 | 23.85 | 12 | 3367.65 | 75.04 | 0.04 | 147.53 | 49.43 |
| RP152 | IRGA 318-11-6-9-2B::IRGC 117339-1 | LL_S | DS | NA | 50.85 | 15.60 | 20.82 | 19 | 3268.62 | 48.77 | 0.02 | 126.05 | 24.84 |
| RP153 | IRGA 370-38-1-1F-C4-2::IRGC 117342-1 | LL_S | DS | 82 | 54.88 | 17.68 | 27.24 | 18 | 2847.01 | 136.19 | 0.04 | 148.91 | 32.87 |
| RP154 | IRGA 370-42-1-1F-C-1::IRGC 117343-1 | LL_S | DS | NA | 50.71 | 16.59 | 23.55 | 13 | 3741.74 | 66.44 | 0.04 | 128.91 | 36.05 |
| RP155 | IRGA 659-1-2-2-2::IRGC 117345-1 | LL_S | DS | 0 | 0.00 | 0.00 | 0.00 | 0 | 0.00 | 0.00 | 0.00 | 0.00 | 0.00 |
| RP156 | IRGA 959-1-2-2F-4-1-4A-6-CA-6X::IRGC 117006-1 | LL_S | DS | 78 | 53.68 | 17.21 | 34.90 | 13 | 2773.21 | 58.13 | 0.03 | 117.48 | 39.48 |
| RP157 | IRI 339::IRGC 46956-1 | LL_S | DS | 106 | 53.27 | 17.63 | 18.68 | 13 | 2454.86 | 87.92 | 0.05 | 0.00 | 39.75 |
| RP158 | IRRIBINI::IRGC 49094-1 | LL_S | DS | 0 | 0.00 | 0.00 | 0.00 | 0 | 0.00 | 0.00 | 0.00 | 0.00 | 0.00 |
| RP159 | JABOR SAIL::IRGC 66831-1 | LL_S | DS | 92 | 72.88 | 17.22 | 33.37 | 14 | 3555.98 | 81.27 | 0.04 | 143.19 | 37.75 |
| RP160 | JAGLI BORO::IRGC 27516-2 | LL_S | DS | 87 | 68.88 | 15.97 | 26.39 | 20 | 4452.37 | 69.81 | 0.03 | 131.77 | 45.07 |
| RP161 | JAMBALI::IRGC 73101-1 | LL_S | DS | 92 | 71.12 | 17.37 | 28.67 | 15 | 3780.17 | 171.45 | 0.09 | 137.48 | 59.46 |
| RP162 | JAO LEUANG::IRGC 65866-1 | LL_S | DS | NA | 85.00 | 17.39 | 28.69 | 4 | 4061.12 | 58.80 | 0.02 | 130.86 | NA |
| RP164 | JHODI BIRUN::IRGC 31812-1 | LL_S | DS | 101 | 62.28 | 17.58 | 31.16 | 15 | 4554.73 | 60.79 | 0.02 | 134.62 | 27.23 |
| RP165 | JIN JUN DAO::IRGC 59710-1 | LL_S | DS | 59 | 68.61 | 18.20 | 26.23 | 34 | 3850.12 | 720.01 | 0.27 | 146.05 | 61.66 |
| RP166 | JUMA 62::IRGC 117011-1 | LL_S | DS | 0 | 0.00 | 0.00 | 0.00 | 0 | 0.00 | 0.00 | 0.00 | 0.00 | 0.00 |
| RP167 | KALABAIL::IRGC 25877-1 | LL_S | DS | 0 | 0.00 | 0.00 | 0.00 | 0 | 0.00 | 0.00 | 0.00 | 0.00 | 0.00 |
| RP168 | KALIA::IRGC 34699-1 | LL_S | DS | 81 | 74.89 | 15.65 | 26.98 | 23 | 3485.00 | 227.04 | 0.13 | 140.34 | 56.43 |
| RP169 | KALO CHAKOL::IRGC 77258-1 | LL_S | DS | 0 | 0.00 | 0.00 | 0.00 | 0 | 0.00 | 0.00 | 0.00 | 0.00 | 0.00 |
| RP170 | KALU ILANKALAYAN::IRGC 36270-1 | LL_S | DS | NA | 74.20 | 16.96 | 30.03 | 7 | 4110.42 | 89.85 | 0.04 | 126.05 | 43.63 |
| RP171 | KAM PAI::IRGC 78245-1 | LL_S | DS | 0 | 0.00 | 0.00 | 0.00 | 0 | 0.00 | 0.00 | 0.00 | 0.00 | 0.00 |
| RP172 | KEERIPALA CHILL PADDY::IRGC 49790-1 | LL_S | DS | 87 | 65.92 | 17.89 | 35.56 | 26 | 3993.99 | 89.05 | 0.05 | 143.19 | 45.26 |
| RP173 | KETAN SERANG::IRGC 14615-1 | LL_S | DS | NA | 59.16 | 15.45 | 24.97 | 4 | 3642.87 | 82.04 | 0.03 | 120.39 | NA |
| **Study designation** | **3KG database designation** | **Env** | **Season** | **DTF** | **PH (cm)** | **PL (cm)** | **FlgLA (cm^2^)** | **NBP** | **BMDW (kgha^-1^)** | **GY (kgha^-1^)** | **HI** | **TGW (g)** | **SPKFT (%)** |
| RP174 | KHAO DAWK MALI 105::IRGC 27748-2 | LL_S | DS | NA | 65.34 | 18.80 | 38.40 | 4 | 3981.56 | 42.96 | 0.02 | 0.00 | NA |
| RP175 | KHAO GRADOOK CHAHNG::IRGC 17111-1 | LL_S | DS | 79 | 84.85 | 19.56 | 31.79 | 14 | 6520.09 | 112.71 | 0.03 | 153.73 | 30.37 |
| RP176 | KHAO' HAWM::IRGC 78257-1 | LL_S | DS | 0 | 0.00 | 0.00 | 0.00 | 0 | 0.00 | 0.00 | 0.00 | 0.00 | 0.00 |
| RP177 | KHAO THI RATE::IRGC 58041-1 | LL_S | DS | 76 | 88.09 | 21.12 | 38.80 | 9 | 4231.12 | 177.72 | 0.04 | 166.05 | 53.91 |
| RP178 | KHARSU 80::IRGC 28016-1 | LL_S | DS | 63 | 63.05 | 18.26 | 32.21 | 27 | 3361.37 | 399.37 | 0.21 | 151.77 | 56.81 |
| RP179 | KITRANA 1007::IRGC 68517-1 | LL_S | DS | 0 | 0.00 | 0.00 | 0.00 | 0 | 0.00 | 0.00 | 0.00 | 0.00 | 0.00 |
| RP180 | KN 1 B 361-1-8-6-9::IRGC 46974-1 | LL_S | DS | 105 | 73.15 | 18.10 | 28.30 | 12 | 3519.34 | 61.25 | 0.02 | 131.77 | 38.02 |
| RP181 | KORASISI::IRGC 5285-1 | LL_S | DS | NA | 71.80 | 17.87 | 28.33 | 6 | 3121.68 | 58.93 | 0.02 | 117.48 | 39.36 |
| RP182 | KOTTEYARAN::IRGC 47383-1 | LL_S | DS | 72 | 83.10 | 21.64 | 32.06 | 21 | 4567.93 | 553.33 | 0.15 | 154.62 | 57.99 |
| RP183 | KOYRA::IRGC 77267-1 | LL_S | DS | 79 | 74.48 | 18.93 | 31.12 | 14 | 5211.56 | 110.02 | 0.04 | 137.48 | 38.88 |
| RP184 | KULA KARUPPAN::IRGC 55328-1 | LL_S | DS | NA | 76.42 | 18.04 | 27.44 | 8 | 4130.23 | 85.14 | 0.04 | 0.00 | 42.14 |
| RP185 | KUNENG::IRGC 71545-1 | LL_S | DS | 0 | 0.00 | 0.00 | 0.00 | 0 | 0.00 | 0.00 | 0.00 | 0.00 | 0.00 |
| RP186 | KURULU WEE (WHITE)::IRGC 66518-1 | LL_S | DS | 75 | 82.15 | 22.06 | 36.29 | 19 | 3463.61 | 248.46 | 0.08 | 123.19 | 48.30 |
| RP188 | KUSHIARA::IRGC 34709-1 | LL_S | DS | 0 | 0.00 | 0.00 | 0.00 | 0 | 0.00 | 0.00 | 0.00 | 0.00 | 0.00 |
| RP189 | KUTTA::IRGC 52184-1 | LL_S | DS | 0 | 0.00 | 0.00 | 0.00 | 0 | 0.00 | 0.00 | 0.00 | 0.00 | 0.00 |
| RP190 | LABRA::IRGC 74757-1 | LL_S | DS | NA | 77.86 | 17.63 | 33.64 | 7 | 3968.49 | 82.34 | 0.02 | 140.34 | 26.48 |
| RP191 | LAI YIP ZIM::IRGC 4955-1 | LL_S | DS | 103 | 64.53 | 16.22 | 30.26 | 8 | 3403.01 | 42.72 | 0.02 | 130.39 | 36.39 |
| RP192 | LALKA (LAL DHAN)::IRGC 64946-1 | LL_S | DS | 0 | 0.00 | 0.00 | 0.00 | 0 | 0.00 | 0.00 | 0.00 | 0.00 | 0.00 |
| RP193 | LALSAITA::IRGC 43915-1 | LL_S | DS | 74 | 75.83 | 18.67 | 35.08 | 23 | 3943.90 | 453.25 | 0.15 | 154.62 | 57.20 |
| RP194 | LARHA MUGAD::IRGC 52339-1 | LL_S | DS | 78 | 67.76 | 18.93 | 32.48 | 24 | 3281.82 | 142.92 | 0.12 | 143.19 | 53.95 |
| RP196 | LENJA MURALI::IRGC 66815-1 | LL_S | DS | 74 | 76.28 | 18.64 | 41.04 | 9 | 4046.09 | 127.97 | 0.03 | 137.48 | 33.02 |
| RP197 | LEUANG YAI 29-12-2::IRGC 881-1 | LL_S | DS | NA | 65.71 | 15.30 | 36.29 | 4 | 5257.83 | 76.13 | 0.03 | 0.00 | 29.49 |
| RP198 | LU MAO ZHAN::IRGC 68159-1 | LL_S | DS | 60 | 74.63 | 19.35 | 26.19 | 23 | 3521.26 | 353.75 | 0.18 | 154.62 | 58.06 |
| RP199 | LUA CHAN HUONG::IRGC 16800-1 | LL_S | DS | 0 | 0.00 | 0.00 | 0.00 | 0 | 0.00 | 0.00 | 0.00 | 0.00 | 0.00 |
| RP200 | MAKALIOKA::IRGC 376-1 | LL_S | DS | 89 | 74.90 | 20.36 | 29.42 | 12 | 3508.19 | 203.41 | 0.06 | 137.48 | 50.02 |
| RP201 | MAKRO::IRGC 74763-1 | LL_S | DS | NA | 79.23 | 18.29 | 33.72 | 4 | 3980.29 | 57.99 | 0.03 | 120.86 | NA |
| RP202 | MAMORIAKA::IRGC 68672-1 | LL_S | DS | NA | 78.94 | 18.60 | 28.33 | 4 | 3453.40 | 81.82 | 0.03 | 131.77 | NA |
| RP203 | MEI FENG 9::IRGC 63735-1 | LL_S | DS | 80 | 53.11 | 19.14 | 30.60 | 24 | 3336.14 | 238.21 | 0.13 | 174.62 | 57.43 |
| **Study designation** | **3KG database designation** | **Env** | **Season** | **DTF** | **PH (cm)** | **PL (cm)** | **FlgLA (cm^2^)** | **NBP** | **BMDW (kgha^-1^)** | **GY (kgha^-1^)** | **HI** | **TGW (g)** | **SPKFT (%)** |
| RP204 | MEKENZIE SMALL::IRGC 49895-1 | LL_S | DS | NA | 69.30 | 19.62 | 30.04 | 4 | 3406.11 | 107.17 | 0.03 | 150.39 | NA |
| RP205 | MELEKE::IRGC 56823-1 | LL_S | DS | NA | 70.47 | 18.71 | 34.73 | 4 | 3902.95 | 79.60 | 0.03 | 0.00 | NA |
| RP206 | MENTIK TJERE BELUT::IRGC 18254-1 | LL_S | DS | 0 | 0.00 | 0.00 | 0.00 | 0 | 0.00 | 0.00 | 0.00 | 0.00 | 0.00 |
| RP207 | MILYANG 30::IRGC 46977-1 | LL_S | DS | 102 | 46.83 | 14.64 | 18.68 | 19 | 2939.63 | 49.03 | 0.02 | 134.20 | 25.28 |
| RP208 | MILYANG 77::IRGC 69340-1 | LL_S | DS | 76 | 54.09 | 18.10 | 25.45 | 31 | 3979.51 | 95.02 | 0.06 | 143.19 | 45.86 |
| RP209 | MIN KE ZHAN::IRGC 72230-1 | LL_S | DS | 73 | 62.30 | 21.12 | 26.73 | 35 | 4090.07 | 819.38 | 0.22 | 140.34 | 58.70 |
| RP210 | MIN ZAO 6::IRGC 63772-1 | LL_S | DS | 88 | 48.71 | 17.16 | 30.89 | 10 | 2843.67 | 68.90 | 0.08 | 128.91 | 60.41 |
| RP211 | MODDAI KARUPPAN::IRGC 15465-1 | LL_S | DS | 102 | 88.49 | 19.14 | 28.09 | 15 | 5214.00 | 69.22 | 0.03 | 160.34 | 36.77 |
| RP212 | MUKKALA BAZAL::IRGC 77279-1 | LL_S | DS | 0 | 0.00 | 0.00 | 0.00 | 0 | 0.00 | 0.00 | 0.00 | 0.00 | 0.00 |
| RP213 | MULLIKURUVA::IRGC 77529-1 | LL_S | DS | NA | 79.49 | 20.49 | 40.05 | 5 | 4523.55 | 79.60 | 0.03 | 0.00 | 28.54 |
| RP214 | MUTA GANJE::IRGC 26744-1 | LL_S | DS | 0 | 0.00 | 0.00 | 0.00 | 0 | 0.00 | 0.00 | 0.00 | 0.00 | 0.00 |
| RP215 | MUTTU SAMBA::IRGC 36333-1 | LL_S | DS | 105 | 92.38 | 16.96 | 35.71 | 5 | 3687.42 | 48.62 | 0.02 | 131.77 | 55.14 |
| RP216 | N 22::IRGC 46459-1 | LL_S | DS | 94 | 63.74 | 15.60 | 25.27 | 12 | 4139.52 | 171.00 | 0.03 | 126.05 | 31.42 |
| RP217 | Na souan::IRGC 11889-1 | LL_S | DS | NA | 75.72 | 16.93 | 30.11 | 6 | 2922.79 | 82.64 | NA | 0.00 | NA |
| RP218 | NAZIRA SAIL::IRGC 77284-1 | LL_S | DS | 0 | 0.00 | 0.00 | 0.00 | 0 | 0.00 | 0.00 | 0.00 | 0.00 | 0.00 |
| RP219 | NCS 194::IRGC 51932-1 | LL_S | DS | 101 | 66.32 | 15.76 | 29.85 | 17 | 4334.51 | 57.51 | 0.03 | 127.53 | 29.49 |
| RP220 | NCS 237::IRGC 62202-1 | LL_S | DS | NA | 70.22 | 18.11 | 30.28 | 5 | 3919.66 | 82.95 | 0.03 | 147.53 | 28.82 |
| RP222 | NCS 964 C::IRGC 62604-1 | LL_S | DS | 0 | 0.00 | 0.00 | 0.00 | 0 | 0.00 | 0.00 | 0.00 | 0.00 | 0.00 |
| RP223 | NIAO YAO::IRGC 5496-1 | LL_S | DS | 90 | 58.39 | 16.07 | 25.33 | 12 | 3481.29 | 60.66 | 0.03 | 123.19 | 41.25 |
| RP224 | NIBARI::IRGC 67742-1 | LL_S | DS | 77 | 90.51 | 20.08 | 38.04 | 16 | 4345.27 | 415.47 | 0.09 | 154.62 | 51.99 |
| RP225 | NONA BOKRA::IRGC 22710-C1 | LL_S | DS | 74 | 81.39 | 21.02 | 35.11 | 26 | 4346.81 | 227.18 | 0.09 | 160.34 | 51.66 |
| RP226 | NOROI::IRGC 31611-1 | LL_S | DS | 65 | 95.87 | 20.19 | 35.40 | 29 | 4258.92 | 307.74 | 0.11 | 166.05 | 51.45 |
| RP228 | NX 3533::IRGC 63796-1 | LL_S | DS | 82 | 48.70 | 16.56 | 26.99 | 22 | 3584.93 | 100.11 | 0.05 | 134.62 | 42.78 |
| RP229 | O. SATIVA::IRGC 17083-1 | LL_S | DS | 76 | 81.79 | 22.16 | 42.49 | 35 | 4128.76 | 282.51 | 0.08 | 151.77 | 49.91 |
| RP230 | PAH WEAN::IRGC 78276-1 | LL_S | DS | 0 | 0.00 | 0.00 | 0.00 | 0 | 0.00 | 0.00 | 0.00 | 0.00 | 0.00 |
| RP231 | PAI CHUEH CHIU LIU::IRGC 34259-1 | LL_S | DS | 104 | 54.35 | 17.13 | 39.54 | 20 | 4354.37 | 96.11 | 0.06 | 128.91 | 53.23 |
| RP232 | PAI YI PING::IRGC 1368-1 | LL_S | DS | 80 | 64.42 | 19.35 | 28.37 | 13 | 3363.81 | 72.53 | 0.05 | 144.20 | 58.02 |
| RP233 | PALEPYU::IRGC 33549-1 | LL_S | DS | 74 | 83.24 | 20.15 | 35.55 | 32 | 4874.63 | 353.29 | 0.11 | 151.77 | 48.96 |
| **Study designation** | **3KG database designation** | **Env** | **Season** | **DTF** | **PH (cm)** | **PL (cm)** | **FlgLA (cm^2^)** | **NBP** | **BMDW (kgha^-1^)** | **GY (kgha^-1^)** | **HI** | **TGW (g)** | **SPKFT (%)** |
| RP234 | PARA NELLU::IRGC 50009-1 | LL_S | DS | 68 | 89.83 | 19.40 | 33.80 | 20 | 3739.18 | 635.06 | 0.06 | 148.91 | 44.98 |
| RP235 | PATISAIL::IRGC 37562-1 | LL_S | DS | 0 | 0.00 | 0.00 | 0.00 | 0 | 0.00 | 0.00 | 0.00 | 0.00 | 0.00 |
| RP237 | PERUNEL::IRGC 63113-1 | LL_S | DS | 76 | 90.90 | 21.43 | 39.23 | 28 | 4761.25 | 211.43 | 0.09 | 157.48 | 53.91 |
| RP238 | PICO NEGRO::IRGC 55849-1 | LL_S | DS | 0 | 0.00 | 0.00 | 0.00 | 0 | 0.00 | 0.00 | 0.00 | 0.00 | 0.00 |
| RP239 | PLI KHAO::IRGC 64596-1 | LL_S | DS | 0 | 0.00 | 0.00 | 0.00 | 0 | 0.00 | 0.00 | 0.00 | 0.00 | 0.00 |
| RP240 | PODI HEENATI::IRGC 36345-1 | LL_S | DS | 84 | 62.52 | 15.51 | 28.03 | 24 | 4805.96 | 148.82 | 0.05 | 134.62 | 50.86 |
| RP241 | PSBRC 50::IRGC 99706-1 | LL_S | DS | NA | 52.43 | 15.60 | 20.76 | 9 | 3206.49 | 40.19 | 0.02 | 0.00 | 29.91 |
| RP242 | PSBRC 68::IRGC 99711-1 | LL_S | DS | 98 | 67.90 | 16.90 | 29.39 | 19 | 4499.39 | 223.90 | 0.05 | 148.91 | 36.00 |
| RP243 | PSBRC 88::IRGC 99717-1 | LL_S | DS | 0 | 0.00 | 0.00 | 0.00 | 0 | 0.00 | 0.00 | 0.00 | 0.00 | 0.00 |
| RP244 | PULUT BARAYA::IRGC 27393-1 | LL_S | DS | 0 | 0.00 | 0.00 | 0.00 | 0 | 0.00 | 0.00 | 0.00 | 0.00 | 0.00 |
| RP245 | PURA BINNI::IRGC 26772-1 | LL_S | DS | 0 | 0.00 | 0.00 | 0.00 | 0 | 0.00 | 0.00 | 0.00 | 0.00 | 0.00 |
| RP247 | QUERO ASSAN::IRGC 28860-1 | LL_S | DS | 0 | 0.00 | 0.00 | 0.00 | 0 | 0.00 | 0.00 | 0.00 | 0.00 | 0.00 |
| RP248 | RACE PERUMAL::IRGC 55347-1 | LL_S | DS | 71 | 69.56 | 18.20 | 28.37 | 27 | 3297.45 | 225.42 | 0.08 | 151.77 | 45.23 |
| RP250 | RELLY::IRGC 14623-1 | LL_S | DS | 0 | 0.00 | 0.00 | 0.00 | 0 | 0.00 | 0.00 | 0.00 | 0.00 | 0.00 |
| RP251 | RIZ TYPE SORGHO::IRGC 69015-1 | LL_S | DS | 86 | 67.01 | 17.63 | 30.17 | 10 | 3393.40 | 80.97 | 0.03 | 157.48 | 43.10 |
| RP252 | ROJOFOTSY::IRGC 69402-1 | LL_S | DS | 0 | 0.00 | 0.00 | 0.00 | 0 | 0.00 | 0.00 | 0.00 | 0.00 | 0.00 |
| RP253 | RPA 5929 (K 45)::IRGC 33963-1 | LL_S | DS | NA | 51.94 | 15.72 | 21.07 | 5 | 3090.93 | 59.22 | 0.03 | 120.86 | 58.11 |
| RP254 | RPW 9-4 (SS 1)::IRGC 50690-1 | LL_S | DS | 105 | 49.24 | 15.54 | 20.28 | 6 | 2729.66 | 56.17 | 0.03 | 0.00 | 45.04 |
| RP255 | RR 272-17-829::IRGC 117354-1 | LL_S | DS | 97 | 53.68 | 16.54 | 32.25 | 12 | 2944.11 | 102.84 | 0.03 | 123.19 | 33.93 |
| RP256 | RTS 16::IRGC 8235-1 | LL_S | DS | 79 | 71.67 | 18.57 | 33.01 | 18 | 3430.04 | 95.86 | 0.03 | 120.34 | 30.87 |
| RP257 | RUSTIC::IRGC 117026-1 | LL_S | DS | NA | 49.28 | 17.35 | 23.61 | 13 | 3364.35 | 81.72 | 0.03 | 130.86 | 30.67 |
| RP258 | SADA RUPA::IRGC 77299-1 | LL_S | DS | 108 | 69.58 | 16.74 | 28.73 | 9 | 3675.59 | 93.94 | 0.03 | 147.53 | 33.25 |
| RP259 | SAN CHIAO TSWEN::IRGC 1565-1 | LL_S | DS | 0 | 0.00 | 0.00 | 0.00 | 0 | 0.00 | 0.00 | 0.00 | 0.00 | 0.00 |
| RP260 | SAN SHIH TSI::IRGC 1038-1 | LL_S | DS | 96 | 58.80 | 15.03 | 26.08 | 7 | 3228.39 | 59.35 | 0.03 | 120.39 | 36.57 |
| RP261 | SIGARDIS::IRGC 15555-1 | LL_S | DS | 0 | 0.00 | 0.00 | 0.00 | 0 | 0.00 | 0.00 | 0.00 | 0.00 | 0.00 |
| RP262 | SIMET 2::IRGC 25734-1 | LL_S | DS | 0 | 0.00 | 0.00 | 0.00 | 0 | 0.00 | 0.00 | 0.00 | 0.00 | 0.00 |
| RP263 | SIMUL KHURI::IRGC 35154-1 | LL_S | DS | 76 | 79.10 | 17.37 | 36.03 | 22 | 3536.12 | 343.70 | 0.16 | 143.19 | 62.84 |
| RP264 | SINNA SITHIRA KALI::IRGC 51064-1 | LL_S | DS | NA | 69.48 | 16.36 | 23.80 | 6 | 3005.48 | 55.03 | 0.03 | 134.62 | 42.46 |
| **Study designation** | **3KG database designation** | **Env** | **Season** | **DTF** | **PH (cm)** | **PL (cm)** | **FlgLA (cm^2^)** | **NBP** | **BMDW (kgha^-1^)** | **GY (kgha^-1^)** | **HI** | **TGW (g)** | **SPKFT (%)** |
| RP266 | SITHAIYAN KOTTAI SAMBA::IRGC 50155-1 | LL_S | DS | 0 | 0.00 | 0.00 | 0.00 | 0 | 0.00 | 0.00 | 0.00 | 0.00 | 0.00 |
| RP267 | SML AWINI::IRGC 13391-1 | LL_S | DS | 98 | 53.79 | 16.13 | 29.73 | 7 | 3158.51 | 78.13 | 0.03 | 134.20 | 30.15 |
| RP268 | SOLOMON RED RICE::IRGC 50950-1 | LL_S | DS | NA | 76.17 | 18.02 | 30.15 | 12 | 4206.01 | 58.51 | 0.02 | 143.73 | 27.51 |
| RP269 | SONA::IRGC 26971-C1 | LL_S | DS | NA | 59.18 | 17.80 | 24.94 | 5 | 3137.76 | 75.60 | 0.03 | 0.00 | 27.27 |
| RP270 | SONAMUKHI::IRGC 46693-1 | LL_S | DS | 70 | 85.52 | 19.77 | 30.96 | 30 | 4458.65 | 544.16 | 0.14 | 140.34 | 57.21 |
| RP272 | SSANGDUJO::IRGC 55632-1 | LL_S | DS | 100 | 76.10 | 18.11 | 28.86 | 11 | 4064.00 | 84.30 | 0.03 | 134.20 | 34.92 |
| RP273 | SUFAID 246::IRGC 28303-1 | LL_S | DS | 81 | 80.04 | 18.05 | 37.86 | 19 | 3879.71 | 149.26 | 0.07 | 143.19 | 53.31 |
| RP274 | SUWEON 311::IRGC 61890-1 | LL_S | DS | 90 | 47.62 | 16.85 | 23.66 | 18 | 2925.92 | 73.20 | 0.05 | 143.19 | 45.01 |
| RP275 | T 315::IRGC 54792-1 | LL_S | DS | 87 | 84.72 | 20.08 | 34.97 | 23 | 4759.97 | 141.60 | 0.07 | 151.77 | 51.16 |
| RP276 | TAIPEI WOO CO::IRGC 112-1 | LL_S | DS | 0 | 0.00 | 0.00 | 0.00 | 0 | 0.00 | 0.00 | 0.00 | 0.00 | 0.00 |
| RP277 | TAITUNG WOO LI::IRGC 111-1 | LL_S | DS | 0 | 0.00 | 0.00 | 0.00 | 0 | 0.00 | 0.00 | 0.00 | 0.00 | 0.00 |
| RP278 | TAK SUFAID::IRGC 73127-1 | LL_S | DS | NA | 54.68 | 15.50 | 29.14 | 9 | 3348.82 | 57.37 | 0.02 | 120.39 | 33.59 |
| RP279 | TAK::IRGC 73124-1 | LL_S | DS | 101 | 55.39 | 14.24 | 20.37 | 6 | 2596.55 | 46.10 | 0.02 | 133.73 | 18.73 |
| RP280 | TIKAL 3::IRGC 50649-1 | LL_S | DS | 103 | 57.30 | 17.22 | 23.42 | 13 | 3911.74 | 57.45 | 0.03 | 147.06 | 25.96 |
| RP281 | TOC 5430::IRGC 70487-1 | LL_S | DS | 88 | 83.54 | 19.70 | 36.92 | 9 | 4272.42 | 109.92 | 0.06 | 147.53 | 48.96 |
| RP282 | TONG GU HONG::IRGC 81026-1 | LL_S | DS | 67 | 76.94 | 20.24 | 34.33 | 36 | 3812.97 | 433.69 | 0.11 | 146.05 | 59.09 |
| RP283 | TSAI YUAN CHON::IRGC 126-1 | LL_S | DS | NA | 57.16 | 16.54 | 30.93 | 7 | 3250.49 | 87.14 | 0.04 | 140.34 | 50.15 |
| RP284 | TSAO SHENG LI 1::IRGC 1309-1 | LL_S | DS | 105 | 69.40 | 18.00 | 33.46 | 16 | 4714.49 | 104.40 | 0.07 | 148.91 | 54.25 |
| RP285 | UPRH 233::IRGC 61667-1 | LL_S | DS | 75 | 52.43 | 19.30 | 31.23 | 21 | 3484.10 | 286.02 | 0.10 | 143.19 | 52.30 |
| RP286 | UQUIHUA::IRGC 117037-1 | LL_S | DS | NA | 51.73 | 16.25 | 23.31 | 5 | 4050.49 | 51.08 | 0.02 | 0.00 | 29.56 |
| RP287 | URAIBOOL::IRGC 52785-1 | LL_S | DS | NA | 67.06 | 16.29 | 26.54 | 4 | 3458.91 | 81.41 | 0.03 | 0.00 | 30.13 |
| RP288 | VEN THAP::IRGC 56138-1 | LL_S | DS | 75 | 78.95 | 19.66 | 36.44 | 27 | 4577.92 | 189.71 | 0.06 | 148.91 | 49.72 |
| RP289 | WANGA BARUGULU::IRGC 52261-1 | LL_S | DS | 79 | 79.35 | 19.87 | 34.56 | 27 | 4918.83 | 360.18 | 0.16 | 148.91 | 61.08 |
| RP290 | WAR 72-2-1-1::IRGC 117361-1 | LL_S | DS | 102 | 65.92 | 17.68 | 34.97 | 5 | 4113.39 | 76.61 | 0.02 | 147.53 | 41.96 |
| RP291 | WI BIR SHUN::IRGC 4602-1 | LL_S | DS | 85 | 78.95 | 16.69 | 31.54 | 13 | 3569.17 | 60.33 | 0.03 | 146.05 | 30.58 |
| RP292 | WP 36::IRGC 55278-1 | LL_S | DS | 103 | 67.33 | 17.20 | 23.83 | 8 | 3976.68 | 81.48 | 0.03 | 134.20 | 33.27 |
| RP293 | WP 65::IRGC 36526-1 | LL_S | DS | NA | 53.27 | 16.55 | 29.20 | 4 | 3530.13 | 89.23 | 0.03 | 0.00 | NA |
| RP294 | XI GAN JING REN::IRGC 60035-1 | LL_S | DS | 59 | 73.99 | 20.08 | 28.87 | 30 | 3923.78 | 522.94 | 0.20 | 160.34 | 59.58 |
| **Study designation** | **3KG database designation** | **Env** | **Season** | **DTF** | **PH (cm)** | **PL (cm)** | **FlgLA (cm^2^)** | **NBP** | **BMDW (kgha^-1^)** | **GY (kgha^-1^)** | **HI** | **TGW (g)** | **SPKFT (%)** |
| RP295 | XIA ZHI BAI::IRGC 53437-1 | LL_S | DS | 61 | 78.14 | 17.99 | 29.99 | 41 | 4207.29 | 580.16 | 0.21 | 137.48 | 59.70 |
| RP296 | XITTO::IRGC 6671-1 | LL_S | DS | 79 | 88.89 | 22.06 | 38.19 | 19 | 5831.75 | 179.23 | 0.05 | 157.48 | 42.92 |
| RP297 | YA NONG ZAO 4::IRGC 63908-1 | LL_S | DS | NA | 55.35 | 16.86 | 29.25 | 5 | 3120.62 | 65.90 | 0.03 | 147.53 | 27.81 |
| RP298 | YEBAWYIN::IRGC 33885-1 | LL_S | DS | 66 | 81.62 | 20.70 | 38.34 | 24 | 4327.98 | 723.86 | 0.20 | 166.05 | 58.29 |
| RP299 | YONG JIN ZAO 3::IRGC 70441-1 | LL_S | DS | 74 | 54.87 | 20.70 | 34.02 | 32 | 3751.09 | 331.77 | 0.22 | 146.05 | 56.76 |
| RP300 | H 6::IRGC 157-1 | LL_S | DS | NA | 88.73 | 20.53 | 35.30 | 6 | 4014.05 | 74.76 | 0.03 | 114.62 | 40.42 |
| RP002 | 19::IRGC 70786-1 | LL_N | WS | 91 | 143.97 | 21.54 | 40.13 | 65 | 18619.69 | 2081.28 | 0.05 | 190.00 | 81.74 |
| RP003 | 3210::IRGC 116950-1 | LL_N | WS | 94 | 81.57 | 17.96 | 37.76 | 42 | 13643.61 | 957.65 | 0.12 | 220.00 | 89.77 |
| RP004 | 498-2A BR 8::IRGC 5891-1 | LL_N | WS | 91 | 125.05 | 23.54 | 34.02 | 47 | 14496.31 | 1704.09 | 0.17 | 240.00 | 85.94 |
| RP005 | ADT 12::IRGC 6254-1 | LL_N | WS | 91 | 158.34 | 27.13 | 52.47 | 55 | 15709.32 | 2112.25 | 0.08 | 250.00 | 88.28 |
| RP006 | AE NOUA::IRGC 89308-1 | LL_N | WS | 88 | 131.02 | 22.69 | 40.74 | 35 | 14242.95 | 1455.54 | 0.08 | 260.00 | 83.24 |
| RP007 | ALTAMIRA 9::IRGC 116953-1 | LL_N | WS | 99 | 91.66 | 16.47 | 26.48 | 23 | 15246.97 | 747.22 | 0.09 | 210.00 | 85.82 |
| RP008 | ARAURE 1::IRGC 116956-1 | LL_N | WS | 91 | 100.60 | 24.83 | 33.50 | 57 | 25135.54 | 886.18 | 0.03 | 270.00 | 87.15 |
| RP009 | ARC 10100::IRGC 20709-1 | LL_N | WS | 89 | 114.49 | 21.77 | 43.14 | 32 | 15438.13 | 1861.32 | 0.14 | 160.00 | 87.52 |
| RP010 | ARC 10594::IRGC 12524-1 | LL_N | WS | 91 | 145.53 | 24.89 | 43.48 | 56 | 17928.79 | 1692.86 | 0.06 | NA | 89.27 |
| RP011 | ARC 10754::IRGC 12603-1 | LL_N | WS | 92 | 126.66 | 26.12 | 38.55 | 55 | 11794.22 | 2601.41 | 0.64 | 200.00 | 90.04 |
| RP012 | ARC 10812::IRGC 21074-1 | LL_N | WS | 93 | 150.99 | 24.98 | 39.98 | 48 | 13841.06 | 804.39 | 0.25 | NA | 87.79 |
| RP013 | ARC 11524::IRGC 42672-1 | LL_N | WS | 92 | 141.86 | 22.20 | 41.16 | 35 | 13916.19 | 2402.89 | 0.25 | 250.00 | 87.58 |
| RP014 | ARC 11857::IRGC 40972-1 | LL_N | WS | 86 | 138.69 | 23.18 | 30.46 | 56 | 13272.12 | 2186.89 | 0.26 | 220.00 | 86.98 |
| RP015 | ARC 11901::IRGC 21727-1 | LL_N | WS | 92 | 153.95 | 27.24 | 53.66 | 48 | 22642.78 | 2431.47 | 0.11 | 220.00 | 88.41 |
| RP018 | ARC 13778::IRGC 41216-1 | LL_N | WS | 88 | 143.67 | 23.18 | 40.31 | 48 | 24341.55 | 1212.55 | 0.01 | 250.00 | 80.05 |
| RP019 | ARC 14060::IRGC 41374-1 | LL_N | WS | 89 | 110.91 | 24.75 | 41.83 | 61 | 15516.76 | 1537.33 | 0.14 | 180.00 | 87.15 |
| RP020 | ARC 14064::IRGC 41377-1 | LL_N | WS | 84 | 141.80 | 26.84 | 29.19 | 34 | 15559.74 | 2242.48 | 0.11 | 260.00 | 86.84 |
| RP021 | ARC 14654::IRGC 41663-1 | LL_N | WS | 86 | 109.35 | 23.26 | 32.88 | 61 | 16088.49 | 1644.53 | 0.15 | 190.00 | 88.16 |
| RP022 | ARC 15873::IRGC 43250-1 | LL_N | WS | 86 | 139.30 | 24.70 | 54.74 | 65 | 11647.09 | 806.77 | 0.15 | 200.00 | 84.05 |
| RP023 | ARC 18092::IRGC 42256-1 | LL_N | WS | 92 | 144.59 | 22.83 | 39.93 | 55 | 20111.23 | 2136.07 | 0.09 | 240.00 | 88.68 |
| RP024 | ARC 18112::IRGC 42274-1 | LL_N | WS | 91 | 131.95 | 22.46 | 38.81 | 58 | 16404.76 | 2402.09 | 0.22 | 230.00 | 89.40 |
| RP025 | ARC 18202::IRGC 42328-1 | LL_N | WS | 88 | 124.02 | 23.54 | 36.03 | 51 | 16875.84 | 1970.90 | 0.13 | 200.00 | 88.16 |
| **Study designation** | **3KG database designation** | **Env** | **Season** | **DTF** | **PH (cm)** | **PL (cm)** | **FlgLA (cm^2^)** | **NBP** | **BMDW (kgha^-1^)** | **GY (kgha^-1^)** | **HI** | **TGW (g)** | **SPKFT (%)** |
| RP026 | ARC 18597::IRGC 43299-1 | LL_N | WS | 92 | 142.57 | 23.54 | 42.38 | 49 | 13209.57 | 1407.90 | 0.12 | 210.00 | 88.41 |
| RP028 | ASHI BINNI::IRGC 77216-1 | LL_N | WS | 94 | 131.51 | 28.33 | 48.92 | 61 | 22154.92 | 3356.58 | 0.17 | 230.00 | 90.87 |
| RP029 | ASHMBER::IRGC 27522-1 | LL_N | WS | 91 | 121.82 | 24.83 | 35.73 | 61 | 14210.10 | 3346.26 | 0.43 | 260.00 | 88.52 |
| RP030 | ASU::IRGC 62154-1 | LL_N | WS | 90 | 131.20 | 21.71 | 46.19 | 49 | 16692.37 | 2110.66 | 0.19 | 210.00 | 89.10 |
| RP031 | AUS 171::IRGC 29004-1 | LL_N | WS | 89 | 120.90 | 24.12 | 37.17 | 43 | 12178.28 | 1785.88 | 0.40 | 60.00 | 90.28 |
| RP032 | AUS 177::IRGC 29009-1 | LL_N | WS | 93 | 107.80 | 21.71 | 37.51 | 63 | 19159.27 | 1118.06 | 0.08 | 220.00 | 85.61 |
| RP033 | AUS 219::IRGC 29031-1 | LL_N | WS | 87 | 106.37 | 18.96 | 32.86 | 41 | 13553.79 | 2092.40 | 0.32 | 240.00 | 89.57 |
| RP034 | AUS 233::IRGC 29036-1 | LL_N | WS | 87 | 125.05 | 21.11 | 28.29 | 30 | 16187.04 | 1295.14 | 0.06 | 230.00 | 88.25 |
| RP036 | AUS 295::IRGC 29083-1 | LL_N | WS | 89 | 132.12 | 22.69 | 34.69 | 42 | 18006.72 | 2714.17 | 0.08 | 240.00 | 87.54 |
| RP037 | AUS 301::IRGC 29089-1 | LL_N | WS | 87 | 119.74 | 20.40 | 30.93 | 84 | 14844.38 | 1679.47 | 0.10 | 250.00 | 81.77 |
| RP038 | AUS 308::IRGC 29096-1 | LL_N | WS | 90 | 126.34 | 21.00 | 32.97 | 55 | 15033.79 | 1808.12 | 0.18 | NA | 88.04 |
| RP039 | AUS 329::IRGC 29116-1 | LL_N | WS | 89 | 114.44 | 19.25 | 41.35 | 27 | 14891.21 | 1171.26 | 0.06 | 240.00 | 88.13 |
| RP040 | AUS 344::IRGC 29131-1 | LL_N | WS | 87 | 105.08 | 19.28 | 25.90 | 71 | 16554.33 | 1378.52 | 0.16 | 260.00 | 90.12 |
| RP041 | AUS 439::IRGC 29221-1 | LL_N | WS | 93 | 109.65 | 23.41 | 44.30 | 35 | 11111.71 | 971.94 | 0.25 | NA | 86.73 |
| RP042 | AUS PADDY (RED)::IRGC 44978-1 | LL_N | WS | 86 | 105.74 | 19.88 | 29.88 | 44 | 10976.11 | 2046.34 | 0.00 | 250.00 | 88.28 |
| RP043 | B 4414 F-MR-6-3::IRGC 117310-1 | LL_N | WS | 94 | 99.82 | 21.11 | 43.96 | 46 | 15891.04 | 1056.12 | 0.09 | 230.00 | 85.92 |
| RP044 | B 6136 E-3-TB-0-1-5::IRGC 117311-1 | LL_N | WS | 91 | 101.55 | 18.02 | 26.58 | 56 | 12922.30 | 1578.63 | 0.36 | 220.00 | 88.59 |
| RP045 | B 6136-3-TB-0-1-5::IRGC 117312-1 | LL_N | WS | 91 | 99.77 | 21.26 | 37.93 | 46 | 21890.02 | 2112.25 | 0.09 | 230.00 | 89.80 |
| RP046 | B 6149 F-MR-7::IRGC 117314-1 | LL_N | WS | 92 | 138.51 | 25.18 | 49.88 | 32 | 14587.17 | 1777.94 | 0.08 | 270.00 | 85.53 |
| RP047 | BA BAI GU::IRGC 79580-1 | LL_N | WS | 87 | 130.40 | 19.97 | 26.98 | 50 | 16937.00 | 1361.84 | 0.16 | 170.00 | 89.40 |
| RP048 | BA SHI ZAO::IRGC 67903-1 | LL_N | WS | 87 | 120.90 | 22.69 | 26.72 | 51 | 17422.06 | 1301.49 | 0.07 | 230.00 | 88.06 |
| RP049 | BADAL 1163::IRGC 32796-1 | LL_N | WS | 88 | 144.92 | 21.40 | 31.11 | 69 | 11764.16 | 1249.08 | 0.54 | 240.00 | 85.29 |
| RP050 | BADUIE::IRGC 53715-1 | LL_N | WS | 93 | 150.84 | 27.93 | 43.13 | 55 | 15318.96 | 2972.24 | 0.35 | 170.00 | 87.98 |
| RP051 | BAI HE::IRGC 76438-1 | LL_N | WS | 88 | 120.06 | 22.17 | 35.29 | 39 | 12420.82 | 1272.90 | 0.19 | 200.00 | 88.04 |
| RP052 | BAIANG 6::IRGC 6129-1 | LL_N | WS | 88 | 97.81 | 22.12 | 32.23 | 60 | 21908.19 | 1487.31 | 0.03 | 240.00 | 85.21 |
| RP053 | BAK TULSI::IRGC 34831-1 | LL_N | WS | 88 | 114.80 | 21.83 | 36.05 | 26 | 12040.94 | 1596.89 | 0.31 | 280.00 | 88.58 |
| RP054 | BAKASI::IRGC 27074-1 | LL_N | WS | 91 | 109.49 | 19.11 | 40.29 | 44 | 13795.98 | 1110.91 | 0.11 | 210.00 | 87.16 |
| RP055 | BALASURIYA A::IRGC 66509-1 | LL_N | WS | 88 | 99.05 | 18.82 | 20.13 | 39 | 12297.10 | 2522.79 | 0.53 | 230.00 | 87.05 |
| **Study designation** | **3KG database designation** | **Env** | **Season** | **DTF** | **PH (cm)** | **PL (cm)** | **FlgLA (cm^2^)** | **NBP** | **BMDW (kgha^-1^)** | **GY (kgha^-1^)** | **HI** | **TGW (g)** | **SPKFT (%)** |
| RP056 | BAMOA A 75::IRGC 51101-1 | LL_N | WS | 91 | 78.92 | 16.24 | 33.50 | 56 | 23902.96 | 654.31 | 0.01 | 260.00 | 86.85 |
| RP057 | BANDI::IRGC 17214-1 | LL_N | WS | 98 | 109.65 | 19.97 | 38.33 | 46 | 18295.74 | 568.55 | 0.03 | NA | 83.77 |
| RP058 | BANGKOUY::IRGC 94037-1 | LL_N | WS | 93 | 127.85 | 25.41 | 53.71 | 38 | 15270.03 | 1542.10 | 0.16 | 350.00 | 84.34 |
| RP059 | BARIK KUDI::IRGC 52807-1 | LL_N | WS | 85 | 97.18 | 17.99 | 27.23 | 42 | 18914.99 | 586.81 | 0.01 | 100.00 | 85.23 |
| RP060 | BAT DO::IRGC 7014-1 | LL_N | WS | 90 | 135.88 | 21.40 | 39.36 | 41 | 11819.03 | 1708.86 | 0.24 | 190.00 | 87.91 |
| RP061 | BATHURI::IRGC 25838-1 | LL_N | WS | 86 | 115.42 | 19.16 | 35.83 | 63 | 14326.47 | 2600.61 | 0.37 | 260.00 | 89.23 |
| RP062 | BAZAIL::IRGC 27526-1 | LL_N | WS | 92 | 91.09 | 23.11 | 39.68 | 50 | 16779.39 | 2077.31 | 0.14 | 220.00 | 87.83 |
| RP063 | BENGALY MORIMO::IRGC 10976-1 | LL_N | WS | 93 | 93.74 | 20.94 | 36.35 | 53 | 15867.63 | 1219.70 | 0.17 | 260.00 | 86.62 |
| RP065 | BHADOIA 303::IRGC 6588-1 | LL_N | WS | 86 | 123.39 | 16.96 | 21.83 | NA | 15402.00 | 1692.86 | 0.00 | NA | 86.85 |
| RP066 | BK 26::IRGC 45197-1 | LL_N | WS | 91 | 120.88 | 23.69 | 39.26 | 60 | 15559.74 | 2209.13 | 0.17 | 190.00 | 88.52 |
| RP067 | BKN BR 1031-78-5-4::IRGC 55927-1 | LL_N | WS | 93 | 150.54 | 27.21 | 38.71 | 74 | 13374.87 | 3709.95 | 0.45 | 270.00 | 90.29 |
| RP068 | BONG SEN::IRGC 7011-1 | LL_N | WS | 92 | 141.59 | 21.54 | 43.14 | 60 | 16256.24 | 1560.36 | 0.20 | 220.00 | 86.79 |
| RP070 | BPI 76 NON SENSITIVE (GREEN)::IRGC 9790-1 | LL_N | WS | 91 | 119.96 | 25.41 | 35.17 | 33 | 15767.68 | 1574.66 | 0.15 | 190.00 | 89.09 |
| RP071 | BR 51-115-4::IRGC 43999-1 | LL_N | WS | 91 | 112.62 | 21.54 | 47.47 | 48 | 16889.47 | 2459.27 | 0.22 | 190.00 | 87.15 |
| RP072 | BR 5230-46-4::IRGC 117318-1 | LL_N | WS | 86 | 95.00 | 20.11 | 35.04 | 36 | 13099.48 | 1308.64 | 0.29 | 210.00 | 79.59 |
| RP073 | BR IRGA 409::IRGC 55915-1 | LL_N | WS | 91 | 91.39 | 23.95 | 22.19 | 36 | 15361.95 | 1338.81 | 0.15 | 200.00 | 83.82 |
| RP074 | BW 295-5::IRGC 63098-1 | LL_N | WS | 93 | 92.50 | 20.40 | 32.04 | 61 | 14534.75 | 1639.77 | 0.24 | 240.00 | 87.46 |
| RP075 | C 1016-1::IRGC 50368-1 | LL_N | WS | 91 | 125.26 | 23.20 | 40.06 | 45 | 13339.92 | 2885.69 | 0.52 | 220.00 | 89.08 |
| RP076 | C 166-135::IRGC 50633-1 | LL_N | WS | 93 | 120.89 | 24.15 | 44.07 | 39 | 15402.00 | 2005.05 | 0.07 | 250.00 | 89.83 |
| RP077 | C 662083::IRGC 62101-1 | LL_N | WS | 91 | 147.25 | 28.70 | 34.72 | 53 | 14540.34 | 3445.52 | 0.27 | 160.00 | 89.42 |
| RP079 | CAUVERY::IRGC 45255-1 | LL_N | WS | 89 | 78.15 | 18.39 | 25.34 | 71 | 12715.42 | 1800.18 | 0.20 | 210.00 | 85.06 |
| RP080 | CE IN TSAN::IRGC 4362-1 | LL_N | WS | 93 | 116.98 | 19.82 | 45.75 | 48 | 13487.39 | 823.45 | 0.09 | 180.00 | 84.19 |
| RP081 | CEA 3::IRGC 116965-1 | LL_N | WS | 93 | 67.95 | 14.75 | 23.72 | 66 | 12829.69 | 964.00 | 0.18 | 190.00 | 80.35 |
| RP082 | CEMPO MANGGAR::IRGC 27107-1 | LL_N | WS | 99 | 117.47 | 19.82 | 44.05 | 35 | 15235.09 | 836.16 | 0.03 | 50.00 | 86.85 |
| RP083 | CHAKOL::IRGC 77226-1 | LL_N | WS | 88 | 116.22 | 24.98 | 44.49 | 48 | 16012.31 | 552.67 | 0.04 | 120.00 | 86.85 |
| RP084 | CHAM LEK::IRGC 89387-1 | LL_N | WS | 92 | 148.76 | 25.13 | 31.10 | 43 | 23026.14 | 1880.38 | 0.07 | 250.00 | 87.94 |
| RP085 | CHAMA (DWARF)::IRGC 69487-1 | LL_N | WS | 93 | 76.58 | 20.25 | 38.77 | 61 | 13134.43 | 1048.18 | 0.22 | 160.00 | 90.15 |
| RP086 | CHANDARHAT::IRGC 25845-1 | LL_N | WS | 89 | 119.95 | 22.26 | 56.25 | 44 | 12852.06 | 1692.86 | 0.17 | 270.00 | 86.69 |
| **Study designation** | **3KG database designation** | **Env** | **Season** | **DTF** | **PH (cm)** | **PL (cm)** | **FlgLA (cm^2^)** | **NBP** | **BMDW (kgha^-1^)** | **GY (kgha^-1^)** | **HI** | **TGW (g)** | **SPKFT (%)** |
| RP087 | CHANDINA::IRGC 36420-1 | LL_N | WS | 88 | 67.37 | 19.25 | 26.34 | 36 | 12463.45 | 1190.32 | 0.12 | 200.00 | 87.48 |
| RP088 | CHANG LE SAN SHU ZAO::IRGC 63561-1 | LL_N | WS | 91 | 68.16 | 16.10 | 28.04 | 63 | 11868.31 | 983.86 | 0.24 | 220.00 | 82.43 |
| RP089 | CHAO PEUAK DENG::IRGC 11602-1 | LL_N | WS | 90 | 158.34 | 27.13 | 47.65 | 40 | 14849.27 | 2968.27 | 0.26 | 250.00 | 88.46 |
| RP090 | CHI SHENG TAO::IRGC 4606-1 | LL_N | WS | 86 | 107.62 | 17.01 | 28.80 | 63 | 13425.19 | 2853.93 | 0.28 | 240.00 | 87.13 |
| RP091 | CHIAYI WU-K'O::IRGC 64974-1 | LL_N | WS | 93 | 148.96 | 21.34 | 33.41 | 39 | 11540.15 | 1588.15 | 0.60 | 260.00 | 88.25 |
| RP092 | CHIH SHEN LI::IRGC 1306-1 | LL_N | WS | 92 | 133.99 | 24.38 | 44.24 | 57 | 12278.93 | 1335.64 | 0.40 | 230.00 | 87.49 |
| RP093 | CHNNOR::IRGC 67485-1 | LL_N | WS | 89 | 137.73 | 24.70 | 45.73 | 58 | 15133.04 | 1280.05 | 0.11 | 240.00 | 90.35 |
| RP094 | CHUA DAU::IRGC 4785-1 | LL_N | WS | 89 | 135.97 | 19.68 | 26.04 | 38 | 18327.54 | 1601.65 | 0.10 | 230.00 | 88.72 |
| RP095 | CHUNGUR BALI::IRGC 25855-1 | LL_N | WS | 86 | 123.71 | 22.69 | 28.45 | 78 | 15531.44 | 1083.12 | 0.03 | 200.00 | 90.73 |
| RP096 | CICA 9::IRGC 53079-1 | LL_N | WS | 94 | 99.19 | 21.14 | 49.32 | 50 | 15480.42 | 2373.50 | 0.24 | 240.00 | 86.37 |
| RP097 | CN 44-40-7::IRGC 45368-1 | LL_N | WS | 89 | 82.83 | 17.31 | 25.52 | 44 | 15402.00 | 1252.26 | 0.03 | 180.00 | 89.66 |
| RP098 | CO 39::IRGC 51231-1 | LL_N | WS | 87 | 79.49 | 20.54 | 24.97 | 28 | 11755.08 | 876.65 | 0.13 | NA | 88.80 |
| RP099 | CR 60-10::IRGC 15777-1 | LL_N | WS | 89 | 86.56 | 21.11 | 38.22 | 53 | 12663.35 | 1426.96 | 0.27 | 230.00 | 87.41 |
| RP100 | CRILLO LA FRIA::IRGC 10793-1 | LL_N | WS | 92 | 127.29 | 20.02 | 31.05 | 47 | 13166.58 | 1448.40 | 0.15 | NA | 86.95 |
| RP101 | CSR-90 IR-2::IRGC 117327-1 | LL_N | WS | 88 | 111.85 | 23.69 | 34.67 | 63 | 17916.56 | 1146.64 | 0.02 | 300.00 | 79.93 |
| RP102 | CT 9737-6-1-1-2-2P-M::IRGC 117330-1 | LL_N | WS | 93 | 90.41 | 19.97 | 31.30 | 49 | 11558.68 | 1074.38 | 0.18 | 210.00 | 87.58 |
| RP103 | CUN GU NUO::IRGC 63576-1 | LL_N | WS | 90 | 92.50 | 23.18 | 35.73 | 28 | 21947.33 | 808.36 | 0.02 | 170.00 | 84.97 |
| RP104 | CUYAMEL 3820::IRGC 116975-1 | LL_N | WS | 98 | 102.02 | 22.26 | 34.95 | 41 | 18004.28 | 652.72 | 0.02 | 180.00 | 83.92 |
| RP105 | DA 11::IRGC 6046-1 | LL_N | WS | 94 | 100.92 | 20.83 | 27.95 | 30 | 20557.15 | 970.36 | 0.01 | 150.00 | 82.68 |
| RP106 | DA GANG ZHAN::IRGC 67103-1 | LL_N | WS | 91 | 150.21 | 19.97 | 29.25 | 31 | 14217.78 | 2198.81 | 0.19 | NA | 89.26 |
| RP107 | DA NUO (ZHAN)::IRGC 72025-1 | LL_N | WS | 91 | 142.74 | 21.11 | 41.90 | 41 | 13248.01 | 1197.46 | 0.15 | 190.00 | 88.66 |
| RP108 | DAA MANSA::IRGC 67559-1 | LL_N | WS | 91 | 91.88 | 18.53 | 33.81 | 43 | 12130.41 | 1390.43 | 0.14 | 210.00 | 89.65 |
| RP109 | DANGAR::IRGC 76296-1 | LL_N | WS | 86 | 125.35 | 22.83 | 32.57 | 44 | 13519.55 | 1785.09 | 0.24 | 220.00 | 84.03 |
| RP110 | DHANE BURWA::IRGC 10105-1 | LL_N | WS | 88 | 125.72 | 26.55 | 49.36 | 38 | 10900.98 | 2131.31 | 0.00 | NA | 87.71 |
| RP111 | DISSI::IRGC 101346-1 | LL_N | WS | 91 | 122.75 | 23.89 | 49.77 | 53 | 10669.63 | 1895.47 | 0.52 | 260.00 | 85.67 |
| RP112 | DUDH KADAR::IRGC 67707-1 | LL_N | WS | 90 | 131.81 | 21.97 | 31.60 | 35 | 12540.68 | 2130.51 | 0.25 | 250.00 | 85.71 |
| RP113 | E 2024::IRGC 67958-1 | LL_N | WS | 89 | 66.38 | 14.95 | 22.55 | 62 | 11095.63 | 944.15 | 0.12 | 200.00 | 86.54 |
| RP114 | E 2040::IRGC 67968-1 | LL_N | WS | 87 | 81.05 | 21.11 | 32.42 | 34 | 17250.82 | 881.42 | 0.07 | 180.00 | 90.26 |
| **Study designation** | **3KG database designation** | **Env** | **Season** | **DTF** | **PH (cm)** | **PL (cm)** | **FlgLA (cm^2^)** | **NBP** | **BMDW (kgha^-1^)** | **GY (kgha^-1^)** | **HI** | **TGW (g)** | **SPKFT (%)** |
| RP115 | E DAW HAWM::IRGC 47938-1 | LL_N | WS | 94 | 145.38 | 24.61 | 43.06 | 39 | 14217.43 | 1248.29 | 0.23 | 280.00 | 87.17 |
| RP116 | ELONI::IRGC 116980-1 | LL_N | WS | 93 | 86.98 | 20.25 | 28.28 | 47 | 15587.00 | 1396.78 | 0.11 | 210.00 | 88.73 |
| RP117 | EPEAL 102::IRGC 78698-1 | LL_N | WS | 94 | 84.08 | 18.82 | 31.17 | 53 | 12524.26 | 637.63 | 0.06 | 170.00 | 83.94 |
| RP118 | ES 21::IRGC 56171-1 | LL_N | WS | 88 | 135.70 | 27.13 | 46.67 | 47 | 18368.43 | 545.52 | 0.01 | 230.00 | 78.76 |
| RP119 | EX FOILAEIN (NAPUTO)::IRGC 81675-1 | LL_N | WS | 93 | 104.14 | 18.11 | 34.43 | 61 | 20593.49 | 1253.05 | 0.07 | 210.00 | 87.25 |
| RP120 | FEI GAI 122::IRGC 63599-1 | LL_N | WS | 89 | 84.99 | 20.97 | 41.59 | NA | 15402.00 | 1692.86 | 0.00 | NA | 86.85 |
| RP121 | FONAIAP 2::IRGC 116985-1 | LL_N | WS | 89 | 124.63 | 23.89 | 49.50 | 41 | 14166.76 | 1310.22 | 0.08 | 230.00 | 84.67 |
| RP122 | FU ZAO XIAN::IRGC 63619-1 | LL_N | WS | 86 | 72.98 | 15.96 | 29.25 | 29 | 15402.00 | 1692.86 | 0.00 | NA | 85.11 |
| RP123 | G 25::IRGC 45733-1 | LL_N | WS | 88 | 118.71 | 23.78 | 40.61 | 51 | 19357.07 | 2104.31 | 0.17 | 280.00 | 90.52 |
| RP124 | GAO JIAO BAI::IRGC 68047-1 | LL_N | WS | 84 | 99.04 | 18.82 | 26.78 | 35 | 11666.31 | 1011.65 | 0.18 | NA | 88.13 |
| RP125 | GENIT::IRGC 3272-1 | LL_N | WS | 91 | 127.74 | 21.26 | 35.85 | 38 | 17899.09 | 1115.67 | 0.07 | 230.00 | 87.58 |
| RP126 | GOJOL GORIA::IRGC 26629-1 | LL_N | WS | 88 | 123.22 | 27.84 | 39.79 | 72 | 20696.94 | 1245.11 | 0.05 | 190.00 | 82.02 |
| RP127 | GOKULGANJA::IRGC 45701-1 | LL_N | WS | 97 | 68.79 | 19.68 | 23.87 | 37 | 21866.61 | 3987.88 | 0.07 | 300.00 | 88.48 |
| RP128 | GUI HUA ZAO::IRGC 68060-1 | LL_N | WS | 88 | 128.70 | 22.17 | 28.53 | 21 | 11846.64 | 840.92 | 0.08 | 240.00 | 85.70 |
| RP130 | HAWM KRUA::IRGC 64333-1 | LL_N | WS | 88 | 158.48 | 22.40 | 36.46 | 48 | 19905.04 | 2565.67 | 0.11 | 210.00 | 88.84 |
| RP131 | HD 10::IRGC 6638-1 | LL_N | WS | 93 | 150.63 | 21.46 | 28.65 | 51 | 11940.65 | 3332.76 | 0.00 | 240.00 | 90.42 |
| RP132 | HE GU TSAO::IRGC 51302-1 | LL_N | WS | 87 | 128.70 | 20.83 | 40.47 | 48 | 19279.49 | 873.48 | 0.03 | 190.00 | 86.18 |
| RP133 | HODARAWALA::IRGC 67631-1 | LL_N | WS | 92 | 131.65 | 21.97 | 35.02 | 56 | 13078.17 | 2122.57 | 0.27 | 200.00 | 89.79 |
| RP134 | HOLOI BASH (SOLOI BASH)::IRGC 64778-1 | LL_N | WS | 91 | 124.78 | 19.97 | 36.15 | 75 | 18098.98 | 1692.86 | 0.32 | 220.00 | 88.68 |
| RP135 | HTA 22::IRGC 45827-1 | LL_N | WS | 93 | 136.18 | 22.40 | 35.39 | 48 | 22072.44 | 1761.27 | 0.07 | 280.00 | 85.80 |
| RP136 | HUA LI ZAO::IRGC 80950-1 | LL_N | WS | 93 | 130.86 | 21.26 | 36.87 | 54 | 15646.76 | 1789.85 | 0.11 | NA | 87.35 |
| RP137 | I KUNG PAO::IRGC 114-1 | LL_N | WS | 91 | 99.36 | 19.11 | 30.61 | 47 | 14307.95 | 1291.96 | 0.16 | 200.00 | 86.03 |
| RP138 | IA CUBA 17::IRGC 116990-1 | LL_N | WS | 93 | 84.80 | 18.82 | 29.73 | 70 | 16485.84 | 1147.44 | 0.04 | 250.00 | 89.09 |
| RP139 | ICTA CRISPO 38::IRGC 116994-1 | LL_N | WS | 91 | 72.67 | 18.53 | 26.13 | 49 | 11548.54 | 1095.03 | 0.26 | 200.00 | 85.95 |
| RP140 | ICTA MOTAGUA::IRGC 116995-1 | LL_N | WS | 94 | 88.45 | 17.10 | 32.29 | 36 | 14823.76 | 552.67 | 0.02 | 180.00 | 82.46 |
| RP141 | INIAP 6::IRGC 117002-1 | LL_N | WS | 94 | 91.39 | 20.40 | 38.53 | 46 | 15918.65 | 1202.23 | 0.10 | 230.00 | 89.42 |
| RP142 | IR 13429-109-2-2-1::IRGC 63491-1 | LL_N | WS | 92 | 87.49 | 19.97 | 20.76 | 35 | 11741.10 | 1448.40 | 0.28 | NA | 88.09 |
| RP143 | IR 21015-72-3-3-3-1::IRGC 117004-1 | LL_N | WS | 91 | 83.60 | 22.12 | 37.60 | 24 | 22868.53 | 759.92 | 0.01 | 190.00 | 86.79 |
| **Study designation** | **3KG database designation** | **Env** | **Season** | **DTF** | **PH (cm)** | **PL (cm)** | **FlgLA (cm^2^)** | **NBP** | **BMDW (kgha^-1^)** | **GY (kgha^-1^)** | **HI** | **TGW (g)** | **SPKFT (%)** |
| RP144 | IR 2344-P1 PB-9-3-2B::IRGC 39317-C1 | LL_N | WS | 86 | 127.23 | 24.55 | 26.51 | 41 | 12232.80 | 1157.76 | 0.50 | 190.00 | 89.48 |
| RP146 | IR 63295-AC 209-7::IRGC 117365-1 | LL_N | WS | 92 | 99.35 | 20.79 | 32.28 | 55 | 21705.50 | 697.99 | 0.05 | NA | 89.28 |
| RP147 | IR 69502-6-SRN-3-UBN-1-B::IRGC 117290-1 | LL_N | WS | 91 | 95.92 | 20.68 | 38.14 | 42 | 12429.20 | 1397.57 | 0.27 | 210.00 | 87.38 |
| RP148 | IR 75870-5-8-5-B-1::IRGC 117297-1 | LL_N | WS | 92 | 104.04 | 24.55 | 34.13 | 48 | 13669.82 | 1671.53 | 0.31 | 180.00 | 87.63 |
| RP149 | IR 77390-1-6-4-19-1-B::IRGC 117303-1 | LL_N | WS | 97 | 92.81 | 16.47 | 26.48 | 38 | 14838.79 | 1044.21 | 0.13 | 360.00 | 85.71 |
| RP150 | IR 80310-12-B-1-3-B::IRGC 117307-1 | LL_N | WS | 91 | 120.90 | 21.83 | 48.11 | 38 | 11154.69 | 1427.75 | 0.65 | 220.00 | 87.07 |
| RP151 | IR 80340-23-B-12-6-B::IRGC 117309-1 | LL_N | WS | 91 | 107.48 | 19.97 | 29.51 | 51 | 14482.33 | 1006.88 | 0.12 | 230.00 | 89.73 |
| RP152 | IRGA 318-11-6-9-2B::IRGC 117339-1 | LL_N | WS | 90 | 74.08 | 19.74 | 30.16 | 58 | 13981.54 | 1219.70 | 0.12 | 210.00 | 87.67 |
| RP153 | IRGA 370-38-1-1F-C4-2::IRGC 117342-1 | LL_N | WS | 93 | 91.55 | 19.25 | 34.66 | 48 | 15402.00 | 1543.69 | 0.05 | 240.00 | 87.42 |
| RP154 | IRGA 370-42-1-1F-C-1::IRGC 117343-1 | LL_N | WS | 89 | 81.25 | 20.97 | 28.62 | 47 | 15402.00 | 1584.18 | 0.05 | 230.00 | 87.61 |
| RP155 | IRGA 659-1-2-2-2::IRGC 117345-1 | LL_N | WS | 93 | 101.24 | 20.83 | 41.23 | 39 | 15204.33 | 1339.61 | 0.14 | 240.00 | 89.88 |
| RP156 | IRGA 959-1-2-2F-4-1-4A-6-CA-6X::IRGC 117006-1 | LL_N | WS | 87 | 83.30 | 19.11 | 28.89 | 44 | 11872.50 | 818.69 | 0.28 | 220.00 | 85.57 |
| RP157 | IRI 339::IRGC 46956-1 | LL_N | WS | 91 | 81.57 | 19.25 | 23.95 | 42 | 12144.04 | 784.54 | 0.17 | 260.00 | 87.51 |
| RP158 | IRRIBINI::IRGC 49094-1 | LL_N | WS | 91 | 129.93 | 27.90 | 52.17 | 41 | 19994.15 | 1960.58 | 0.14 | 260.00 | 87.35 |
| RP159 | JABOR SAIL::IRGC 66831-1 | LL_N | WS | 90 | 114.03 | 19.68 | 54.87 | 39 | 12844.02 | 1615.95 | 0.16 | 260.00 | 85.63 |
| RP160 | JAGLI BORO::IRGC 27516-2 | LL_N | WS | 88 | 125.35 | 18.82 | 25.21 | 80 | 14390.42 | 3936.26 | 0.34 | 220.00 | 88.98 |
| RP161 | JAMBALI::IRGC 73101-1 | LL_N | WS | 89 | 109.02 | 17.25 | 29.29 | 28 | 16882.13 | 1257.02 | 0.07 | 220.00 | 88.79 |
| RP162 | JAO LEUANG::IRGC 65866-1 | LL_N | WS | 89 | 133.47 | 25.32 | 33.25 | 27 | 17125.02 | 569.34 | 0.01 | 240.00 | 77.19 |
| RP164 | JHODI BIRUN::IRGC 31812-1 | LL_N | WS | 89 | 113.70 | 28.13 | 38.27 | 67 | 21652.03 | 1515.89 | 0.08 | 220.00 | 85.50 |
| RP165 | JIN JUN DAO::IRGC 59710-1 | LL_N | WS | 88 | 109.96 | 22.83 | 30.81 | 35 | 13172.52 | 875.86 | 0.07 | 200.00 | 87.55 |
| RP166 | JUMA 62::IRGC 117011-1 | LL_N | WS | 93 | 92.59 | 22.83 | 41.35 | 48 | 13735.17 | 710.69 | 0.10 | 240.00 | 84.79 |
| RP167 | KALABAIL::IRGC 25877-1 | LL_N | WS | 91 | 130.57 | 23.41 | 48.85 | 39 | 11737.26 | 2477.53 | 0.36 | 210.00 | 83.65 |
| RP168 | KALIA::IRGC 34699-1 | LL_N | WS | 90 | 112.14 | 16.39 | 30.61 | 49 | 13760.33 | 1015.62 | 0.08 | NA | 88.77 |
| RP169 | KALO CHAKOL::IRGC 77258-1 | LL_N | WS | 98 | 87.49 | 23.26 | 31.33 | 61 | 14090.93 | 2918.25 | 0.21 | 290.00 | 85.21 |
| RP170 | KALU ILANKALAYAN::IRGC 36270-1 | LL_N | WS | 94 | 99.99 | 16.30 | 31.69 | 48 | 18400.58 | 1883.55 | 0.06 | 260.00 | 89.27 |
| RP171 | KAM PAI::IRGC 78245-1 | LL_N | WS | 89 | 137.84 | 27.13 | 56.15 | 23 | 12970.88 | 1261.79 | 0.08 | 230.00 | 77.83 |
| RP172 | KEERIPALA CHILL PADDY::IRGC 49790-1 | LL_N | WS | 88 | 127.13 | 22.00 | 34.76 | 55 | 13477.96 | 1829.56 | 0.37 | 230.00 | 90.82 |
| **Study designation** | **3KG database designation** | **Env** | **Season** | **DTF** | **PH (cm)** | **PL (cm)** | **FlgLA (cm^2^)** | **NBP** | **BMDW (kgha^-1^)** | **GY (kgha^-1^)** | **HI** | **TGW (g)** | **SPKFT (%)** |
| RP173 | KETAN SERANG::IRGC 14615-1 | LL_N | WS | 98 | 154.58 | 26.98 | 47.08 | 58 | 17276.68 | 1702.50 | 0.12 | 210.00 | 85.52 |
| RP174 | KHAO DAWK MALI 105::IRGC 27748-2 | LL_N | WS | 91 | 136.02 | 23.97 | 46.46 | 48 | 17977.72 | 993.38 | 0.02 | 320.00 | 86.85 |
| RP175 | KHAO GRADOOK CHAHNG::IRGC 17111-1 | LL_N | WS | 91 | 83.44 | 19.54 | 22.91 | 55 | 15068.39 | 1900.23 | 0.09 | 250.00 | 89.08 |
| RP176 | KHAO' HAWM::IRGC 78257-1 | LL_N | WS | 98 | 87.49 | 23.26 | 31.33 | 27 | 13937.51 | 1692.86 | 0.00 | NA | 86.85 |
| RP177 | KHAO THI RATE::IRGC 58041-1 | LL_N | WS | 86 | 96.03 | 17.96 | 27.89 | 32 | 11954.28 | 751.98 | 0.12 | 200.00 | 78.65 |
| RP178 | KHARSU 80::IRGC 28016-1 | LL_N | WS | 87 | 98.42 | 18.60 | 26.79 | 46 | 11655.48 | 1272.11 | 0.19 | 230.00 | 84.95 |
| RP179 | KITRANA 1007::IRGC 68517-1 | LL_N | WS | 93 | 136.33 | 22.77 | 34.05 | 68 | 22562.05 | 1851.00 | 0.09 | 290.00 | 87.43 |
| RP180 | KN 1 B 361-1-8-6-9::IRGC 46974-1 | LL_N | WS | 89 | 134.41 | 24.27 | 51.27 | 44 | 14926.86 | 2751.49 | 0.38 | 280.00 | 89.15 |
| RP181 | KORASISI::IRGC 5285-1 | LL_N | WS | 89 | 151.92 | 24.55 | 40.16 | 54 | 22820.66 | 957.65 | 0.06 | 260.00 | 86.37 |
| RP182 | KOTTEYARAN::IRGC 47383-1 | LL_N | WS | 89 | 128.05 | 23.46 | 37.42 | 47 | 19799.15 | 1834.32 | 0.02 | 260.00 | 85.71 |
| RP183 | KOYRA::IRGC 77267-1 | LL_N | WS | 89 | 109.44 | 20.68 | 41.26 | 56 | 13589.44 | 3001.63 | 0.40 | 200.00 | 89.27 |
| RP184 | KULA KARUPPAN::IRGC 55328-1 | LL_N | WS | 91 | 141.80 | 23.46 | 44.58 | 44 | 20386.96 | 4253.10 | 0.14 | 240.00 | 89.86 |
| RP185 | KUNENG::IRGC 71545-1 | LL_N | WS | 92 | 81.57 | 17.96 | 37.76 | NA | 15402.00 | 1692.86 | 0.00 | NA | 86.85 |
| RP186 | KURULU WEE (WHITE)::IRGC 66518-1 | LL_N | WS | 88 | 107.80 | 21.54 | 33.18 | 47 | 16677.70 | 1051.35 | 0.09 | 90.00 | 88.08 |
| RP188 | KUSHIARA::IRGC 34709-1 | LL_N | WS | 91 | 126.82 | 25.26 | 43.77 | 42 | 16618.29 | 2028.87 | 0.08 | 220.00 | 86.40 |
| RP189 | KUTTA::IRGC 52184-1 | LL_N | WS | 92 | 150.99 | 23.97 | 37.71 | 46 | 12106.99 | 1519.86 | 0.42 | 210.00 | 87.09 |
| RP190 | LABRA::IRGC 74757-1 | LL_N | WS | 90 | 129.31 | 22.86 | 47.86 | 46 | 22868.18 | 940.98 | 0.03 | 230.00 | 86.59 |
| RP191 | LAI YIP ZIM::IRGC 4955-1 | LL_N | WS | 91 | 124.63 | 17.10 | 42.22 | 51 | 16182.85 | 2103.52 | 0.13 | NA | 87.13 |
| RP192 | LALKA (LAL DHAN)::IRGC 64946-1 | LL_N | WS | 90 | 138.99 | 26.33 | 48.53 | 37 | 14378.89 | 2871.40 | 0.19 | 290.00 | 90.66 |
| RP193 | LALSAITA::IRGC 43915-1 | LL_N | WS | 88 | 109.35 | 18.45 | 39.47 | 51 | 11757.52 | 3465.37 | 0.00 | 250.00 | 89.33 |
| RP194 | LARHA MUGAD::IRGC 52339-1 | LL_N | WS | 91 | 127.76 | 21.40 | 32.50 | 42 | 14072.41 | 2367.95 | 0.29 | 210.00 | 90.05 |
| RP196 | LENJA MURALI::IRGC 66815-1 | LL_N | WS | 90 | 127.44 | 27.84 | 54.23 | 37 | 16829.36 | 880.62 | 0.06 | 230.00 | 86.22 |
| RP197 | LEUANG YAI 29-12-2::IRGC 881-1 | LL_N | WS | 92 | 128.07 | 22.26 | 39.44 | 24 | 18797.22 | 711.48 | 0.00 | 240.00 | 86.85 |
| RP198 | LU MAO ZHAN::IRGC 68159-1 | LL_N | WS | 86 | 118.18 | 19.16 | 24.36 | 46 | 11466.42 | 1569.89 | 0.57 | 230.00 | 87.94 |
| RP199 | LUA CHAN HUONG::IRGC 16800-1 | LL_N | WS | 88 | 78.92 | 18.42 | 21.11 | NA | 15402.00 | 1692.86 | 0.00 | NA | 86.85 |
| RP200 | MAKALIOKA::IRGC 376-1 | LL_N | WS | 86 | 108.51 | 21.54 | 34.51 | 58 | 13509.76 | 2768.16 | 0.30 | 230.00 | 87.36 |
| RP201 | MAKRO::IRGC 74763-1 | LL_N | WS | 90 | 129.31 | 22.98 | 43.93 | 45 | 16385.19 | 2639.52 | 0.19 | 260.00 | 88.31 |
| RP202 | MAMORIAKA::IRGC 68672-1 | LL_N | WS | 88 | 120.10 | 20.54 | 49.52 | 54 | 16484.79 | 1125.20 | 0.06 | 200.00 | 89.06 |
| **Study designation** | **3KG database designation** | **Env** | **Season** | **DTF** | **PH (cm)** | **PL (cm)** | **FlgLA (cm^2^)** | **NBP** | **BMDW (kgha^-1^)** | **GY (kgha^-1^)** | **HI** | **TGW (g)** | **SPKFT (%)** |
| RP203 | MEI FENG 9::IRGC 63735-1 | LL_N | WS | 86 | 77.62 | 19.54 | 33.21 | 41 | 11692.52 | 2431.47 | 0.00 | 260.00 | 88.76 |
| RP204 | MEKENZIE SMALL::IRGC 49895-1 | LL_N | WS | 93 | 91.66 | 21.26 | 31.17 | 55 | 11123.94 | 4053.79 | 0.00 | 210.00 | 81.35 |
| RP205 | MELEKE::IRGC 56823-1 | LL_N | WS | 88 | 120.58 | 24.70 | 44.14 | 39 | 15711.76 | 2859.48 | 0.12 | 240.00 | 80.80 |
| RP206 | MENTIK TJERE BELUT::IRGC 18254-1 | LL_N | WS | 91 | 151.00 | 24.55 | 51.83 | 34 | 13735.17 | 1338.81 | 0.12 | 170.00 | 82.04 |
| RP207 | MILYANG 30::IRGC 46977-1 | LL_N | WS | 91 | 61.61 | 16.02 | 24.01 | 44 | 10639.57 | 1692.86 | 0.28 | 150.00 | 82.20 |
| RP208 | MILYANG 77::IRGC 69340-1 | LL_N | WS | 87 | 79.54 | 18.25 | 24.64 | 51 | 12409.98 | 1443.63 | 0.34 | 170.00 | 85.25 |
| RP209 | MIN KE ZHAN::IRGC 72230-1 | LL_N | WS | 89 | 88.76 | 21.97 | 32.90 | 38 | 16003.22 | 695.60 | 0.06 | 150.00 | 88.05 |
| RP210 | MIN ZAO 6::IRGC 63772-1 | LL_N | WS | 89 | 78.13 | 19.45 | 25.72 | 33 | 18863.97 | 1160.14 | 0.06 | 220.00 | 87.36 |
| RP211 | MODDAI KARUPPAN::IRGC 15465-1 | LL_N | WS | 89 | 143.97 | 25.18 | 38.61 | 56 | 22846.17 | 1677.09 | 0.02 | 290.00 | 83.86 |
| RP212 | MUKKALA BAZAL::IRGC 77279-1 | LL_N | WS | 92 | 120.27 | 20.88 | 31.14 | 55 | 11580.34 | 2059.05 | 0.64 | 250.00 | 88.32 |
| RP213 | MULLIKURUVA::IRGC 77529-1 | LL_N | WS | 91 | 152.24 | 25.13 | 35.73 | NA | 14003.91 | 1692.86 | 0.00 | NA | 86.85 |
| RP214 | MUTA GANJE::IRGC 26744-1 | LL_N | WS | 88 | 117.56 | 25.18 | 30.93 | 51 | 16506.11 | 1520.66 | 0.09 | 200.00 | 84.46 |
| RP215 | MUTTU SAMBA::IRGC 36333-1 | LL_N | WS | 94 | 143.46 | 23.26 | 43.25 | 51 | 17502.79 | 900.48 | 0.05 | 120.00 | 83.37 |
| RP216 | N 22::IRGC 46459-1 | LL_N | WS | 90 | 117.46 | 18.96 | 28.74 | 53 | 13246.26 | 2141.63 | 0.35 | 210.00 | 88.78 |
| RP217 | Na souan::IRGC 11889-1 | LL_N | WS | 87 | 119.64 | 24.55 | 44.39 | 27 | 14110.85 | 1692.86 | 0.00 | 310.00 | 83.80 |
| RP218 | NAZIRA SAIL::IRGC 77284-1 | LL_N | WS | 91 | 121.35 | 25.47 | 45.49 | 53 | 18311.81 | 1831.94 | 0.04 | 160.00 | 75.43 |
| RP219 | NCS 194::IRGC 51932-1 | LL_N | WS | 91 | 134.92 | 22.49 | 49.01 | 45 | 23888.28 | 1896.26 | 0.07 | 230.00 | 89.13 |
| RP220 | NCS 237::IRGC 62202-1 | LL_N | WS | 90 | 124.74 | 20.37 | 38.18 | 58 | 14614.08 | 2266.30 | 0.17 | 210.00 | 90.41 |
| RP222 | NCS 964 C::IRGC 62604-1 | LL_N | WS | 98 | 94.98 | 16.53 | 31.25 | 25 | 16030.13 | 1692.86 | 0.00 | 180.00 | 82.55 |
| RP223 | NIAO YAO::IRGC 5496-1 | LL_N | WS | 93 | 146.17 | 23.26 | 36.15 | 37 | 16747.59 | 1463.48 | 0.12 | 190.00 | 87.96 |
| RP224 | NIBARI::IRGC 67742-1 | LL_N | WS | 89 | 117.46 | 21.69 | 28.83 | 37 | 12112.24 | 2155.93 | 0.27 | 220.00 | 86.89 |
| RP225 | NONA BOKRA::IRGC 22710-C1 | LL_N | WS | 86 | 113.55 | 28.44 | 38.66 | 58 | 17181.28 | 1332.46 | 0.10 | 260.00 | 89.36 |
| RP226 | NOROI::IRGC 31611-1 | LL_N | WS | 86 | 105.91 | 16.79 | 30.96 | 60 | 12782.87 | 2461.65 | 0.39 | 260.00 | 89.00 |
| RP228 | NX 3533::IRGC 63796-1 | LL_N | WS | 94 | 64.72 | 17.39 | 28.54 | 44 | 11162.38 | 752.78 | 0.28 | 220.00 | 85.34 |
| RP229 | O. SATIVA::IRGC 17083-1 | LL_N | WS | 93 | 148.66 | 29.28 | 41.65 | 44 | 13609.01 | 3044.51 | 0.31 | 210.00 | 86.65 |
| RP230 | PAH WEAN::IRGC 78276-1 | LL_N | WS | 91 | 91.66 | 16.47 | 26.48 | 36 | 19397.96 | 566.96 | 0.02 | 240.00 | 76.22 |
| RP231 | PAI CHUEH CHIU LIU::IRGC 34259-1 | LL_N | WS | 91 | 92.28 | 20.25 | 32.76 | 37 | 22243.33 | 1817.65 | 0.05 | 190.00 | 88.92 |
| RP232 | PAI YI PING::IRGC 1368-1 | LL_N | WS | 87 | 101.38 | 21.69 | 26.57 | 35 | 16983.48 | 1944.70 | 0.07 | 230.00 | 82.91 |
| **Study designation** | **3KG database designation** | **Env** | **Season** | **DTF** | **PH (cm)** | **PL (cm)** | **FlgLA (cm^2^)** | **NBP** | **BMDW (kgha^-1^)** | **GY (kgha^-1^)** | **HI** | **TGW (g)** | **SPKFT (%)** |
| RP233 | PALEPYU::IRGC 33549-1 | LL_N | WS | 86 | 118.08 | 23.11 | 31.13 | 58 | 14357.92 | 3721.86 | 0.37 | 230.00 | 88.88 |
| RP234 | PARA NELLU::IRGC 50009-1 | LL_N | WS | 87 | 144.29 | 24.83 | 30.40 | 53 | 14570.05 | 3719.48 | 0.40 | 290.00 | 89.10 |
| RP235 | PATISAIL::IRGC 37562-1 | LL_N | WS | 99 | 79.54 | 23.69 | 38.99 | 72 | 21103.72 | 3201.74 | 0.13 | 140.00 | 88.19 |
| RP237 | PERUNEL::IRGC 63113-1 | LL_N | WS | 89 | 147.82 | 25.13 | 33.18 | 37 | 11562.52 | 2116.22 | 0.68 | 240.00 | 90.71 |
| RP238 | PICO NEGRO::IRGC 55849-1 | LL_N | WS | 98 | 71.58 | 17.91 | 28.69 | 25 | 15912.36 | 2144.01 | 0.03 | 270.00 | 79.19 |
| RP239 | PLI KHAO::IRGC 64596-1 | LL_N | WS | 93 | 146.88 | 25.26 | 55.65 | 39 | 13373.47 | 1713.62 | 0.09 | 260.00 | 82.86 |
| RP240 | PODI HEENATI::IRGC 36345-1 | LL_N | WS | 89 | 116.20 | 19.80 | 30.27 | 40 | 18890.18 | 1341.19 | 0.07 | 150.00 | 88.59 |
| RP241 | PSBRC 50::IRGC 99706-1 | LL_N | WS | 93 | 106.86 | 20.40 | 29.05 | 48 | 13636.62 | 1365.02 | 0.24 | 260.00 | 88.97 |
| RP242 | PSBRC 68::IRGC 99711-1 | LL_N | WS | 91 | 112.88 | 25.26 | 50.95 | 44 | 15387.46 | 1896.26 | 0.19 | 250.00 | 86.38 |
| RP243 | PSBRC 88::IRGC 99717-1 | LL_N | WS | 92 | 113.23 | 23.69 | 40.51 | 60 | 17724.00 | 2225.01 | 0.12 | 210.00 | 87.03 |
| RP244 | PULUT BARAYA::IRGC 27393-1 | LL_N | WS | 92 | 108.41 | 24.55 | 42.87 | 35 | 13404.22 | 1692.86 | 0.00 | NA | 86.85 |
| RP245 | PURA BINNI::IRGC 26772-1 | LL_N | WS | 94 | 143.51 | 24.12 | 37.94 | 49 | 15432.89 | 1686.62 | 0.17 | 200.00 | 88.72 |
| RP247 | QUERO ASSAN::IRGC 28860-1 | LL_N | WS | 88 | 124.42 | 23.66 | 51.83 | 70 | 18725.93 | 1264.17 | 0.05 | 250.00 | 79.97 |
| RP248 | RACE PERUMAL::IRGC 55347-1 | LL_N | WS | 87 | 127.23 | 21.83 | 24.18 | 43 | 15402.00 | 1249.87 | 0.02 | NA | 88.11 |
| RP250 | RELLY::IRGC 14623-1 | LL_N | WS | 99 | 67.55 | 19.68 | 23.87 | 39 | 15369.28 | 719.43 | 0.07 | 170.00 | 75.53 |
| RP251 | RIZ TYPE SORGHO::IRGC 69015-1 | LL_N | WS | 86 | 83.14 | 18.82 | 25.02 | 34 | 11861.67 | 1692.86 | 0.00 | 120.00 | 78.02 |
| RP252 | ROJOFOTSY::IRGC 69402-1 | LL_N | WS | 93 | 133.16 | 21.40 | 52.35 | 32 | 18179.36 | 1692.86 | 0.00 | NA | 86.85 |
| RP253 | RPA 5929 (K 45)::IRGC 33963-1 | LL_N | WS | 93 | 88.54 | 20.97 | 49.39 | 32 | 14920.92 | 836.95 | 0.07 | 220.00 | 85.40 |
| RP254 | RPW 9-4 (SS 1)::IRGC 50690-1 | LL_N | WS | 92 | 94.98 | 20.83 | 29.13 | 60 | 11383.59 | 937.80 | 0.54 | 200.00 | 89.99 |
| RP255 | RR 272-17-829::IRGC 117354-1 | LL_N | WS | 90 | 104.36 | 22.77 | 34.97 | 48 | 11944.49 | 2965.10 | 0.00 | 180.00 | 89.35 |
| RP256 | RTS 16::IRGC 8235-1 | LL_N | WS | 88 | 107.16 | 23.84 | 38.46 | 62 | 11708.95 | 1962.17 | 0.51 | 180.00 | 87.98 |
| RP257 | RUSTIC::IRGC 117026-1 | LL_N | WS | 94 | 79.71 | 19.97 | 21.41 | 44 | 13803.66 | 697.99 | 0.09 | 160.00 | 87.12 |
| RP258 | SADA RUPA::IRGC 77299-1 | LL_N | WS | 94 | 110.42 | 21.83 | 37.89 | 49 | 18543.16 | 1762.85 | 0.12 | 250.00 | 89.27 |
| RP259 | SAN CHIAO TSWEN::IRGC 1565-1 | LL_N | WS | 91 | 143.98 | 24.70 | 37.07 | 62 | 15402.00 | 2838.04 | 0.05 | 230.00 | 88.59 |
| RP260 | SAN SHIH TSI::IRGC 1038-1 | LL_N | WS | 92 | 127.13 | 19.82 | 42.45 | 39 | 14083.24 | 1094.23 | 0.09 | 200.00 | 90.17 |
| RP261 | SIGARDIS::IRGC 15555-1 | LL_N | WS | 91 | 72.84 | 17.91 | 28.69 | 30 | 21075.06 | 2036.02 | 0.06 | 250.00 | 87.58 |
| RP262 | SIMET 2::IRGC 25734-1 | LL_N | WS | 94 | 87.65 | 23.26 | 31.33 | 37 | 15402.00 | 1692.86 | 0.00 | NA | 86.85 |
| RP263 | SIMUL KHURI::IRGC 35154-1 | LL_N | WS | 91 | 119.95 | 17.19 | 40.64 | 46 | 15043.93 | 1173.64 | 0.12 | 230.00 | 90.00 |
| **Study designation** | **3KG database designation** | **Env** | **Season** | **DTF** | **PH (cm)** | **PL (cm)** | **FlgLA (cm^2^)** | **NBP** | **BMDW (kgha^-1^)** | **GY (kgha^-1^)** | **HI** | **TGW (g)** | **SPKFT (%)** |
| RP264 | SINNA SITHIRA KALI::IRGC 51064-1 | LL_N | WS | 94 | 112.14 | 20.06 | 23.97 | 34 | 13094.24 | 1372.96 | 0.21 | 180.00 | 86.56 |
| RP266 | SITHAIYAN KOTTAI SAMBA::IRGC 50155-1 | LL_N | WS | 90 | 132.12 | 28.01 | 45.73 | 46 | 20079.07 | 2491.82 | 0.07 | 160.00 | 86.87 |
| RP267 | SML AWINI::IRGC 13391-1 | LL_N | WS | 92 | 89.99 | 18.53 | 25.81 | 48 | 16035.37 | 670.99 | 0.07 | 240.00 | 87.49 |
| RP268 | SOLOMON RED RICE::IRGC 50950-1 | LL_N | WS | 91 | 130.86 | 23.32 | 46.67 | 72 | 13854.34 | 2707.81 | 0.59 | 200.00 | 89.38 |
| RP269 | SONA::IRGC 26971-C1 | LL_N | WS | 93 | 82.61 | 21.54 | 30.31 | 37 | 14588.22 | 689.25 | 0.06 | 140.00 | 87.11 |
| RP270 | SONAMUKHI::IRGC 46693-1 | LL_N | WS | 88 | 114.03 | 15.10 | 25.17 | 35 | 11540.15 | 1272.11 | 0.21 | 190.00 | 88.68 |
| RP272 | SSANGDUJO::IRGC 55632-1 | LL_N | WS | 91 | 137.89 | 22.98 | 47.83 | 48 | 18228.99 | 2844.40 | 0.16 | 240.00 | 88.33 |
| RP273 | SUFAID 246::IRGC 28303-1 | LL_N | WS | 90 | 136.19 | 23.97 | 41.44 | 48 | 15513.96 | 1199.85 | 0.09 | 170.00 | 86.68 |
| RP274 | SUWEON 311::IRGC 61890-1 | LL_N | WS | 88 | 78.29 | 17.96 | 27.72 | 32 | 14629.81 | 694.81 | 0.06 | 210.00 | 85.25 |
| RP275 | T 315::IRGC 54792-1 | LL_N | WS | 90 | 141.17 | 24.70 | 45.76 | 42 | 11565.32 | 1856.56 | 0.69 | 270.00 | 90.02 |
| RP276 | TAIPEI WOO CO::IRGC 112-1 | LL_N | WS | 92 | 136.91 | 23.11 | 49.11 | 47 | 12624.21 | 1489.69 | 0.36 | 190.00 | 89.97 |
| RP277 | TAITUNG WOO LI::IRGC 111-1 | LL_N | WS | 94 | 143.19 | 20.45 | 38.48 | 46 | 18764.02 | 1045.79 | 0.03 | 200.00 | 85.87 |
| RP278 | TAK SUFAID::IRGC 73127-1 | LL_N | WS | 90 | 120.99 | 18.96 | 37.50 | 23 | 13417.50 | 618.58 | 0.03 | NA | 88.45 |
| RP279 | TAK::IRGC 73124-1 | LL_N | WS | 92 | 112.93 | 20.83 | 35.32 | 56 | 12714.72 | 937.80 | 0.12 | 130.00 | 79.73 |
| RP280 | TIKAL 3::IRGC 50649-1 | LL_N | WS | 93 | 104.04 | 22.12 | 35.17 | 51 | 18778.35 | 2864.25 | 0.14 | 310.00 | 87.87 |
| RP281 | TOC 5430::IRGC 70487-1 | LL_N | WS | 91 | 86.41 | 23.18 | 30.94 | 45 | 14555.72 | 1129.17 | 0.10 | 220.00 | 87.28 |
| RP282 | TONG GU HONG::IRGC 81026-1 | LL_N | WS | 87 | 119.43 | 22.69 | 29.68 | 49 | 20371.23 | 1309.43 | 0.05 | 230.00 | 85.56 |
| RP283 | TSAI YUAN CHON::IRGC 126-1 | LL_N | WS | 90 | 130.26 | 26.55 | 45.38 | 49 | 14733.25 | 2887.28 | 0.21 | 210.00 | 89.70 |
| RP284 | TSAO SHENG LI 1::IRGC 1309-1 | LL_N | WS | 93 | 123.06 | 22.69 | 47.83 | 35 | 13401.08 | 1255.43 | 0.19 | 250.00 | 89.84 |
| RP285 | UPRH 233::IRGC 61667-1 | LL_N | WS | 86 | 68.48 | 15.67 | 25.34 | NA | 15402.00 | 1692.86 | 0.00 | NA | 86.85 |
| RP286 | UQUIHUA::IRGC 117037-1 | LL_N | WS | 94 | 75.97 | 16.33 | 18.96 | 69 | 16388.33 | 1064.06 | 0.06 | 240.00 | 85.80 |
| RP287 | URAIBOOL::IRGC 52785-1 | LL_N | WS | 92 | 91.55 | 16.47 | 26.48 | 41 | 15030.65 | 1075.97 | 0.14 | 240.00 | 85.79 |
| RP288 | VEN THAP::IRGC 56138-1 | LL_N | WS | 91 | 139.09 | 22.55 | 31.62 | 57 | 17088.67 | 1276.87 | 0.08 | 180.00 | 88.40 |
| RP289 | WANGA BARUGULU::IRGC 52261-1 | LL_N | WS | 90 | 117.60 | 22.98 | 33.14 | 50 | 15493.00 | 1506.36 | 0.13 | 190.00 | 86.69 |
| RP290 | WAR 72-2-1-1::IRGC 117361-1 | LL_N | WS | 87 | 115.27 | 23.84 | 35.18 | 61 | 15837.22 | 1469.84 | 0.01 | 250.00 | 88.75 |
| RP291 | WI BIR SHUN::IRGC 4602-1 | LL_N | WS | 91 | 135.03 | 22.12 | 44.98 | 70 | 17020.17 | 1838.29 | 0.14 | 200.00 | 85.98 |
| RP292 | WP 36::IRGC 55278-1 | LL_N | WS | 99 | 91.70 | 16.96 | 21.75 | 43 | 20805.97 | 1563.54 | 0.02 | 210.00 | 86.85 |
| RP293 | WP 65::IRGC 36526-1 | LL_N | WS | 94 | 94.35 | 22.98 | 35.58 | 44 | 12228.61 | 1108.53 | 0.25 | 280.00 | 86.52 |
| **Study designation** | **3KG database designation** | **Env** | **Season** | **DTF** | **PH (cm)** | **PL (cm)** | **FlgLA (cm^2^)** | **NBP** | **BMDW (kgha^-1^)** | **GY (kgha^-1^)** | **HI** | **TGW (g)** | **SPKFT (%)** |
| RP294 | XI GAN JING REN::IRGC 60035-1 | LL_N | WS | 86 | 102.27 | 21.56 | 25.40 | 36 | 12023.82 | 1307.05 | 0.26 | 200.00 | 90.66 |
| RP295 | XIA ZHI BAI::IRGC 53437-1 | LL_N | WS | 84 | 93.74 | 17.53 | 23.26 | 46 | 11377.65 | 1063.26 | 0.18 | 150.00 | 82.78 |
| RP296 | XITTO::IRGC 6671-1 | LL_N | WS | 89 | 137.73 | 25.18 | 34.36 | 42 | 12478.48 | 3709.16 | 0.70 | 330.00 | 87.87 |
| RP297 | YA NONG ZAO 4::IRGC 63908-1 | LL_N | WS | 92 | 84.70 | 20.34 | 29.19 | 42 | 11513.25 | 832.19 | 0.19 | 210.00 | 81.85 |
| RP298 | YEBAWYIN::IRGC 33885-1 | LL_N | WS | 90 | 138.20 | 27.33 | 46.37 | 46 | 18934.56 | 4747.82 | 0.23 | 290.00 | 88.83 |
| RP299 | YONG JIN ZAO 3::IRGC 70441-1 | LL_N | WS | 86 | 81.05 | 21.26 | 29.13 | 31 | 20656.40 | 1249.87 | 0.04 | NA | 88.00 |
| RP300 | H 6::IRGC 157-1 | LL_N | WS | 91 | 140.22 | 24.40 | 37.95 | 61 | 13385.00 | 2680.82 | 0.29 | 250.00 | 87.43 |
| RP002 | 19::IRGC 70786-1 | LL_N | DS | 93 | 144.81 | 21.62 | 40.90 | 64 | 22828.81 | 7364.87 | 0.40 | 184.31 | 97.68 |
| RP003 | 3210::IRGC 116950-1 | LL_N | DS | 102 | 79.52 | 18.24 | 38.11 | 58 | 11327.58 | 3433.00 | 0.33 | 193.25 | 98.49 |
| RP004 | 498-2A BR 8::IRGC 5891-1 | LL_N | DS | 90 | 125.66 | 23.52 | 33.73 | 50 | 13244.79 | 6331.54 | 0.41 | 273.69 | 97.45 |
| RP005 | ADT 12::IRGC 6254-1 | LL_N | DS | 85 | 135.92 | 27.31 | 47.44 | 57 | 16048.01 | 7463.47 | 0.45 | 237.94 | 97.65 |
| RP006 | AE NOUA::IRGC 89308-1 | LL_N | DS | 86 | 131.75 | 22.70 | 41.61 | 43 | 12658.79 | 6510.04 | 0.42 | 291.56 | 97.27 |
| RP007 | ALTAMIRA 9::IRGC 116953-1 | LL_N | DS | NA | 91.07 | 16.83 | 24.89 | 35 | 8302.70 | 2173.89 | 0.21 | 229.00 | 94.61 |
| RP008 | ARAURE 1::IRGC 116956-1 | LL_N | DS | 93 | 94.73 | 21.76 | 27.31 | 58 | 12793.20 | 4203.27 | 0.35 | 246.87 | 96.57 |
| RP009 | ARC 10100::IRGC 20709-1 | LL_N | DS | 89 | 123.61 | 22.30 | 48.91 | 47 | 14865.02 | 4565.43 | 0.39 | 175.37 | 96.74 |
| RP010 | ARC 10594::IRGC 12524-1 | LL_N | DS | 84 | 150.56 | 25.42 | 35.76 | 57 | 20621.69 | 6102.30 | 0.39 | 229.00 | 96.64 |
| RP011 | ARC 10754::IRGC 12603-1 | LL_N | DS | 91 | 121.68 | 23.65 | 32.36 | 52 | 14973.66 | 6427.47 | 0.41 | 202.19 | 96.46 |
| RP012 | ARC 10812::IRGC 21074-1 | LL_N | DS | 76 | 118.79 | 23.17 | 38.62 | 52 | 8158.46 | 3912.97 | 0.38 | 237.94 | 96.22 |
| RP013 | ARC 11524::IRGC 42672-1 | LL_N | DS | 79 | 121.68 | 22.98 | 47.30 | 49 | 11444.69 | 5156.70 | 0.40 | 237.94 | 96.34 |
| RP014 | ARC 11857::IRGC 40972-1 | LL_N | DS | 79 | 138.96 | 23.17 | 29.56 | 57 | 16710.09 | 5651.92 | 0.37 | 220.06 | 96.75 |
| RP015 | ARC 11901::IRGC 21727-1 | LL_N | DS | 80 | 138.87 | 27.85 | 37.76 | 67 | 16994.66 | 6721.90 | 0.36 | 237.94 | 98.54 |
| RP018 | ARC 13778::IRGC 41216-1 | LL_N | DS | 83 | 144.09 | 23.17 | 41.10 | 52 | 18299.29 | 6192.67 | 0.40 | 237.94 | 97.89 |
| RP019 | ARC 14060::IRGC 41374-1 | LL_N | DS | 86 | 109.94 | 24.65 | 42.88 | 61 | 15603.36 | 5949.37 | 0.41 | 193.25 | 97.40 |
| RP020 | ARC 14064::IRGC 41377-1 | LL_N | DS | 77 | 141.44 | 26.64 | 28.07 | 52 | 15756.75 | 6136.08 | 0.51 | 237.94 | 97.97 |
| RP021 | ARC 14654::IRGC 41663-1 | LL_N | DS | 79 | 109.49 | 23.25 | 32.39 | 68 | 16795.08 | 7204.38 | 0.48 | 237.94 | 97.86 |
| RP022 | ARC 15873::IRGC 43250-1 | LL_N | DS | 77 | 139.01 | 24.61 | 58.02 | 63 | 16659.79 | 4880.63 | 0.35 | 220.06 | 97.70 |
| RP023 | ARC 18092::IRGC 42256-1 | LL_N | DS | 80 | 127.64 | 21.36 | 38.40 | 56 | 13145.77 | 4595.14 | 0.43 | 220.06 | 96.86 |
| RP024 | ARC 18112::IRGC 42274-1 | LL_N | DS | 85 | 135.98 | 22.44 | 38.09 | 59 | 16060.52 | 5711.55 | 0.44 | 220.06 | 97.53 |
| **Study designation** | **3KG database designation** | **Env** | **Season** | **DTF** | **PH (cm)** | **PL (cm)** | **FlgLA (cm^2^)** | **NBP** | **BMDW (kgha^-1^)** | **GY (kgha^-1^)** | **HI** | **TGW (g)** | **SPKFT (%)** |
| RP025 | ARC 18202::IRGC 42328-1 | LL_N | DS | 86 | 123.88 | 23.52 | 36.09 | 53 | 15792.11 | 7405.15 | 0.43 | 193.25 | 95.49 |
| RP026 | ARC 18597::IRGC 43299-1 | LL_N | DS | 75 | 108.53 | 19.86 | 25.72 | 53 | 9742.01 | 4347.71 | 0.46 | 229.00 | 96.47 |
| RP028 | ASHI BINNI::IRGC 77216-1 | LL_N | DS | 99 | 132.13 | 28.04 | 51.19 | 61 | 30915.00 | 8431.68 | 0.36 | 229.00 | 97.64 |
| RP029 | ASHMBER::IRGC 27522-1 | LL_N | DS | 93 | 122.32 | 24.74 | 35.73 | 61 | 20305.87 | 7565.18 | 0.41 | 211.12 | 97.36 |
| RP030 | ASU::IRGC 62154-1 | LL_N | DS | 86 | 125.21 | 23.90 | 54.61 | 53 | 17763.55 | 7177.40 | 0.45 | 229.00 | 97.81 |
| RP031 | AUS 171::IRGC 29004-1 | LL_N | DS | 90 | 132.13 | 22.30 | 52.19 | 49 | 11439.45 | 4589.96 | 0.37 | 237.94 | 97.23 |
| RP032 | AUS 177::IRGC 29009-1 | LL_N | DS | 95 | 102.62 | 22.03 | 52.04 | 62 | 19737.30 | 5234.83 | 0.41 | 193.25 | 96.34 |
| RP033 | AUS 219::IRGC 29031-1 | LL_N | DS | 80 | 114.81 | 22.17 | 35.59 | 47 | 9211.40 | 4021.70 | 0.40 | 211.12 | 97.50 |
| RP034 | AUS 233::IRGC 29036-1 | LL_N | DS | 80 | 122.51 | 25.01 | 34.51 | 68 | 17206.62 | 8480.53 | 0.46 | 220.06 | 98.30 |
| RP036 | AUS 295::IRGC 29083-1 | LL_N | DS | 86 | 120.20 | 25.28 | 40.36 | 48 | 11805.83 | 5720.26 | 0.42 | 202.19 | 97.64 |
| RP037 | AUS 301::IRGC 29089-1 | LL_N | DS | 80 | 100.96 | 21.62 | 25.70 | 63 | 12588.11 | 4604.60 | 0.37 | 237.94 | 97.46 |
| RP038 | AUS 308::IRGC 29096-1 | LL_N | DS | 85 | 126.94 | 23.39 | 34.58 | 57 | 10865.23 | 4168.09 | 0.37 | 211.12 | 97.90 |
| RP039 | AUS 329::IRGC 29116-1 | LL_N | DS | 83 | 115.77 | 20.14 | 23.26 | 52 | 12460.88 | 5026.14 | 0.39 | 246.87 | 97.67 |
| RP040 | AUS 344::IRGC 29131-1 | LL_N | DS | 84 | 124.20 | 20.00 | 44.64 | 56 | 13702.92 | 4918.35 | 0.34 | 246.87 | 97.53 |
| RP041 | AUS 439::IRGC 29221-1 | LL_N | DS | 87 | 111.60 | 23.11 | 54.37 | 43 | 8036.15 | 3406.45 | 0.37 | 157.50 | 96.00 |
| RP042 | AUS PADDY (RED)::IRGC 44978-1 | LL_N | DS | 79 | 103.26 | 20.81 | 32.63 | 56 | 13702.70 | 4546.55 | 0.40 | 229.00 | 98.20 |
| RP043 | B 4414 F-MR-6-3::IRGC 117310-1 | LL_N | DS | 99 | 99.41 | 21.22 | 45.38 | 53 | 13602.20 | 5614.69 | 0.36 | 202.19 | 98.11 |
| RP044 | B 6136 E-3-TB-0-1-5::IRGC 117311-1 | LL_N | DS | 95 | 104.86 | 18.78 | 33.54 | 58 | 12192.65 | 5494.29 | 0.39 | 193.25 | 97.19 |
| RP045 | B 6136-3-TB-0-1-5::IRGC 117312-1 | LL_N | DS | 96 | 94.86 | 21.62 | 38.49 | 47 | 8588.65 | 3853.83 | 0.42 | 202.19 | 96.49 |
| RP046 | B 6149 F-MR-7::IRGC 117314-1 | LL_N | DS | 93 | 124.57 | 24.06 | 48.96 | 42 | 12897.80 | 5909.48 | 0.43 | 264.75 | 96.96 |
| RP047 | BA BAI GU::IRGC 79580-1 | LL_N | DS | 77 | 112.24 | 25.14 | 25.59 | 46 | 8421.44 | 3743.10 | 0.45 | 166.44 | 98.60 |
| RP048 | BA SHI ZAO::IRGC 67903-1 | LL_N | DS | 82 | 126.77 | 23.17 | 34.52 | 54 | 16656.78 | 5878.24 | 0.41 | 255.81 | 97.82 |
| RP049 | BADAL 1163::IRGC 32796-1 | LL_N | DS | 85 | 145.37 | 21.49 | 30.31 | 66 | 23310.84 | 9470.63 | 0.43 | 264.75 | 97.35 |
| RP050 | BADUIE::IRGC 53715-1 | LL_N | DS | 90 | 132.91 | 24.79 | 39.33 | 59 | 14589.58 | 7183.81 | 0.38 | 193.25 | 97.49 |
| RP051 | BAI HE::IRGC 76438-1 | LL_N | DS | 81 | 121.49 | 24.77 | 31.78 | 61 | 15128.41 | 6258.88 | 0.43 | 237.94 | 97.93 |
| RP052 | BAIANG 6::IRGC 6129-1 | LL_N | DS | 85 | 97.49 | 22.17 | 31.63 | 60 | 13309.34 | 5084.46 | 0.41 | 237.94 | 98.41 |
| RP053 | BAK TULSI::IRGC 34831-1 | LL_N | DS | 89 | 86.58 | 22.17 | 42.97 | 44 | 8273.15 | 3856.01 | 0.38 | 237.94 | 97.70 |
| RP054 | BAKASI::IRGC 27074-1 | LL_N | DS | 95 | 109.49 | 19.32 | 41.07 | 49 | 15727.67 | 5646.72 | 0.41 | 229.00 | 97.66 |
| **Study designation** | **3KG database designation** | **Env** | **Season** | **DTF** | **PH (cm)** | **PL (cm)** | **FlgLA (cm^2^)** | **NBP** | **BMDW (kgha^-1^)** | **GY (kgha^-1^)** | **HI** | **TGW (g)** | **SPKFT (%)** |
| RP055 | BALASURIYA A::IRGC 66509-1 | LL_N | DS | 88 | 98.77 | 19.05 | 17.44 | 45 | 9586.52 | 4647.03 | 0.35 | 211.12 | 95.90 |
| RP056 | BAMOA A 75::IRGC 51101-1 | LL_N | DS | 95 | 88.50 | 26.23 | 32.04 | 57 | 7471.48 | 2297.74 | 0.27 | 211.12 | 86.83 |
| RP057 | BANDI::IRGC 17214-1 | LL_N | DS | NA | 108.66 | 20.14 | 38.78 | 69 | 22026.41 | 6122.27 | 0.31 | 229.00 | 96.34 |
| RP058 | BANGKOUY::IRGC 94037-1 | LL_N | DS | 92 | 128.28 | 25.28 | 56.81 | 45 | 15002.80 | 6896.94 | 0.37 | 291.56 | 95.23 |
| RP059 | BARIK KUDI::IRGC 52807-1 | LL_N | DS | 77 | 105.59 | 20.54 | 22.98 | 45 | 10348.83 | 3630.84 | 0.45 | 112.81 | 95.82 |
| RP060 | BAT DO::IRGC 7014-1 | LL_N | DS | 79 | 120.35 | 21.36 | 32.17 | 56 | 9523.33 | 4315.47 | 0.41 | 184.31 | 96.99 |
| RP061 | BATHURI::IRGC 25838-1 | LL_N | DS | 80 | 117.06 | 24.87 | 52.79 | 58 | 13856.64 | 5959.16 | 0.44 | 246.87 | 98.86 |
| RP062 | BAZAIL::IRGC 27526-1 | LL_N | DS | 86 | 82.41 | 21.32 | 22.48 | 56 | 18575.72 | 4311.54 | 0.42 | 220.06 | 96.52 |
| RP063 | BENGALY MORIMO::IRGC 10976-1 | LL_N | DS | 96 | 92.76 | 21.06 | 36.46 | 53 | 10889.60 | 5291.89 | 0.42 | 246.87 | 96.64 |
| RP065 | BHADOIA 303::IRGC 6588-1 | LL_N | DS | 77 | 119.62 | 19.59 | 23.00 | 64 | 16417.94 | 3636.06 | 0.34 | 193.25 | 95.44 |
| RP066 | BK 26::IRGC 45197-1 | LL_N | DS | 93 | 121.36 | 23.65 | 39.87 | 60 | 19140.32 | 5938.66 | 0.37 | 193.25 | 94.25 |
| RP067 | BKN BR 1031-78-5-4::IRGC 55927-1 | LL_N | DS | 99 | 144.65 | 23.65 | 41.43 | 69 | 25584.00 | 7315.06 | 0.35 | 273.69 | 97.44 |
| RP068 | BONG SEN::IRGC 7011-1 | LL_N | DS | 84 | 114.81 | 24.33 | 34.02 | 49 | 12778.66 | 5331.12 | 0.41 | 184.31 | 97.40 |
| RP070 | BPI 76 NON SENSITIVE (GREEN)::IRGC 9790-1 | LL_N | DS | 96 | 115.07 | 24.55 | 56.64 | 46 | 14567.04 | 4535.59 | 0.45 | 184.31 | 97.43 |
| RP071 | BR 51-115-4::IRGC 43999-1 | LL_N | DS | 92 | 112.01 | 21.62 | 49.50 | 52 | 13777.63 | 4416.60 | 0.32 | 193.25 | 96.40 |
| RP072 | BR 5230-46-4::IRGC 117318-1 | LL_N | DS | 79 | 94.73 | 20.27 | 34.92 | 50 | 9459.30 | 3987.68 | 0.40 | 229.00 | 96.45 |
| RP073 | BR IRGA 409::IRGC 55915-1 | LL_N | DS | 94 | 90.75 | 23.90 | 19.86 | 44 | 6566.43 | 2662.46 | 0.27 | 184.31 | 83.19 |
| RP074 | BW 295-5::IRGC 63098-1 | LL_N | DS | 96 | 90.19 | 20.54 | 31.41 | 61 | 13498.97 | 3646.80 | 0.37 | 202.19 | 97.05 |
| RP075 | C 1016-1::IRGC 50368-1 | LL_N | DS | 97 | 108.39 | 20.14 | 38.78 | 50 | 10541.32 | 4011.74 | 0.35 | 193.25 | 95.61 |
| RP076 | C 166-135::IRGC 50633-1 | LL_N | DS | 92 | 117.70 | 20.00 | 45.48 | 52 | 17085.93 | 9902.05 | 0.35 | 255.81 | 98.42 |
| RP077 | C 662083::IRGC 62101-1 | LL_N | DS | 89 | 147.62 | 28.39 | 34.55 | 60 | 21762.02 | 7029.41 | 0.30 | 175.37 | 97.39 |
| RP079 | CAUVERY::IRGC 45255-1 | LL_N | DS | 86 | 95.56 | 22.84 | 31.41 | 61 | 12485.45 | 4881.68 | NA | 184.31 | 96.81 |
| RP080 | CE IN TSAN::IRGC 4362-1 | LL_N | DS | 95 | 128.69 | 22.19 | 43.58 | 52 | 12330.99 | 6024.04 | 0.45 | 193.25 | 97.82 |
| RP081 | CEA 3::IRGC 116965-1 | LL_N | DS | NA | 66.69 | 15.21 | 21.66 | 64 | 17960.63 | 6059.24 | 0.35 | 193.25 | 96.10 |
| RP082 | CEMPO MANGGAR::IRGC 27107-1 | LL_N | DS | NA | 117.70 | 20.00 | 45.48 | 57 | 35716.00 | 7512.40 | 0.30 | 193.25 | 95.34 |
| RP083 | CHAKOL::IRGC 77226-1 | LL_N | DS | 86 | 116.55 | 24.87 | 46.01 | 65 | 21754.53 | 7640.26 | 0.39 | 273.69 | 97.38 |
| RP084 | CHAM LEK::IRGC 89387-1 | LL_N | DS | 86 | 123.88 | 24.79 | 34.65 | 49 | 14683.50 | 6152.83 | 0.42 | 229.00 | 96.75 |
| RP085 | CHAMA (DWARF)::IRGC 69487-1 | LL_N | DS | 95 | 74.39 | 20.41 | 39.29 | 61 | 13085.19 | 4926.50 | 0.41 | 166.44 | 97.96 |
| **Study designation** | **3KG database designation** | **Env** | **Season** | **DTF** | **PH (cm)** | **PL (cm)** | **FlgLA (cm^2^)** | **NBP** | **BMDW (kgha^-1^)** | **GY (kgha^-1^)** | **HI** | **TGW (g)** | **SPKFT (%)** |
| RP086 | CHANDARHAT::IRGC 25845-1 | LL_N | DS | 87 | 90.24 | 23.11 | 27.22 | 49 | 8887.43 | 2972.34 | 0.36 | 237.94 | 97.04 |
| RP087 | CHANDINA::IRGC 36420-1 | LL_N | DS | 87 | 120.35 | 23.52 | 37.07 | 49 | 8711.57 | 3310.22 | 0.32 | 211.12 | 92.49 |
| RP088 | CHANG LE SAN SHU ZAO::IRGC 63561-1 | LL_N | DS | 83 | 82.49 | 19.45 | 34.32 | 48 | 9321.80 | 4538.40 | 0.41 | 211.12 | 95.87 |
| RP089 | CHAO PEUAK DENG::IRGC 11602-1 | LL_N | DS | 94 | 130.85 | 25.28 | 44.66 | 52 | 23221.02 | 6694.34 | 0.40 | 246.87 | 97.89 |
| RP090 | CHI SHENG TAO::IRGC 4606-1 | LL_N | DS | 81 | 107.89 | 19.05 | 28.63 | 45 | 11251.65 | 3611.29 | 0.39 | 229.00 | 96.05 |
| RP091 | CHIAYI WU-K'O::IRGC 64974-1 | LL_N | DS | 84 | 101.28 | 23.65 | 40.95 | 46 | 11815.52 | 4655.03 | 0.44 | 229.00 | 96.97 |
| RP092 | CHIH SHEN LI::IRGC 1306-1 | LL_N | DS | 94 | 96.84 | 22.52 | 34.25 | 58 | 19284.09 | 6554.04 | 0.41 | 193.25 | 96.68 |
| RP093 | CHNNOR::IRGC 67485-1 | LL_N | DS | 83 | 118.47 | 25.82 | 33.84 | 58 | 19208.17 | 6695.95 | 0.42 | 255.81 | 97.29 |
| RP094 | CHUA DAU::IRGC 4785-1 | LL_N | DS | 80 | 117.06 | 21.08 | 21.38 | 61 | 13345.35 | 4463.34 | 0.40 | 229.00 | 97.57 |
| RP095 | CHUNGUR BALI::IRGC 25855-1 | LL_N | DS | 77 | 115.13 | 24.87 | 30.96 | 72 | 15690.92 | 7127.70 | 0.45 | 193.25 | 98.38 |
| RP096 | CICA 9::IRGC 53079-1 | LL_N | DS | 98 | 98.21 | 21.24 | 51.66 | 45 | 10060.88 | 2652.30 | 0.33 | 246.87 | 94.86 |
| RP097 | CN 44-40-7::IRGC 45368-1 | LL_N | DS | 85 | 83.83 | 19.19 | 26.60 | 49 | 8513.05 | 4053.85 | 0.47 | 202.19 | 97.63 |
| RP098 | CO 39::IRGC 51231-1 | LL_N | DS | 75 | 69.57 | 19.76 | 29.33 | 49 | 6542.66 | 2777.91 | 0.39 | 166.44 | 93.51 |
| RP099 | CR 60-10::IRGC 15777-1 | LL_N | DS | 82 | 62.33 | 19.19 | 22.58 | 55 | 7973.49 | 4004.75 | 0.37 | 220.06 | 92.23 |
| RP100 | CRILLO LA FRIA::IRGC 10793-1 | LL_N | DS | 88 | 127.73 | 21.08 | 36.44 | 51 | 10336.71 | 5716.67 | 0.50 | 229.00 | 96.75 |
| RP101 | CSR-90 IR-2::IRGC 117327-1 | LL_N | DS | 88 | 111.92 | 23.65 | 34.49 | 62 | 15812.14 | 6657.87 | 0.41 | 282.62 | 92.40 |
| RP102 | CT 9737-6-1-1-2-2P-M::IRGC 117330-1 | LL_N | DS | 86 | 84.47 | 21.76 | 42.55 | 53 | 14846.84 | 4059.54 | 0.38 | 220.06 | 95.90 |
| RP103 | CUN GU NUO::IRGC 63576-1 | LL_N | DS | 86 | 79.46 | 22.17 | 37.02 | 52 | 9192.02 | 3613.70 | 0.35 | 211.12 | 93.49 |
| RP104 | CUYAMEL 3820::IRGC 116975-1 | LL_N | DS | NA | 100.53 | 22.30 | 34.82 | 38 | 7067.62 | 1875.82 | 0.24 | 175.37 | 96.16 |
| RP105 | DA 11::IRGC 6046-1 | LL_N | DS | 99 | 100.96 | 20.95 | 26.61 | 74 | 30550.00 | 5466.25 | 0.30 | 166.44 | 95.91 |
| RP106 | DA GANG ZHAN::IRGC 67103-1 | LL_N | DS | 82 | 125.53 | 23.65 | 30.05 | 60 | 15413.87 | 5262.00 | 0.41 | 211.12 | 97.86 |
| RP107 | DA NUO (ZHAN)::IRGC 72025-1 | LL_N | DS | 93 | 140.10 | 22.70 | 50.58 | 47 | 10359.59 | 4883.82 | 0.36 | 193.25 | 96.59 |
| RP108 | DAA MANSA::IRGC 67559-1 | LL_N | DS | 95 | 91.39 | 18.78 | 33.48 | 49 | 9147.99 | 4236.55 | 0.32 | 229.00 | 95.92 |
| RP109 | DANGAR::IRGC 76296-1 | LL_N | DS | 75 | 105.45 | 22.98 | 35.59 | 58 | 14364.30 | 5849.79 | 0.43 | 220.06 | 97.77 |
| RP110 | DHANE BURWA::IRGC 10105-1 | LL_N | DS | 80 | 126.04 | 26.36 | 51.72 | 60 | 21662.04 | 7927.68 | 0.35 | 246.87 | 98.18 |
| RP111 | DISSI::IRGC 101346-1 | LL_N | DS | 92 | 123.15 | 23.84 | 52.19 | 55 | 16162.70 | 7405.08 | 0.40 | 237.94 | 98.01 |
| RP112 | DUDH KADAR::IRGC 67707-1 | LL_N | DS | 84 | 132.07 | 23.22 | 32.52 | 47 | 13590.50 | 5014.44 | 0.45 | 229.00 | 97.41 |
| RP113 | E 2024::IRGC 67958-1 | LL_N | DS | 86 | 82.22 | 18.84 | 24.72 | 61 | 8665.30 | 2449.38 | 0.40 | 166.44 | 92.20 |
| **Study designation** | **3KG database designation** | **Env** | **Season** | **DTF** | **PH (cm)** | **PL (cm)** | **FlgLA (cm^2^)** | **NBP** | **BMDW (kgha^-1^)** | **GY (kgha^-1^)** | **HI** | **TGW (g)** | **SPKFT (%)** |
| RP114 | E 2040::IRGC 67968-1 | LL_N | DS | 80 | 78.82 | 17.29 | 26.14 | 46 | 6524.50 | 3078.77 | 0.35 | 175.37 | 92.27 |
| RP115 | E DAW HAWM::IRGC 47938-1 | LL_N | DS | 99 | 146.51 | 24.52 | 44.32 | 65 | 23939.10 | 8944.85 | 0.37 | 300.50 | 97.16 |
| RP116 | ELONI::IRGC 116980-1 | LL_N | DS | 97 | 74.39 | 20.41 | 19.79 | 55 | 9878.98 | 2944.46 | 0.28 | 175.37 | 92.38 |
| RP117 | EPEAL 102::IRGC 78698-1 | LL_N | DS | 101 | 81.21 | 21.89 | 38.57 | 55 | 15448.01 | 4347.00 | 0.41 | 202.19 | 96.88 |
| RP118 | ES 21::IRGC 56171-1 | LL_N | DS | 84 | 136.31 | 26.90 | 48.56 | 51 | 14382.00 | 6211.50 | 0.44 | 327.31 | 97.76 |
| RP119 | EX FOILAEIN (NAPUTO)::IRGC 81675-1 | LL_N | DS | 102 | 96.84 | 22.14 | 20.84 | 61 | 13241.55 | 3629.96 | 0.30 | 175.37 | 96.55 |
| RP120 | FEI GAI 122::IRGC 63599-1 | LL_N | DS | 87 | 85.38 | 20.62 | 34.39 | 50 | 8161.50 | 4135.68 | 0.38 | 237.94 | 92.72 |
| RP121 | FONAIAP 2::IRGC 116985-1 | LL_N | DS | 87 | 124.52 | 23.84 | 51.88 | 47 | 13783.29 | 5858.97 | 0.42 | 202.19 | 97.29 |
| RP122 | FU ZAO XIAN::IRGC 63619-1 | LL_N | DS | 81 | 75.99 | 20.67 | 25.84 | 55 | 9356.38 | 3308.28 | 0.42 | 157.50 | 96.83 |
| RP123 | G 25::IRGC 45733-1 | LL_N | DS | 86 | 117.14 | 23.74 | 41.46 | 54 | 15448.82 | 6527.80 | 0.45 | 237.94 | 97.09 |
| RP124 | GAO JIAO BAI::IRGC 68047-1 | LL_N | DS | 73 | 98.45 | 22.46 | 33.15 | 43 | 6757.45 | 2779.34 | 0.40 | 211.12 | 97.74 |
| RP125 | GENIT::IRGC 3272-1 | LL_N | DS | 83 | 123.88 | 22.84 | 35.89 | 45 | 10425.16 | 4964.39 | 0.48 | 211.12 | 97.24 |
| RP126 | GOJOL GORIA::IRGC 26629-1 | LL_N | DS | 88 | 122.92 | 27.58 | 40.49 | 68 | 19259.68 | 6272.94 | 0.40 | 184.31 | 97.09 |
| RP127 | GOKULGANJA::IRGC 45701-1 | LL_N | DS | NA | 66.50 | 19.86 | 21.83 | 68 | 29723.00 | 6584.71 | 0.31 | 282.62 | 95.44 |
| RP128 | GUI HUA ZAO::IRGC 68060-1 | LL_N | DS | 74 | 102.70 | 21.62 | 39.62 | 45 | 7285.93 | 2646.85 | 0.37 | 211.12 | 96.44 |
| RP130 | HAWM KRUA::IRGC 64333-1 | LL_N | DS | 82 | 124.20 | 24.69 | 40.00 | 52 | 17927.74 | 7777.93 | 0.43 | 211.12 | 97.58 |
| RP131 | HD 10::IRGC 6638-1 | LL_N | DS | 93 | 151.84 | 21.54 | 27.43 | 54 | 14935.29 | NA | 0.35 | 237.94 | 97.25 |
| RP132 | HE GU TSAO::IRGC 51302-1 | LL_N | DS | 77 | 129.33 | 22.70 | 28.96 | 52 | 10663.04 | 5036.46 | 0.43 | 229.00 | 97.52 |
| RP133 | HODARAWALA::IRGC 67631-1 | LL_N | DS | 83 | 120.99 | 25.55 | 43.21 | 51 | 11398.07 | 4744.26 | 0.42 | 211.12 | 97.40 |
| RP134 | HOLOI BASH (SOLOI BASH)::IRGC 64778-1 | LL_N | DS | 89 | 123.79 | 21.49 | 44.24 | 70 | 21541.36 | 7692.59 | 0.42 | 211.12 | 97.86 |
| RP135 | HTA 22::IRGC 45827-1 | LL_N | DS | 77 | 135.11 | 22.44 | 35.33 | 52 | 18189.04 | 6028.31 | 0.40 | 246.87 | 97.79 |
| RP136 | HUA LI ZAO::IRGC 80950-1 | LL_N | DS | 92 | 126.49 | 24.65 | 45.64 | 56 | 16462.30 | 7920.94 | 0.42 | 220.06 | 96.58 |
| RP137 | I KUNG PAO::IRGC 114-1 | LL_N | DS | 88 | 116.82 | 21.00 | 36.31 | 51 | 14955.70 | 5676.82 | 0.41 | 220.06 | 97.07 |
| RP138 | IA CUBA 17::IRGC 116990-1 | LL_N | DS | 96 | 85.06 | 19.19 | 46.51 | 67 | 18008.11 | 4973.28 | 0.36 | 255.81 | 94.21 |
| RP139 | ICTA CRISPO 38::IRGC 116994-1 | LL_N | DS | 93 | 95.33 | 20.54 | 33.50 | 45 | 8481.36 | 3485.90 | 0.37 | 202.19 | 96.17 |
| RP140 | ICTA MOTAGUA::IRGC 116995-1 | LL_N | DS | 102 | 87.30 | 17.42 | 31.70 | 43 | 9656.61 | 2451.90 | 0.34 | 193.25 | 95.85 |
| RP141 | INIAP 6::IRGC 117002-1 | LL_N | DS | 102 | 91.65 | 20.30 | 39.95 | 58 | 16532.24 | 5516.77 | 0.33 | 211.12 | 96.31 |
| RP142 | IR 13429-109-2-2-1::IRGC 63491-1 | LL_N | DS | 94 | 76.89 | 20.54 | 25.69 | 55 | 8035.36 | 2492.82 | 0.32 | 175.37 | 92.34 |
| **Study designation** | **3KG database designation** | **Env** | **Season** | **DTF** | **PH (cm)** | **PL (cm)** | **FlgLA (cm^2^)** | **NBP** | **BMDW (kgha^-1^)** | **GY (kgha^-1^)** | **HI** | **TGW (g)** | **SPKFT (%)** |
| RP143 | IR 21015-72-3-3-3-1::IRGC 117004-1 | LL_N | DS | 95 | 82.99 | 22.17 | 37.92 | 49 | 10040.14 | 3901.27 | 0.41 | 193.25 | 96.10 |
| RP144 | IR 2344-P1 PB-9-3-2B::IRGC 39317-C1 | LL_N | DS | 77 | 107.05 | 19.86 | 31.94 | 47 | 9386.68 | 3700.98 | 0.40 | 193.25 | 95.99 |
| RP146 | IR 63295-AC 209-7::IRGC 117365-1 | LL_N | DS | 94 | 100.19 | 20.41 | 37.61 | 56 | 12069.47 | 3766.40 | 0.38 | 255.81 | 96.87 |
| RP147 | IR 69502-6-SRN-3-UBN-1-B::IRGC 117290-1 | LL_N | DS | 90 | 99.03 | 22.76 | 35.12 | 48 | 11643.47 | 5867.54 | 0.47 | 211.12 | 94.13 |
| RP148 | IR 75870-5-8-5-B-1::IRGC 117297-1 | LL_N | DS | 96 | 86.90 | 23.25 | 30.57 | 63 | 11304.22 | 3104.56 | 0.35 | 193.25 | 94.39 |
| RP149 | IR 77390-1-6-4-19-1-B::IRGC 117303-1 | LL_N | DS | NA | 90.51 | 16.83 | 24.89 | 52 | 12079.79 | 2574.14 | 0.32 | 202.19 | 94.53 |
| RP150 | IR 80310-12-B-1-3-B::IRGC 117307-1 | LL_N | DS | 95 | 111.92 | 26.90 | 52.07 | 45 | 9275.54 | 4466.86 | 0.34 | 220.06 | 97.28 |
| RP151 | IR 80340-23-B-12-6-B::IRGC 117309-1 | LL_N | DS | 89 | 91.39 | 21.22 | 35.76 | 54 | 11084.11 | 4284.96 | 0.32 | 220.06 | 93.45 |
| RP152 | IRGA 318-11-6-9-2B::IRGC 117339-1 | LL_N | DS | 87 | 87.48 | 24.87 | 33.08 | 49 | 12055.01 | 5627.05 | 0.44 | 220.06 | 97.24 |
| RP153 | IRGA 370-38-1-1F-C4-2::IRGC 117342-1 | LL_N | DS | 85 | 80.74 | 17.56 | 28.27 | 52 | 7524.47 | 2715.41 | 0.31 | 184.31 | 91.47 |
| RP154 | IRGA 370-42-1-1F-C-1::IRGC 117343-1 | LL_N | DS | 87 | 81.12 | 21.22 | 34.46 | 51 | 7167.04 | 3854.85 | 0.57 | 220.06 | 94.64 |
| RP155 | IRGA 659-1-2-2-2::IRGC 117345-1 | LL_N | DS | 98 | 86.98 | 19.84 | 31.41 | 46 | 11119.00 | 4778.76 | 0.38 | 229.00 | 95.68 |
| RP156 | IRGA 959-1-2-2F-4-1-4A-6-CA-6X::IRGC 117006-1 | LL_N | DS | 85 | 78.56 | 23.65 | 39.56 | 49 | 7779.63 | 2528.69 | 0.28 | 175.37 | 88.63 |
| RP157 | IRI 339::IRGC 46956-1 | LL_N | DS | 96 | 70.86 | 18.37 | 36.58 | 48 | 7777.68 | 2842.42 | 0.28 | 193.25 | 91.14 |
| RP158 | IRRIBINI::IRGC 49094-1 | LL_N | DS | 94 | 130.53 | 27.64 | 55.00 | 61 | 16784.99 | 6229.29 | 0.38 | 220.06 | 98.16 |
| RP159 | JABOR SAIL::IRGC 66831-1 | LL_N | DS | 86 | 124.25 | 23.79 | 48.09 | 56 | 14393.30 | 4269.44 | 0.39 | 229.00 | 97.40 |
| RP160 | JAGLI BORO::IRGC 27516-2 | LL_N | DS | 79 | 100.69 | 20.95 | 26.61 | 78 | 23218.13 | 9314.76 | 0.29 | 193.25 | 96.28 |
| RP161 | JAMBALI::IRGC 73101-1 | LL_N | DS | 83 | 114.30 | 19.59 | 34.66 | 60 | 17112.11 | 5358.06 | 0.42 | 220.06 | 97.40 |
| RP162 | JAO LEUANG::IRGC 65866-1 | LL_N | DS | 86 | 134.32 | 25.20 | 32.83 | 57 | 19320.52 | 5359.52 | 0.35 | 309.43 | 98.02 |
| RP164 | JHODI BIRUN::IRGC 31812-1 | LL_N | DS | 85 | 113.85 | 27.85 | 38.71 | 65 | 15864.18 | 6849.02 | 0.45 | 202.19 | 97.42 |
| RP165 | JIN JUN DAO::IRGC 59710-1 | LL_N | DS | 74 | 77.09 | 16.77 | 21.84 | 47 | 5208.64 | 1924.28 | 0.39 | 184.31 | 95.27 |
| RP166 | JUMA 62::IRGC 117011-1 | LL_N | DS | 96 | 69.98 | 18.19 | 27.47 | 52 | 11650.48 | 3374.36 | 0.34 | 246.87 | 94.54 |
| RP167 | KALABAIL::IRGC 25877-1 | LL_N | DS | 96 | 130.62 | 23.39 | 51.11 | 70 | 30572.00 | 5040.73 | 0.27 | 282.62 | 96.62 |
| RP168 | KALIA::IRGC 34699-1 | LL_N | DS | 87 | 87.09 | 17.42 | 31.38 | 53 | 11597.76 | 4269.06 | 0.42 | 220.06 | 96.61 |
| RP169 | KALO CHAKOL::IRGC 77258-1 | LL_N | DS | 108 | 87.16 | 23.25 | 30.57 | 69 | 37876.00 | 3827.63 | 0.22 | 273.69 | 95.48 |
| RP170 | KALU ILANKALAYAN::IRGC 36270-1 | LL_N | DS | 107 | 98.45 | 16.67 | 30.99 | 70 | 22238.42 | 7934.33 | 0.41 | 229.00 | 98.69 |
| RP171 | KAM PAI::IRGC 78245-1 | LL_N | DS | 86 | 137.99 | 26.90 | 59.67 | 44 | 13280.88 | 6766.93 | 0.49 | 345.18 | 96.01 |
| **Study designation** | **3KG database designation** | **Env** | **Season** | **DTF** | **PH (cm)** | **PL (cm)** | **FlgLA (cm^2^)** | **NBP** | **BMDW (kgha^-1^)** | **GY (kgha^-1^)** | **HI** | **TGW (g)** | **SPKFT (%)** |
| RP172 | KEERIPALA CHILL PADDY::IRGC 49790-1 | LL_N | DS | 82 | 123.56 | 23.92 | 34.06 | 56 | 15686.70 | 6284.40 | 0.46 | 220.06 | 96.92 |
| RP173 | KETAN SERANG::IRGC 14615-1 | LL_N | DS | 105 | 155.32 | 26.77 | 49.04 | 58 | 19836.40 | 6697.14 | 0.39 | 193.25 | 97.70 |
| RP174 | KHAO DAWK MALI 105::IRGC 27748-2 | LL_N | DS | 91 | 136.89 | 23.92 | 48.31 | 58 | 20408.12 | 7067.87 | 0.37 | 318.37 | 96.95 |
| RP175 | KHAO GRADOOK CHAHNG::IRGC 17111-1 | LL_N | DS | 93 | 134.06 | 25.47 | 37.64 | 57 | 14536.74 | 5280.00 | 0.41 | 237.94 | 97.45 |
| RP176 | KHAO' HAWM::IRGC 78257-1 | LL_N | DS | NA | 86.90 | 23.25 | 30.57 | 60 | 27801.00 | 2076.45 | 0.19 | 229.00 | 90.60 |
| RP177 | KHAO THI RATE::IRGC 58041-1 | LL_N | DS | 79 | 95.82 | 18.24 | 26.54 | 48 | 10549.00 | 5065.64 | 0.44 | 282.62 | 97.32 |
| RP178 | KHARSU 80::IRGC 28016-1 | LL_N | DS | 75 | 73.10 | 19.86 | 33.24 | 42 | 7634.71 | 2279.20 | 0.38 | 220.06 | 98.27 |
| RP179 | KITRANA 1007::IRGC 68517-1 | LL_N | DS | 99 | 130.85 | 21.62 | 42.14 | 65 | 15648.98 | 4904.34 | 0.35 | 202.19 | 93.08 |
| RP180 | KN 1 B 361-1-8-6-9::IRGC 46974-1 | LL_N | DS | 92 | 97.49 | 20.08 | 30.37 | 49 | 14293.95 | 6104.06 | 0.41 | 237.94 | 95.95 |
| RP181 | KORASISI::IRGC 5285-1 | LL_N | DS | 87 | 153.15 | 24.47 | 40.92 | 56 | 18145.20 | 4562.92 | 0.35 | 237.94 | 96.66 |
| RP182 | KOTTEYARAN::IRGC 47383-1 | LL_N | DS | 80 | 124.05 | 22.01 | 34.30 | 51 | 14668.01 | 6146.25 | 0.45 | 246.87 | 97.32 |
| RP183 | KOYRA::IRGC 77267-1 | LL_N | DS | 85 | 109.62 | 20.81 | 42.21 | 49 | 11148.34 | 3652.24 | 0.33 | 193.25 | 97.85 |
| RP184 | KULA KARUPPAN::IRGC 55328-1 | LL_N | DS | 96 | 142.16 | 23.44 | 46.11 | 49 | 14082.95 | 7316.03 | 0.40 | 246.87 | 97.46 |
| RP185 | KUNENG::IRGC 71545-1 | LL_N | DS | 105 | 79.52 | 18.24 | 38.11 | 60 | 25769.00 | 4729.41 | 0.25 | 237.94 | 96.29 |
| RP186 | KURULU WEE (WHITE)::IRGC 66518-1 | LL_N | DS | 77 | 103.26 | 25.28 | 38.02 | 52 | 17238.12 | 5879.22 | 0.42 | 193.25 | 95.80 |
| RP188 | KUSHIARA::IRGC 34709-1 | LL_N | DS | 92 | 126.17 | 25.14 | 45.16 | 74 | 26634.00 | NA | 0.41 | 282.62 | 96.99 |
| RP189 | KUTTA::IRGC 52184-1 | LL_N | DS | 89 | 162.06 | 26.64 | 47.51 | 52 | 16271.90 | 5280.51 | 0.35 | 211.12 | 97.79 |
| RP190 | LABRA::IRGC 74757-1 | LL_N | DS | 89 | 128.05 | 22.87 | 49.95 | 51 | 16165.28 | 5760.78 | 0.37 | 255.81 | 98.09 |
| RP191 | LAI YIP ZIM::IRGC 4955-1 | LL_N | DS | 85 | 138.23 | 23.52 | 54.94 | 54 | 21338.21 | 7154.51 | 0.39 | 193.25 | 97.55 |
| RP192 | LALKA (LAL DHAN)::IRGC 64946-1 | LL_N | DS | 89 | 138.55 | 26.15 | 50.74 | 44 | 13027.03 | 4906.43 | 0.36 | 246.87 | 96.16 |
| RP193 | LALSAITA::IRGC 43915-1 | LL_N | DS | 83 | 111.92 | 22.03 | 42.10 | 55 | 11568.75 | 4961.18 | 0.43 | 237.94 | 98.81 |
| RP194 | LARHA MUGAD::IRGC 52339-1 | LL_N | DS | 77 | 108.85 | 18.64 | 28.60 | 48 | 8398.35 | 3197.12 | 0.37 | 237.94 | 93.79 |
| RP196 | LENJA MURALI::IRGC 66815-1 | LL_N | DS | 77 | 126.81 | 27.58 | 57.43 | 44 | 18080.58 | 6639.62 | 0.43 | 229.00 | 96.71 |
| RP197 | LEUANG YAI 29-12-2::IRGC 881-1 | LL_N | DS | 86 | 127.46 | 22.30 | 40.09 | 61 | 22628.49 | 5535.94 | 0.28 | 300.50 | 96.88 |
| RP198 | LU MAO ZHAN::IRGC 68159-1 | LL_N | DS | 75 | 94.09 | 19.65 | 21.68 | 50 | 5685.19 | 2353.51 | 0.39 | 202.19 | 94.18 |
| RP199 | LUA CHAN HUONG::IRGC 16800-1 | LL_N | DS | 89 | 78.05 | 18.68 | 18.59 | 51 | 20126.15 | 4502.10 | 0.31 | 237.94 | 97.08 |
| RP200 | MAKALIOKA::IRGC 376-1 | LL_N | DS | 80 | 108.39 | 21.62 | 34.30 | 52 | 14758.87 | 5578.79 | 0.44 | 193.25 | 98.16 |
| RP201 | MAKRO::IRGC 74763-1 | LL_N | DS | 86 | 128.61 | 22.98 | 45.35 | 50 | 13450.75 | 8149.92 | 0.40 | 246.87 | 98.09 |
| **Study designation** | **3KG database designation** | **Env** | **Season** | **DTF** | **PH (cm)** | **PL (cm)** | **FlgLA (cm^2^)** | **NBP** | **BMDW (kgha^-1^)** | **GY (kgha^-1^)** | **HI** | **TGW (g)** | **SPKFT (%)** |
| RP202 | MAMORIAKA::IRGC 68672-1 | LL_N | DS | 89 | 126.12 | 18.35 | 35.20 | 56 | 18005.68 | 5427.08 | 0.42 | 211.12 | 96.76 |
| RP203 | MEI FENG 9::IRGC 63735-1 | LL_N | DS | 77 | 84.01 | 20.14 | 18.97 | 48 | 8416.19 | 2996.99 | 0.39 | 255.81 | 94.67 |
| RP204 | MEKENZIE SMALL::IRGC 49895-1 | LL_N | DS | 94 | 91.07 | 21.36 | 30.39 | 54 | 16902.91 | 5128.57 | 0.34 | 184.31 | 97.30 |
| RP205 | MELEKE::IRGC 56823-1 | LL_N | DS | 85 | 121.04 | 24.61 | 45.59 | 57 | 16302.77 | 7400.82 | 0.43 | 264.75 | NA |
| RP206 | MENTIK TJERE BELUT::IRGC 18254-1 | LL_N | DS | 96 | 152.19 | 24.47 | 54.61 | 61 | 20204.50 | 5302.90 | 0.31 | 211.12 | 97.77 |
| RP207 | MILYANG 30::IRGC 46977-1 | LL_N | DS | 94 | 78.56 | 19.59 | 32.98 | 59 | 9062.30 | 3415.96 | 0.32 | 202.19 | 92.48 |
| RP208 | MILYANG 77::IRGC 69340-1 | LL_N | DS | 87 | 73.42 | 18.37 | 25.69 | 43 | 4909.44 | 2456.63 | 0.25 | 148.56 | 87.28 |
| RP209 | MIN KE ZHAN::IRGC 72230-1 | LL_N | DS | 83 | 81.85 | 22.09 | 32.63 | 46 | 10190.52 | 3964.17 | 0.42 | 166.44 | 96.72 |
| RP210 | MIN ZAO 6::IRGC 63772-1 | LL_N | DS | 82 | 74.20 | 20.06 | 36.09 | 48 | 7921.39 | 3535.15 | 0.41 | 211.12 | 94.43 |
| RP211 | MODDAI KARUPPAN::IRGC 15465-1 | LL_N | DS | 86 | 145.10 | 25.06 | 39.11 | 57 | 18845.50 | 6092.34 | 0.39 | 282.62 | 98.02 |
| RP212 | MUKKALA BAZAL::IRGC 77279-1 | LL_N | DS | 96 | 95.33 | 21.54 | 31.21 | 57 | 20395.75 | 8191.61 | 0.42 | 255.81 | 97.89 |
| RP213 | MULLIKURUVA::IRGC 77529-1 | LL_N | DS | 92 | 153.31 | 25.01 | 35.73 | 61 | 20292.54 | 6639.12 | 0.39 | 166.44 | 98.25 |
| RP214 | MUTA GANJE::IRGC 26744-1 | LL_N | DS | 83 | 117.83 | 25.06 | 30.11 | 54 | 17374.63 | 6944.74 | 0.44 | 220.06 | 97.74 |
| RP215 | MUTTU SAMBA::IRGC 36333-1 | LL_N | DS | 98 | 144.46 | 23.25 | 44.55 | 54 | 22621.22 | 6772.55 | 0.40 | 157.50 | 96.89 |
| RP216 | N 22::IRGC 46459-1 | LL_N | DS | 89 | 120.20 | 24.20 | 37.48 | 43 | 9558.73 | 4918.38 | 0.39 | 202.19 | 97.94 |
| RP217 | Na souan::IRGC 11889-1 | LL_N | DS | 85 | 118.79 | 24.47 | 45.88 | 52 | 14767.69 | 5675.39 | 0.40 | 354.12 | 96.43 |
| RP218 | NAZIRA SAIL::IRGC 77284-1 | LL_N | DS | 92 | 121.81 | 25.34 | 47.17 | 54 | 22063.56 | 8026.58 | 0.44 | 175.37 | 97.19 |
| RP219 | NCS 194::IRGC 51932-1 | LL_N | DS | 92 | 88.82 | 17.81 | 35.82 | 54 | 15511.20 | 7019.78 | 0.44 | 202.19 | 98.33 |
| RP220 | NCS 237::IRGC 62202-1 | LL_N | DS | 93 | 121.49 | 24.79 | 54.58 | 59 | 20202.96 | 6985.85 | 0.36 | 202.19 | 97.79 |
| RP222 | NCS 964 C::IRGC 62604-1 | LL_N | DS | NA | 94.60 | 16.89 | 30.47 | 51 | 14413.16 | 4359.67 | 0.34 | 202.19 | 97.07 |
| RP223 | NIAO YAO::IRGC 5496-1 | LL_N | DS | 80 | 126.04 | 25.42 | 36.42 | 61 | 18417.87 | 6873.46 | 0.42 | 211.12 | 96.92 |
| RP224 | NIBARI::IRGC 67742-1 | LL_N | DS | 80 | 118.02 | 22.03 | 43.44 | 45 | 11161.18 | 7313.73 | 0.45 | 202.19 | 97.72 |
| RP225 | NONA BOKRA::IRGC 22710-C1 | LL_N | DS | 77 | 113.53 | 28.15 | 39.17 | 59 | 15323.40 | 5508.52 | 0.46 | 255.81 | 97.68 |
| RP226 | NOROI::IRGC 31611-1 | LL_N | DS | 77 | 112.50 | 20.41 | 30.65 | 48 | 8781.70 | 3548.99 | 0.39 | 264.75 | 97.14 |
| RP228 | NX 3533::IRGC 63796-1 | LL_N | DS | 98 | 67.97 | 16.48 | 41.43 | 55 | 8017.58 | 2517.79 | 0.31 | 237.94 | 88.93 |
| RP229 | O. SATIVA::IRGC 17083-1 | LL_N | DS | 89 | 136.30 | 28.67 | 40.90 | 49 | 13685.80 | 4525.45 | 0.28 | 202.19 | 96.06 |
| RP230 | PAH WEAN::IRGC 78276-1 | LL_N | DS | 92 | 91.07 | 16.83 | 24.89 | 50 | 16732.48 | 8306.21 | 0.57 | 282.62 | 95.65 |
| RP231 | PAI CHUEH CHIU LIU::IRGC 34259-1 | LL_N | DS | 90 | 81.90 | 19.19 | 43.10 | 55 | 12027.87 | 4240.27 | 0.36 | 211.12 | 95.68 |
| **Study designation** | **3KG database designation** | **Env** | **Season** | **DTF** | **PH (cm)** | **PL (cm)** | **FlgLA (cm^2^)** | **NBP** | **BMDW (kgha^-1^)** | **GY (kgha^-1^)** | **HI** | **TGW (g)** | **SPKFT (%)** |
| RP232 | PAI YI PING::IRGC 1368-1 | LL_N | DS | 80 | 66.37 | 19.86 | 21.83 | 52 | 10781.55 | 4847.88 | 0.38 | 166.44 | 95.18 |
| RP233 | PALEPYU::IRGC 33549-1 | LL_N | DS | 74 | 99.03 | 22.44 | 28.92 | 59 | 11571.59 | 4277.31 | 0.41 | 220.06 | 96.85 |
| RP234 | PARA NELLU::IRGC 50009-1 | LL_N | DS | 75 | 127.58 | 23.65 | 27.78 | 55 | 14289.59 | 5908.81 | 0.43 | 255.81 | 97.76 |
| RP235 | PATISAIL::IRGC 37562-1 | LL_N | DS | NA | 78.69 | 23.65 | 39.56 | 68 | 27960.00 | 6971.78 | 0.36 | 166.44 | 97.37 |
| RP237 | PERUNEL::IRGC 63113-1 | LL_N | DS | 86 | 115.27 | 22.98 | 35.60 | 53 | 13882.01 | 5645.72 | 0.44 | 255.81 | 96.54 |
| RP238 | PICO NEGRO::IRGC 55849-1 | LL_N | DS | NA | 70.54 | 18.19 | 27.47 | 68 | 31130.00 | 7917.96 | 0.37 | 309.43 | 97.79 |
| RP239 | PLI KHAO::IRGC 64596-1 | LL_N | DS | 95 | 147.85 | 25.14 | 59.09 | 53 | 17628.32 | 4513.47 | 0.38 | 246.87 | 95.33 |
| RP240 | PODI HEENATI::IRGC 36345-1 | LL_N | DS | 85 | 101.98 | 22.44 | 31.68 | 65 | 15043.19 | 5257.60 | 0.44 | 148.56 | 98.38 |
| RP241 | PSBRC 50::IRGC 99706-1 | LL_N | DS | 96 | 96.14 | 21.54 | 31.21 | 58 | 17939.31 | 7692.32 | 0.41 | 237.94 | 97.03 |
| RP242 | PSBRC 68::IRGC 99711-1 | LL_N | DS | 96 | 112.33 | 25.14 | 53.58 | 49 | 15469.42 | 6865.77 | 0.42 | 282.62 | 97.29 |
| RP243 | PSBRC 88::IRGC 99717-1 | LL_N | DS | 101 | 100.96 | 24.47 | 38.51 | 60 | 12537.63 | 5057.84 | 0.40 | 211.12 | 97.71 |
| RP244 | PULUT BARAYA::IRGC 27393-1 | LL_N | DS | 97 | 79.52 | 18.24 | 38.11 | 71 | 21700.82 | 4422.45 | 0.29 | 121.75 | 96.44 |
| RP245 | PURA BINNI::IRGC 26772-1 | LL_N | DS | 98 | 133.74 | 23.82 | 36.19 | 53 | 15463.54 | 5170.55 | 0.35 | 175.37 | 97.77 |
| RP247 | QUERO ASSAN::IRGC 28860-1 | LL_N | DS | 89 | 124.89 | 23.63 | 54.61 | 67 | 20248.52 | 7384.46 | 0.44 | 273.69 | 98.30 |
| RP248 | RACE PERUMAL::IRGC 55347-1 | LL_N | DS | 79 | 106.79 | 20.95 | 39.68 | 49 | 12976.62 | 5380.70 | 0.43 | 246.87 | 98.70 |
| RP250 | RELLY::IRGC 14623-1 | LL_N | DS | NA | 66.37 | 19.86 | 21.83 | 52 | 31378.00 | 6688.46 | 0.35 | 220.06 | 97.01 |
| RP251 | RIZ TYPE SORGHO::IRGC 69015-1 | LL_N | DS | 77 | 82.67 | 19.05 | 23.17 | 44 | 7155.34 | 2834.46 | 0.31 | 220.06 | 90.22 |
| RP252 | ROJOFOTSY::IRGC 69402-1 | LL_N | DS | 94 | 149.40 | 25.28 | 47.51 | 62 | 17108.56 | 4398.26 | 0.32 | 255.81 | 96.08 |
| RP253 | RPA 5929 (K 45)::IRGC 33963-1 | LL_N | DS | 96 | 77.86 | 19.73 | 47.27 | 41 | 8600.36 | 3500.33 | 0.38 | 193.25 | 94.24 |
| RP254 | RPW 9-4 (SS 1)::IRGC 50690-1 | LL_N | DS | 96 | 82.17 | 22.44 | 32.42 | 60 | 14551.84 | 4169.83 | 0.34 | 220.06 | 93.24 |
| RP255 | RR 272-17-829::IRGC 117354-1 | LL_N | DS | 87 | 87.54 | 20.41 | 29.25 | 50 | 12669.68 | 3959.09 | 0.40 | 175.37 | 96.12 |
| RP256 | RTS 16::IRGC 8235-1 | LL_N | DS | 83 | 106.09 | 23.79 | 38.94 | 64 | 14869.13 | 5511.85 | 0.42 | 175.37 | 97.62 |
| RP257 | RUSTIC::IRGC 117026-1 | LL_N | DS | 100 | 67.27 | 17.56 | 18.93 | 49 | 7758.30 | 1846.77 | 0.19 | 229.00 | NA |
| RP258 | SADA RUPA::IRGC 77299-1 | LL_N | DS | 99 | 110.45 | 21.89 | 38.26 | 52 | 16887.16 | 6830.90 | 0.41 | 264.75 | 96.77 |
| RP259 | SAN CHIAO TSWEN::IRGC 1565-1 | LL_N | DS | 93 | 134.32 | 22.98 | 43.73 | 61 | 22397.56 | 7597.45 | 0.38 | 193.25 | 96.37 |
| RP260 | SAN SHIH TSI::IRGC 1038-1 | LL_N | DS | 93 | 124.52 | 21.22 | 56.49 | 46 | 11831.41 | 4874.23 | 0.37 | 193.25 | 96.14 |
| RP261 | SIGARDIS::IRGC 15555-1 | LL_N | DS | 93 | 70.67 | 18.19 | 27.47 | 52 | 27893.00 | NA | NA | 220.57 | 95.99 |
| RP262 | SIMET 2::IRGC 25734-1 | LL_N | DS | 99 | 87.16 | 23.25 | 30.57 | 62 | 27646.00 | 5289.79 | 0.31 | 184.31 | 97.26 |
| **Study designation** | **3KG database designation** | **Env** | **Season** | **DTF** | **PH (cm)** | **PL (cm)** | **FlgLA (cm^2^)** | **NBP** | **BMDW (kgha^-1^)** | **GY (kgha^-1^)** | **HI** | **TGW (g)** | **SPKFT (%)** |
| RP263 | SIMUL KHURI::IRGC 35154-1 | LL_N | DS | 86 | 118.34 | 21.62 | 45.32 | 60 | 14479.80 | 4906.76 | 0.42 | 246.87 | 98.42 |
| RP264 | SINNA SITHIRA KALI::IRGC 51064-1 | LL_N | DS | 98 | 112.38 | 20.22 | 21.94 | 48 | 28376.00 | 4670.02 | 0.24 | 220.06 | 95.54 |
| RP266 | SITHAIYAN KOTTAI SAMBA::IRGC 50155-1 | LL_N | DS | 87 | 130.94 | 27.74 | 47.46 | 66 | 19834.38 | 6932.65 | 0.35 | 166.44 | 96.79 |
| RP267 | SML AWINI::IRGC 13391-1 | LL_N | DS | 102 | 68.61 | 16.89 | 18.79 | 52 | 5960.70 | 2482.84 | 0.30 | 220.06 | 94.59 |
| RP268 | SOLOMON RED RICE::IRGC 50950-1 | LL_N | DS | 95 | 130.94 | 23.31 | 48.56 | 68 | 27579.00 | 6393.32 | 0.39 | 211.12 | 95.14 |
| RP269 | SONA::IRGC 26971-C1 | LL_N | DS | 98 | 61.49 | 19.05 | 37.98 | 45 | 6562.06 | 1783.24 | 0.23 | 148.56 | 91.60 |
| RP270 | SONAMUKHI::IRGC 46693-1 | LL_N | DS | 79 | 102.62 | 19.19 | 32.36 | 43 | 8564.81 | 3544.33 | 0.41 | 184.31 | 97.19 |
| RP272 | SSANGDUJO::IRGC 55632-1 | LL_N | DS | 83 | 98.45 | 21.89 | 28.81 | 55 | 13785.49 | 5292.16 | 0.37 | 184.31 | 96.79 |
| RP273 | SUFAID 246::IRGC 28303-1 | LL_N | DS | 85 | 97.81 | 23.52 | 38.76 | 52 | 8156.03 | 4664.67 | 0.56 | 184.31 | 96.17 |
| RP274 | SUWEON 311::IRGC 61890-1 | LL_N | DS | 85 | 113.15 | 23.82 | 38.57 | 50 | 11675.38 | 6532.81 | NA | 211.12 | 97.02 |
| RP275 | T 315::IRGC 54792-1 | LL_N | DS | 85 | 118.34 | 23.65 | 57.82 | 55 | 12329.56 | 5105.09 | 0.43 | 264.75 | 97.89 |
| RP276 | TAIPEI WOO CO::IRGC 112-1 | LL_N | DS | 94 | 90.43 | 19.59 | 21.12 | 46 | 12574.71 | 6047.14 | 0.38 | 175.37 | 95.35 |
| RP277 | TAITUNG WOO LI::IRGC 111-1 | LL_N | DS | 98 | 144.14 | 20.59 | 38.95 | 50 | 19080.95 | 5937.23 | 0.40 | 229.00 | 96.68 |
| RP278 | TAK SUFAID::IRGC 73127-1 | LL_N | DS | 84 | 74.39 | 15.88 | 24.38 | 52 | 11569.89 | 4471.37 | 0.36 | 193.25 | 95.09 |
| RP279 | TAK::IRGC 73124-1 | LL_N | DS | 84 | 114.25 | 18.92 | 31.07 | 60 | 15971.42 | 7314.61 | 0.41 | 148.56 | 97.61 |
| RP280 | TIKAL 3::IRGC 50649-1 | LL_N | DS | 93 | 85.88 | 22.17 | 46.89 | 59 | 13026.71 | 4851.44 | 0.44 | 255.81 | 96.08 |
| RP281 | TOC 5430::IRGC 70487-1 | LL_N | DS | 90 | 85.88 | 23.17 | 30.12 | 50 | 12667.67 | 3201.61 | 0.39 | 220.06 | 94.81 |
| RP282 | TONG GU HONG::IRGC 81026-1 | LL_N | DS | 74 | 106.09 | 22.98 | 34.76 | 55 | 9363.66 | 3666.32 | 0.40 | 193.25 | 96.14 |
| RP283 | TSAI YUAN CHON::IRGC 126-1 | LL_N | DS | 89 | 120.85 | 25.28 | 48.50 | 53 | 13792.43 | 6564.12 | 0.47 | 193.25 | 96.98 |
| RP284 | TSAO SHENG LI 1::IRGC 1309-1 | LL_N | DS | 94 | 128.10 | 24.79 | 45.59 | 58 | 24757.00 | 9522.56 | 0.40 | 282.62 | 96.58 |
| RP285 | UPRH 233::IRGC 61667-1 | LL_N | DS | 80 | 71.63 | 19.59 | 30.65 | 43 | 7213.41 | 3316.78 | 0.43 | 202.19 | 95.97 |
| RP286 | UQUIHUA::IRGC 117037-1 | LL_N | DS | 102 | 75.29 | 16.69 | 16.07 | 66 | 19732.06 | 4254.32 | 0.33 | 237.94 | 95.84 |
| RP287 | URAIBOOL::IRGC 52785-1 | LL_N | DS | 94 | 91.07 | 16.83 | 24.89 | 54 | 18354.00 | NA | 0.44 | 220.06 | 97.65 |
| RP288 | VEN THAP::IRGC 56138-1 | LL_N | DS | 83 | 121.81 | 20.92 | 30.88 | 58 | 14911.94 | 7008.96 | 0.43 | 220.06 | 98.01 |
| RP289 | WANGA BARUGULU::IRGC 52261-1 | LL_N | DS | 85 | 133.74 | 23.65 | 33.41 | 53 | 14320.61 | 4608.46 | 0.41 | 193.25 | 97.67 |
| RP290 | WAR 72-2-1-1::IRGC 117361-1 | LL_N | DS | 83 | 114.17 | 23.79 | 35.09 | 61 | 12506.12 | 4906.11 | 0.44 | 229.00 | 97.32 |
| RP291 | WI BIR SHUN::IRGC 4602-1 | LL_N | DS | 94 | 131.17 | 23.52 | 36.80 | 67 | 19047.90 | 7513.82 | 0.42 | 193.25 | 98.78 |
| RP292 | WP 36::IRGC 55278-1 | LL_N | DS | NA | 91.20 | 17.29 | 19.34 | 69 | 15623.47 | NA | 0.31 | 264.75 | 96.27 |
| **Study designation** | **3KG database designation** | **Env** | **Season** | **DTF** | **PH (cm)** | **PL (cm)** | **FlgLA (cm^2^)** | **NBP** | **BMDW (kgha^-1^)** | **GY (kgha^-1^)** | **HI** | **TGW (g)** | **SPKFT (%)** |
| RP293 | WP 65::IRGC 36526-1 | LL_N | DS | 99 | 67.78 | 17.42 | 29.58 | 49 | 12136.51 | 4662.91 | 0.40 | 237.94 | 96.61 |
| RP294 | XI GAN JING REN::IRGC 60035-1 | LL_N | DS | 73 | 89.41 | 19.45 | 33.71 | 49 | 7529.72 | 2529.98 | 0.39 | 211.12 | 97.29 |
| RP295 | XIA ZHI BAI::IRGC 53437-1 | LL_N | DS | 73 | 97.17 | 20.41 | 42.06 | 61 | 9278.44 | 3264.14 | 0.43 | 139.63 | 98.98 |
| RP296 | XITTO::IRGC 6671-1 | LL_N | DS | 85 | 138.81 | 25.06 | 34.13 | 48 | 8580.57 | 4165.12 | 0.52 | 282.62 | 95.43 |
| RP297 | YA NONG ZAO 4::IRGC 63908-1 | LL_N | DS | 87 | 78.96 | 20.41 | 37.07 | 47 | 6048.09 | 2740.90 | 0.37 | 237.94 | 91.71 |
| RP298 | YEBAWYIN::IRGC 33885-1 | LL_N | DS | 74 | 115.45 | 23.19 | 26.30 | 50 | 12812.25 | 5431.92 | 0.43 | 237.94 | 96.48 |
| RP299 | YONG JIN ZAO 3::IRGC 70441-1 | LL_N | DS | 81 | 82.73 | 22.17 | 40.15 | 49 | 10385.02 | 4600.46 | 0.48 | 193.25 | 97.40 |
| RP300 | H 6::IRGC 157-1 | LL_N | DS | 96 | 141.12 | 24.33 | 38.34 | 61 | 28971.00 | 7867.20 | 0.40 | 246.87 | 97.77 |
| RP002 | 19::IRGC 70786-1 | UL_S | WS | 89 | 104.17 | 20.07 | 36.97 | 21 | 2104.00 | 958.63 | 0.08 | 177.16 | 41.42 |
| RP003 | 3210::IRGC 116950-1 | UL_S | WS | 84 | 102.26 | 19.68 | 36.73 | 21 | 3492.40 | 963.69 | 0.07 | 180.65 | 42.97 |
| RP004 | 498-2A BR 8::IRGC 5891-1 | UL_S | WS | 74 | 112.60 | 20.60 | 38.36 | 24 | 9273.60 | 1011.71 | 0.21 | 193.30 | 85.52 |
| RP005 | ADT 12::IRGC 6254-1 | UL_S | WS | 85 | 107.96 | 20.81 | 39.02 | 23 | 4004.80 | 978.40 | 0.12 | 183.66 | 49.54 |
| RP006 | AE NOUA::IRGC 89308-1 | UL_S | WS | 87 | 98.38 | 19.94 | 37.21 | 22 | 5298.40 | 961.40 | 0.03 | 190.66 | 80.42 |
| RP007 | ALTAMIRA 9::IRGC 116953-1 | UL_S | WS | 85 | 93.12 | 19.21 | 35.85 | 22 | 1282.00 | 955.40 | 0.07 | 186.66 | 56.10 |
| RP008 | ARAURE 1::IRGC 116956-1 | UL_S | WS | 0 | 0.00 | 0.00 | 0.00 | 0 | 0.00 | 0.00 | 0.00 | 0.00 | 0.00 |
| RP009 | ARC 10100::IRGC 20709-1 | UL_S | WS | 67 | 116.70 | 20.90 | 37.42 | 24 | 5124.00 | 993.05 | 0.12 | 189.09 | 75.77 |
| RP010 | ARC 10594::IRGC 12524-1 | UL_S | WS | 84 | 105.04 | 20.28 | 36.54 | 20 | NA | 958.77 | 0.00 | 188.57 | 28.60 |
| RP011 | ARC 10754::IRGC 12603-1 | UL_S | WS | 83 | 113.78 | 20.73 | 40.73 | 25 | 14614.80 | 1013.38 | 0.15 | 191.16 | 78.04 |
| RP012 | ARC 10812::IRGC 21074-1 | UL_S | WS | 78 | 115.39 | 21.02 | 39.28 | 23 | 6440.40 | 985.83 | 0.24 | 192.45 | 91.46 |
| RP013 | ARC 11524::IRGC 42672-1 | UL_S | WS | 84 | 106.69 | 20.22 | 37.88 | 21 | 1396.80 | 955.69 | 0.10 | 179.36 | 52.23 |
| RP014 | ARC 11857::IRGC 40972-1 | UL_S | WS | 72 | 112.87 | 20.47 | 39.61 | 23 | 3124.40 | 977.50 | 0.09 | 190.16 | 58.87 |
| RP015 | ARC 11901::IRGC 21727-1 | UL_S | WS | 0 | 0.00 | 0.00 | 0.00 | 0 | 0.00 | 0.00 | 0.00 | 0.00 | 0.00 |
| RP018 | ARC 13778::IRGC 41216-1 | UL_S | WS | 87 | 96.61 | 19.45 | 34.09 | 23 | 5790.00 | 932.29 | 0.09 | 178.70 | 55.45 |
| RP019 | ARC 14060::IRGC 41374-1 | UL_S | WS | 79 | 111.78 | 20.48 | 38.83 | 21 | 2425.20 | 958.26 | 0.08 | 190.55 | 41.32 |
| RP020 | ARC 14064::IRGC 41377-1 | UL_S | WS | 85 | 123.64 | 20.51 | 37.74 | 23 | 10490.00 | 954.09 | 0.11 | 192.41 | 83.16 |
| RP021 | ARC 14654::IRGC 41663-1 | UL_S | WS | 83 | 101.09 | 20.00 | 35.73 | 22 | 1483.20 | 946.78 | 0.12 | 191.93 | 72.73 |
| RP022 | ARC 15873::IRGC 43250-1 | UL_S | WS | 79 | 114.99 | 20.02 | 38.90 | 23 | 8768.80 | 956.11 | 0.12 | 187.69 | 68.43 |
| RP023 | ARC 18092::IRGC 42256-1 | UL_S | WS | 76 | 120.15 | 20.44 | 37.86 | 23 | 7931.60 | 1005.24 | 0.20 | 193.03 | 84.80 |
| **Study designation** | **3KG database designation** | **Env** | **Season** | **DTF** | **PH (cm)** | **PL (cm)** | **FlgLA (cm^2^)** | **NBP** | **BMDW (kgha^-1^)** | **GY (kgha^-1^)** | **HI** | **TGW (g)** | **SPKFT (%)** |
| RP024 | ARC 18112::IRGC 42274-1 | UL_S | WS | 0 | 0.00 | 0.00 | 0.00 | 0 | 0.00 | 0.00 | 0.00 | 0.00 | 0.00 |
| RP025 | ARC 18202::IRGC 42328-1 | UL_S | WS | 78 | 103.59 | 20.46 | 37.60 | 22 | 3246.40 | 962.77 | 0.03 | 191.87 | 51.66 |
| RP026 | ARC 18597::IRGC 43299-1 | UL_S | WS | 0 | 0.00 | 0.00 | 0.00 | 0 | 0.00 | 0.00 | 0.00 | 0.00 | 0.00 |
| RP028 | ASHI BINNI::IRGC 77216-1 | UL_S | WS | 71 | 116.26 | 20.98 | 40.05 | 23 | 5242.00 | 1003.60 | 0.00 | 194.48 | 82.68 |
| RP029 | ASHMBER::IRGC 27522-1 | UL_S | WS | 82 | 104.89 | 20.35 | 39.08 | 25 | 5740.80 | 974.83 | 0.10 | 187.77 | 73.82 |
| RP030 | ASU::IRGC 62154-1 | UL_S | WS | 81 | 115.53 | 20.84 | 39.36 | 21 | 3216.40 | 1001.62 | 0.13 | 190.75 | 42.09 |
| RP031 | AUS 171::IRGC 29004-1 | UL_S | WS | 73 | 113.71 | 20.70 | 37.28 | 24 | 5990.40 | 1043.75 | 0.18 | 182.02 | 54.63 |
| RP032 | AUS 177::IRGC 29009-1 | UL_S | WS | 72 | 107.54 | 19.97 | 36.59 | 21 | 1145.20 | 961.91 | 0.14 | 174.06 | 42.02 |
| RP033 | AUS 219::IRGC 29031-1 | UL_S | WS | 82 | 101.98 | 20.15 | 36.48 | 20 | 835.20 | 955.63 | 0.10 | 188.39 | 34.49 |
| RP034 | AUS 233::IRGC 29036-1 | UL_S | WS | 84 | 98.60 | 19.80 | 37.49 | 21 | 2339.20 | 923.87 | 0.06 | 185.82 | 60.42 |
| RP036 | AUS 295::IRGC 29083-1 | UL_S | WS | 80 | 111.91 | 20.21 | 37.99 | 25 | 5510.40 | 969.36 | 0.13 | 191.02 | 81.11 |
| RP037 | AUS 301::IRGC 29089-1 | UL_S | WS | 77 | 101.74 | 20.13 | 36.08 | 22 | 4599.20 | 948.53 | 0.19 | 190.58 | 82.13 |
| RP038 | AUS 308::IRGC 29096-1 | UL_S | WS | 74 | 112.48 | 19.98 | 36.76 | 21 | 2332.40 | 959.26 | 0.13 | 179.25 | 72.03 |
| RP039 | AUS 329::IRGC 29116-1 | UL_S | WS | 0 | 0.00 | 0.00 | 0.00 | 0 | 0.00 | 0.00 | 0.00 | 0.00 | 0.00 |
| RP040 | AUS 344::IRGC 29131-1 | UL_S | WS | 78 | 114.85 | 20.68 | 38.95 | 25 | 14687.20 | 994.16 | 0.13 | 195.13 | 66.78 |
| RP041 | AUS 439::IRGC 29221-1 | UL_S | WS | 81 | 117.00 | 20.67 | 37.26 | 21 | 3731.60 | 998.82 | 0.11 | 188.74 | 41.99 |
| RP042 | AUS PADDY (RED)::IRGC 44978-1 | UL_S | WS | 90 | 129.10 | 20.92 | 43.01 | 23 | 9858.80 | 968.25 | 0.07 | 198.58 | 64.79 |
| RP043 | B 4414 F-MR-6-3::IRGC 117310-1 | UL_S | WS | 88 | 109.34 | 20.18 | 38.02 | 22 | 2202.00 | 941.92 | 0.16 | 191.16 | 62.84 |
| RP044 | B 6136 E-3-TB-0-1-5::IRGC 117311-1 | UL_S | WS | 74 | 118.07 | 20.97 | 39.83 | 24 | 8506.40 | 1057.66 | 0.21 | 181.39 | 83.42 |
| RP045 | B 6136-3-TB-0-1-5::IRGC 117312-1 | UL_S | WS | 79 | 115.57 | 20.91 | 39.67 | 23 | 8280.40 | 970.61 | 0.17 | 194.45 | 77.31 |
| RP046 | B 6149 F-MR-7::IRGC 117314-1 | UL_S | WS | 0 | 0.00 | 0.00 | 0.00 | 0 | 0.00 | 0.00 | 0.00 | 0.00 | 0.00 |
| RP047 | BA BAI GU::IRGC 79580-1 | UL_S | WS | 76 | 116.88 | 20.54 | 37.29 | 21 | 3562.80 | 970.34 | 0.07 | 180.53 | 73.70 |
| RP048 | BA SHI ZAO::IRGC 67903-1 | UL_S | WS | 79 | 118.75 | 20.38 | 38.35 | 24 | 7257.20 | 1004.25 | 0.09 | 194.54 | 35.32 |
| RP049 | BADAL 1163::IRGC 32796-1 | UL_S | WS | 85 | 100.65 | 20.17 | 36.34 | 22 | 5151.60 | 941.26 | 0.13 | 186.57 | 74.92 |
| RP050 | BADUIE::IRGC 53715-1 | UL_S | WS | 79 | 117.43 | 21.15 | 41.96 | 23 | 8487.20 | 972.14 | 0.11 | 194.73 | 69.92 |
| RP051 | BAI HE::IRGC 76438-1 | UL_S | WS | 67 | 117.47 | 20.74 | 35.64 | 27 | 11139.20 | 1103.16 | 0.23 | 191.83 | 86.30 |
| RP052 | BAIANG 6::IRGC 6129-1 | UL_S | WS | 77 | 110.76 | 20.72 | 38.77 | 25 | 10125.60 | 1010.10 | 0.25 | 192.00 | 83.22 |
| RP053 | BAK TULSI::IRGC 34831-1 | UL_S | WS | 82 | 117.19 | 20.49 | 38.52 | 21 | NA | 0.00 | 0.00 | 191.11 | 51.52 |
| **Study designation** | **3KG database designation** | **Env** | **Season** | **DTF** | **PH (cm)** | **PL (cm)** | **FlgLA (cm^2^)** | **NBP** | **BMDW (kgha^-1^)** | **GY (kgha^-1^)** | **HI** | **TGW (g)** | **SPKFT (%)** |
| RP054 | BAKASI::IRGC 27074-1 | UL_S | WS | 84 | 99.12 | 19.90 | 36.20 | 20 | 936.80 | 0.00 | 0.00 | 167.46 | 13.64 |
| RP055 | BALASURIYA A::IRGC 66509-1 | UL_S | WS | 73 | 110.50 | 20.43 | 37.09 | 23 | 6529.60 | 991.39 | 0.11 | 178.70 | 40.63 |
| RP056 | BAMOA A 75::IRGC 51101-1 | UL_S | WS | 80 | 107.92 | 20.52 | 38.58 | 22 | 6806.80 | 949.50 | 0.11 | 192.47 | 72.06 |
| RP057 | BANDI::IRGC 17214-1 | UL_S | WS | NA | 100.92 | 20.08 | 36.27 | 20 | 300.80 | 949.20 | 0.15 | 174.57 | 37.93 |
| RP058 | BANGKOUY::IRGC 94037-1 | UL_S | WS | 75 | 114.68 | 20.14 | 36.39 | 25 | 9197.20 | 1007.60 | 0.22 | 192.39 | 82.08 |
| RP059 | BARIK KUDI::IRGC 52807-1 | UL_S | WS | 78 | 114.61 | 20.86 | 42.54 | 23 | 9448.40 | 968.11 | 0.11 | 194.03 | 65.81 |
| RP060 | BAT DO::IRGC 7014-1 | UL_S | WS | 85 | 103.43 | 20.16 | 36.95 | 21 | 2356.40 | 954.06 | 0.07 | 187.84 | 40.86 |
| RP061 | BATHURI::IRGC 25838-1 | UL_S | WS | 87 | 106.36 | 19.95 | 37.15 | 23 | NA | 971.97 | 0.00 | 185.29 | 59.66 |
| RP062 | BAZAIL::IRGC 27526-1 | UL_S | WS | 0 | 0.00 | 0.00 | 0.00 | 0 | 0.00 | 0.00 | 0.00 | 0.00 | 0.00 |
| RP063 | BENGALY MORIMO::IRGC 10976-1 | UL_S | WS | 96 | 117.73 | 20.49 | 36.80 | 19 | NA | 956.69 | 0.00 | 181.67 | 26.11 |
| RP065 | BHADOIA 303::IRGC 6588-1 | UL_S | WS | 78 | 112.88 | 20.42 | 37.74 | 20 | 2881.20 | 975.77 | 0.02 | 185.69 | 32.05 |
| RP066 | BK 26::IRGC 45197-1 | UL_S | WS | 83 | 119.74 | 20.67 | 38.76 | 21 | 7939.20 | 995.82 | 0.11 | 196.69 | 91.88 |
| RP067 | BKN BR 1031-78-5-4::IRGC 55927-1 | UL_S | WS | 85 | 116.49 | 20.68 | 38.94 | 21 | 3922.40 | 962.77 | 0.05 | 179.80 | 31.85 |
| RP068 | BONG SEN::IRGC 7011-1 | UL_S | WS | 0 | 0.00 | 0.00 | 0.00 | 0 | 0.00 | 0.00 | 0.00 | 0.00 | 0.00 |
| RP070 | BPI 76 NON SENSITIVE (GREEN)::IRGC 9790-1 | UL_S | WS | 80 | 115.36 | 20.66 | 40.59 | 23 | 7810.40 | 969.72 | 0.21 | 190.55 | 80.91 |
| RP071 | BR 51-115-4::IRGC 43999-1 | UL_S | WS | 78 | 97.58 | 19.65 | 35.31 | 22 | 3668.00 | 953.11 | 0.09 | 185.22 | 49.80 |
| RP072 | BR 5230-46-4::IRGC 117318-1 | UL_S | WS | 72 | 97.78 | 19.88 | 35.06 | 23 | 7488.00 | 940.01 | 0.13 | 189.13 | 67.62 |
| RP073 | BR IRGA 409::IRGC 55915-1 | UL_S | WS | 76 | 108.00 | 20.12 | 39.51 | 24 | 8953.60 | 970.94 | 0.19 | 193.73 | 79.19 |
| RP074 | BW 295-5::IRGC 63098-1 | UL_S | WS | 85 | 117.44 | 20.81 | 39.21 | 21 | 5946.00 | 957.63 | 0.03 | 189.46 | 29.34 |
| RP075 | C 1016-1::IRGC 50368-1 | UL_S | WS | 76 | 108.31 | 20.31 | 39.14 | 23 | 7836.00 | 978.30 | 0.13 | 195.86 | 72.58 |
| RP076 | C 166-135::IRGC 50633-1 | UL_S | WS | 72 | 117.06 | 20.34 | 37.40 | 22 | 6264.40 | 1008.52 | 0.22 | 193.26 | 88.34 |
| RP077 | C 662083::IRGC 62101-1 | UL_S | WS | 0 | 0.00 | 0.00 | 0.00 | 0 | 0.00 | 0.00 | 0.00 | 0.00 | 0.00 |
| RP079 | CAUVERY::IRGC 45255-1 | UL_S | WS | 96 | 96.63 | 19.70 | 35.87 | 21 | 2068.40 | 955.40 | 0.05 | 179.67 | 42.19 |
| RP080 | CE IN TSAN::IRGC 4362-1 | UL_S | WS | 85 | 128.76 | 21.15 | 44.65 | 22 | 9110.40 | 963.58 | 0.12 | 189.91 | 74.04 |
| RP081 | CEA 3::IRGC 116965-1 | UL_S | WS | 78 | 111.23 | 20.38 | 37.23 | 22 | 3468.40 | 968.54 | 0.00 | 186.76 | 76.21 |
| RP082 | CEMPO MANGGAR::IRGC 27107-1 | UL_S | WS | 0 | 0.00 | 0.00 | 0.00 | 0 | 0.00 | 0.00 | 0.00 | 0.00 | 0.00 |
| RP083 | CHAKOL::IRGC 77226-1 | UL_S | WS | 85 | 108.05 | 20.09 | 37.86 | 22 | 7534.40 | 944.09 | 0.09 | 190.88 | 76.93 |
| RP084 | CHAM LEK::IRGC 89387-1 | UL_S | WS | 88 | 105.09 | 20.05 | 37.81 | 22 | 1516.40 | 947.64 | 0.06 | 192.63 | 70.33 |
| **Study designation** | **3KG database designation** | **Env** | **Season** | **DTF** | **PH (cm)** | **PL (cm)** | **FlgLA (cm^2^)** | **NBP** | **BMDW (kgha^-1^)** | **GY (kgha^-1^)** | **HI** | **TGW (g)** | **SPKFT (%)** |
| RP085 | CHAMA (DWARF)::IRGC 69487-1 | UL_S | WS | 86 | 118.78 | 20.94 | 38.27 | 21 | 2069.20 | 958.26 | 0.07 | 184.41 | 48.04 |
| RP086 | CHANDARHAT::IRGC 25845-1 | UL_S | WS | NA | 111.46 | 20.19 | 37.06 | 19 | 2882.40 | 946.98 | NA | 178.63 | NA |
| RP087 | CHANDINA::IRGC 36420-1 | UL_S | WS | 0 | 0.00 | 0.00 | 0.00 | 0 | 0.00 | 0.00 | 0.00 | 0.00 | 0.00 |
| RP088 | CHANG LE SAN SHU ZAO::IRGC 63561-1 | UL_S | WS | 85 | 101.66 | 19.86 | 36.75 | 20 | 3696.00 | 953.77 | 0.08 | 177.80 | 37.66 |
| RP089 | CHAO PEUAK DENG::IRGC 11602-1 | UL_S | WS | 0 | 0.00 | 0.00 | 0.00 | 0 | 0.00 | 0.00 | 0.00 | 0.00 | 0.00 |
| RP090 | CHI SHENG TAO::IRGC 4606-1 | UL_S | WS | 82 | 95.95 | 20.00 | 37.74 | 22 | 1318.00 | 932.37 | 0.07 | 187.05 | 64.27 |
| RP091 | CHIAYI WU-K'O::IRGC 64974-1 | UL_S | WS | 89 | 121.71 | 20.89 | 40.41 | 20 | NA | 962.77 | 0.00 | 181.44 | 25.73 |
| RP092 | CHIH SHEN LI::IRGC 1306-1 | UL_S | WS | 91 | 97.65 | 19.84 | 36.19 | 21 | 5394.80 | 949.40 | 0.03 | 178.65 | 59.79 |
| RP093 | CHNNOR::IRGC 67485-1 | UL_S | WS | 76 | 118.07 | 20.61 | 38.71 | 25 | 16520.40 | 982.55 | 0.12 | 195.66 | 74.05 |
| RP094 | CHUA DAU::IRGC 4785-1 | UL_S | WS | 95 | 104.58 | 19.96 | 35.54 | 21 | NA | 953.83 | 0.00 | 191.55 | 50.14 |
| RP095 | CHUNGUR BALI::IRGC 25855-1 | UL_S | WS | 76 | 116.02 | 20.28 | 36.35 | 21 | 3983.20 | 987.19 | 0.06 | 178.91 | 35.43 |
| RP096 | CICA 9::IRGC 53079-1 | UL_S | WS | 81 | 104.87 | 20.49 | 37.01 | 21 | 2371.20 | 978.62 | 0.09 | 179.12 | 42.52 |
| RP097 | CN 44-40-7::IRGC 45368-1 | UL_S | WS | 75 | 102.25 | 19.93 | 36.87 | 24 | 7866.00 | 977.64 | 0.19 | 195.31 | 76.14 |
| RP098 | CO 39::IRGC 51231-1 | UL_S | WS | 87 | 101.89 | 19.76 | 35.81 | 22 | 5620.40 | 947.28 | 0.11 | 186.77 | 78.66 |
| RP099 | CR 60-10::IRGC 15777-1 | UL_S | WS | 82 | 109.98 | 20.29 | 38.69 | 22 | 4844.00 | 959.28 | 0.20 | 188.53 | 84.58 |
| RP100 | CRILLO LA FRIA::IRGC 10793-1 | UL_S | WS | 85 | 101.55 | 20.13 | 37.52 | 21 | 3252.00 | 0.00 | 0.01 | 178.60 | 48.08 |
| RP101 | CSR-90 IR-2::IRGC 117327-1 | UL_S | WS | 71 | 118.80 | 20.38 | 39.23 | 24 | 10558.40 | 1087.89 | 0.28 | 194.61 | 84.37 |
| RP102 | CT 9737-6-1-1-2-2P-M::IRGC 117330-1 | UL_S | WS | 71 | 116.35 | 20.53 | 35.71 | 26 | 10932.80 | 1043.04 | 0.33 | 190.50 | 92.54 |
| RP103 | CUN GU NUO::IRGC 63576-1 | UL_S | WS | 82 | 111.37 | 20.11 | 38.47 | 22 | 5096.40 | 936.17 | 0.09 | 193.83 | 74.80 |
| RP104 | CUYAMEL 3820::IRGC 116975-1 | UL_S | WS | 83 | 128.81 | 20.75 | 42.73 | 22 | 9806.00 | 1022.04 | 0.08 | 183.07 | 49.86 |
| RP105 | DA 11::IRGC 6046-1 | UL_S | WS | 85 | 108.79 | 20.28 | 37.10 | 22 | 3814.40 | 979.25 | 0.14 | 179.98 | 70.89 |
| RP106 | DA GANG ZHAN::IRGC 67103-1 | UL_S | WS | 77 | 110.50 | 20.50 | 36.65 | 22 | 6394.40 | 938.26 | 0.12 | 190.80 | 67.99 |
| RP107 | DA NUO (ZHAN)::IRGC 72025-1 | UL_S | WS | 85 | 118.10 | 20.73 | 40.80 | 21 | 5501.20 | 993.82 | 0.05 | 186.54 | 36.87 |
| RP108 | DAA MANSA::IRGC 67559-1 | UL_S | WS | 80 | 108.81 | 20.26 | 37.64 | 24 | 18862.00 | 1001.80 | 0.11 | 192.53 | 82.70 |
| RP109 | DANGAR::IRGC 76296-1 | UL_S | WS | 72 | 109.10 | 20.11 | 37.26 | 24 | 4788.80 | 980.47 | 0.21 | 192.22 | 86.70 |
| RP110 | DHANE BURWA::IRGC 10105-1 | UL_S | WS | 75 | 106.23 | 20.26 | 38.05 | 23 | 8250.40 | 935.81 | 0.12 | 188.63 | 67.30 |
| RP111 | DISSI::IRGC 101346-1 | UL_S | WS | 83 | 98.58 | 19.97 | 35.67 | 25 | 7583.20 | 969.03 | 0.21 | 190.02 | 84.14 |
| RP112 | DUDH KADAR::IRGC 67707-1 | UL_S | WS | 0 | 0.00 | 0.00 | 0.00 | 0 | 0.00 | 0.00 | 0.00 | 0.00 | 0.00 |
| **Study designation** | **3KG database designation** | **Env** | **Season** | **DTF** | **PH (cm)** | **PL (cm)** | **FlgLA (cm^2^)** | **NBP** | **BMDW (kgha^-1^)** | **GY (kgha^-1^)** | **HI** | **TGW (g)** | **SPKFT (%)** |
| RP113 | E 2024::IRGC 67958-1 | UL_S | WS | 0 | 0.00 | 0.00 | 0.00 | 0 | 0.00 | 0.00 | 0.00 | 0.00 | 0.00 |
| RP114 | E 2040::IRGC 67968-1 | UL_S | WS | 83 | 101.71 | 20.04 | 36.75 | 21 | 3112.80 | 965.26 | 0.05 | 188.62 | 34.00 |
| RP115 | E DAW HAWM::IRGC 47938-1 | UL_S | WS | 83 | 114.69 | 20.35 | 37.56 | 22 | 5471.60 | 971.48 | 0.06 | 176.25 | 38.51 |
| RP116 | ELONI::IRGC 116980-1 | UL_S | WS | 90 | 93.91 | 19.37 | 35.66 | 21 | NA | 0.00 | 0.00 | 165.47 | 24.17 |
| RP117 | EPEAL 102::IRGC 78698-1 | UL_S | WS | 85 | 112.72 | 20.07 | 38.34 | 22 | 4132.80 | 954.49 | 0.02 | 188.41 | 41.25 |
| RP118 | ES 21::IRGC 56171-1 | UL_S | WS | 81 | 103.10 | 20.24 | 36.12 | 21 | 1386.80 | 955.40 | 0.09 | 192.10 | 60.42 |
| RP119 | EX FOILAEIN (NAPUTO)::IRGC 81675-1 | UL_S | WS | 76 | 109.87 | 20.30 | 38.41 | 25 | 10540.00 | 1075.31 | 0.31 | 191.78 | 91.64 |
| RP120 | FEI GAI 122::IRGC 63599-1 | UL_S | WS | 83 | 105.22 | 19.91 | 36.63 | 21 | 2762.80 | 966.48 | 0.08 | 178.52 | 44.84 |
| RP121 | FONAIAP 2::IRGC 116985-1 | UL_S | WS | 92 | 102.08 | 19.89 | 39.45 | 23 | 8425.60 | 939.28 | 0.11 | 192.57 | 60.11 |
| RP122 | FU ZAO XIAN::IRGC 63619-1 | UL_S | WS | 83 | 101.69 | 20.08 | 38.91 | 23 | 6809.20 | 954.28 | 0.14 | 192.32 | 75.04 |
| RP123 | G 25::IRGC 45733-1 | UL_S | WS | 69 | 116.37 | 20.55 | 37.88 | 22 | 6983.60 | 1021.90 | 0.15 | 182.74 | 47.70 |
| RP124 | GAO JIAO BAI::IRGC 68047-1 | UL_S | WS | 70 | 115.77 | 20.15 | 37.53 | 21 | 3785.60 | 951.55 | 0.01 | 177.75 | 9.79 |
| RP125 | GENIT::IRGC 3272-1 | UL_S | WS | 72 | 111.35 | 20.47 | 37.89 | 24 | 9875.20 | 1056.56 | 0.35 | 191.15 | 88.74 |
| RP126 | GOJOL GORIA::IRGC 26629-1 | UL_S | WS | 0 | 0.00 | 0.00 | 0.00 | 0 | 0.00 | 0.00 | 0.00 | 0.00 | 0.00 |
| RP127 | GOKULGANJA::IRGC 45701-1 | UL_S | WS | 74 | 113.20 | 20.50 | 37.84 | 22 | 4193.60 | 984.11 | 0.06 | 179.80 | 39.00 |
| RP128 | GUI HUA ZAO::IRGC 68060-1 | UL_S | WS | 83 | 122.58 | 20.91 | 38.17 | 22 | 7990.40 | 975.40 | 0.07 | 191.45 | 41.22 |
| RP130 | HAWM KRUA::IRGC 64333-1 | UL_S | WS | 70 | 117.11 | 20.02 | 38.95 | 24 | 8450.40 | 1077.88 | 0.15 | 190.42 | 79.34 |
| RP131 | HD 10::IRGC 6638-1 | UL_S | WS | 81 | 112.43 | 20.25 | 36.98 | 24 | 8994.00 | 974.77 | 0.16 | 195.41 | 74.02 |
| RP132 | HE GU TSAO::IRGC 51302-1 | UL_S | WS | 81 | 110.84 | 20.53 | 37.63 | 22 | 4252.00 | 991.82 | 0.11 | 181.28 | 76.09 |
| RP133 | HODARAWALA::IRGC 67631-1 | UL_S | WS | 0 | 0.00 | 0.00 | 0.00 | 0 | 0.00 | 0.00 | 0.00 | 0.00 | 0.00 |
| RP134 | HOLOI BASH (SOLOI BASH)::IRGC 64778-1 | UL_S | WS | 80 | 114.60 | 20.33 | 36.46 | 23 | 8767.60 | 975.25 | 0.00 | 194.71 | 77.40 |
| RP135 | HTA 22::IRGC 45827-1 | UL_S | WS | 84 | 108.54 | 20.17 | 36.46 | 20 | 3848.00 | 948.06 | 0.01 | 167.10 | 30.77 |
| RP136 | HUA LI ZAO::IRGC 80950-1 | UL_S | WS | 83 | 106.86 | 20.44 | 37.16 | 22 | 3429.60 | 987.34 | 0.11 | 195.16 | 40.60 |
| RP137 | I KUNG PAO::IRGC 114-1 | UL_S | WS | 81 | 118.49 | 20.82 | 38.63 | 22 | 3819.60 | 970.97 | 0.09 | 179.93 | 45.63 |
| RP138 | IA CUBA 17::IRGC 116990-1 | UL_S | WS | 85 | 110.23 | 20.27 | 37.19 | 21 | 4784.40 | 967.63 | 0.07 | 179.25 | 41.19 |
| RP139 | ICTA CRISPO 38::IRGC 116994-1 | UL_S | WS | 83 | 121.72 | 20.75 | 38.81 | 20 | 2695.20 | 955.92 | 0.02 | 188.46 | 21.78 |
| RP140 | ICTA MOTAGUA::IRGC 116995-1 | UL_S | WS | 82 | 119.07 | 20.77 | 40.38 | 24 | 8470.80 | 964.58 | 0.12 | 191.27 | 77.75 |
| RP141 | INIAP 6::IRGC 117002-1 | UL_S | WS | 85 | 107.98 | 19.99 | 37.75 | 21 | 3329.60 | 935.20 | 0.08 | 193.78 | 66.97 |
| **Study designation** | **3KG database designation** | **Env** | **Season** | **DTF** | **PH (cm)** | **PL (cm)** | **FlgLA (cm^2^)** | **NBP** | **BMDW (kgha^-1^)** | **GY (kgha^-1^)** | **HI** | **TGW (g)** | **SPKFT (%)** |
| RP142 | IR 13429-109-2-2-1::IRGC 63491-1 | UL_S | WS | 64 | 108.03 | 20.43 | 37.05 | 22 | 2951.60 | 975.83 | 0.13 | 191.30 | 42.43 |
| RP143 | IR 21015-72-3-3-3-1::IRGC 117004-1 | UL_S | WS | 79 | 98.26 | 20.17 | 37.69 | 23 | 2093.20 | 939.70 | 0.06 | 191.39 | 64.53 |
| RP144 | IR 2344-P1 PB-9-3-2B::IRGC 39317-C1 | UL_S | WS | 84 | 105.87 | 19.86 | 36.44 | 22 | 5561.60 | 949.70 | 0.16 | 191.27 | 80.36 |
| RP146 | IR 63295-AC 209-7::IRGC 117365-1 | UL_S | WS | 73 | 106.97 | 19.86 | 38.70 | 24 | 8844.40 | 999.49 | 0.20 | 183.17 | 58.60 |
| RP147 | IR 69502-6-SRN-3-UBN-1-B::IRGC 117290-1 | UL_S | WS | 75 | 111.16 | 20.93 | 37.82 | 24 | 8850.00 | 1022.40 | 0.23 | 192.44 | 85.15 |
| RP148 | IR 75870-5-8-5-B-1::IRGC 117297-1 | UL_S | WS | 80 | 112.05 | 20.38 | 36.70 | 26 | 11082.80 | 981.11 | 0.12 | 192.05 | 70.00 |
| RP149 | IR 77390-1-6-4-19-1-B::IRGC 117303-1 | UL_S | WS | 78 | 110.42 | 20.19 | 38.14 | 23 | 4376.00 | 974.25 | 0.14 | 179.13 | 47.67 |
| RP150 | IR 80310-12-B-1-3-B::IRGC 117307-1 | UL_S | WS | 79 | 114.81 | 19.95 | 37.97 | 25 | 8006.40 | 972.75 | 0.37 | 192.32 | 74.15 |
| RP151 | IR 80340-23-B-12-6-B::IRGC 117309-1 | UL_S | WS | 80 | 111.07 | 20.65 | 39.56 | 21 | 4027.60 | 975.34 | 0.12 | 158.30 | 61.55 |
| RP152 | IRGA 318-11-6-9-2B::IRGC 117339-1 | UL_S | WS | 73 | 113.57 | 20.93 | 38.25 | 22 | 5587.60 | 1035.04 | 0.12 | 179.76 | 41.67 |
| RP153 | IRGA 370-38-1-1F-C4-2::IRGC 117342-1 | UL_S | WS | 81 | 117.65 | 20.25 | 37.45 | 21 | 6180.80 | 975.11 | 0.11 | 184.17 | 80.47 |
| RP154 | IRGA 370-42-1-1F-C-1::IRGC 117343-1 | UL_S | WS | 84 | 106.72 | 19.82 | 36.71 | 22 | 8364.00 | 955.47 | 0.00 | 185.80 | 78.91 |
| RP155 | IRGA 659-1-2-2-2::IRGC 117345-1 | UL_S | WS | 73 | 120.30 | 20.99 | 38.22 | 23 | 15968.40 | 1073.66 | 0.12 | 181.18 | 45.30 |
| RP156 | IRGA 959-1-2-2F-4-1-4A-6-CA-6X::IRGC 117006-1 | UL_S | WS | 72 | 117.29 | 20.51 | 37.90 | 23 | 4991.20 | 1001.77 | 0.00 | 191.09 | 69.28 |
| RP157 | IRI 339::IRGC 46956-1 | UL_S | WS | 71 | 110.40 | 20.37 | 39.02 | 23 | 6518.80 | 1051.75 | 0.18 | 186.54 | 79.37 |
| RP158 | IRRIBINI::IRGC 49094-1 | UL_S | WS | 80 | 121.58 | 20.52 | 41.21 | 21 | 8261.60 | 956.03 | 0.07 | 197.19 | 49.72 |
| RP159 | JABOR SAIL::IRGC 66831-1 | UL_S | WS | 86 | 103.30 | 19.94 | 37.05 | 21 | 4189.20 | 943.95 | 0.03 | 183.95 | 71.21 |
| RP160 | JAGLI BORO::IRGC 27516-2 | UL_S | WS | 86 | 118.04 | 20.53 | 37.00 | 22 | 7355.20 | 953.81 | 0.13 | 191.62 | 84.92 |
| RP161 | JAMBALI::IRGC 73101-1 | UL_S | WS | 87 | 119.01 | 21.00 | 38.52 | 22 | NA | 1016.47 | 0.00 | 192.39 | 47.49 |
| RP162 | JAO LEUANG::IRGC 65866-1 | UL_S | WS | 0 | 0.00 | 0.00 | 0.00 | 0 | 0.00 | 0.00 | 0.00 | 0.00 | 0.00 |
| RP164 | JHODI BIRUN::IRGC 31812-1 | UL_S | WS | 83 | 103.76 | 19.93 | 36.35 | 22 | 2688.00 | 955.77 | 0.06 | 189.20 | 64.77 |
| RP165 | JIN JUN DAO::IRGC 59710-1 | UL_S | WS | 0 | 0.00 | 0.00 | 0.00 | 0 | 0.00 | 0.00 | 0.00 | 0.00 | 0.00 |
| RP166 | JUMA 62::IRGC 117011-1 | UL_S | WS | 83 | 112.46 | 20.42 | 36.91 | 22 | 4880.80 | 1022.33 | 0.10 | 192.47 | 61.37 |
| RP167 | KALABAIL::IRGC 25877-1 | UL_S | WS | 82 | 119.73 | 20.59 | 38.93 | 23 | 7418.80 | 949.56 | 0.17 | 191.87 | 84.21 |
| RP168 | KALIA::IRGC 34699-1 | UL_S | WS | 76 | 108.81 | 20.34 | 36.82 | 23 | 2547.60 | 945.28 | 0.10 | 190.91 | 86.93 |
| RP169 | KALO CHAKOL::IRGC 77258-1 | UL_S | WS | 71 | 100.00 | 20.02 | 36.55 | 22 | 5009.60 | 942.70 | 0.00 | 187.25 | 69.67 |
| RP170 | KALU ILANKALAYAN::IRGC 36270-1 | UL_S | WS | 79 | 113.31 | 20.36 | 39.92 | 22 | 5354.00 | 957.97 | 0.03 | 188.87 | 38.31 |
| **Study designation** | **3KG database designation** | **Env** | **Season** | **DTF** | **PH (cm)** | **PL (cm)** | **FlgLA (cm^2^)** | **NBP** | **BMDW (kgha^-1^)** | **GY (kgha^-1^)** | **HI** | **TGW (g)** | **SPKFT (%)** |
| RP171 | KAM PAI::IRGC 78245-1 | UL_S | WS | 83 | 116.15 | 20.36 | 38.07 | 22 | 4813.20 | 980.25 | 0.07 | 180.61 | 39.96 |
| RP172 | KEERIPALA CHILL PADDY::IRGC 49790-1 | UL_S | WS | 85 | 104.83 | 19.94 | 36.34 | 21 | NA | 972.63 | 0.00 | 191.26 | 39.52 |
| RP173 | KETAN SERANG::IRGC 14615-1 | UL_S | WS | 94 | 112.11 | 21.03 | 40.50 | 22 | 4175.60 | 963.81 | 0.09 | 192.07 | 70.18 |
| RP174 | KHAO DAWK MALI 105::IRGC 27748-2 | UL_S | WS | 83 | 105.88 | 20.17 | 36.55 | 22 | 3132.00 | 970.20 | 0.08 | 183.18 | 40.63 |
| RP175 | KHAO GRADOOK CHAHNG::IRGC 17111-1 | UL_S | WS | 75 | 111.46 | 20.75 | 39.23 | 24 | 9924.00 | 1022.40 | 0.20 | 196.36 | 85.58 |
| RP176 | KHAO' HAWM::IRGC 78257-1 | UL_S | WS | 0 | 0.00 | 0.00 | 0.00 | 0 | 0.00 | 0.00 | 0.00 | 0.00 | 0.00 |
| RP177 | KHAO THI RATE::IRGC 58041-1 | UL_S | WS | 84 | 112.90 | 20.87 | 40.38 | 25 | 5025.60 | 962.50 | 0.10 | 196.28 | 66.80 |
| RP178 | KHARSU 80::IRGC 28016-1 | UL_S | WS | 87 | 100.48 | 20.19 | 36.07 | 23 | 26311.20 | 936.51 | 0.04 | 196.05 | 66.78 |
| RP179 | KITRANA 1007::IRGC 68517-1 | UL_S | WS | 91 | 97.58 | 19.50 | 35.00 | 20 | 788.40 | 949.63 | 0.04 | 184.96 | 67.55 |
| RP180 | KN 1 B 361-1-8-6-9::IRGC 46974-1 | UL_S | WS | 82 | 125.46 | 20.36 | 39.92 | 22 | 5893.60 | 942.98 | 0.12 | 187.93 | 72.09 |
| RP181 | KORASISI::IRGC 5285-1 | UL_S | WS | 94 | 97.73 | 20.50 | 36.17 | 22 | 2115.60 | 955.83 | 0.06 | 184.15 | 45.93 |
| RP182 | KOTTEYARAN::IRGC 47383-1 | UL_S | WS | 69 | 101.14 | 20.38 | 38.85 | 22 | 2356.40 | 938.59 | 0.05 | 193.20 | 60.02 |
| RP183 | KOYRA::IRGC 77267-1 | UL_S | WS | 87 | 118.41 | 20.90 | 39.71 | 21 | 6422.80 | 975.68 | 0.01 | 189.85 | 21.43 |
| RP184 | KULA KARUPPAN::IRGC 55328-1 | UL_S | WS | 71 | 119.80 | 20.38 | 40.06 | 25 | 7729.60 | 1063.72 | 0.15 | 193.44 | 86.12 |
| RP185 | KUNENG::IRGC 71545-1 | UL_S | WS | 70 | 107.14 | 19.91 | 36.26 | 26 | 10383.20 | 1023.98 | 0.22 | 192.20 | 84.87 |
| RP186 | KURULU WEE (WHITE)::IRGC 66518-1 | UL_S | WS | 80 | 121.15 | 21.29 | 42.63 | 23 | 9146.80 | 965.11 | 0.07 | 192.04 | 61.22 |
| RP188 | KUSHIARA::IRGC 34709-1 | UL_S | WS | 83 | 104.43 | 20.24 | 36.86 | 22 | 4348.80 | 968.68 | 0.06 | 191.68 | 43.38 |
| RP189 | KUTTA::IRGC 52184-1 | UL_S | WS | 91 | 110.66 | 20.29 | 38.15 | 22 | 5326.00 | 975.25 | 0.13 | 185.76 | 45.11 |
| RP190 | LABRA::IRGC 74757-1 | UL_S | WS | 94 | 122.09 | 21.42 | 38.22 | 23 | NA | 966.86 | 0.00 | 196.54 | 70.01 |
| RP191 | LAI YIP ZIM::IRGC 4955-1 | UL_S | WS | 82 | 115.05 | 20.37 | 41.84 | 22 | 4498.80 | 935.70 | 0.12 | 181.21 | 52.72 |
| RP192 | LALKA (LAL DHAN)::IRGC 64946-1 | UL_S | WS | 75 | 119.23 | 20.57 | 42.06 | 22 | 4624.80 | 1045.59 | 0.13 | 192.50 | 79.33 |
| RP193 | LALSAITA::IRGC 43915-1 | UL_S | WS | 75 | 100.09 | 20.54 | 37.75 | 22 | 2151.60 | 964.69 | 0.12 | 188.06 | 73.77 |
| RP194 | LARHA MUGAD::IRGC 52339-1 | UL_S | WS | 71 | 115.84 | 20.69 | 37.57 | 22 | 5399.60 | 1007.47 | 0.17 | 179.41 | 44.35 |
| RP196 | LENJA MURALI::IRGC 66815-1 | UL_S | WS | 87 | 118.32 | 20.36 | 34.12 | 23 | 5267.20 | 938.76 | 0.12 | 192.67 | 71.06 |
| RP197 | LEUANG YAI 29-12-2::IRGC 881-1 | UL_S | WS | 79 | 101.90 | 20.59 | 37.40 | 25 | 7513.60 | 991.44 | 0.23 | 197.82 | 86.97 |
| RP198 | LU MAO ZHAN::IRGC 68159-1 | UL_S | WS | 80 | 100.33 | 20.16 | 37.50 | 21 | 1259.60 | 956.83 | 0.07 | 177.18 | 37.50 |
| RP199 | LUA CHAN HUONG::IRGC 16800-1 | UL_S | WS | 74 | 114.96 | 20.43 | 37.80 | 23 | 4854.00 | 987.62 | 0.09 | 176.78 | 42.86 |
| RP200 | MAKALIOKA::IRGC 376-1 | UL_S | WS | 83 | 106.13 | 20.40 | 37.70 | 21 | 3776.00 | 967.68 | 0.08 | 191.41 | 80.37 |
| **Study designation** | **3KG database designation** | **Env** | **Season** | **DTF** | **PH (cm)** | **PL (cm)** | **FlgLA (cm^2^)** | **NBP** | **BMDW (kgha^-1^)** | **GY (kgha^-1^)** | **HI** | **TGW (g)** | **SPKFT (%)** |
| RP201 | MAKRO::IRGC 74763-1 | UL_S | WS | 80 | 105.84 | 20.55 | 36.46 | 24 | 4820.00 | 968.72 | 0.06 | 193.18 | 62.31 |
| RP202 | MAMORIAKA::IRGC 68672-1 | UL_S | WS | 78 | 105.59 | 20.37 | 37.62 | 22 | 2568.00 | 963.91 | 0.08 | 178.63 | 38.08 |
| RP203 | MEI FENG 9::IRGC 63735-1 | UL_S | WS | 0 | 0.00 | 0.00 | 0.00 | 0 | 0.00 | 0.00 | 0.00 | 0.00 | 0.00 |
| RP204 | MEKENZIE SMALL::IRGC 49895-1 | UL_S | WS | 67 | 106.20 | 20.16 | 36.60 | 24 | 4785.60 | 1004.76 | 0.19 | 188.83 | 42.87 |
| RP205 | MELEKE::IRGC 56823-1 | UL_S | WS | 78 | 99.09 | 20.08 | 36.82 | 23 | 4088.80 | 962.69 | 0.18 | 191.89 | 72.96 |
| RP206 | MENTIK TJERE BELUT::IRGC 18254-1 | UL_S | WS | 78 | 110.39 | 20.73 | 37.49 | 23 | 4419.20 | 1020.04 | 0.15 | 181.80 | 58.47 |
| RP207 | MILYANG 30::IRGC 46977-1 | UL_S | WS | 78 | 116.34 | 20.62 | 36.44 | 21 | 2046.80 | 951.81 | 0.07 | 188.11 | 79.13 |
| RP208 | MILYANG 77::IRGC 69340-1 | UL_S | WS | 78 | 114.84 | 20.21 | 37.83 | 25 | 11964.00 | 1016.57 | 0.20 | 193.30 | 87.98 |
| RP209 | MIN KE ZHAN::IRGC 72230-1 | UL_S | WS | 72 | 110.07 | 20.69 | 41.02 | 23 | 4638.00 | 986.77 | 0.11 | 193.03 | 71.32 |
| RP210 | MIN ZAO 6::IRGC 63772-1 | UL_S | WS | 78 | 105.79 | 20.49 | 37.02 | 22 | 5212.40 | 935.48 | 0.09 | 186.81 | 93.21 |
| RP211 | MODDAI KARUPPAN::IRGC 15465-1 | UL_S | WS | 71 | 114.25 | 20.90 | 38.30 | 24 | 11516.80 | 982.27 | 0.14 | 193.29 | 73.88 |
| RP212 | MUKKALA BAZAL::IRGC 77279-1 | UL_S | WS | 76 | 97.70 | 19.99 | 36.81 | 22 | 3909.60 | 940.20 | 0.11 | 193.74 | 77.63 |
| RP213 | MULLIKURUVA::IRGC 77529-1 | UL_S | WS | 82 | 104.79 | 19.68 | 36.35 | 22 | 4096.00 | 946.03 | 0.19 | 198.62 | 85.99 |
| RP214 | MUTA GANJE::IRGC 26744-1 | UL_S | WS | 78 | 109.46 | 20.61 | 38.01 | 21 | 2838.40 | 956.77 | 0.08 | 181.52 | 40.30 |
| RP215 | MUTTU SAMBA::IRGC 36333-1 | UL_S | WS | 78 | 104.23 | 20.28 | 37.09 | 21 | 1971.60 | 955.06 | 0.00 | 191.42 | 34.35 |
| RP216 | N 22::IRGC 46459-1 | UL_S | WS | 78 | 112.87 | 20.85 | 36.20 | 22 | 8726.40 | 948.53 | 0.12 | 178.57 | 63.19 |
| RP217 | Na souan::IRGC 11889-1 | UL_S | WS | 82 | 100.95 | 19.96 | 38.22 | 22 | 2855.20 | 942.78 | 0.18 | 182.81 | 71.29 |
| RP218 | NAZIRA SAIL::IRGC 77284-1 | UL_S | WS | 0 | 0.00 | 0.00 | 0.00 | 0 | 0.00 | 0.00 | 0.00 | 0.00 | 0.00 |
| RP219 | NCS 194::IRGC 51932-1 | UL_S | WS | 74 | 115.30 | 20.52 | 38.71 | 22 | 7715.20 | 1006.33 | 0.11 | 192.50 | 63.57 |
| RP220 | NCS 237::IRGC 62202-1 | UL_S | WS | 78 | 121.33 | 20.85 | 39.75 | 25 | 11743.20 | 1068.22 | 0.32 | 197.33 | 94.61 |
| RP222 | NCS 964 C::IRGC 62604-1 | UL_S | WS | NA | 98.89 | 19.57 | 36.06 | 20 | 1076.40 | 947.35 | 0.01 | 173.74 | 55.00 |
| RP223 | NIAO YAO::IRGC 5496-1 | UL_S | WS | 80 | 100.70 | 19.96 | 37.00 | 21 | 2665.60 | 955.49 | 0.05 | 177.15 | 34.09 |
| RP224 | NIBARI::IRGC 67742-1 | UL_S | WS | 88 | 118.64 | 21.02 | 39.40 | 22 | 10132.00 | 939.64 | 0.07 | 189.82 | 62.30 |
| RP225 | NONA BOKRA::IRGC 22710-C1 | UL_S | WS | 83 | 104.11 | 19.97 | 37.62 | 21 | NA | 952.97 | 0.00 | 177.83 | 29.25 |
| RP226 | NOROI::IRGC 31611-1 | UL_S | WS | 78 | 105.54 | 20.17 | 37.03 | 20 | 5662.40 | 960.26 | 0.18 | 182.44 | 21.21 |
| RP228 | NX 3533::IRGC 63796-1 | UL_S | WS | 72 | 115.95 | 20.46 | 39.30 | 25 | 12552.40 | 1001.57 | 0.20 | 194.55 | 88.02 |
| RP229 | O. SATIVA::IRGC 17083-1 | UL_S | WS | 82 | 104.72 | 20.68 | 37.16 | 24 | 9692.00 | 1040.79 | 0.19 | 195.76 | 83.95 |
| RP230 | PAH WEAN::IRGC 78276-1 | UL_S | WS | 85 | 116.35 | 20.85 | 38.12 | 22 | 2483.60 | 944.92 | 0.09 | 190.68 | 82.52 |
| **Study designation** | **3KG database designation** | **Env** | **Season** | **DTF** | **PH (cm)** | **PL (cm)** | **FlgLA (cm^2^)** | **NBP** | **BMDW (kgha^-1^)** | **GY (kgha^-1^)** | **HI** | **TGW (g)** | **SPKFT (%)** |
| RP231 | PAI CHUEH CHIU LIU::IRGC 34259-1 | UL_S | WS | 84 | 112.82 | 20.41 | 37.47 | 22 | 3647.60 | 964.64 | 0.05 | 193.97 | 59.25 |
| RP232 | PAI YI PING::IRGC 1368-1 | UL_S | WS | 77 | 119.63 | 20.90 | 39.91 | 23 | 3595.60 | 964.97 | 0.11 | 192.42 | 61.17 |
| RP233 | PALEPYU::IRGC 33549-1 | UL_S | WS | 84 | 105.24 | 20.17 | 37.12 | 22 | 5295.60 | 970.91 | 0.08 | 180.44 | 55.17 |
| RP234 | PARA NELLU::IRGC 50009-1 | UL_S | WS | 76 | 106.94 | 20.12 | 35.51 | 20 | NA | 0.00 | 0.00 | 174.86 | 15.22 |
| RP235 | PATISAIL::IRGC 37562-1 | UL_S | WS | 85 | 109.98 | 21.48 | 40.39 | 23 | 11695.60 | 1028.37 | 0.23 | 191.69 | 85.98 |
| RP237 | PERUNEL::IRGC 63113-1 | UL_S | WS | 84 | 120.84 | 20.57 | 38.96 | 20 | 4682.00 | 969.77 | 0.03 | 184.53 | 35.80 |
| RP238 | PICO NEGRO::IRGC 55849-1 | UL_S | WS | 91 | 97.64 | 19.70 | 36.75 | 22 | 1958.80 | 935.90 | 0.05 | 191.67 | 70.31 |
| RP239 | PLI KHAO::IRGC 64596-1 | UL_S | WS | 79 | 111.22 | 20.92 | 38.49 | 22 | 5395.60 | 955.25 | 0.22 | 195.15 | 85.06 |
| RP240 | PODI HEENATI::IRGC 36345-1 | UL_S | WS | 83 | 106.09 | 20.25 | 39.54 | 21 | 2978.00 | 957.20 | 0.08 | 192.62 | 58.06 |
| RP241 | PSBRC 50::IRGC 99706-1 | UL_S | WS | 84 | 102.12 | 19.97 | 37.36 | 21 | 4265.20 | 940.20 | 0.06 | 187.18 | 70.07 |
| RP242 | PSBRC 68::IRGC 99711-1 | UL_S | WS | 74 | 111.15 | 20.29 | 38.42 | 21 | 3330.00 | 985.11 | 0.17 | 190.90 | 45.46 |
| RP243 | PSBRC 88::IRGC 99717-1 | UL_S | WS | 0 | 0.00 | 0.00 | 0.00 | 0 | 0.00 | 0.00 | 0.00 | 0.00 | 0.00 |
| RP244 | PULUT BARAYA::IRGC 27393-1 | UL_S | WS | 78 | 108.74 | 20.55 | 37.96 | 24 | 6566.40 | 969.00 | 0.13 | 190.46 | 72.25 |
| RP245 | PURA BINNI::IRGC 26772-1 | UL_S | WS | 76 | 111.78 | 20.40 | 38.33 | 24 | 18233.20 | 1018.47 | 0.06 | 192.89 | 61.84 |
| RP247 | QUERO ASSAN::IRGC 28860-1 | UL_S | WS | 91 | 113.48 | 20.62 | 37.23 | 21 | NA | 956.54 | 0.00 | 189.74 | 31.04 |
| RP248 | RACE PERUMAL::IRGC 55347-1 | UL_S | WS | 74 | 124.12 | 20.62 | 37.73 | 24 | 10020.00 | 1006.35 | 0.19 | 198.00 | 77.22 |
| RP250 | RELLY::IRGC 14623-1 | UL_S | WS | 84 | 117.62 | 21.30 | 39.43 | 22 | 4482.40 | 967.33 | 0.06 | 191.15 | 63.69 |
| RP251 | RIZ TYPE SORGHO::IRGC 69015-1 | UL_S | WS | 83 | 103.30 | 20.17 | 37.62 | 24 | 9219.60 | 961.78 | 0.15 | 189.77 | 74.74 |
| RP252 | ROJOFOTSY::IRGC 69402-1 | UL_S | WS | 80 | 114.40 | 20.61 | 39.25 | 21 | 3456.40 | 970.63 | 0.09 | 181.27 | 74.12 |
| RP253 | RPA 5929 (K 45)::IRGC 33963-1 | UL_S | WS | 84 | 126.48 | 20.64 | 38.54 | 22 | 7197.60 | 971.25 | 0.05 | 191.07 | 73.97 |
| RP254 | RPW 9-4 (SS 1)::IRGC 50690-1 | UL_S | WS | 0 | 0.00 | 0.00 | 0.00 | 0 | 0.00 | 0.00 | 0.00 | 0.00 | 0.00 |
| RP255 | RR 272-17-829::IRGC 117354-1 | UL_S | WS | 80 | 121.71 | 20.29 | 39.10 | 22 | 6346.80 | 968.25 | 0.20 | 191.01 | 80.91 |
| RP256 | RTS 16::IRGC 8235-1 | UL_S | WS | 71 | 114.21 | 20.03 | 39.11 | 23 | 8219.20 | 1013.79 | 0.22 | 194.11 | 88.69 |
| RP257 | RUSTIC::IRGC 117026-1 | UL_S | WS | 73 | 115.09 | 20.46 | 37.19 | 22 | 6462.00 | 951.17 | 0.15 | 192.34 | 66.51 |
| RP258 | SADA RUPA::IRGC 77299-1 | UL_S | WS | 85 | 99.00 | 19.99 | 37.36 | 22 | 1797.20 | 953.69 | 0.05 | 186.52 | 49.11 |
| RP259 | SAN CHIAO TSWEN::IRGC 1565-1 | UL_S | WS | 85 | 99.87 | 19.67 | 36.20 | 23 | 4678.40 | 944.50 | 0.09 | 193.04 | 63.71 |
| RP260 | SAN SHIH TSI::IRGC 1038-1 | UL_S | WS | 90 | 102.44 | 19.77 | 35.51 | 20 | 1905.20 | 948.63 | 0.01 | 175.33 | 31.34 |
| RP261 | SIGARDIS::IRGC 15555-1 | UL_S | WS | 0 | 0.00 | 0.00 | 0.00 | 0 | 0.00 | 0.00 | 0.00 | 0.00 | 0.00 |
| **Study designation** | **3KG database designation** | **Env** | **Season** | **DTF** | **PH (cm)** | **PL (cm)** | **FlgLA (cm^2^)** | **NBP** | **BMDW (kgha^-1^)** | **GY (kgha^-1^)** | **HI** | **TGW (g)** | **SPKFT (%)** |
| RP262 | SIMET 2::IRGC 25734-1 | UL_S | WS | 77 | 110.89 | 20.60 | 38.37 | 21 | 3943.60 | 1003.82 | 0.12 | 190.73 | 42.76 |
| RP263 | SIMUL KHURI::IRGC 35154-1 | UL_S | WS | 80 | 116.91 | 20.10 | 38.29 | 24 | 8190.80 | 994.44 | 0.23 | 197.13 | 88.91 |
| RP264 | SINNA SITHIRA KALI::IRGC 51064-1 | UL_S | WS | 84 | 95.48 | 19.70 | 34.17 | 22 | 2023.60 | 952.97 | 0.05 | 179.05 | 44.28 |
| RP266 | SITHAIYAN KOTTAI SAMBA::IRGC 50155-1 | UL_S | WS | 0 | 0.00 | 0.00 | 0.00 | 0 | 0.00 | 0.00 | 0.00 | 0.00 | 0.00 |
| RP267 | SML AWINI::IRGC 13391-1 | UL_S | WS | 83 | 115.39 | 20.44 | 37.52 | 20 | 2038.00 | 956.77 | 0.01 | 177.77 | 20.00 |
| RP268 | SOLOMON RED RICE::IRGC 50950-1 | UL_S | WS | 72 | 110.54 | 20.58 | 34.87 | 24 | 5846.80 | 1031.67 | 0.16 | 186.01 | 67.42 |
| RP269 | SONA::IRGC 26971-C1 | UL_S | WS | 85 | 118.97 | 20.50 | 38.21 | 21 | 5677.60 | 973.83 | 0.05 | 194.45 | 57.32 |
| RP270 | SONAMUKHI::IRGC 46693-1 | UL_S | WS | 81 | 112.58 | 20.65 | 37.63 | 21 | 3155.60 | 968.83 | 0.07 | 190.69 | 41.34 |
| RP272 | SSANGDUJO::IRGC 55632-1 | UL_S | WS | 81 | 110.79 | 20.21 | 38.46 | 21 | 4267.20 | 989.34 | 0.10 | 179.23 | 37.69 |
| RP273 | SUFAID 246::IRGC 28303-1 | UL_S | WS | 77 | 106.33 | 19.85 | 36.73 | 25 | 6808.40 | 978.30 | 0.20 | 193.37 | 92.03 |
| RP274 | SUWEON 311::IRGC 61890-1 | UL_S | WS | 85 | 102.85 | 19.94 | 36.64 | 21 | NA | 952.83 | 0.00 | 183.06 | 22.31 |
| RP275 | T 315::IRGC 54792-1 | UL_S | WS | 83 | 105.26 | 20.07 | 38.54 | 22 | 6402.40 | 993.80 | 0.16 | 188.51 | 72.51 |
| RP276 | TAIPEI WOO CO::IRGC 112-1 | UL_S | WS | 85 | 100.70 | 20.11 | 36.05 | 20 | 1882.80 | 955.12 | 0.08 | 188.54 | 38.95 |
| RP277 | TAITUNG WOO LI::IRGC 111-1 | UL_S | WS | 78 | 120.15 | 19.88 | 36.05 | 23 | 10228.40 | 1010.04 | 0.17 | 196.84 | 86.26 |
| RP278 | TAK SUFAID::IRGC 73127-1 | UL_S | WS | 81 | 101.14 | 19.66 | 35.54 | 20 | 1180.80 | 950.77 | 0.04 | 183.69 | 48.93 |
| RP279 | TAK::IRGC 73124-1 | UL_S | WS | 79 | 113.27 | 21.07 | 39.53 | 23 | 7686.80 | 969.97 | 0.23 | 183.68 | 88.98 |
| RP280 | TIKAL 3::IRGC 50649-1 | UL_S | WS | 78 | 108.30 | 20.35 | 38.17 | 23 | 5347.20 | 991.99 | 0.16 | 190.40 | 76.90 |
| RP281 | TOC 5430::IRGC 70487-1 | UL_S | WS | 82 | 109.10 | 20.44 | 38.96 | 22 | 6404.00 | 958.39 | 0.22 | 193.23 | 78.71 |
| RP282 | TONG GU HONG::IRGC 81026-1 | UL_S | WS | 73 | 110.16 | 20.25 | 37.03 | 21 | 2714.80 | 958.20 | 0.11 | 191.29 | 39.90 |
| RP283 | TSAI YUAN CHON::IRGC 126-1 | UL_S | WS | 80 | 113.64 | 20.35 | 38.50 | 22 | 6035.60 | 969.40 | 0.03 | 188.02 | 29.52 |
| RP284 | TSAO SHENG LI 1::IRGC 1309-1 | UL_S | WS | 0 | 0.00 | 0.00 | 0.00 | 0 | 0.00 | 0.00 | 0.00 | 0.00 | 0.00 |
| RP285 | UPRH 233::IRGC 61667-1 | UL_S | WS | 0 | 0.00 | 0.00 | 0.00 | 0 | 0.00 | 0.00 | 0.00 | 0.00 | 0.00 |
| RP286 | UQUIHUA::IRGC 117037-1 | UL_S | WS | 73 | 117.68 | 20.95 | 40.58 | 22 | 6362.80 | 943.20 | 0.10 | 190.57 | 71.52 |
| RP287 | URAIBOOL::IRGC 52785-1 | UL_S | WS | 76 | 95.56 | 20.04 | 36.27 | 24 | 5630.00 | 970.75 | 0.25 | 186.50 | 79.97 |
| RP288 | VEN THAP::IRGC 56138-1 | UL_S | WS | 83 | 96.00 | 19.75 | 34.89 | 21 | 3206.40 | 931.31 | 0.09 | 191.10 | 61.95 |
| RP289 | WANGA BARUGULU::IRGC 52261-1 | UL_S | WS | 71 | 106.98 | 20.07 | 38.64 | 23 | 5820.00 | 985.19 | 0.16 | 198.55 | 76.29 |
| RP290 | WAR 72-2-1-1::IRGC 117361-1 | UL_S | WS | 73 | 112.37 | 20.58 | 38.41 | 22 | 4808.40 | 1013.76 | 0.10 | 189.05 | 62.09 |
| RP291 | WI BIR SHUN::IRGC 4602-1 | UL_S | WS | 94 | 103.38 | 20.39 | 39.26 | 22 | 1316.40 | 930.90 | 0.08 | 193.83 | 64.83 |
| **Study designation** | **3KG database designation** | **Env** | **Season** | **DTF** | **PH (cm)** | **PL (cm)** | **FlgLA (cm^2^)** | **NBP** | **BMDW (kgha^-1^)** | **GY (kgha^-1^)** | **HI** | **TGW (g)** | **SPKFT (%)** |
| RP292 | WP 36::IRGC 55278-1 | UL_S | WS | 0 | 0.00 | 0.00 | 0.00 | 0 | 0.00 | 0.00 | 0.00 | 0.00 | 0.00 |
| RP293 | WP 65::IRGC 36526-1 | UL_S | WS | 83 | 116.75 | 20.49 | 39.96 | 22 | 2168.80 | 944.09 | 0.08 | 189.30 | 76.95 |
| RP294 | XI GAN JING REN::IRGC 60035-1 | UL_S | WS | 0 | 0.00 | 0.00 | 0.00 | 0 | 0.00 | 0.00 | 0.00 | 0.00 | 0.00 |
| RP295 | XIA ZHI BAI::IRGC 53437-1 | UL_S | WS | 83 | 124.05 | 20.86 | 39.92 | 23 | 10370.80 | 1050.45 | 0.22 | 194.01 | 85.55 |
| RP296 | XITTO::IRGC 6671-1 | UL_S | WS | 0 | 0.00 | 0.00 | 0.00 | 0 | 0.00 | 0.00 | 0.00 | 0.00 | 0.00 |
| RP297 | YA NONG ZAO 4::IRGC 63908-1 | UL_S | WS | 80 | 113.73 | 20.18 | 38.19 | 22 | 4239.60 | 950.55 | 0.02 | 187.19 | 28.13 |
| RP298 | YEBAWYIN::IRGC 33885-1 | UL_S | WS | 0 | 0.00 | 0.00 | 0.00 | 0 | 0.00 | 0.00 | 0.00 | 0.00 | 0.00 |
| RP299 | YONG JIN ZAO 3::IRGC 70441-1 | UL_S | WS | 70 | 105.17 | 20.03 | 35.12 | 24 | 7194.40 | 953.95 | 0.15 | 192.65 | 72.71 |
| RP300 | H 6::IRGC 157-1 | UL_S | WS | 84 | 113.93 | 20.32 | 36.69 | 21 | 5120.40 | 986.39 | 0.10 | 182.81 | 59.26 |
| RP002 | 19::IRGC 70786-1 | UL_S | DS | 103 | 74.73 | 17.29 | 21.57 | 8 | 6446.06 | 102.08 | 0.02 | 80.27 | 13.73 |
| RP003 | 3210::IRGC 116950-1 | UL_S | DS | 114 | 55.71 | 15.81 | 19.72 | 23 | 5319.21 | 106.25 | 0.02 | 120.00 | 25.21 |
| RP004 | 498-2A BR 8::IRGC 5891-1 | UL_S | DS | NA | 55.83 | 17.04 | 20.78 | 4 | 6212.73 | 0.00 | 0.02 | 120.00 | NA |
| RP005 | ADT 12::IRGC 6254-1 | UL_S | DS | 99 | 102.61 | 19.95 | 30.19 | 21 | 7071.38 | 142.08 | 0.02 | 123.02 | 9.09 |
| RP006 | AE NOUA::IRGC 89308-1 | UL_S | DS | NA | 59.48 | 16.68 | 28.70 | 4 | 6023.77 | 0.00 | 0.02 | 120.00 | NA |
| RP007 | ALTAMIRA 9::IRGC 116953-1 | UL_S | DS | 117 | 42.79 | 15.38 | 21.65 | 10 | 6052.21 | 58.00 | 0.02 | 120.00 | 11.79 |
| RP008 | ARAURE 1::IRGC 116956-1 | UL_S | DS | 95 | 71.55 | 17.37 | 21.12 | 30 | 6115.55 | 95.41 | 0.02 | 119.87 | 7.88 |
| RP009 | ARC 10100::IRGC 20709-1 | UL_S | DS | 90 | 81.14 | 16.99 | 29.31 | 16 | 5174.93 | 115.41 | 0.02 | 118.03 | 6.21 |
| RP010 | ARC 10594::IRGC 12524-1 | UL_S | DS | 104 | 84.99 | 17.78 | 24.98 | 19 | 5176.31 | 98.75 | 0.02 | 110.98 | 7.05 |
| RP011 | ARC 10754::IRGC 12603-1 | UL_S | DS | 85 | 86.32 | 17.61 | 22.18 | 42 | 6630.97 | 128.75 | 0.04 | 133.86 | 37.55 |
| RP012 | ARC 10812::IRGC 21074-1 | UL_S | DS | 82 | 91.65 | 17.25 | 23.83 | 16 | 6576.06 | 189.58 | 0.02 | 135.40 | 21.68 |
| RP013 | ARC 11524::IRGC 42672-1 | UL_S | DS | 109 | 85.25 | 17.43 | 32.05 | 14 | 5884.15 | 137.08 | 0.02 | 128.40 | 12.68 |
| RP014 | ARC 11857::IRGC 40972-1 | UL_S | DS | 108 | 80.68 | 17.97 | 26.26 | 13 | 6483.78 | 101.25 | 0.02 | 66.42 | 28.67 |
| RP015 | ARC 11901::IRGC 21727-1 | UL_S | DS | 91 | 90.38 | 17.83 | 29.06 | 26 | 6336.57 | 108.75 | 0.02 | 153.19 | 14.64 |
| RP018 | ARC 13778::IRGC 41216-1 | UL_S | DS | 0 | 0.00 | 0.00 | 0.00 | 0 | 0.00 | 0.00 | 0.00 | 0.00 | 0.00 |
| RP019 | ARC 14060::IRGC 41374-1 | UL_S | DS | NA | 79.53 | 17.52 | 21.33 | 9 | 5911.00 | 145.69 | 0.03 | 95.07 | 13.12 |
| RP020 | ARC 14064::IRGC 41377-1 | UL_S | DS | 0 | 0.00 | 0.00 | 0.00 | 0 | 0.00 | 0.00 | 0.00 | 0.00 | 0.00 |
| RP021 | ARC 14654::IRGC 41663-1 | UL_S | DS | 0 | 0.00 | 0.00 | 0.00 | 0 | 0.00 | 0.00 | 0.00 | 0.00 | 0.00 |
| RP022 | ARC 15873::IRGC 43250-1 | UL_S | DS | 116 | 74.41 | 16.50 | 26.51 | 16 | 6432.22 | 97.91 | 0.02 | 120.00 | 8.11 |
| **Study designation** | **3KG database designation** | **Env** | **Season** | **DTF** | **PH (cm)** | **PL (cm)** | **FlgLA (cm^2^)** | **NBP** | **BMDW (kgha^-1^)** | **GY (kgha^-1^)** | **HI** | **TGW (g)** | **SPKFT (%)** |
| RP023 | ARC 18092::IRGC 42256-1 | UL_S | DS | 84 | 84.73 | 17.19 | 24.73 | 20 | 5529.42 | 124.58 | 0.02 | 138.34 | 11.14 |
| RP024 | ARC 18112::IRGC 42274-1 | UL_S | DS | 111 | 67.77 | 15.92 | 18.97 | 26 | 6150.66 | 99.58 | 0.02 | 56.42 | 8.20 |
| RP025 | ARC 18202::IRGC 42328-1 | UL_S | DS | NA | 84.43 | 17.33 | 25.24 | 5 | 6173.22 | 114.31 | 0.03 | 120.00 | NA |
| RP026 | ARC 18597::IRGC 43299-1 | UL_S | DS | 82 | 93.23 | 16.60 | 28.53 | 22 | 5759.56 | 117.91 | 0.03 | 126.34 | 29.97 |
| RP028 | ASHI BINNI::IRGC 77216-1 | UL_S | DS | 0 | 0.00 | 0.00 | 0.00 | 0 | 0.00 | 0.00 | 0.00 | 0.00 | 0.00 |
| RP029 | ASHMBER::IRGC 27522-1 | UL_S | DS | 0 | 0.00 | 0.00 | 0.00 | 0 | 0.00 | 0.00 | 0.00 | 0.00 | 0.00 |
| RP030 | ASU::IRGC 62154-1 | UL_S | DS | 107 | 78.98 | 17.44 | 23.78 | 28 | 6311.79 | 123.75 | 0.02 | 74.70 | 8.20 |
| RP031 | AUS 171::IRGC 29004-1 | UL_S | DS | 86 | 75.63 | 16.34 | 21.47 | 25 | 5323.33 | 237.91 | 0.05 | 155.90 | 42.96 |
| RP032 | AUS 177::IRGC 29009-1 | UL_S | DS | 83 | 66.88 | 16.02 | 24.20 | 26 | 5496.75 | 117.08 | 0.02 | 74.70 | 11.36 |
| RP033 | AUS 219::IRGC 29031-1 | UL_S | DS | 65 | 88.17 | 16.55 | 27.25 | 21 | 5577.54 | 1684.57 | 0.11 | 163.86 | 40.81 |
| RP034 | AUS 233::IRGC 29036-1 | UL_S | DS | 62 | 97.02 | 17.79 | 29.55 | 32 | 6861.87 | 2227.07 | 0.23 | 163.86 | 80.69 |
| RP036 | AUS 295::IRGC 29083-1 | UL_S | DS | 76 | 87.73 | 16.76 | 21.62 | 44 | 7146.20 | 1247.91 | 0.15 | 155.90 | 75.77 |
| RP037 | AUS 301::IRGC 29089-1 | UL_S | DS | 68 | 94.96 | 17.48 | 27.79 | 45 | 7011.27 | 2340.41 | 0.22 | 195.68 | 75.27 |
| RP038 | AUS 308::IRGC 29096-1 | UL_S | DS | 83 | 87.28 | 17.27 | 24.63 | 45 | 7008.38 | 982.08 | 0.15 | 147.95 | 75.82 |
| RP039 | AUS 329::IRGC 29116-1 | UL_S | DS | 65 | 87.81 | 17.17 | 23.15 | 30 | 6429.07 | 1677.07 | 0.22 | 211.53 | 82.86 |
| RP040 | AUS 344::IRGC 29131-1 | UL_S | DS | 67 | 87.37 | 16.75 | 26.91 | 36 | 6590.38 | 2359.57 | 0.26 | 171.76 | 84.91 |
| RP041 | AUS 439::IRGC 29221-1 | UL_S | DS | 97 | 84.27 | 17.90 | 25.14 | 21 | 5442.75 | 571.24 | 0.06 | 118.44 | 42.80 |
| RP042 | AUS PADDY (RED)::IRGC 44978-1 | UL_S | DS | 64 | 84.57 | 15.61 | 22.50 | 34 | 5978.33 | 1350.41 | 0.21 | 147.89 | 72.70 |
| RP043 | B 4414 F-MR-6-3::IRGC 117310-1 | UL_S | DS | 114 | 60.69 | 17.40 | 26.95 | 22 | 6187.44 | 98.75 | 0.02 | 120.00 | 8.37 |
| RP044 | B 6136 E-3-TB-0-1-5::IRGC 117311-1 | UL_S | DS | 90 | 75.33 | 17.49 | 22.14 | 33 | 5954.44 | 159.58 | 0.03 | 96.94 | 22.76 |
| RP045 | B 6136-3-TB-0-1-5::IRGC 117312-1 | UL_S | DS | 90 | 75.33 | 16.65 | 22.68 | 31 | 5636.41 | 183.75 | 0.02 | 136.72 | 12.57 |
| RP046 | B 6149 F-MR-7::IRGC 117314-1 | UL_S | DS | 99 | 77.99 | 16.31 | 24.15 | 23 | 6094.81 | 102.08 | 0.02 | 148.49 | 8.72 |
| RP047 | BA BAI GU::IRGC 79580-1 | UL_S | DS | 65 | 81.19 | 16.86 | 24.07 | 47 | 6587.79 | 887.08 | 0.14 | 122.43 | 65.79 |
| RP048 | BA SHI ZAO::IRGC 67903-1 | UL_S | DS | 69 | 84.01 | 17.69 | 24.83 | 33 | 5763.39 | 503.75 | 0.10 | 181.15 | 60.04 |
| RP049 | BADAL 1163::IRGC 32796-1 | UL_S | DS | 0 | 0.00 | 0.00 | 0.00 | 0 | 0.00 | 0.00 | 0.00 | 0.00 | 0.00 |
| RP050 | BADUIE::IRGC 53715-1 | UL_S | DS | 115 | 75.88 | 17.27 | 25.36 | 11 | 5776.82 | 93.75 | 0.02 | 120.00 | 8.20 |
| RP051 | BAI HE::IRGC 76438-1 | UL_S | DS | 66 | 73.54 | 16.76 | 21.41 | 38 | 6330.01 | 659.58 | 0.09 | 157.29 | 69.59 |
| RP052 | BAIANG 6::IRGC 6129-1 | UL_S | DS | 0 | 0.00 | 0.00 | 0.00 | 0 | 0.00 | 0.00 | 0.00 | 0.00 | 0.00 |
| **Study designation** | **3KG database designation** | **Env** | **Season** | **DTF** | **PH (cm)** | **PL (cm)** | **FlgLA (cm^2^)** | **NBP** | **BMDW (kgha^-1^)** | **GY (kgha^-1^)** | **HI** | **TGW (g)** | **SPKFT (%)** |
| RP053 | BAK TULSI::IRGC 34831-1 | UL_S | DS | 78 | 86.93 | 17.85 | 25.76 | 26 | 5998.41 | 1567.91 | 0.17 | 187.67 | 74.66 |
| RP054 | BAKASI::IRGC 27074-1 | UL_S | DS | 113 | 62.88 | 16.25 | 25.81 | 32 | 6721.23 | 100.41 | 0.02 | 120.00 | 5.52 |
| RP055 | BALASURIYA A::IRGC 66509-1 | UL_S | DS | NA | 68.57 | 16.56 | 28.74 | 5 | 6677.29 | 54.37 | 0.02 | 68.71 | 9.15 |
| RP056 | BAMOA A 75::IRGC 51101-1 | UL_S | DS | 108 | 51.56 | 15.32 | 22.75 | 39 | 6137.73 | 97.08 | 0.02 | 120.00 | 7.32 |
| RP057 | BANDI::IRGC 17214-1 | UL_S | DS | 0 | 0.00 | 0.00 | 0.00 | 0 | 0.00 | 0.00 | 0.00 | 0.00 | 0.00 |
| RP058 | BANGKOUY::IRGC 94037-1 | UL_S | DS | 110 | 83.98 | 17.68 | 28.32 | 18 | 5983.61 | 97.91 | 0.02 | 120.00 | 5.52 |
| RP059 | BARIK KUDI::IRGC 52807-1 | UL_S | DS | 78 | 75.90 | 17.10 | 24.07 | 44 | 5938.65 | 207.91 | 0.03 | 69.78 | 22.94 |
| RP060 | BAT DO::IRGC 7014-1 | UL_S | DS | 85 | 87.74 | 17.13 | 25.52 | 23 | 6955.17 | 142.08 | 0.02 | 88.47 | 27.12 |
| RP061 | BATHURI::IRGC 25838-1 | UL_S | DS | 57 | 86.63 | 16.63 | 25.87 | 41 | 6883.96 | 3020.41 | 0.23 | 166.17 | 75.31 |
| RP062 | BAZAIL::IRGC 27526-1 | UL_S | DS | 98 | 76.94 | 18.01 | 22.39 | 29 | 5675.18 | 136.25 | 0.03 | 136.23 | 21.24 |
| RP063 | BENGALY MORIMO::IRGC 10976-1 | UL_S | DS | 113 | 56.87 | 16.08 | 18.55 | 22 | 5258.20 | 96.25 | 0.02 | 120.00 | 7.32 |
| RP065 | BHADOIA 303::IRGC 6588-1 | UL_S | DS | 57 | 94.45 | 15.45 | 21.25 | 31 | 6278.72 | 1885.41 | 0.23 | 166.17 | 81.23 |
| RP066 | BK 26::IRGC 45197-1 | UL_S | DS | 0 | 0.00 | 0.00 | 0.00 | 0 | 0.00 | 0.00 | 0.00 | 0.00 | 0.00 |
| RP067 | BKN BR 1031-78-5-4::IRGC 55927-1 | UL_S | DS | 113 | 82.94 | 18.02 | 27.05 | 20 | 6393.85 | 794.38 | 0.03 | 144.49 | 20.51 |
| RP068 | BONG SEN::IRGC 7011-1 | UL_S | DS | 103 | 77.51 | 16.12 | 23.63 | 22 | 5652.88 | 101.25 | 0.02 | 69.19 | 8.92 |
| RP070 | BPI 76 NON SENSITIVE (GREEN)::IRGC 9790-1 | UL_S | DS | 111 | 69.96 | 17.05 | 23.12 | 25 | 5567.64 | 97.08 | 0.02 | 50.75 | 6.41 |
| RP071 | BR 51-115-4::IRGC 43999-1 | UL_S | DS | 113 | 62.47 | 16.63 | 18.95 | 31 | 6080.65 | 100.41 | 0.02 | 120.00 | 8.92 |
| RP072 | BR 5230-46-4::IRGC 117318-1 | UL_S | DS | NA | 70.12 | 16.92 | 21.84 | 4 | 5314.27 | 9.19 | 0.02 | 120.00 | NA |
| RP073 | BR IRGA 409::IRGC 55915-1 | UL_S | DS | 83 | 65.72 | 16.25 | 20.95 | 30 | 5518.53 | 122.08 | 0.02 | 157.29 | 13.57 |
| RP074 | BW 295-5::IRGC 63098-1 | UL_S | DS | 110 | 63.09 | 15.55 | 19.04 | 29 | 6218.18 | 97.08 | 0.02 | 66.44 | 8.72 |
| RP075 | C 1016-1::IRGC 50368-1 | UL_S | DS | 94 | 80.61 | 17.73 | 21.79 | 36 | 6672.28 | 219.58 | 0.02 | 120.00 | 8.92 |
| RP076 | C 166-135::IRGC 50633-1 | UL_S | DS | 113 | 68.07 | 16.55 | 26.89 | 13 | 5579.37 | 104.58 | 0.02 | 69.19 | 8.92 |
| RP077 | C 662083::IRGC 62101-1 | UL_S | DS | 0 | 0.00 | 0.00 | 0.00 | 0 | 0.00 | 0.00 | 0.00 | 0.00 | 0.00 |
| RP079 | CAUVERY::IRGC 45255-1 | UL_S | DS | 84 | 53.87 | 15.68 | 20.50 | 45 | 5231.13 | 123.75 | 0.02 | 66.50 | 8.63 |
| RP080 | CE IN TSAN::IRGC 4362-1 | UL_S | DS | 93 | 81.94 | 16.97 | 23.40 | 18 | 6095.63 | 111.41 | 0.02 | 75.59 | 24.33 |
| RP081 | CEA 3::IRGC 116965-1 | UL_S | DS | 109 | 56.01 | 15.72 | 21.74 | 30 | 5618.55 | 98.75 | 0.02 | 120.00 | 8.72 |
| RP082 | CEMPO MANGGAR::IRGC 27107-1 | UL_S | DS | 0 | 0.00 | 0.00 | 0.00 | 0 | 0.00 | 0.00 | 0.00 | 0.00 | 0.00 |
| RP083 | CHAKOL::IRGC 77226-1 | UL_S | DS | NA | 74.27 | 16.58 | 21.30 | 6 | 5933.00 | 55.41 | 0.02 | 107.35 | 8.26 |
| **Study designation** | **3KG database designation** | **Env** | **Season** | **DTF** | **PH (cm)** | **PL (cm)** | **FlgLA (cm^2^)** | **NBP** | **BMDW (kgha^-1^)** | **GY (kgha^-1^)** | **HI** | **TGW (g)** | **SPKFT (%)** |
| RP084 | CHAM LEK::IRGC 89387-1 | UL_S | DS | 116 | 84.30 | 17.22 | 28.45 | 19 | 6187.17 | 101.25 | 0.02 | 57.44 | 15.77 |
| RP085 | CHAMA (DWARF)::IRGC 69487-1 | UL_S | DS | 118 | 54.61 | 16.90 | 19.52 | 27 | 5179.55 | 101.25 | 0.02 | 120.00 | 14.76 |
| RP086 | CHANDARHAT::IRGC 25845-1 | UL_S | DS | 59 | 75.50 | 16.98 | 17.83 | 32 | 5184.54 | 862.91 | 0.23 | 168.02 | 75.70 |
| RP087 | CHANDINA::IRGC 36420-1 | UL_S | DS | 89 | 58.15 | 16.69 | 22.89 | 44 | 5642.22 | 128.75 | 0.02 | 98.32 | 19.49 |
| RP088 | CHANG LE SAN SHU ZAO::IRGC 63561-1 | UL_S | DS | 86 | 52.12 | 15.41 | 20.19 | 25 | 4747.63 | 97.08 | 0.02 | 95.32 | 6.94 |
| RP089 | CHAO PEUAK DENG::IRGC 11602-1 | UL_S | DS | 110 | 71.28 | 17.13 | 24.70 | 14 | 5685.01 | 52.75 | 0.02 | 120.00 | 10.13 |
| RP090 | CHI SHENG TAO::IRGC 4606-1 | UL_S | DS | 76 | 70.69 | 17.45 | 24.62 | 27 | 5554.42 | 399.58 | 0.09 | 130.14 | 45.78 |
| RP091 | CHIAYI WU-K'O::IRGC 64974-1 | UL_S | DS | 87 | 71.62 | 16.92 | 21.08 | 35 | 6312.49 | 166.25 | 0.04 | 151.54 | 25.94 |
| RP092 | CHIH SHEN LI::IRGC 1306-1 | UL_S | DS | 113 | 64.64 | 15.93 | 22.77 | 15 | 5405.88 | 99.58 | 0.02 | 120.00 | 8.63 |
| RP093 | CHNNOR::IRGC 67485-1 | UL_S | DS | NA | 75.12 | 17.20 | 26.14 | 6 | 5921.79 | 102.08 | 0.02 | 57.02 | 8.63 |
| RP094 | CHUA DAU::IRGC 4785-1 | UL_S | DS | 101 | 84.42 | 17.56 | 19.34 | 35 | 6250.30 | 88.74 | 0.02 | 86.62 | 6.41 |
| RP095 | CHUNGUR BALI::IRGC 25855-1 | UL_S | DS | 65 | 93.31 | 17.99 | 25.21 | 48 | 6354.91 | 1635.41 | 0.20 | 127.62 | 76.44 |
| RP096 | CICA 9::IRGC 53079-1 | UL_S | DS | 111 | 55.50 | 14.58 | 22.95 | 20 | 5274.69 | 102.08 | 0.02 | 94.14 | 8.63 |
| RP097 | CN 44-40-7::IRGC 45368-1 | UL_S | DS | 77 | 68.82 | 17.39 | 24.93 | 30 | 5572.15 | 294.58 | 0.06 | 119.72 | 50.69 |
| RP098 | CO 39::IRGC 51231-1 | UL_S | DS | 71 | 64.65 | 16.97 | 22.24 | 44 | 5523.57 | 579.58 | 0.03 | 142.30 | 22.41 |
| RP099 | CR 60-10::IRGC 15777-1 | UL_S | DS | 119 | 49.01 | 14.99 | 22.28 | 35 | 5446.49 | 131.25 | 0.02 | 160.90 | 7.41 |
| RP100 | CRILLO LA FRIA::IRGC 10793-1 | UL_S | DS | 92 | 83.97 | 16.94 | 24.57 | 31 | 5574.61 | 310.41 | 0.05 | 176.81 | 52.00 |
| RP101 | CSR-90 IR-2::IRGC 117327-1 | UL_S | DS | 109 | 67.94 | 16.03 | 18.06 | 32 | 6106.49 | 98.75 | 0.02 | 120.00 | 7.91 |
| RP102 | CT 9737-6-1-1-2-2P-M::IRGC 117330-1 | UL_S | DS | 108 | 45.29 | 15.27 | 18.52 | 19 | 4812.28 | 95.41 | 0.02 | 120.00 | 7.91 |
| RP103 | CUN GU NUO::IRGC 63576-1 | UL_S | DS | 91 | 56.72 | 16.42 | 24.13 | 20 | 5304.66 | 372.91 | 0.04 | 95.85 | 28.62 |
| RP104 | CUYAMEL 3820::IRGC 116975-1 | UL_S | DS | 93 | 64.86 | 17.41 | 20.19 | 32 | 5400.75 | 349.58 | 0.23 | 66.44 | 44.55 |
| RP105 | DA 11::IRGC 6046-1 | UL_S | DS | 0 | 0.00 | 0.00 | 0.00 | 0 | 0.00 | 0.00 | 0.00 | 0.00 | 0.00 |
| RP106 | DA GANG ZHAN::IRGC 67103-1 | UL_S | DS | 86 | 85.00 | 17.19 | 22.14 | 29 | 6625.50 | 86.25 | 0.02 | 81.34 | 19.38 |
| RP107 | DA NUO (ZHAN)::IRGC 72025-1 | UL_S | DS | 84 | 82.79 | 16.68 | 24.20 | 34 | 6790.55 | 477.91 | 0.04 | 89.30 | 35.39 |
| RP108 | DAA MANSA::IRGC 67559-1 | UL_S | DS | 119 | 64.55 | 16.36 | 20.17 | 10 | 5563.39 | 96.25 | 0.02 | 120.00 | 9.15 |
| RP109 | DANGAR::IRGC 76296-1 | UL_S | DS | 62 | 97.00 | 17.21 | 26.56 | 34 | 6492.40 | 1157.08 | 0.13 | 175.36 | 43.03 |
| RP110 | DHANE BURWA::IRGC 10105-1 | UL_S | DS | 0 | 0.00 | 0.00 | 0.00 | 0 | 0.00 | 0.00 | 0.00 | 0.00 | 0.00 |
| RP111 | DISSI::IRGC 101346-1 | UL_S | DS | NA | 64.64 | 16.58 | 30.08 | 5 | 5972.19 | 0.00 | 0.02 | 120.00 | NA |
| **Study designation** | **3KG database designation** | **Env** | **Season** | **DTF** | **PH (cm)** | **PL (cm)** | **FlgLA (cm^2^)** | **NBP** | **BMDW (kgha^-1^)** | **GY (kgha^-1^)** | **HI** | **TGW (g)** | **SPKFT (%)** |
| RP112 | DUDH KADAR::IRGC 67707-1 | UL_S | DS | 92 | 82.51 | 17.28 | 20.86 | 24 | 5819.71 | 207.08 | 0.03 | 126.60 | 21.34 |
| RP113 | E 2024::IRGC 67958-1 | UL_S | DS | 79 | 56.82 | 15.92 | 19.27 | 60 | 6272.25 | 320.41 | 0.04 | 113.17 | 30.46 |
| RP114 | E 2040::IRGC 67968-1 | UL_S | DS | 71 | 58.00 | 17.36 | 19.21 | 33 | 5505.19 | 752.91 | 0.12 | 137.03 | 61.91 |
| RP115 | E DAW HAWM::IRGC 47938-1 | UL_S | DS | 0 | 0.00 | 0.00 | 0.00 | 0 | 0.00 | 0.00 | 0.00 | 0.00 | 0.00 |
| RP116 | ELONI::IRGC 116980-1 | UL_S | DS | 109 | 49.11 | 15.26 | 19.82 | 22 | 5202.18 | 97.91 | 0.02 | 120.00 | 8.95 |
| RP117 | EPEAL 102::IRGC 78698-1 | UL_S | DS | 113 | 49.21 | 15.05 | 18.64 | 24 | 4852.99 | 84.58 | 0.02 | 120.00 | 8.32 |
| RP118 | ES 21::IRGC 56171-1 | UL_S | DS | 0 | 0.00 | 0.00 | 0.00 | 0 | 0.00 | 0.00 | 0.00 | 0.00 | 0.00 |
| RP119 | EX FOILAEIN (NAPUTO)::IRGC 81675-1 | UL_S | DS | 115 | 63.46 | 16.77 | 22.97 | 14 | 6024.34 | 101.25 | 0.02 | 52.22 | 7.41 |
| RP120 | FEI GAI 122::IRGC 63599-1 | UL_S | DS | 81 | 55.95 | 15.67 | 21.94 | 27 | 5227.74 | 192.91 | 0.03 | 150.47 | 24.08 |
| RP121 | FONAIAP 2::IRGC 116985-1 | UL_S | DS | 0 | 0.00 | 0.00 | 0.00 | 0 | 0.00 | 0.00 | 0.00 | 0.00 | 0.00 |
| RP122 | FU ZAO XIAN::IRGC 63619-1 | UL_S | DS | 96 | 46.50 | 15.16 | 16.90 | 24 | 4397.48 | 90.41 | 0.02 | 57.54 | 15.64 |
| RP123 | G 25::IRGC 45733-1 | UL_S | DS | 0 | 0.00 | 0.00 | 0.00 | 0 | 0.00 | 0.00 | 0.00 | 0.00 | 0.00 |
| RP124 | GAO JIAO BAI::IRGC 68047-1 | UL_S | DS | 61 | 86.63 | 17.38 | 24.92 | 36 | 5770.93 | 1806.24 | 0.22 | 172.24 | 75.97 |
| RP125 | GENIT::IRGC 3272-1 | UL_S | DS | 69 | 75.87 | 16.62 | 22.01 | 39 | 6416.67 | 613.74 | 0.08 | 164.28 | 55.50 |
| RP126 | GOJOL GORIA::IRGC 26629-1 | UL_S | DS | 0 | 0.00 | 0.00 | 0.00 | 0 | 0.00 | 0.00 | 0.00 | 0.00 | 0.00 |
| RP127 | GOKULGANJA::IRGC 45701-1 | UL_S | DS | 0 | 0.00 | 0.00 | 0.00 | 0 | 0.00 | 0.00 | 0.00 | 0.00 | 0.00 |
| RP128 | GUI HUA ZAO::IRGC 68060-1 | UL_S | DS | 65 | 89.44 | 17.13 | 25.43 | 23 | 6049.98 | 972.91 | 0.13 | 148.37 | 70.00 |
| RP130 | HAWM KRUA::IRGC 64333-1 | UL_S | DS | NA | 78.77 | 16.95 | 28.19 | 7 | 6748.37 | 139.54 | 0.03 | 79.39 | 14.73 |
| RP131 | HD 10::IRGC 6638-1 | UL_S | DS | 117 | 77.05 | 17.04 | 21.18 | 11 | 6479.97 | 100.41 | 0.02 | 120.00 | 5.61 |
| RP132 | HE GU TSAO::IRGC 51302-1 | UL_S | DS | 66 | 95.04 | 18.86 | 25.16 | 15 | 5093.71 | 537.91 | 0.08 | 148.37 | 62.62 |
| RP133 | HODARAWALA::IRGC 67631-1 | UL_S | DS | 104 | 75.75 | 15.86 | 27.56 | 32 | 6073.82 | 116.25 | 0.02 | 107.83 | 10.02 |
| RP134 | HOLOI BASH (SOLOI BASH)::IRGC 64778-1 | UL_S | DS | 106 | 82.69 | 16.11 | 25.65 | 27 | 5588.87 | 98.75 | 0.02 | 120.00 | 7.91 |
| RP135 | HTA 22::IRGC 45827-1 | UL_S | DS | 95 | 90.39 | 18.00 | 25.35 | 10 | 5504.94 | 108.75 | 0.02 | 105.63 | 34.86 |
| RP136 | HUA LI ZAO::IRGC 80950-1 | UL_S | DS | 101 | 69.44 | 16.35 | 19.08 | 39 | 5885.24 | 126.25 | 0.02 | 104.87 | 7.62 |
| RP137 | I KUNG PAO::IRGC 114-1 | UL_S | DS | 81 | 81.39 | 17.03 | 20.90 | 27 | 5374.26 | 306.25 | 0.07 | 113.58 | 38.61 |
| RP138 | IA CUBA 17::IRGC 116990-1 | UL_S | DS | 113 | 61.95 | 16.70 | 22.34 | 23 | 5516.11 | 112.91 | 0.02 | 169.40 | 8.95 |
| RP139 | ICTA CRISPO 38::IRGC 116994-1 | UL_S | DS | 90 | 61.65 | 15.77 | 21.35 | 40 | 5566.14 | 266.25 | 0.06 | 132.23 | 38.99 |
| RP140 | ICTA MOTAGUA::IRGC 116995-1 | UL_S | DS | 118 | 57.15 | 16.66 | 20.22 | 26 | 6112.17 | 97.08 | 0.02 | 120.00 | 8.95 |
| **Study designation** | **3KG database designation** | **Env** | **Season** | **DTF** | **PH (cm)** | **PL (cm)** | **FlgLA (cm^2^)** | **NBP** | **BMDW (kgha^-1^)** | **GY (kgha^-1^)** | **HI** | **TGW (g)** | **SPKFT (%)** |
| RP141 | INIAP 6::IRGC 117002-1 | UL_S | DS | 114 | 58.07 | 17.24 | 22.95 | 22 | 5321.67 | 125.51 | 0.02 | 53.85 | 9.09 |
| RP142 | IR 13429-109-2-2-1::IRGC 63491-1 | UL_S | DS | 88 | 62.21 | 16.61 | 18.06 | 37 | 6486.23 | 522.08 | 0.11 | 153.36 | 54.50 |
| RP143 | IR 21015-72-3-3-3-1::IRGC 117004-1 | UL_S | DS | 109 | 52.65 | 15.18 | 22.03 | 31 | 5764.20 | 102.08 | 0.02 | 52.97 | 8.95 |
| RP144 | IR 2344-P1 PB-9-3-2B::IRGC 39317-C1 | UL_S | DS | 77 | 75.94 | 18.14 | 24.25 | 19 | 5039.05 | 373.75 | 0.05 | 144.15 | 45.17 |
| RP146 | IR 63295-AC 209-7::IRGC 117365-1 | UL_S | DS | 109 | 63.82 | 15.38 | 21.03 | 23 | 5185.04 | 103.75 | 0.02 | 120.00 | 9.09 |
| RP147 | IR 69502-6-SRN-3-UBN-1-B::IRGC 117290-1 | UL_S | DS | 101 | 62.98 | 15.77 | 20.03 | 30 | 5785.30 | 107.08 | 0.02 | 65.98 | 8.95 |
| RP148 | IR 75870-5-8-5-B-1::IRGC 117297-1 | UL_S | DS | 109 | 56.56 | 16.38 | 14.61 | 46 | 5789.62 | 124.58 | 0.02 | 145.46 | 8.37 |
| RP149 | IR 77390-1-6-4-19-1-B::IRGC 117303-1 | UL_S | DS | 115 | 60.94 | 16.27 | 23.35 | 12 | 5390.42 | 96.25 | 0.02 | 120.00 | 9.09 |
| RP150 | IR 80310-12-B-1-3-B::IRGC 117307-1 | UL_S | DS | 99 | 70.06 | 16.78 | 22.77 | 21 | 5093.28 | 161.25 | 0.02 | 89.85 | 12.93 |
| RP151 | IR 80340-23-B-12-6-B::IRGC 117309-1 | UL_S | DS | 90 | 79.66 | 17.75 | 22.63 | 26 | 6409.72 | 342.91 | 0.03 | 150.20 | 30.65 |
| RP152 | IRGA 318-11-6-9-2B::IRGC 117339-1 | UL_S | DS | 90 | 68.08 | 16.83 | 20.01 | 22 | 4910.29 | 339.58 | 0.04 | 151.49 | 30.33 |
| RP153 | IRGA 370-38-1-1F-C4-2::IRGC 117342-1 | UL_S | DS | 67 | 62.84 | 16.31 | 20.17 | 43 | 6311.88 | 614.58 | 0.12 | 150.20 | 64.45 |
| RP154 | IRGA 370-42-1-1F-C-1::IRGC 117343-1 | UL_S | DS | 97 | 60.63 | 17.32 | 16.81 | 31 | 5229.18 | 325.41 | 0.06 | 189.98 | 32.41 |
| RP155 | IRGA 659-1-2-2-2::IRGC 117345-1 | UL_S | DS | 76 | 66.17 | 16.96 | 21.05 | 22 | 5475.55 | 74.58 | 0.02 | 48.07 | 7.91 |
| RP156 | IRGA 959-1-2-2F-4-1-4A-6-CA-6X::IRGC 117006-1 | UL_S | DS | 71 | 62.75 | 17.14 | 21.36 | 40 | 5945.65 | 452.91 | 0.10 | 150.51 | 57.87 |
| RP157 | IRI 339::IRGC 46956-1 | UL_S | DS | 99 | 52.92 | 15.51 | 22.11 | 39 | 5551.56 | 112.91 | 0.02 | 118.08 | 14.70 |
| RP158 | IRRIBINI::IRGC 49094-1 | UL_S | DS | 0 | 0.00 | 0.00 | 0.00 | 0 | 0.00 | 0.00 | 0.00 | 0.00 | 0.00 |
| RP159 | JABOR SAIL::IRGC 66831-1 | UL_S | DS | 66 | 86.86 | 17.80 | 28.52 | 39 | 6713.55 | 2070.41 | 0.26 | 160.07 | 82.06 |
| RP160 | JAGLI BORO::IRGC 27516-2 | UL_S | DS | 85 | 89.99 | 16.40 | 17.85 | 44 | 7588.41 | 384.58 | 0.05 | 125.99 | 73.89 |
| RP161 | JAMBALI::IRGC 73101-1 | UL_S | DS | 72 | 77.79 | 16.87 | 24.01 | 28 | 6074.77 | 1458.74 | 0.15 | 156.73 | 75.79 |
| RP162 | JAO LEUANG::IRGC 65866-1 | UL_S | DS | 0 | 0.00 | 0.00 | 0.00 | 0 | 0.00 | 0.00 | 0.00 | 0.00 | 0.00 |
| RP164 | JHODI BIRUN::IRGC 31812-1 | UL_S | DS | 0 | 0.00 | 0.00 | 0.00 | 0 | 0.00 | 0.00 | 0.00 | 0.00 | 0.00 |
| RP165 | JIN JUN DAO::IRGC 59710-1 | UL_S | DS | 52 | 80.15 | 16.77 | 22.51 | 37 | 6565.68 | 2927.07 | 0.30 | 150.47 | 84.02 |
| RP166 | JUMA 62::IRGC 117011-1 | UL_S | DS | 114 | 48.59 | 15.20 | 22.15 | 22 | 4818.54 | 97.91 | 0.02 | 92.37 | 8.37 |
| RP167 | KALABAIL::IRGC 25877-1 | UL_S | DS | 0 | 0.00 | 0.00 | 0.00 | 0 | 0.00 | 0.00 | 0.00 | 0.00 | 0.00 |
| RP168 | KALIA::IRGC 34699-1 | UL_S | DS | 73 | 78.08 | 16.62 | 19.05 | 45 | 6870.07 | 1897.07 | 0.20 | 172.64 | 79.22 |
| RP169 | KALO CHAKOL::IRGC 77258-1 | UL_S | DS | 0 | 0.00 | 0.00 | 0.00 | 0 | 0.00 | 0.00 | 0.00 | 0.00 | 0.00 |
| **Study designation** | **3KG database designation** | **Env** | **Season** | **DTF** | **PH (cm)** | **PL (cm)** | **FlgLA (cm^2^)** | **NBP** | **BMDW (kgha^-1^)** | **GY (kgha^-1^)** | **HI** | **TGW (g)** | **SPKFT (%)** |
| RP170 | KALU ILANKALAYAN::IRGC 36270-1 | UL_S | DS | 113 | 85.22 | 16.95 | 25.03 | 18 | 6471.25 | 97.91 | 0.02 | 58.42 | 7.62 |
| RP171 | KAM PAI::IRGC 78245-1 | UL_S | DS | 0 | 0.00 | 0.00 | 0.00 | 0 | 0.00 | 0.00 | 0.00 | 0.00 | 0.00 |
| RP172 | KEERIPALA CHILL PADDY::IRGC 49790-1 | UL_S | DS | 91 | 80.74 | 17.45 | 24.37 | 33 | 6067.12 | 413.75 | 0.07 | 134.56 | 46.29 |
| RP173 | KETAN SERANG::IRGC 14615-1 | UL_S | DS | 0 | 0.00 | 0.00 | 0.00 | 0 | 0.00 | 0.00 | 0.00 | 0.00 | 0.00 |
| RP174 | KHAO DAWK MALI 105::IRGC 27748-2 | UL_S | DS | 0 | 0.00 | 0.00 | 0.00 | 0 | 0.00 | 0.00 | 0.00 | 0.00 | 0.00 |
| RP175 | KHAO GRADOOK CHAHNG::IRGC 17111-1 | UL_S | DS | 92 | 81.76 | 18.27 | 21.98 | 17 | 5090.64 | 155.41 | 0.04 | 150.52 | 32.22 |
| RP176 | KHAO' HAWM::IRGC 78257-1 | UL_S | DS | 0 | 0.00 | 0.00 | 0.00 | 0 | 0.00 | 0.00 | 0.00 | 0.00 | 0.00 |
| RP177 | KHAO THI RATE::IRGC 58041-1 | UL_S | DS | 0 | 0.00 | 0.00 | 0.00 | 0 | 0.00 | 0.00 | 0.00 | 0.00 | 0.00 |
| RP178 | KHARSU 80::IRGC 28016-1 | UL_S | DS | 53 | 72.62 | 15.88 | 21.05 | 31 | 5581.83 | 1977.91 | 0.24 | 180.59 | 79.96 |
| RP179 | KITRANA 1007::IRGC 68517-1 | UL_S | DS | 114 | 67.90 | 16.41 | 20.68 | 20 | 4964.73 | 97.08 | 0.02 | 120.00 | 7.95 |
| RP180 | KN 1 B 361-1-8-6-9::IRGC 46974-1 | UL_S | DS | 85 | 77.19 | 17.26 | 24.17 | 25 | 5381.44 | 259.58 | 0.08 | 142.56 | 47.69 |
| RP181 | KORASISI::IRGC 5285-1 | UL_S | DS | 0 | 0.00 | 0.00 | 0.00 | 0 | 0.00 | 0.00 | 0.00 | 0.00 | 0.00 |
| RP182 | KOTTEYARAN::IRGC 47383-1 | UL_S | DS | 78 | 82.19 | 16.85 | 22.42 | 40 | 6254.07 | 615.41 | 0.28 | 158.71 | 67.21 |
| RP183 | KOYRA::IRGC 77267-1 | UL_S | DS | NA | 71.52 | 16.07 | 23.30 | 7 | 6126.49 | 80.41 | 0.02 | 53.31 | 4.16 |
| RP184 | KULA KARUPPAN::IRGC 55328-1 | UL_S | DS | 117 | 77.41 | 16.83 | 24.26 | 7 | 6180.83 | 122.91 | 0.02 | 145.61 | 7.95 |
| RP185 | KUNENG::IRGC 71545-1 | UL_S | DS | 0 | 0.00 | 0.00 | 0.00 | 0 | 0.00 | 0.00 | 0.00 | 0.00 | 0.00 |
| RP186 | KURULU WEE (WHITE)::IRGC 66518-1 | UL_S | DS | 64 | 87.37 | 17.13 | 27.29 | 36 | 5903.93 | 1188.74 | 0.17 | 93.08 | 75.38 |
| RP188 | KUSHIARA::IRGC 34709-1 | UL_S | DS | 0 | 0.00 | 0.00 | 0.00 | 0 | 0.00 | 0.00 | 0.00 | 0.00 | 0.00 |
| RP189 | KUTTA::IRGC 52184-1 | UL_S | DS | NA | 72.33 | 16.24 | 20.53 | 7 | 5622.80 | 30.63 | 0.02 | 120.00 | 11.65 |
| RP190 | LABRA::IRGC 74757-1 | UL_S | DS | NA | 75.22 | 17.26 | 26.94 | 4 | 5572.80 | 161.87 | 0.03 | 120.00 | NA |
| RP191 | LAI YIP ZIM::IRGC 4955-1 | UL_S | DS | 81 | 86.32 | 16.51 | 20.01 | 25 | 5288.10 | 335.41 | 0.05 | 126.89 | 32.55 |
| RP192 | LALKA (LAL DHAN)::IRGC 64946-1 | UL_S | DS | 0 | 0.00 | 0.00 | 0.00 | 0 | 0.00 | 0.00 | 0.00 | 0.00 | 0.00 |
| RP193 | LALSAITA::IRGC 43915-1 | UL_S | DS | 65 | 86.64 | 16.62 | 23.05 | 42 | 7437.88 | 2416.24 | 0.13 | 172.64 | 57.00 |
| RP194 | LARHA MUGAD::IRGC 52339-1 | UL_S | DS | 94 | 79.09 | 17.44 | 28.76 | 22 | 5607.99 | 179.58 | 0.02 | 118.93 | 15.99 |
| RP196 | LENJA MURALI::IRGC 66815-1 | UL_S | DS | 63 | 74.25 | 16.28 | 30.05 | 4 | 5558.69 | 117.91 | 0.02 | 120.00 | NA |
| RP197 | LEUANG YAI 29-12-2::IRGC 881-1 | UL_S | DS | 0 | 0.00 | 0.00 | 0.00 | 0 | 0.00 | 0.00 | 0.00 | 0.00 | 0.00 |
| RP198 | LU MAO ZHAN::IRGC 68159-1 | UL_S | DS | 55 | 94.46 | 17.80 | 21.56 | 38 | 5887.90 | 1819.58 | 0.31 | 158.02 | 77.88 |
| RP199 | LUA CHAN HUONG::IRGC 16800-1 | UL_S | DS | 0 | 0.00 | 0.00 | 0.00 | 0 | 0.00 | 0.00 | 0.00 | 0.00 | 0.00 |
| **Study designation** | **3KG database designation** | **Env** | **Season** | **DTF** | **PH (cm)** | **PL (cm)** | **FlgLA (cm^2^)** | **NBP** | **BMDW (kgha^-1^)** | **GY (kgha^-1^)** | **HI** | **TGW (g)** | **SPKFT (%)** |
| RP200 | MAKALIOKA::IRGC 376-1 | UL_S | DS | 0 | 0.00 | 0.00 | 0.00 | 0 | 0.00 | 0.00 | 0.00 | 0.00 | 0.00 |
| RP201 | MAKRO::IRGC 74763-1 | UL_S | DS | 114 | 80.44 | 16.99 | 31.44 | 9 | 5859.03 | 97.91 | 0.02 | 120.00 | 8.43 |
| RP202 | MAMORIAKA::IRGC 68672-1 | UL_S | DS | 90 | 88.11 | 17.17 | 24.61 | 28 | 6648.33 | 77.91 | 0.02 | 155.66 | 12.46 |
| RP203 | MEI FENG 9::IRGC 63735-1 | UL_S | DS | 81 | 53.01 | 15.90 | 21.96 | 22 | 4940.65 | 149.58 | 0.05 | 208.50 | 30.64 |
| RP204 | MEKENZIE SMALL::IRGC 49895-1 | UL_S | DS | 0 | 0.00 | 0.00 | 0.00 | 0 | 0.00 | 0.00 | 0.00 | 0.00 | 0.00 |
| RP205 | MELEKE::IRGC 56823-1 | UL_S | DS | NA | 67.63 | 16.44 | 22.70 | 8 | 5796.50 | 52.42 | 0.02 | 120.00 | 9.15 |
| RP206 | MENTIK TJERE BELUT::IRGC 18254-1 | UL_S | DS | 0 | 0.00 | 0.00 | 0.00 | 0 | 0.00 | 0.00 | 0.00 | 0.00 | 0.00 |
| RP207 | MILYANG 30::IRGC 46977-1 | UL_S | DS | 113 | 44.83 | 14.96 | 18.99 | 36 | 4856.93 | 98.75 | 0.02 | 120.00 | 8.61 |
| RP208 | MILYANG 77::IRGC 69340-1 | UL_S | DS | 91 | 56.19 | 15.80 | 17.34 | 32 | 4993.06 | 105.41 | 0.03 | 89.09 | 20.16 |
| RP209 | MIN KE ZHAN::IRGC 72230-1 | UL_S | DS | 88 | 60.17 | 16.31 | 20.89 | 28 | 5585.03 | 132.08 | 0.02 | 57.27 | 8.61 |
| RP210 | MIN ZAO 6::IRGC 63772-1 | UL_S | DS | 96 | 51.62 | 15.88 | 18.96 | 33 | 5383.40 | 137.91 | 0.04 | 136.82 | 26.22 |
| RP211 | MODDAI KARUPPAN::IRGC 15465-1 | UL_S | DS | NA | 86.45 | 17.08 | 21.66 | 7 | 6534.86 | 15.12 | 0.02 | 120.00 | 7.47 |
| RP212 | MUKKALA BAZAL::IRGC 77279-1 | UL_S | DS | 110 | 68.16 | 17.18 | 23.14 | 17 | 5516.18 | 55.76 | 0.02 | 120.00 | 9.15 |
| RP213 | MULLIKURUVA::IRGC 77529-1 | UL_S | DS | 0 | 0.00 | 0.00 | 0.00 | 0 | 0.00 | 0.00 | 0.00 | 0.00 | 0.00 |
| RP214 | MUTA GANJE::IRGC 26744-1 | UL_S | DS | 0 | 0.00 | 0.00 | 0.00 | 0 | 0.00 | 0.00 | 0.00 | 0.00 | 0.00 |
| RP215 | MUTTU SAMBA::IRGC 36333-1 | UL_S | DS | NA | 76.68 | 16.92 | 18.28 | 9 | 6016.65 | 51.77 | 0.02 | 69.23 | 9.20 |
| RP216 | N 22::IRGC 46459-1 | UL_S | DS | 81 | 80.63 | 17.16 | 25.29 | 28 | 5556.88 | 861.24 | 0.05 | 137.32 | 44.50 |
| RP217 | Na souan::IRGC 11889-1 | UL_S | DS | 0 | 0.00 | 0.00 | 0.00 | 0 | 0.00 | 0.00 | 0.00 | 0.00 | 0.00 |
| RP218 | NAZIRA SAIL::IRGC 77284-1 | UL_S | DS | 0 | 0.00 | 0.00 | 0.00 | 0 | 0.00 | 0.00 | 0.00 | 0.00 | 0.00 |
| RP219 | NCS 194::IRGC 51932-1 | UL_S | DS | 110 | 73.98 | 16.29 | 26.27 | 25 | 5775.66 | 123.75 | 0.02 | 214.13 | 8.61 |
| RP220 | NCS 237::IRGC 62202-1 | UL_S | DS | 111 | 63.68 | 17.19 | 31.79 | 9 | 5769.98 | 59.23 | 0.02 | 120.00 | 10.24 |
| RP222 | NCS 964 C::IRGC 62604-1 | UL_S | DS | 109 | 66.75 | 16.29 | 23.41 | 24 | 6465.47 | 97.91 | 0.02 | 102.93 | 19.88 |
| RP223 | NIAO YAO::IRGC 5496-1 | UL_S | DS | NA | 70.86 | 15.87 | 25.12 | 12 | 6310.30 | 110.41 | 0.02 | 145.21 | 8.37 |
| RP224 | NIBARI::IRGC 67742-1 | UL_S | DS | 82 | 87.37 | 17.70 | 23.55 | 34 | 6121.81 | 527.08 | 0.09 | 144.38 | 59.43 |
| RP225 | NONA BOKRA::IRGC 22710-C1 | UL_S | DS | NA | 80.89 | 16.83 | 29.42 | 5 | 5977.62 | 50.60 | 0.02 | 120.00 | NA |
| RP226 | NOROI::IRGC 31611-1 | UL_S | DS | 59 | 100.06 | 16.71 | 22.85 | 34 | 6371.69 | 2257.91 | 0.26 | 212.41 | 79.81 |
| RP228 | NX 3533::IRGC 63796-1 | UL_S | DS | 92 | 67.05 | 16.54 | 21.65 | 39 | 5905.31 | 132.08 | 0.03 | 161.29 | 20.50 |
| RP229 | O. SATIVA::IRGC 17083-1 | UL_S | DS | 114 | 73.69 | 18.57 | 26.37 | 43 | 6680.54 | 99.58 | 0.02 | 120.00 | 8.61 |
| **Study designation** | **3KG database designation** | **Env** | **Season** | **DTF** | **PH (cm)** | **PL (cm)** | **FlgLA (cm^2^)** | **NBP** | **BMDW (kgha^-1^)** | **GY (kgha^-1^)** | **HI** | **TGW (g)** | **SPKFT (%)** |
| RP230 | PAH WEAN::IRGC 78276-1 | UL_S | DS | 0 | 0.00 | 0.00 | 0.00 | 0 | 0.00 | 0.00 | 0.00 | 0.00 | 0.00 |
| RP231 | PAI CHUEH CHIU LIU::IRGC 34259-1 | UL_S | DS | 91 | 62.92 | 16.88 | 18.94 | 25 | 5033.27 | 93.75 | 0.02 | 134.75 | 13.45 |
| RP232 | PAI YI PING::IRGC 1368-1 | UL_S | DS | 70 | 78.96 | 17.53 | 20.56 | 32 | 5391.50 | 492.08 | 0.13 | 160.29 | 77.41 |
| RP233 | PALEPYU::IRGC 33549-1 | UL_S | DS | 67 | 86.50 | 17.10 | 23.22 | 44 | 6975.09 | 1905.41 | 0.20 | 190.64 | 82.07 |
| RP234 | PARA NELLU::IRGC 50009-1 | UL_S | DS | 70 | 93.73 | 18.03 | 21.99 | 27 | 6251.76 | 1127.08 | 0.10 | 190.64 | 65.13 |
| RP235 | PATISAIL::IRGC 37562-1 | UL_S | DS | 0 | 0.00 | 0.00 | 0.00 | 0 | 0.00 | 0.00 | 0.00 | 0.00 | 0.00 |
| RP237 | PERUNEL::IRGC 63113-1 | UL_S | DS | 91 | 102.11 | 17.95 | 24.83 | 28 | 6555.08 | 1011.24 | 0.09 | 182.58 | 64.91 |
| RP238 | PICO NEGRO::IRGC 55849-1 | UL_S | DS | 0 | 0.00 | 0.00 | 0.00 | 0 | 0.00 | 0.00 | 0.00 | 0.00 | 0.00 |
| RP239 | PLI KHAO::IRGC 64596-1 | UL_S | DS | NA | 84.50 | 17.34 | 25.79 | 7 | 6124.04 | 137.44 | 0.03 | 55.18 | 13.27 |
| RP240 | PODI HEENATI::IRGC 36345-1 | UL_S | DS | 97 | 70.48 | 15.94 | 21.04 | 34 | 5844.40 | 166.25 | 0.02 | 80.51 | 9.32 |
| RP241 | PSBRC 50::IRGC 99706-1 | UL_S | DS | 90 | 63.05 | 16.34 | 19.79 | 28 | 5669.83 | 99.58 | 0.02 | 157.11 | 7.30 |
| RP242 | PSBRC 68::IRGC 99711-1 | UL_S | DS | 110 | 71.16 | 17.10 | 24.37 | 31 | 5971.68 | 97.91 | 0.02 | 68.09 | 12.57 |
| RP243 | PSBRC 88::IRGC 99717-1 | UL_S | DS | 109 | 61.72 | 16.17 | 24.88 | 33 | 6026.81 | 99.58 | 0.02 | 64.64 | 7.30 |
| RP244 | PULUT BARAYA::IRGC 27393-1 | UL_S | DS | 0 | 0.00 | 0.00 | 0.00 | 0 | 0.00 | 0.00 | 0.00 | 0.00 | 0.00 |
| RP245 | PURA BINNI::IRGC 26772-1 | UL_S | DS | 0 | 0.00 | 0.00 | 0.00 | 0 | 0.00 | 0.00 | 0.00 | 0.00 | 0.00 |
| RP247 | QUERO ASSAN::IRGC 28860-1 | UL_S | DS | NA | 65.93 | 16.40 | 20.75 | 6 | 4899.85 | 40.79 | 0.02 | 120.00 | 8.32 |
| RP248 | RACE PERUMAL::IRGC 55347-1 | UL_S | DS | 72 | 80.63 | 16.85 | 16.20 | 31 | 6346.59 | 457.08 | 0.06 | 159.77 | 42.82 |
| RP250 | RELLY::IRGC 14623-1 | UL_S | DS | 0 | 0.00 | 0.00 | 0.00 | 0 | 0.00 | 0.00 | 0.00 | 0.00 | 0.00 |
| RP251 | RIZ TYPE SORGHO::IRGC 69015-1 | UL_S | DS | 0 | 0.00 | 0.00 | 0.00 | 0 | 0.00 | 0.00 | 0.00 | 0.00 | 0.00 |
| RP252 | ROJOFOTSY::IRGC 69402-1 | UL_S | DS | 116 | 78.52 | 17.38 | 27.24 | 11 | 5237.54 | 96.25 | 0.02 | 120.00 | 8.44 |
| RP253 | RPA 5929 (K 45)::IRGC 33963-1 | UL_S | DS | 91 | 57.32 | 16.09 | 21.84 | 11 | 5540.74 | 99.58 | 0.02 | 52.79 | 6.05 |
| RP254 | RPW 9-4 (SS 1)::IRGC 50690-1 | UL_S | DS | 109 | 56.10 | 16.03 | 19.92 | 31 | 5513.03 | 100.41 | 0.02 | 120.00 | 8.44 |
| RP255 | RR 272-17-829::IRGC 117354-1 | UL_S | DS | 95 | 58.87 | 15.72 | 20.38 | 33 | 5212.70 | 115.41 | 0.02 | 144.00 | 6.21 |
| RP256 | RTS 16::IRGC 8235-1 | UL_S | DS | 0 | 0.00 | 0.00 | 0.00 | 0 | 0.00 | 0.00 | 0.00 | 0.00 | 0.00 |
| RP257 | RUSTIC::IRGC 117026-1 | UL_S | DS | 113 | 52.41 | 15.94 | 19.83 | 41 | 5676.03 | 96.25 | 0.02 | 120.00 | 8.44 |
| RP258 | SADA RUPA::IRGC 77299-1 | UL_S | DS | NA | 73.13 | 16.56 | 26.06 | 7 | 5875.25 | 179.48 | 0.03 | 81.53 | 15.83 |
| RP259 | SAN CHIAO TSWEN::IRGC 1565-1 | UL_S | DS | 92 | 81.91 | 17.78 | 20.94 | 42 | 7226.12 | 209.58 | 0.04 | 82.11 | 30.37 |
| RP260 | SAN SHIH TSI::IRGC 1038-1 | UL_S | DS | 89 | 75.32 | 16.09 | 20.46 | 31 | 5697.28 | 230.41 | 0.03 | 175.68 | 36.07 |
| **Study designation** | **3KG database designation** | **Env** | **Season** | **DTF** | **PH (cm)** | **PL (cm)** | **FlgLA (cm^2^)** | **NBP** | **BMDW (kgha^-1^)** | **GY (kgha^-1^)** | **HI** | **TGW (g)** | **SPKFT (%)** |
| RP261 | SIGARDIS::IRGC 15555-1 | UL_S | DS | 0 | 0.00 | 0.00 | 0.00 | 0 | 0.00 | 0.00 | 0.00 | 0.00 | 0.00 |
| RP262 | SIMET 2::IRGC 25734-1 | UL_S | DS | 0 | 0.00 | 0.00 | 0.00 | 0 | 0.00 | 0.00 | 0.00 | 0.00 | 0.00 |
| RP263 | SIMUL KHURI::IRGC 35154-1 | UL_S | DS | 63 | 92.94 | 16.78 | 31.76 | 25 | 5638.60 | 1480.41 | 0.16 | 173.72 | 77.05 |
| RP264 | SINNA SITHIRA KALI::IRGC 51064-1 | UL_S | DS | NA | 59.46 | 16.09 | 24.52 | 7 | 5727.14 | 53.93 | 0.02 | 120.00 | 7.43 |
| RP266 | SITHAIYAN KOTTAI SAMBA::IRGC 50155-1 | UL_S | DS | 0 | 0.00 | 0.00 | 0.00 | 0 | 0.00 | 0.00 | 0.00 | 0.00 | 0.00 |
| RP267 | SML AWINI::IRGC 13391-1 | UL_S | DS | 113 | 57.84 | 16.43 | 20.24 | 26 | 5001.34 | 97.91 | 0.02 | 120.00 | 7.88 |
| RP268 | SOLOMON RED RICE::IRGC 50950-1 | UL_S | DS | 114 | 75.92 | 16.90 | 26.73 | 23 | 5707.85 | 97.91 | 0.02 | 56.25 | 8.77 |
| RP269 | SONA::IRGC 26971-C1 | UL_S | DS | 111 | 50.50 | 15.67 | 23.80 | 21 | 5063.37 | 97.91 | 0.02 | 120.00 | 7.19 |
| RP270 | SONAMUKHI::IRGC 46693-1 | UL_S | DS | 59 | 92.83 | 15.98 | 27.12 | 29 | 5583.89 | 1548.74 | 0.25 | 127.45 | 80.66 |
| RP272 | SSANGDUJO::IRGC 55632-1 | UL_S | DS | 88 | 82.24 | 17.36 | 22.71 | 32 | 6099.57 | 141.25 | 0.02 | 97.93 | 18.56 |
| RP273 | SUFAID 246::IRGC 28303-1 | UL_S | DS | 73 | 83.94 | 17.92 | 22.63 | 28 | 5897.24 | 940.41 | 0.12 | 133.94 | 78.77 |
| RP274 | SUWEON 311::IRGC 61890-1 | UL_S | DS | 73 | 54.50 | 17.10 | 20.03 | 36 | 5389.68 | 142.08 | 0.03 | 169.53 | 27.10 |
| RP275 | T 315::IRGC 54792-1 | UL_S | DS | 114 | 81.23 | 18.00 | 26.53 | 18 | 5924.11 | 97.08 | 0.02 | 67.13 | 8.77 |
| RP276 | TAIPEI WOO CO::IRGC 112-1 | UL_S | DS | 99 | 77.37 | 17.53 | 20.59 | 22 | 5277.89 | 121.25 | 0.03 | 82.02 | 25.03 |
| RP277 | TAITUNG WOO LI::IRGC 111-1 | UL_S | DS | 98 | 70.29 | 17.02 | 28.95 | 16 | 5852.85 | 105.41 | 0.02 | 55.45 | 8.28 |
| RP278 | TAK SUFAID::IRGC 73127-1 | UL_S | DS | 79 | 76.13 | 16.40 | 22.71 | 24 | 5164.21 | 264.58 | 0.03 | 149.85 | 40.49 |
| RP279 | TAK::IRGC 73124-1 | UL_S | DS | 84 | 80.37 | 15.69 | 19.76 | 57 | 6235.08 | 335.41 | 0.05 | 96.42 | 49.27 |
| RP280 | TIKAL 3::IRGC 50649-1 | UL_S | DS | 99 | 54.36 | 14.65 | 21.03 | 22 | 5108.59 | 97.91 | 0.02 | 120.00 | 8.28 |
| RP281 | TOC 5430::IRGC 70487-1 | UL_S | DS | 112 | 60.40 | 16.85 | 20.78 | 24 | 5214.25 | 102.91 | 0.02 | 58.15 | 8.28 |
| RP282 | TONG GU HONG::IRGC 81026-1 | UL_S | DS | 82 | 77.66 | 17.36 | 22.50 | 41 | 6789.38 | 179.58 | 0.03 | 129.75 | 33.10 |
| RP283 | TSAI YUAN CHON::IRGC 126-1 | UL_S | DS | 92 | 79.43 | 16.34 | 21.98 | 24 | 6219.36 | 177.08 | 0.04 | 108.27 | 34.59 |
| RP284 | TSAO SHENG LI 1::IRGC 1309-1 | UL_S | DS | 108 | 71.11 | 16.86 | 23.79 | 25 | 5976.88 | 95.41 | 0.02 | 120.00 | 7.88 |
| RP285 | UPRH 233::IRGC 61667-1 | UL_S | DS | 85 | 53.43 | 16.09 | 20.18 | 25 | 4996.45 | 122.91 | 0.03 | 110.98 | 18.23 |
| RP286 | UQUIHUA::IRGC 117037-1 | UL_S | DS | 107 | 59.39 | 16.14 | 20.31 | 23 | 5030.20 | 99.58 | 0.02 | 59.06 | 11.66 |
| RP287 | URAIBOOL::IRGC 52785-1 | UL_S | DS | 89 | 88.90 | 17.75 | 22.98 | 19 | 6022.29 | 164.58 | 0.02 | 105.46 | 16.48 |
| RP288 | VEN THAP::IRGC 56138-1 | UL_S | DS | 85 | 82.47 | 17.11 | 20.92 | 30 | 6191.10 | 96.25 | 0.02 | 131.90 | 8.82 |
| RP289 | WANGA BARUGULU::IRGC 52261-1 | UL_S | DS | 78 | 81.29 | 17.11 | 20.30 | 34 | 5920.55 | 231.25 | 0.04 | 135.04 | 28.00 |
| RP290 | WAR 72-2-1-1::IRGC 117361-1 | UL_S | DS | NA | 63.66 | 16.06 | 25.88 | 6 | 6180.03 | 96.25 | 0.02 | 120.00 | 11.19 |
| **Study designation** | **3KG database designation** | **Env** | **Season** | **DTF** | **PH (cm)** | **PL (cm)** | **FlgLA (cm^2^)** | **NBP** | **BMDW (kgha^-1^)** | **GY (kgha^-1^)** | **HI** | **TGW (g)** | **SPKFT (%)** |
| RP291 | WI BIR SHUN::IRGC 4602-1 | UL_S | DS | 108 | 71.70 | 16.18 | 23.90 | 23 | 6195.36 | 97.91 | 0.02 | 120.00 | 7.88 |
| RP292 | WP 36::IRGC 55278-1 | UL_S | DS | 0 | 0.00 | 0.00 | 0.00 | 0 | 0.00 | 0.00 | 0.00 | 0.00 | 0.00 |
| RP293 | WP 65::IRGC 36526-1 | UL_S | DS | 114 | 56.21 | 15.34 | 20.21 | 20 | 5436.26 | 95.41 | 0.02 | 120.00 | 6.83 |
| RP294 | XI GAN JING REN::IRGC 60035-1 | UL_S | DS | 56 | 79.96 | 16.98 | 24.26 | 30 | 5724.33 | 1268.74 | 0.17 | 150.20 | 75.31 |
| RP295 | XIA ZHI BAI::IRGC 53437-1 | UL_S | DS | 61 | 80.99 | 16.82 | 19.89 | 35 | 6187.70 | 1763.74 | 0.23 | 119.13 | 81.87 |
| RP296 | XITTO::IRGC 6671-1 | UL_S | DS | NA | 82.08 | 16.34 | 29.07 | 7 | 5759.58 | 69.67 | 0.02 | 119.97 | 11.79 |
| RP297 | YA NONG ZAO 4::IRGC 63908-1 | UL_S | DS | 93 | 53.41 | 15.59 | 19.24 | 22 | 4642.52 | 162.91 | 0.02 | 111.18 | 18.16 |
| RP298 | YEBAWYIN::IRGC 33885-1 | UL_S | DS | 114 | 79.39 | 17.19 | 27.59 | 20 | 6063.84 | 108.75 | 0.02 | 79.16 | 14.20 |
| RP299 | YONG JIN ZAO 3::IRGC 70441-1 | UL_S | DS | 72 | 61.38 | 17.03 | 26.05 | 30 | 5473.56 | 225.41 | 0.05 | 150.95 | 33.24 |
| RP300 | H 6::IRGC 157-1 | UL_S | DS | 114 | 84.42 | 17.80 | 24.44 | 13 | 6790.84 | 98.75 | 0.02 | 120.00 | 5.61 |

**Table S6** Allelic variation at the GWAS-identified significant loci in the selected 7 accessions for grain yield under lowland and upland drought

|  | **Allele** | | **LL_S_WS** | | **LL_S_DS** | | **UL_S_WS** | | **UL_S_DS** | | **Selected promising accessions** | | | | | | |
| --- | --- | --- | --- | --- | --- | --- | --- | --- | --- | --- | --- | --- | --- | --- | --- | --- | --- |
| **SNP id** | **Major** | **Minor** | **ME** | **me** | **ME** | **me** | **ME** | **me** | **ME** | **me** | **AUS 329** | **AUS 344** | **CHUNGUR BALI** | **DANGAR** | **LAL SAITA** | **PARA NELLU** | **SIMUL KHURI** |
| S1_3473291 | T | C | 477 | 0 | 142 | 0 | 858 | 0 | 330 | 0 | T | T | T | T | T | T | T |
| S1_3824622 | G | A | 480 | 338 | 135 | 282 | 853 | 705 | 324 | 231 | G | G | G | G | G | G | G |
| S1_24304274 | G | T | 476 | 502 | 135 | 297 | 854 | 692 | 299 | 1228 | G | G | G | G | T | G | G |
| S1_33418648 | G | A | 486 | 148 | 141 | 69 | 849 | 852 | 330 | 92 | G | G | G | G | G | G | G |
| S1_33689947 | T | C | 458 | 511 | 160 | 92 | 852 | 848 | 411 | 158 | T | T | T | T | T | T | T |
| S1_34359106 | C | G | 447 | 543 | 141 | 132 | 842 | 873 | 246 | 470 | G | G | C | G | G | C | G |
| S1_34400345 | G | A | 461 | 536 | 149 | 97 | 846 | 863 | 359 | 179 | G | G | G | G | G | G | G |
| S1_37960019 | C | T | 477 | 494 | 139 | 143 | 848 | 871 | 338 | 110 | C | C | C | C | C | C | C |
| S1_38418739 | A | G | 466 | 505 | 146 | 109 | 843 | 880 | 384 | 146 | A | A | A | A | A | A | A |
| S1_39044781 | G | A | 469 | 490 | 152 | 111 | 836 | 876 | 400 | 162 | G | G | G | G | G | G | G |
| S1_40390527 | G | A | 477 | 0 | 142 | 0 | 858 | 0 | 330 | 0 | G | G | G | G | G | G | G |
| S2_17808669 | G | T | 489 | 275 | 145 | 38 | 849 | 862 | 340 | 42 | G | G | G | G | G | G | G |
| S2_18794394 | G | A | 473 | 590 | 137 | 191 | 850 | 846 | 304 | 1033 | A | A | G | G | G | G | G |
| S2_19821147 | C | T | 472 | 586 | 141 | 116 | 846 | 902 | 318 | 465 | C | C | C | C | C | C | C |
| S2_20248668 | G | A | 469 | 590 | 129 | 278 | 849 | 856 | 257 | 1277 | A | A | G | G | G | G | A |
| S2_20774488 | C | A | 472 | 588 | 132 | 289 | 852 | 755 | 262 | 1972 | A | A | M | C | A | C | C |
| S2_21953460 | C | T | 471 | 598 | 132 | 242 | 852 | 766 | 295 | 928 | C | C | C | C | C | T | C |
| S2_22142784 | A | G | 475 | 504 | 131 | 280 | 847 | 908 | 297 | 792 | A | A | A | G | A | G | A |
| S2_22201226 | C | T | 470 | 588 | 132 | 267 | 850 | 890 | 276 | 1308 | T | T | C | C | C | C | C |
| S2_23065933 | C | T | 476 | 497 | 137 | 215 | 850 | 836 | 312 | 730 | C | C | C | C | C | C | C |
| S2_24797737 | C | T | 478 | 387 | 140 | 56 | 847 | 980 | 326 | 124 | C | C | C | C | C | C | C |
| S2_25205930 | G | C | 478 | 387 | 140 | 56 | 847 | 980 | 326 | 124 | G | G | G | G | G | G | G |
|  | **Allele** | | **LL_S_WS** | | **LL_S_DS** | | **UL_S_WS** | | **UL_S_DS** | | **Selected promising accessions** | | | | | | |
| **SNP id** | **Major** | **Minor** | **ME** | **me** | **ME** | **me** | **ME** | **me** | **ME** | **me** | **AUS 329** | **AUS 344** | **CHUNGUR BALI** | **DANGAR** | **LAL SAITA** | **PARA NELLU** | **SIMUL KHURI** |
| S2_25979458 | G | A | 475 | 476 | 136 | 245 | 850 | 780 | 302 | 1052 | G | G | G | G | R | G | G |
| S2_29167141 | C | T | 474 | 502 | 137 | 244 | 860 | 552 | 325 | 308 | C | C | C | C | C | T | C |
| S3_28646420 | A | G | 475 | 573 | 135 | 205 | 858 | 0 | 300 | 1568 | R | A | A | A | A | A | A |
| S3_33600040 | A | G | 475 | 481 | 116 | 169 | 860 | 833 | 212 | 462 | G | G | G | G | G | A | G |
| S3_33600989 | A | T | 475 | 478 | 116 | 170 | 860 | 835 | 212 | 474 | T | T | T | T | T | A | T |
| S4_1441702 | T | G | 479 | 582 | 139 | 635 | 854 | 0 | 327 | 1127 | T | T | T | T | T | G | T |
| S4_25920763 | G | T | 480 | 0 | 132 | 0 | 854 | 0 | 308 | 0 | G | G | G | G | G | G | G |
| S4_31088649 | C | T | 478 | 410 | 129 | 392 | 844 | 970 | 282 | 1143 | C | C | T | C | C | C | C |
| S5_4140355 | G | A | 474 | 508 | 133 | 199 | 849 | 921 | 294 | 648 | G | G | G | G | G | G | G |
| S5_4266313 | C | T | 476 | 479 | 133 | 232 | 848 | 871 | 301 | 647 | C | C | C | C | C | C | C |
| S5_4502747 | T | A | 477 | 582 | 142 | 82 | 858 | 965 | 330 | 97 | T | T | T | T | T | T | T |
| S5_4502961 | G | A | 477 | 582 | 142 | 82 | 861 | 965 | 326 | 97 | G | G | G | G | G | G | G |
| S5_4505637 | A | G | 477 | 582 | 142 | 82 | 858 | 965 | 330 | 97 | A | A | A | A | A | A | A |
| S5_5891992 | C | T | 495 | 123 | 145 | 23 | 850 | 849 | 338 | 15 | C | C | C | C | C | C | C |
| S5_12494100 | T | A | 475 | 578 | 135 | 237 | 862 | 490 | 298 | 1041 | W | A | T | T | T | T | T |
| S5_19224697 | A | T | 472 | 585 | 125 | 522 | 853 | 745 | 274 | 1534 | A | W | A | A | T | A | A |
| S5_24708194 | T | C | 474 | 518 | 143 | 63 | 844 | 943 | 334 | 110 | T | T | T | T | T | T | T |
| S5_29913640 | T | C | 477 | 0 | 142 | 0 | 858 | 0 | 330 | 0 | T | T | T | T | T | T | T |
| S6_6025083 | C | T | 473 | 592 | 133 | 325 | 860 | 487 | 320 | 409 | C | C | C | C | C | T | C |
| S6_7611279 | C | T | 478 | 477 | 122 | 157 | 838 | 858 | 404 | 240 | C | C | C | C | C | T | C |
| S6_7749410 | G | A | 475 | 476 | 115 | 168 | 855 | 841 | 378 | 260 | G | G | G | G | G | A | G |
| S6_9539728 | T | G | 489 | 450 | 138 | 138 | 849 | 846 | 204 | 560 | G | G | G | G | G | T | G |
| S6_10279310 | G | A | 465 | 488 | 111 | 177 | 866 | 823 | 190 | 505 | A | A | A | A | A | G | A |
| S6_10371528 | C | T | 489 | 459 | 140 | 138 | 867 | 818 | 383 | 218 | C | C | C | C | C | C | C |
| S6_13439145 | G | A | 474 | 506 | 137 | 152 | 838 | 985 | 336 | 167 | G | G | G | G | G | G | G |
| S6_22794237 | A | G | 476 | 489 | 139 | 126 | 853 | 844 | 322 | 379 | A | A | A | A | A | A | A |
| S6_23132086 | A | G | 474 | 593 | 140 | 142 | 863 | 612 | 323 | 459 | A | A | A | A | A | A | A |
|  |  |  |  |  |  |  |  |  |  |  |  |  |  |  |  |  |  |
|  | **Allele** | | **LL_S_WS** | | **LL_S_DS** | | **UL_S_WS** | | **UL_S_DS** | | **Selected promising accessions** | | | | | | |
| **SNP id** | **Major** | **Minor** | **ME** | **me** | **ME** | **me** | **ME** | **me** | **ME** | **me** | **AUS 329** | **AUS 344** | **CHUNGUR BALI** | **DANGAR** | **LAL SAITA** | **PARA NELLU** | **SIMUL KHURI** |
| S6_27655258 | T | G | 479 | 470 | 139 | 213 | 853 | 593 | 314 | 981 | T | T | T | T | T | G | T |
| S6_28876857 | T | G | 457 | 502 | 102 | 192 | 819 | 891 | 200 | 498 | G | G | G | T | T | G | K |
| S6_31179920 | G | A | 473 | 587 | 133 | 325 | 853 | 725 | 323 | 289 | G | G | G | G | G | A | G |
| S7_13019061 | G | T | 471 | 0 | 129 | 0 | 859 | 0 | 242 | 0 | K | K | G | G | K | G | G |
| S7_16180595 | A | T | 477 | 0 | 142 | 0 | 858 | 0 | 330 | 0 | A | A | A | A | A | A | A |
| S7_16181810 | G | A | 477 | 579 | 142 | 171 | 857 | 335 | 326 | 708 | G | G | G | G | G | G | G |
| S7_16183053 | A | G | 477 | 578 | 142 | 76 | 857 | 0 | 326 | 113 | A | A | A | A | A | A | A |
| S7_19598023 | G | A | 486 | 335 | 144 | 59 | 849 | 907 | 337 | 110 | G | G | G | G | G | G | G |
| S7_20159780 | C | A | 474 | 486 | 136 | 148 | 840 | 884 | 213 | 621 | A | A | A | C | A | C | A |
| S8_466752 | G | A | 480 | 424 | 140 | 114 | 851 | 830 | 338 | 95 | G | G | G | G | G | G | G |
| S8_500943 | A | T | 479 | 423 | 140 | 118 | 851 | 883 | 339 | 95 | A | A | A | A | A | A | A |
| S8_541934 | G | A | 478 | 457 | 137 | 155 | 847 | 876 | 329 | 261 | G | G | G | G | G | G | G |
| S9_3032058 | T | C | 477 | 0 | 142 | 0 | 858 | 0 | 330 | 0 | T | T | T | T | T | T | T |
| S9_5467194 | C | T | 476 | 577 | 142 | 146 | 864 | 252 | 327 | 563 | C | C | C | C | C | C | C |
| S9_9426722 | A | G | 475 | 506 | 138 | 62 | 846 | 979 | 328 | 120 | A | A | A | A | A | A | A |
| S9_10118145 | G | A | 475 | 586 | 138 | 167 | 845 | 978 | 329 | 126 | G | G | G | G | G | G | G |
| S9_11072513 | G | A | 471 | 504 | 128 | 201 | 861 | 807 | 234 | 822 | A | A | A | A | A | G | A |
| S9_11791517 | C | T | 470 | 494 | 122 | 189 | 851 | 843 | 203 | 639 | T | T | T | T | T | C | T |
| S9_11838142 | C | T | 479 | 463 | 134 | 157 | 852 | 838 | 325 | 309 | C | C | C | T | C | C | T |
| S9_13423222 | G | A | 473 | 599 | 135 | 256 | 847 | 955 | 324 | 246 | G | G | G | G | G | G | G |
| S9_13934171 | C | T | 465 | 587 | 121 | 315 | 850 | 848 | 230 | 1260 | T | T | T | T | T | C | T |
| S9_15970207 | T | C | 466 | 584 | 131 | 218 | 849 | 849 | 289 | 688 | T | T | T | T | T | T | C |
| S9_16154337 | A | G | 480 | 452 | 143 | 97 | 853 | 804 | 342 | 113 | A | A | A | A | A | A | A |
| S9_19316065 | C | T | 491 | 144 | 144 | 26 | 852 | 803 | 336 | 16 | C | C | C | C | C | C | C |
[truncated: 36,353 more chars]
